# Demethylzeylasteral inhibits cell proliferation and enhances cell chemosensitivity to 5-fluorouracil in Colorectal Cancer cells

**DOI:** 10.7150/jca.44375

**Published:** 2020-08-19

**Authors:** Yang Yang, Jing Han, Yanlin Ma, Jianfeng Zhang, Zhenya Zhang, Guiying Wang

**Affiliations:** 1 Department of General Surgery, The Fourth Hospital of Hebei Medical University, Shijiazhuang, 050011, China.; 2 Department of Medical Oncology, The Fourth Hospital of Hebei Medical University, Shijiazhuang, 050011, China.; 3 The Fourth Hospital of Hebei Medical University, Shijiazhuang, 050011, China.; 4 The Third Hospital of Hebei Medical University, Shijiazhuang, 050011, China.

**Keywords:** Colorectal, Demethylzeylasteral, 5-Fluorouracil, Chemosensitivity

## Abstract

Malignant growth and chemotherapy resistance to 5-fluorouracil (5-FU) are the obstacles to the treatment of Colorectal cancer (CRC). There is need to develop effective therapeutic option. Demethylzeylasteral benefits to immune and anti-tumor function. However, the role demethylzeylasteral played in colorectal cancer remains unclear. Here, our study confirmed that demethylzeylasteral could inhibit the cell malignant capacity, such as proliferation, migration and invasion. And we also found demethylzeylasteral could cause cell cycle arrest and apoptosis. Followed we verified that combination demethylzeylasteral with 5-FU has a better curative effect *in vitro*. The two drugs function synergistically in SW480 and additionally in RKO. IC50 values of 5-FU decreased when combined with demethylzeylasteral. Next, we used the network pharmacology approach to explore the the potential molecular mechanism of demethylzeylasteral. We constructed the “Colorectal - targets - Demethylzeylasteral” and protein-protein interactions (PPI) networks. And 15 hub genes were found in PPI network. Then Gene Ontology (GO) enrichment analysis showed that demethylzeylasteral may affect cell cycle, apoptosis, invasion and response to chemotherapy drugs. Kyoto Encyclopedia of Genes and Genomes (KEGG) pathway analysis indicated demethylzeylasteral may be involved in many cancer-related pathways. Taken together, the network pharmacology approach provided a potential mechanism of demethylzeylasteral in colorectal cells. Our study indicated that demethylzeylasteral could exert anti-tumor effects and enhance the sensitivity of the Colorectal cells to 5-FU, suggesting a promising ability to serve as an anti-cancer agent in Colorectal cancer.

## Introduction

Colorectal cancer (CRC), one of the most common cancers worldwide, had caused a higher and higher morbidity and mortality each year 1-3. It had been reported that about 900000 people died every year 4. In China, 20.7 per 100,000 were projected to be diagnosed with colorectal in 2020 and the case-fatality ratio in China was 14.0 % 5. The treatment for colorectal was an enormous challenge.

Unfortunately, those about 20% patients were in the middle and late period when see a doctor complicated the treatment 6. Chemotherapy represented an essential treatment for patients who were in advanced stage 7. 5-Fluorouracil (5-FU), playing a critical role in regulating the cell cycle and apoptosis 8, had emerged as the front-line chemotherapeutic drug for colorectal cancer 9. However, many patients had no response to 5-FU, leading to recurrence or even worsened condition 10. Chemotherapy resistance to 5-FU became an issue urgently needed to be solved.

Demethylzeylasteral originally was thought to have immunosuppressive and anti-inflammatory effects 11. It was recently confirmed that demethylzeylasteral could inhibit angiogenesis as well as cell activity in many cancers, such as breast cancer, glioma, melanoma and so on 12-14. Furthermore, recent study evidence showed that demethylzeylasteral could enhance chemosensitivity to gemcitabine in human pancreatic cancer cells 15.

However, the influence of demethylzeylasteral on colorectal cell has remained unclear. In our study, we indicated that demethylzeylasteral could suppress the cell proliferation, migration and invasion in a dose dependent manner. The study on enhancing chemosensitivity to 5-FU was also at the heart. Then, the binding targets of demethylzeylasteral were predicted to explore the mechanism. We got the intersection genes of binding targets and colorectal cancer-related genes. These genes were used to construct the “Colorectal - targets - Demethylzeylasteral” and PPI networks. In addition, 15 hub genes in the PPI network had been found. Followed GO and KEGG pathway analyses revealed us the potential mechanism of Demethylzeylasteral. Our study showed that the combination demethylzeylasteral with 5-FU could achieve a better therapeutic effect.

## Materials and Methods

### Cell Culture

The RKO was maintained in RPMI-1640 complemented with 10% fetal bovine serum. The SW480 was complemented in DMEM (Dulbecco's Modified Eagle Medium) containing 10% fetal bovine serum. All cell lines were cultured at 37℃ in 5% CO2. When the cells proliferated to 80% confluence, the cells were digested with trypsin, centrifuged, washed with aseptic PBS twice and plated on the cell petri dish. The cells in the logarithmic proliferative phase were taken for the experiment.

### Western Blotting analysis of protein

Western Blotting assays were performed as previously described 16.

### Proliferation assay

The cells were plated on 96 wells (5000 cells per well) when the cells were logarithmic phase. The absorbance was examined 0, 24, 48, 72 and 96 h after inoculation by MTS assay.

### Colony formation assay

The cells were plated on 24 wells (20000 cells per well) when the cells were logarithmic phase. After 2 days, the cells were fixed with methanol for 5 minutes, stained with crystal violet for 5 minutes, and the number of cells was observed.

### Transwell analysis

Cells were plated on upper chamber of transwell (300000 cells per well) when the cells were logarithmic phase. After 36 hours, the chamber was fixed in methanol for 5 minutes and stained with crystal violet for 5 minutes. The number of cells passing through Transwell chambers was observed under microscope.

### Cell cycle analysis

After the cells were treated for the day ahead (centrifuged, washed, re- centrifuged, and fixed with 70% precooled ethanol), the cells were placed in the-20 refrigerator overnight. The next day, the cells were centrifuged, washed, centrifuged again, incubated in dark for 15 minutes with PI (BD Biosciences, San Jose, CA, USA) reagent and were detected by upflow cytometry.

### Apoptosis detection

Cells were digested by trypsin, centrifuged, washed, centrifuged again and a series of operations are carried out according to the instructions of apoptosis kit (Neo Bioscience, Beijing, China). The cells were detected by upflow cytometry last.

### Combined effect analysis

The drug-drug interaction coefficient (coefficient of drug interaction, CDI) was used to evaluate the drug-drug interaction 17. If CDI < 1, it was proved that the action of the two drugs was synergistic, and when CDI < 0.7, the synergistic effect of the two drugs was very significant; if CDI = 1, the action of the two drugs was additive; if CDI > 1, the action of the two drugs was antagonistic.

### Data collection and processing

The 3D molecular structure of demethylzeylasteral was got from PubChem database (https://pubchem.ncbi.nlm.nih.gov). The binding sites of demethylzeylasteral were predicted by PharmMapper database (http://www.lilab-ecust.cn/pharmmapper/). The sequencing data of Colorectal normal tissues and tumor tissues were downloaded from The Cancer Genome Atlas database (TCGA, https://portal.gdc.cancer.gov/). The colorectal related genes and predicted binding genes of demethylzeylasteral were inputed into jvenn (http://jvenn.toulouse.inra.fr/app/example.html) to achieve the intersection genes. The “Colorectal - targets - Demethylzeylasteral” network was constructed by using Cytoscape software and PPI network was constructed by using STRING database (https://string-db.org). The hub genes of PPI network were found by using Cytoscape software (according to the degree algorithm). The GO and KEGG pathway analyses were carried out by using DAVID 6.8 database (https://david.ncifcrf.gov). The Bubble charts were made by Rstudio 3.6.2 software. The cox regression analysis of the survival rate of these hub genes in CRC through Oncolnc database (http://www.oncolnc.org/).

### Statistical analysis

The experimental results were analyzed by Student's t-test (unpaired, two tailed). *P*<0.05 were considered to be significant. All statistical analyses were performed using Prism5 (GraphPad Software Inc., La Jolla, CA).

## Results

### Demethylzeylasteral inhibited the colorectal cell malignancy

**Figure 1:**
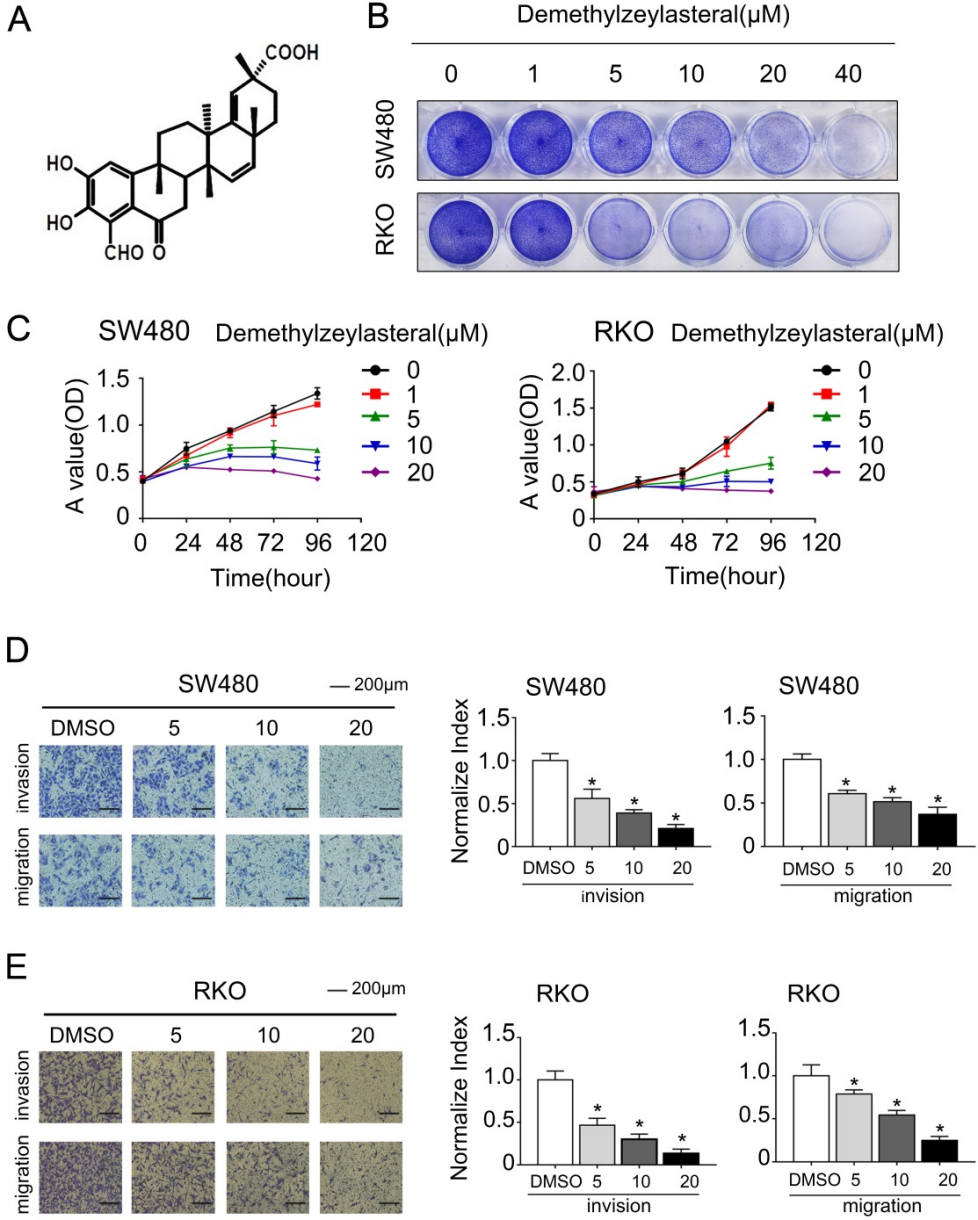
** The effect of Demethylzeylasteral on the growth, invasion and migration of colorectal cancer cells. A.** The chemical structural formula of demethylzeylasteral. **B.** SW480 and RKO were treated for 48 hours by Demethylzeylasteral of gradient concentrations. Colony formation experiments had been performed. The darker the colour, the more the cells. **C.** SW480 and RKO were treated by the concentration gradient of demethylzeylasteral. The cell viability was measured by MTS assay. Statistics of MTS experiments of SW480 and RKO were shown in Table S1. **D and E.** SW480 and RKO were treated by demethylzeylasteral of gradient concentrations. The invasion and migration activity were detected by transwell experiments. The right is the statistical chart. *P < 0.001.

To investigate whether Demethylzeylasteral affects the cell viability, we treated colorectal cells by demethylzeylasteral of gradient concentrations. Its molecular structure was shown in Figure 1A. We found that demethylzeylasteral inhibited the proliferation of colorectal cells, showing a dose-dependent manner (Figure 1B). Then the MTS assays were used to verify this result. With the dosage increasing, the proliferation and growth of cells were inhibited (Figure 1C). Moreover, we also found that the higher the dose, the weaker the invasive and migrate ability (Figure 1D, E). All these indicated that demethylzeylasteral could inhibit the proliferation, invasion and migration of colorectal cells.

### Demethylzeylasteral inhibited the cell proliferation by causing cell cycle arrest and apoptosis

**Figure 2:**
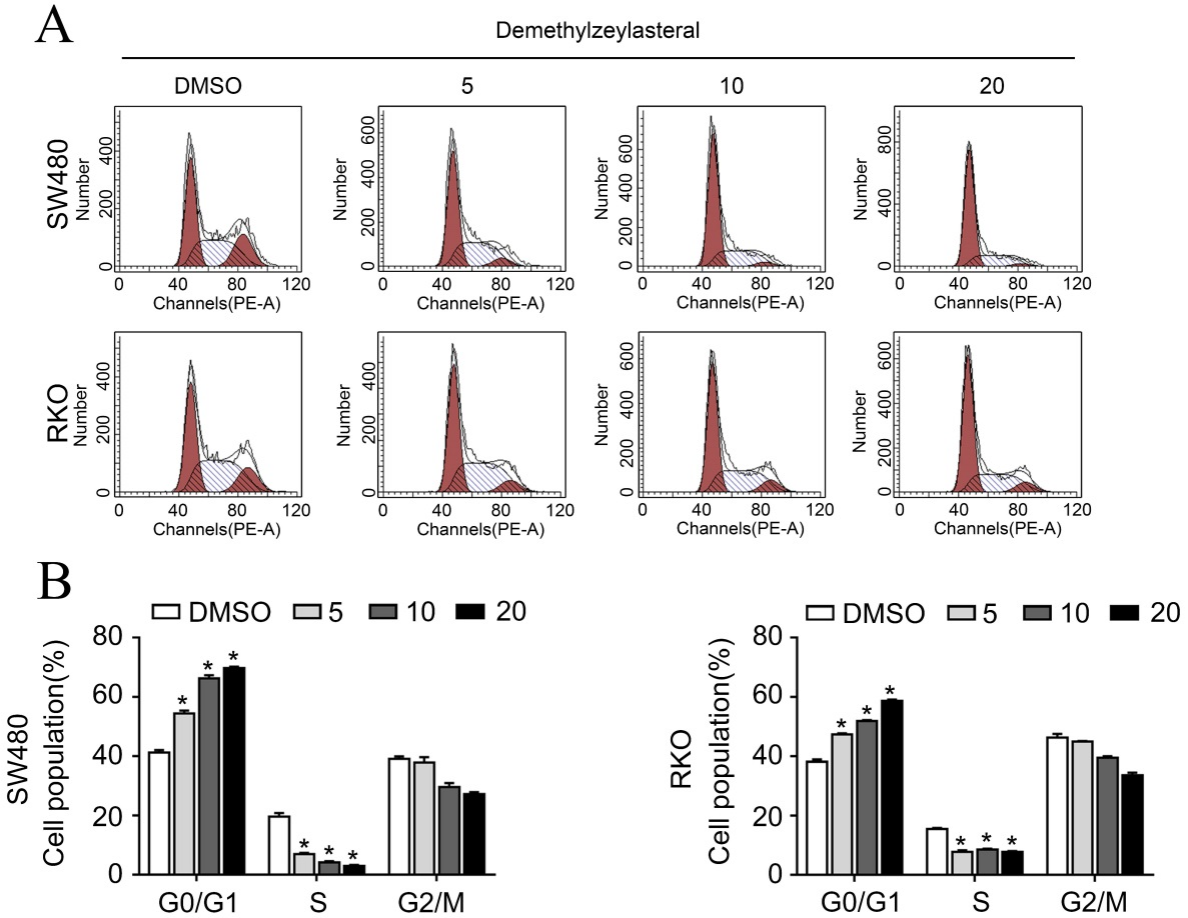
** The effect of demethylzeylasteral on the cell cycle of colorectal cancer cells. A.** SW480 and RKO were treated for 24 hours by demethylzeylasteral of gradient concentrations. Cell cycle was detected by flow cytometry. **B.** Statistical analysis on chart A.

**Figure 3:**
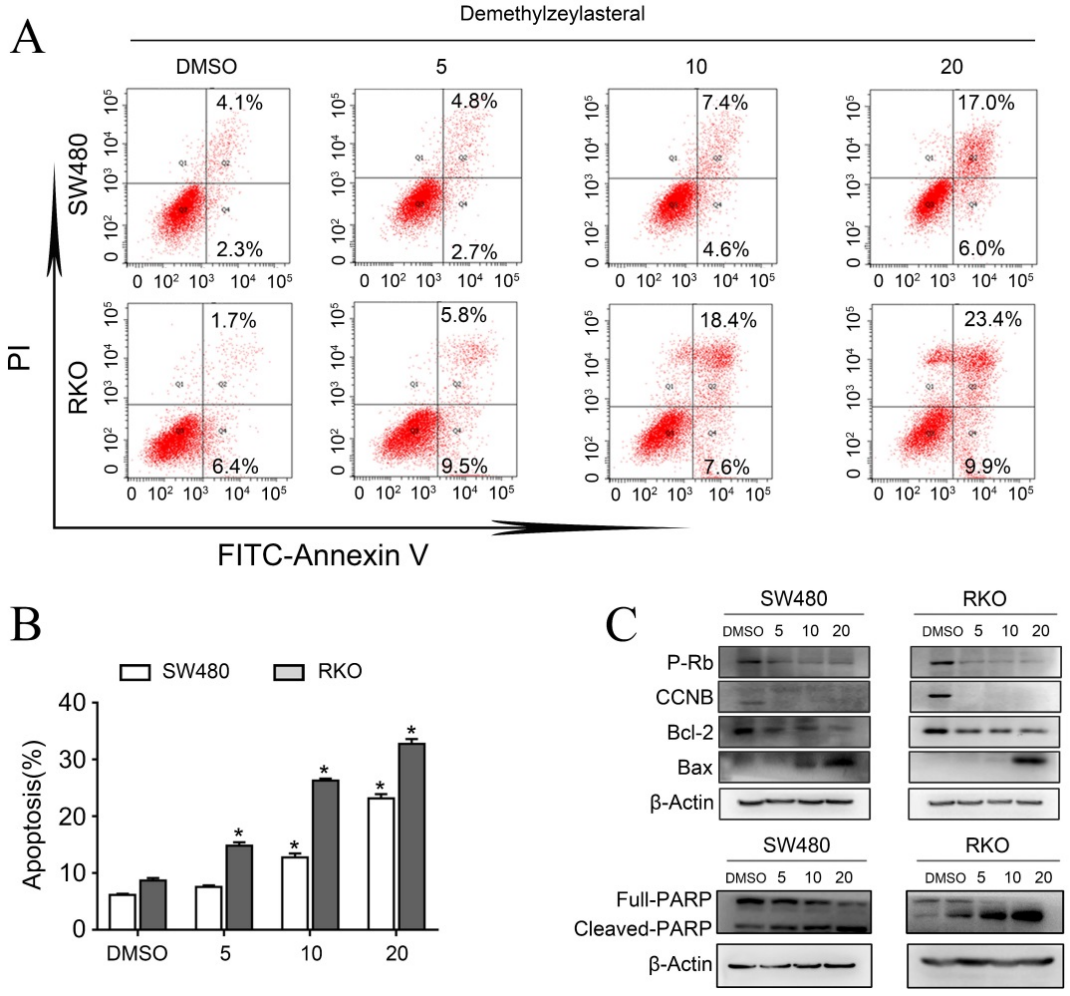
** The effect of demethylzeylasteral on the apoptosis of colorectal cancer cells. A.** SW480 and RKO were treated for 24 h by demethylzeylasteral of gradient concentrations. Apoptosis was detected by flow cytometry. **B.** Statistical chart of A chart. **C.** SW480 and RKO were treated for 24 h by demethylzeylasteral of gradient concentrations. The protein expression of BAX, BCL-2, CCNB, P-Rb and Cleaved-PARP were detected by Western blot. *P < 0.001.

In order to clarify the mechanism of its effect on the proliferation of colorectal cancer cells, we examined that whether demethylzeylasteral could affect the cell cycle arrest and apoptosis using the flow cytometry. What could be clearly seen was that the cell number in the G0/G1 phase increased, whereas decreased in S and G2/M phase in a dose-dependent manner (Figure 2A, B). Previously, it had been reported that cell cycle related proteins express in different periods. For example, P-Rb and CCNB proteins tend to be highly expressed in S or G2/M phase 18, 19. As we supposed, compared with the control group, the protein levels of P-Rb, CCNB were lower in drug treatment group (Figure 3C). As was showed in Figure 3A, with the increase of drug concentration, the apoptotic rate increased gradually. The apoptotic rate of control group was 6.4% in SW480; however, it was 23% when the drug concentration was 20 μM. Similar results were achieved in the RKO (Figure 3A, B). In addition, the apoptosis related protein levels of BCL-2, BAX and Cleaved-PARP were monitored by Western blot. After treatment with demethylzeylasteral, the protein expression of Cleaved-PARP, BAX were enhanced, and the level of BCL-2 was inhibited (Figure 3C). These results showed that the cell cycle arrest and apoptosis were involved in the cell proliferation inhibited by demethylzeylasteral.

### Demethylzeylasteral enhanced the chemosensitivity of human CRC cells to 5-FU

**Figure 4:**
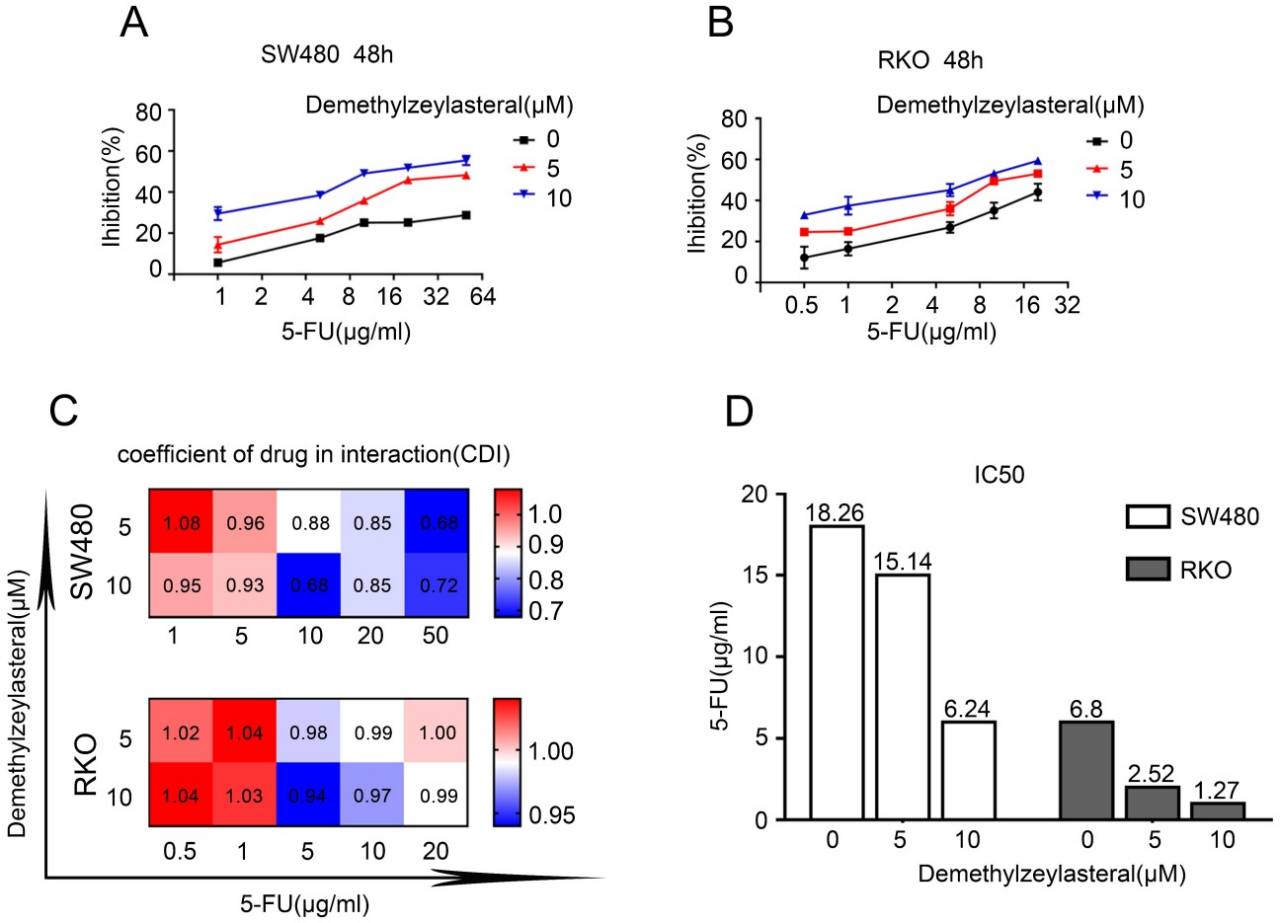
** Demethylzeylasteral enhanced sensitivity of Colorectal cancer cells to 5-FU. A and B.** SW480 and RKO were treated by demethylzeylasteral and 5-FU of gradient concentrations. The inhibition rates of colorectal cancer cells were tested by MTS assay. **C.** SW480 were treated for 60 h by demethylzeylasteral and 5-FU of gradient concentrations. RKO were treated for 24 h by demethylzeylasteral and 5-FU of gradient concentrations. The interaction coefficient of the two drugs was calculated. CDI > 1 antagonism, CDI = 1 addition, CDI < 1 synergism. **D.** SW480 were treated for 90 h by demethylzeylasteral and 5-FU of gradient concentrations. RKO were treated for 60 hours by demethylzeylasteral and 5-FU of gradient concentrations. The IC50 values of 5-FU were calculated when combined with demethylzeylasteral.

As explained in the introduction, it was clear that 5-FU was the first choice for treatment of colorectal cancer. Therefore, we supposed that it may be more effective than single drug, when combined 5-FU with Demethylzeylasteral. Interesting, the combination 5-FU with Demethylzeylasteral demonstrated a higher inhibition rate compared with 5-FU monotherapy (Figure 4A, B). Furthermore, the inhibition rates of combination were significantly higher when demethylzeylasteral concentration increased. Notably, the most CDI values were below 1 in SW480 when combined 5-FU with demethylzeylasteral, showing they function synergistically. And the most CDI values were close to 1 in RKO, suggesting they function additionally (Figure 4C). From Figure 4D, we could draw a conclusion that Demethylzeylasteral brought down the IC50 value of 5-FU. So we could get that Demethylzeylasteral enhance the chemosensitivity to 5-FU.

### The effect of 5-FU combined with demethylzeylasteral was more dramatic

**Figure 5:**
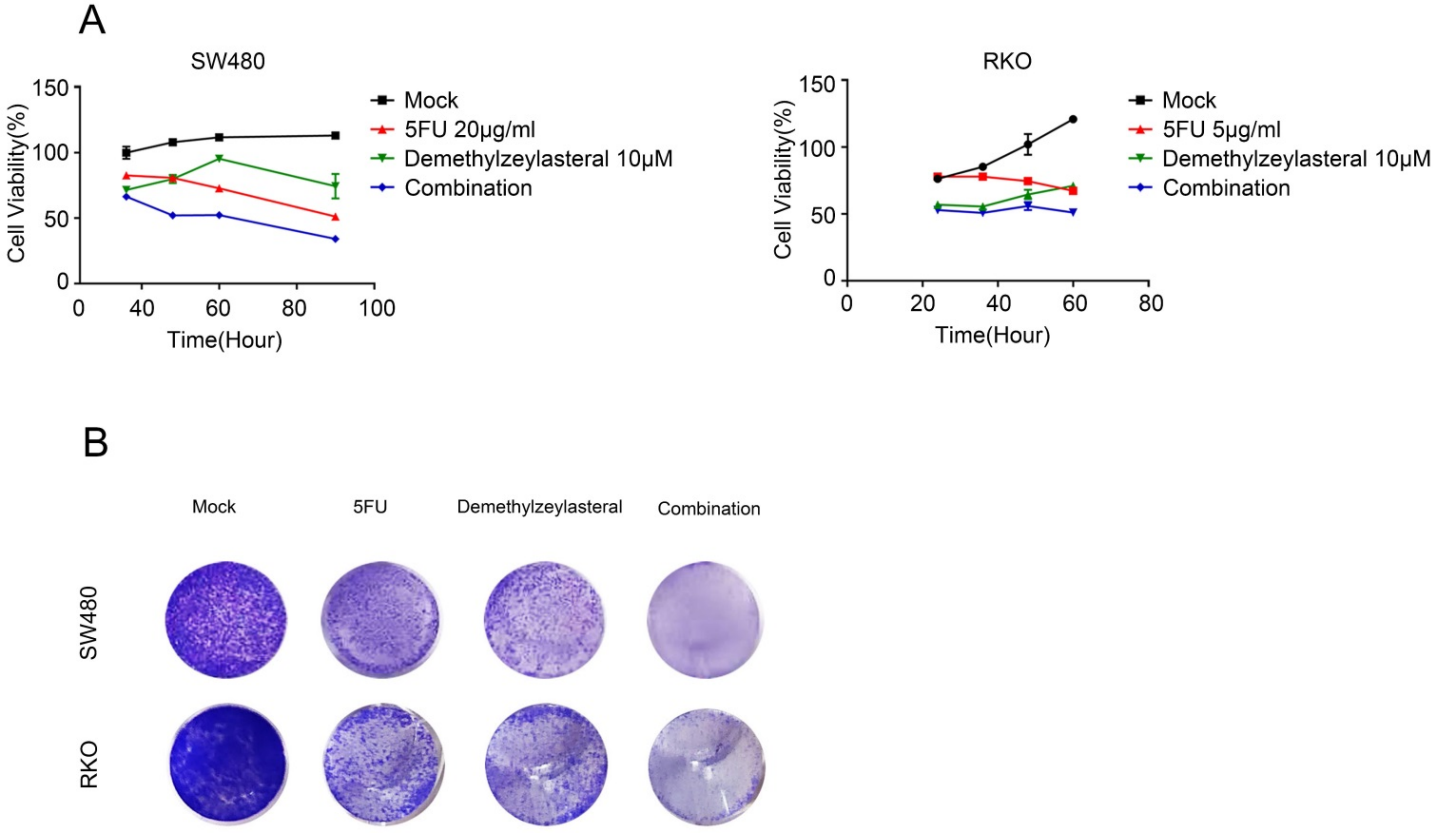
** Combined therapeutic effect of demethylzeylasteral and 5-FU on colorectal cancer cells.** SW480 and RKO were treated with demethylzeylasteral and 5-FU. The concentration of 5-FU was 20 µg/ml in SW480, while it was 5 µg/ml in RKO. The cell toxicity was tested by MTS assay. B. SW480 and RKO were treated with demethylzeylasteral and 5-FU. Colony formation experiments had been performed.

In order to verify whether the combination of the two drugs could really work better, we selected the appropriate concentration for the experiment. Figure 5A revealed that there was a gradual decrease in the cell number that was treated with 5-FU and demethylzeylasteral. The evidences confirmed that demethylzeylasteral could enhance the anti-tumor effect of 5-FU in Colorectal cancer cells. The same result was verified by the clony experiment (Figure 5B). It can be seen that there was a better effect when the two drugs were combined.

### The network pharmacological relationship between demethylzeylasteral and colorectal cancer

**Figure 6:**
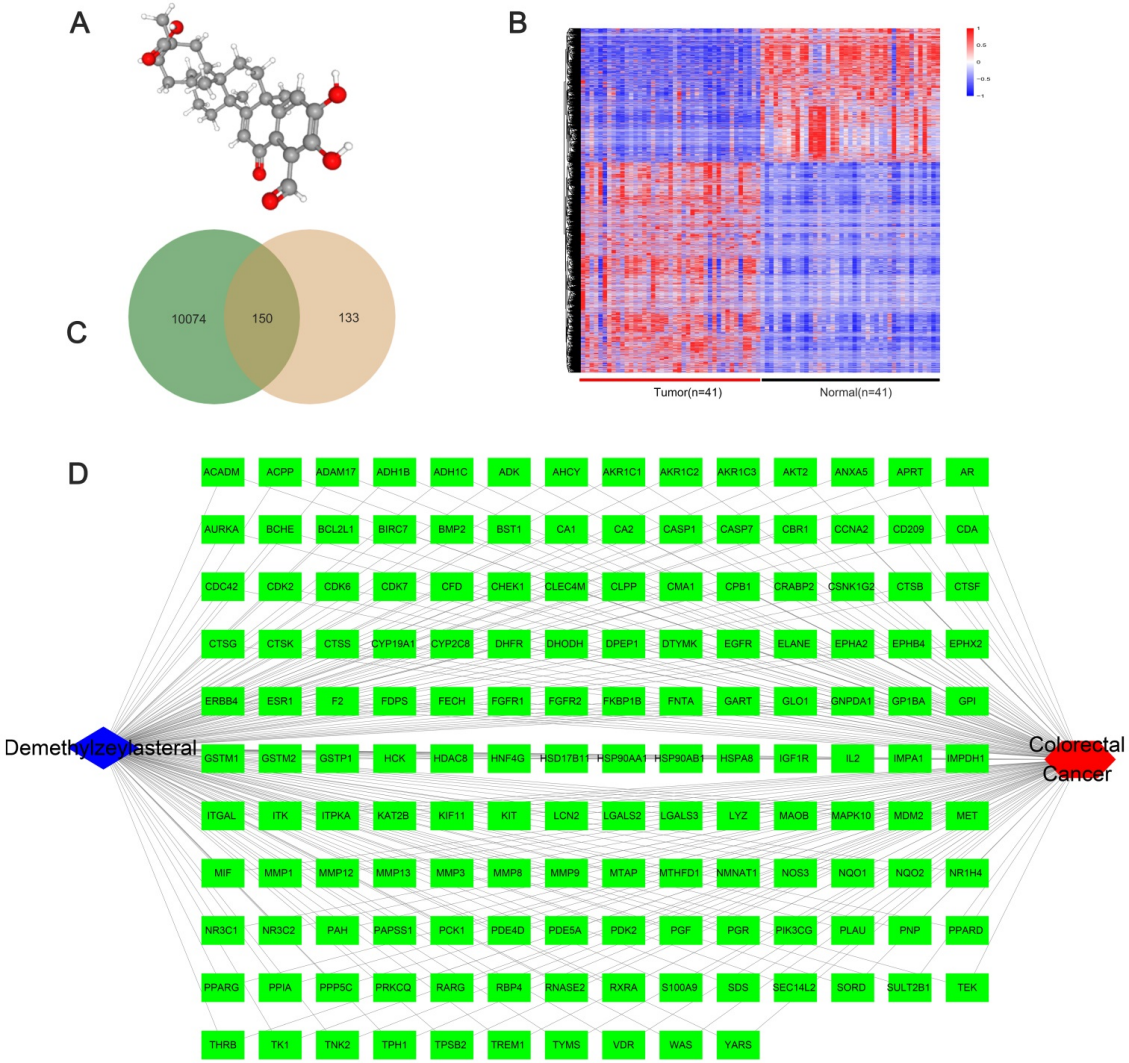
** The network pharmacological relationship between demethylzeylasteral and colorectal cancer. A.** The 3D molecular structure of Demethylzeylasteral was derived from PubChem database. **B.** The heat map of differentially expressed genes in colorectal cancer tissue and normal tissue from TCGA database. **C.** A Venn diagram was constructed to show the relationship between colorectal cancer and demethylzeylasteral. Green colour represented colorectal cancer-related genes, and pink colour represented predicted binding genes. **D.** The “Colorectal - targets - Demethylzeylasteral” network.

**Figure 7:**
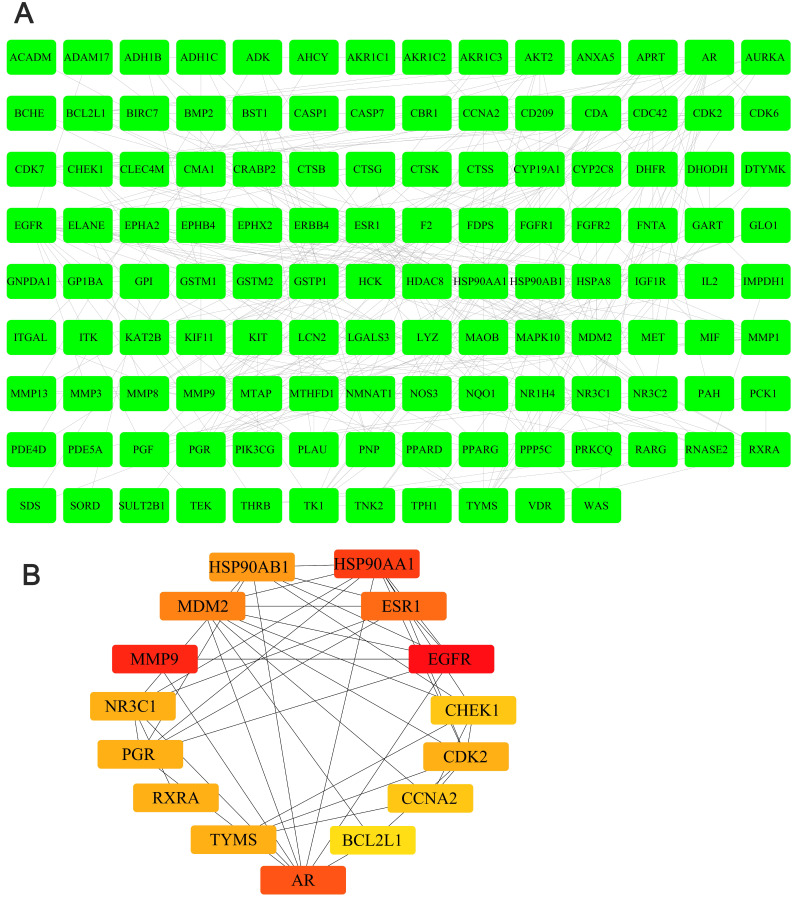
** The PPI analysis and hub genes in PPI network. A.** The PPI analysis of 150 intersection genes from figure 6C (interaction score > 0. 7). **B.** The hub genes in PPI network.

We obtained the 3D structure of demethylzeylasteral from PubChem database (Figure 6A). A total of 283 possible binding sites of demethylzeylasteral were predicted by PharmMapper database (Table S2). At the same time, we searched for a total of 10224 genes related to Colorectal cancer through TCGA database, which were differentially expressed genes between normal and neoplastic tissues (fold change > 1.4 or < 0.7, *P* < 0.05) (Table S3). Compared with normal tissues, 6227 genes were up-regulated and 3997 genes were down-regulated in cancer tissues (Figure 6B). As shown in Figure 6C, we had a total of 150 genes which were associated with demethylzeylasteral and Colorectal cancer by constructing a Venn diagram (Table S4). We used cytoscape software to visualize the “Colorectal - targets - Demethylzeylasteral” network (Figure 6D). A PPI was analyzed by using String database. (Figure 7A, Table S5). In the PPI network, we found 15 hub genes (Figure 7B, Table S6). These genes may play an important role in the regulation of the whole PPI network. For example, EGFR, CCNA2 and CDK2 played important roles in cell cycle. Or MDM2 and BCL2L1 could affect the cell apoptosis. Morever, we also carried out the analysis of the survival rate of these hub genes in CRC through Oncolnc database. The cox regression results were shown in Figure S1. The genes, including AR, BCL2L1, EGFR, ESR1, were correlated negatively with overall survival. However, CCNA2, CHEK1, MDM2 played the opposite role.

### The GO and KEGG pathway enrichment analysis of 150 genes related to demethylzeylasteral and Colorectal cancer

**Figure 8:**
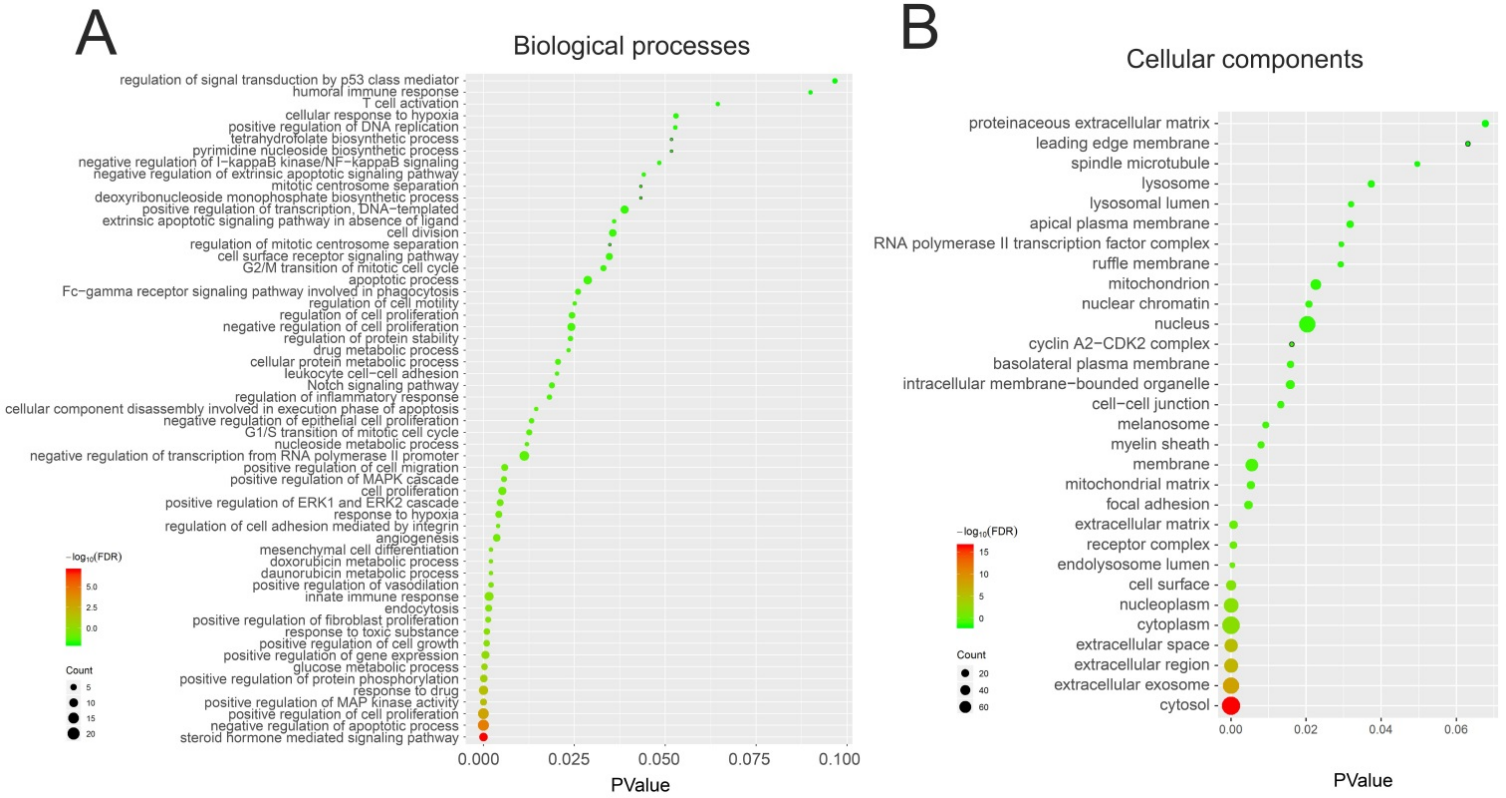
** The GO analysis of 150 genes related to Demethylzeylasteral and Colorectal cancer. A.** Biological processes. **B.** Cellular components.

**Figure 9:**
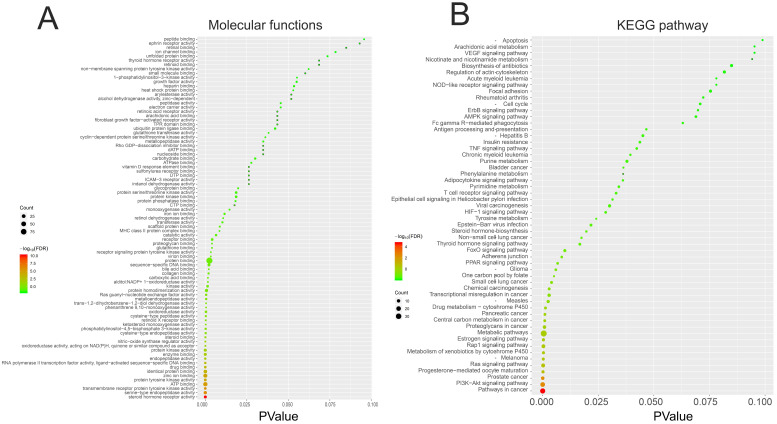
** The GO and KEGG pathway enrichment analysis of 150 genes related to Demethylzeylasteral and Colorectal cancer. A.** Molecular functions. **B.** KEGG pathway analysis.

We performed GO and KEGG pathway enrichment analysis of these 150 genes by using DAVID database. The results showed that these 150 genes were mainly related to cytosol, extracellular region, cell surface, cell-cell junction, nuclear chromatin, mitochondrion, lysosome, or spindle microtubule (Figure 8B, Table S7). At the same time, they had the function of receptor, enzyme activity and so on (Figure 9A, Table S8). They were involved in the regulation of apoptotic process, regulation of cell proliferation, response to drug, glucose metabolic, immune response or regulation of inflammatory response (Figure 8A, Table S9). KEGG pathway analysis showed that these genes were enriched in many cancer-related pathways, such as PI3K-Akt signaling pathway, Ras signaling pathway, metabolic pathways, cell cycle, VEGF signaling pathway, apoptosis and so on (Figure 9B, Table S10). This suggested that demethylzeylasteral may affect colorectal cancer through these pathways. This provided a theoretical basis for our future research.

## Discussion

Demethylzeylasteral was firstly found to have an effect on immunosuppressive and anti-inflammatory. Previous studies also had shown that demethylzeylasteral was involved in anti-cancer. Up to now, demethylzeylasteral had been reported to have an anti-cancer effect in breast cancer, glioma and so on. However, there was no study that demethylzeylasteral had an anti-cancer effect in colorectal. In our study, we indicated that demethylzeylasteral could inhibit cell viability in dose-dependent manner. Forever, demethylzeylasteral was found to cause cell cycle arrest and apoptosis in colorectal. Our research showed that demethylzeylasteral may have the potential to be applied to clinical trials in the future.

Colorectal was one of the most common cancers in China. In recent decades, 5-FU had been used as a first-line treatment for colorectal cancer 20. Nevertheless, when used as a single drug, the non-response rate for 5-FU was about 50% for metastatic Colorectal cancer patients 21, 22. Hence, it was crucial to enhance chemosensitivity of 5-FU.

5-FU and gemcitabine belong to Pyrimidine analogues and could cause the cell apoptosis. Also, a present study indicated that Demethylzeylasteral could enhance PC cells' chemosensitivity to gemcitabine. But it was unknown that whether demethylzeylasteral could enhance the coloerectal cells' chemosensitivity to 5-FU. Based on these, we supposed that demethylzeylasteral may potentiate the activity of 5-FU. Followed, we verified that demethylzeylasteral can decrease the IC50 value of 5-FU and showed the combination demethylzeylasteral and 5-FU could achieve a better therapeutic effect, compared with single drug. Here, we carried out for the first time that demethylzeylasteral could enhance the coloerectal cells' chemosensitivity to 5-FU.

In order to explore the potential mechanism of the effect of demethylzeylasteral on colorectal cancer cells, we predicted the possible target genes of demethylzeylasteral and got the genes related to colorectal cancer. Followed, we took the intersection of the above two types of genes. We thought that these intersection genes may play important roles in the mechanism of demethylzeylasteral on colorectal cancer cells. To more intuitively show the relationship among these intersection genes, demethylzeylasteral and colorectal cancer, we carried out a “Colorectal - targets - Demethylzeylasteral” network. After that, the PPI network of these intersection genes was constructed through String database and 15 hub genes were found (EGFR, MMP9, HSP90AA, AR, ESR1, MDM2, HSP90AB1, TYMS, CDK2, RXRA, PGR, NR3C1, CCNA2, CHEK1, BCL2L1). Many genes were related to overall survival. We believed that these 15 genes were key points in the regulation of the whole PPI network. We found that some genes were related to the proliferation and apoptosis of Colorectal cancer, such as EGFR, CDK2, CCN2 and BCL2L1 23-26, and some were related to the prediction of chemotherapy for colorectal cancer, such as EGFR, TYMS 27, 28. Others, such as MMP9 29, were associated with the metastasis of colorectal cancer. These phenomena were consistent with our experimental results and our pathway analysis. As our experimental results shown, demethylzeylasteral could promote apoptosis, inhibit proliferation and inhibit metastasis. GO enrichment analysis showed that these genes may affect cell cycle, apoptosis and invasion. KEGG pathway analysis showed that many cancer-related pathways were enriched. Among them, the PI3k-AKT pathway (ranked first in KEGG pathways) was officially the downstream pathway of EGFR 30 (ranked first in hub genes). In addition, many previous studies had reported that demethylzeylasteral were associated with proliferation, apoptosis and invasion in other cancers, which were consistent with the function of demethylzeylasteral verified by us in Colorectal cancer.

Finally, we carried out GO and KEGG pathway analysis. GO enrichment analysis showed that these genes may affect cell cycle, apoptosis and invasion, in turn verifying our experimental results. KEGG pathway analysis showed that many cancer-related pathways were enriched. This provided a theoretical basis for our future research.

Now, we had noted the key role that demethylzeylasteral played in colorectal cells. Further research would be conducted to verify that whether a consistent result could be observed *in vivo*. In addition, it was essential to explore the toxicity of demethylzeylasteral *in vivo*. Our study may represent a new therapeutic strategy to overcome the chemotherapeutic resistance in CRC.

## Supplementary Material

Supplementary figure and tables.

## Abbreviations

CRC: Colorectal cancer
5-FU: 5-fluorouracil
GO: Gene Ontology
KEGG: Kyoto Encyclopedia of Genes and Genomes
TCGA: The Cancer Genome Atlas database
