# Supplementary material for: Demethylzeylasteral inhibits cell proliferation and enhances cell chemosensitivity to 5-fluorouracil in Colorectal Cancer cells
Source: J Cancer. 2020 Aug 19;11(20):6059–69. doi: 10.7150/jca.44375 (PMC7477418; doi:10.7150/jca.44375)
Supplement: Supplementary file 1 — Supplementary figure and tables. [file jcav11p6059s1.pdf]

Figure 1S

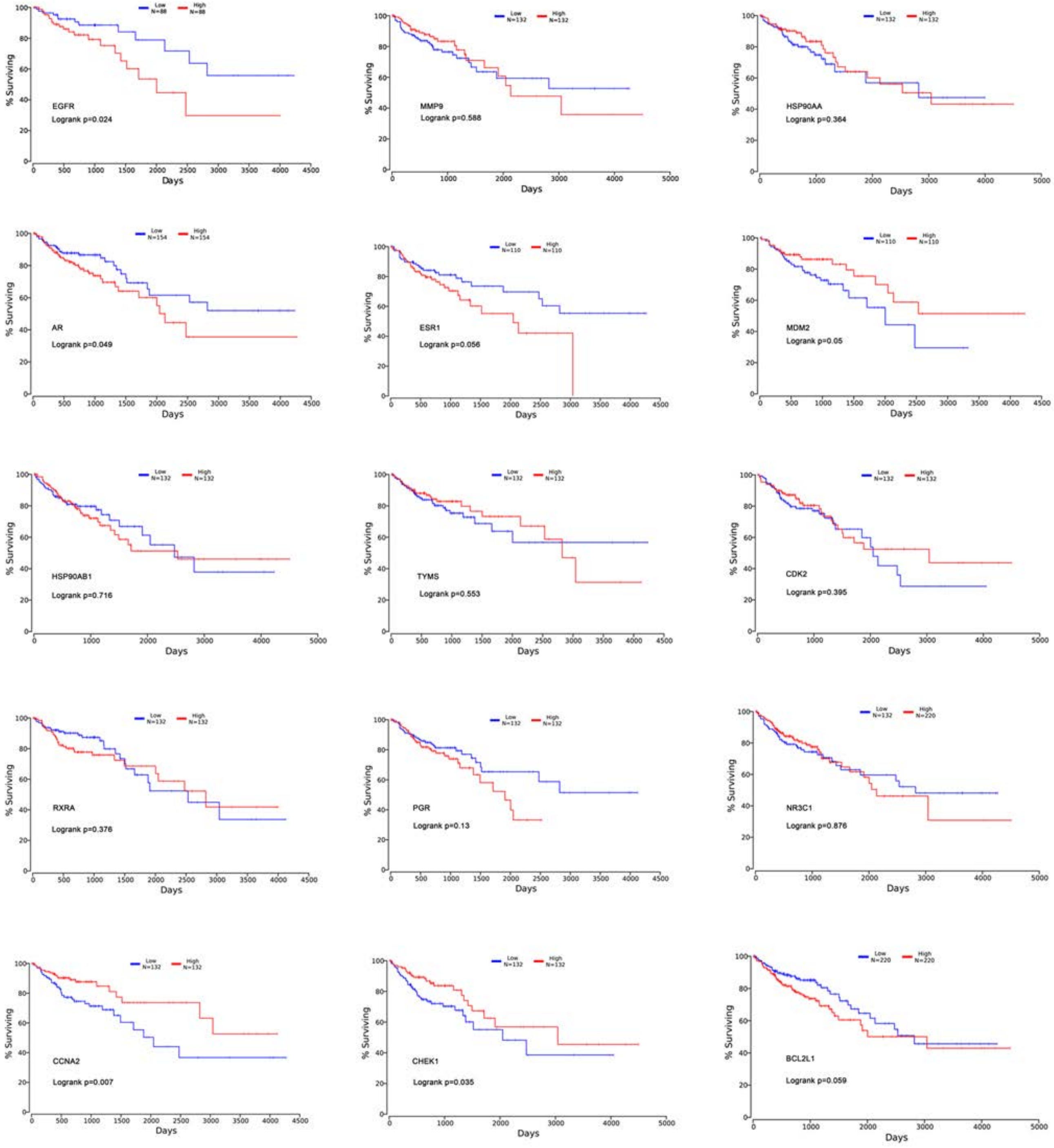

Low N=88
High N=88
EGFR
Logrank p=0.024
Low N=132
High N=132
MMP9
Logrank p=0.588
Low N=132
High N=132
HSP90AA
Logrank p=0.364
Low N=154
High N=154
AR
Logrank p=0.049
Low N=110
High N=110
ESR1
Logrank p=0.056
Low N=110
High N=110
MDM2
Logrank p=0.05
Low N=132
High N=132
HSP90AB1
Logrank p=0.716
Low N=132
High N=132
TYMS
Logrank p=0.553
Low N=132
High N=132
CDK2
Logrank p=0.395
Low N=132
High N=132
RXRA
Logrank p=0.376
Low N=132
High N=132
PGR
Logrank p=0.13
Low N=132
High N=220
NR3C1
Logrank p=0.876
Low N=132
High N=132
CCNA2
Logrank p=0.007
Low N=132
High N=132
CHEK1
Logrank p=0.035
Low N=220
High N=220
BCL2L1
Logrank p=0.059
% Surviving
Days

Table 1S Statistics of MTS experiments of SW480 and RKO

		SW480(Compared with 0μmol)				RKO(Compared with 0μmol)			
Time		1	5	10	20	1	5	10	20
24h	t	2.133	2.115	5.496	5.538	0.8428	1.169	1.832	1.614
	p	0.0769	0.0788	0.0015	0.0015	0.4315	0.2868	0.1167	0.1576
48h	t	0.7661	9.239	15.24	28.72	0.0354	3.13	5.1	5.718
	p	0.4727	<0.0001	<0.0001	<0.0001	0.9729	0.0203	0.0022	0.0012
72h	t	0.7101	8.088	14.36	19.06	1.079	19.83	14.57	54.43
	p	0.5043	0.0002	<0.0001	<0.0001	0.3222	<0.0001	<0.0001	<0.0001
96h	t	3.514	19.3	16.05	27.52	1.518	16.52	40.25	49.83
	p	0.0126	<0.0001	<0.0001	<0.0001	0.1798	<0.0001	<0.0001	<0.0001

Ligand:
Table 2S 10322911

GENEsymbl	Pharma Mc	Num Featu	Fit	Norm Fit	zscore	Num Hydrc	Num HB Ac	Num HB Dc	Num Posit	Num Negat	Num Aroma	Name	Class	Uniplot	Function	Disease	Idication
ITGAL	1xdd_v	3	2.99	0.9967	1.14692	3	0	0	0	0	0	Integrin	IMMUNE SY	ITAL_HUMA	Integrin	NONE	NONE
BMP2	1reu_v	3	2.989	0.9964	0.910619	3	0	0	0	0	0	Bone morp	HORMONE/C	BMP2_HUMA	Induces c	NONE	Bone dise
AKR1C2	1j96_v	3	2.94	0.98	1.02408	3	0	0	0	0	0	Aldo-keto	OXIDOREDU	AK1C2_HUM	Works in	NONE	NONE
PGR	2ovm_v	3	2.937	0.9789	1.05785	2	1	0	0	0	0	Progester	TRANSCRIP	PRGR_HUMA	The sterc	NONE	Uterine f
GSTP1	1lbk_v	3	2.931	0.9772	1.01928	1	2	0	0	0	0	Glutathio	TRANSFERA	GSTP1_HUM	Conjugati	NONE	NONE
MAPKAPK2	2p3g_v	3	2.922	0.9741	0.923294	3	0	0	0	0	0	MAP kinas	NONE	P49137	Involved	NONE	NONE
CA2	1okl_v	3	2.9	0.9666	0.922467	2	1	0	0	0	0	Carbonic	LYASE	CAH2_HUMA	Essential	Defects i	NONE
TTR	1ijn_v	3	2.899	0.9663	0.914745	2	0	1	0	0	0	Transthyr	TRANSPORT	TTHY_HUMA	Thyroid h	Defects i	NONE
ALB	1e7e_v	3	2.869	0.9565	0.825613	3	0	0	0	0	0	Serum alb	PLASMA PR	ALBU_HUMA	Serum alb	Defects i	NONE
STS	1p49_v	3	2.868	0.9559	0.760295	3	0	0	0	0	0	Steryl-su	HYDROLASE	STS_HUMAN	Conversio	Defects i	Cognitive
APOA2	1l6l_v	3	2.848	0.9494	0.736608	3	0	0	0	0	0	Apolipopr	LIPID TRA	APOA2_HUM	May stabi	NONE	NONE
GC	1j78_v	4	3.778	0.9444	2.48627	4	0	0	0	0	0	Vitamin D	TRANSPORT	VTDB_HUMA	Multifunc	NONE	NONE
ANG	1k59_v	4	3.743	0.9358	3.76049	0	4	0	0	0	0	Angiogeni	HYDROLASE	ANGI_HUMA	May funct	Defects i	NONE
BCHE	1p0i_v	3	2.803	0.9345	0.662629	1	2	0	0	0	0	Cholinest	HYDROLASE	CHLE_HUMA	NONE	Defects i	Neurotoxi
HSD17B11	1yb1_v	3	2.801	0.9337	0.65998	2	0	1	0	0	0	Estradiol	OXIDOREDU	DHB11_HUM	Can conve	NONE	Prostate
CASP3	3dej_v	3	2.785	0.9284	0.701773	2	1	0	0	0	0	Caspase-3	HYDROLASE	CASP3_HUM	Involved	-Gly-217	NONE
CASP7	1shj_v	3	2.773	0.9245	0.452576	3	0	0	0	0	0	Caspase-7	HYDROLASE	CASP7_HUM	Involved	-Gly- 217	NONE
PIM1	2obj_v	3	2.766	0.922	0.579684	2	1	0	0	0	0	Proto-onc	NONE	P11309	Involved	NONE	For nutri
CLPP	1tg6_v	3	2.76	0.92	0.636951	2	0	1	0	0	0	Putative	HYDROLASE	CLPP_HUMA	Clp cleav	NONE	NONE
CA1	1czm_v	3	2.745	0.9149	0.640749	1	1	1	0	0	0	Carbonic	LYASE(OXC	CAH1_HUMA	Reversibl	NONE	NONE
BACE1	3exo_v	3	2.744	0.9146	0.577274	2	0	1	0	0	0	Beta-secr	NONE	P56817	Involved	NONE	NONE
ESR1	1qku_v	3	2.733	0.911	0.5315	2	0	1	0	0	0	Estrogen	NUCLEAR R	ESR1_HUMA	Nuclear h	NONE	Melanoma;
AKR1B1	2agt_v	3	2.717	0.9057	0.621009	2	1	0	0	0	0	Aldose re	OXIDOREDU	ALDR_HUMA	Catalyzes	In diabet	Neuropath
PPIA	1w8l_v	3	2.679	0.8929	0.257317	2	1	0	0	0	0	Peptidyl-	NONE	P62937	Posttrans	NONE	For treat
#N/A	3hvc_v	3	2.626	0.8754	0.281576	3	0	0	0	0	0	NONE	NONE	NONE	NONE	NONE	NONE
PDE4B	1xn0_v	3	2.583	0.8609	0.303081	0	2	1	0	0	0	cAMP-spec	HYDROLASE	PDE4B_HUM	May be in	NONE	Chronic c
AKR1C3	1xf0_v	3	2.567	0.8555	0.263055	2	1	0	0	0	0	Aldo-keto	OXIDOREDU	AK1C3_HUM	Catalyzes	NONE	NONE
THRB	2pin_v	3	2.562	0.8539	-0.243333	3	0	0	0	0	0	Thyroid h	NONE	P10828	Nuclear h	NONE	NONE
GSR	1xan_v	4	3.371	0.8428	1.92828	2	1	1	0	0	0	Glutathio	NONE	P00390	RNA proce	NONE	For nutri
#N/A	1rs0_v	3	2.525	0.8418	-0.259725	2	1	0	0	0	0	Complemen	HYDROLASE	CFAB_HUMA	Factor B	NONE	NONE
CYP19A1	3eqm_v	3	2.512	0.8374	0.0468122	2	1	0	0	0	0	Cytochrom	NONE	P11511	Catalyzes	NONE	NONE
MAPK1	1pme_v	3	2.473	0.8242	-0.333706	3	0	0	0	0	0	Mitogen-a	TRANSFERA	MK01_HUMA	Involved	NONE	NONE
KIF11	2pg2_v	3	2.447	0.8156	-0.291779	3	0	0	0	0	0	Kinesin-l	MOTOR PRC	KIF11_HUM	Motor prc	NONE	Melanoma;
MAPK10	1pmv_v	3	2.446	0.8152	-0.944538	2	1	0	0	0	0	Mitogen-a	TRANSFERA	MK10_HUMA	Responds	A chromos	Central n
CDK2	2vta_v	3	2.435	0.8116	0.162992	1	1	1	0	0	0	Cell divi	NONE	P24941	Involved	NONE	NONE
MAPK8	1uki_v	3	2.334	0.7781	-0.738976	2	1	0	0	0	0	Mitogen-a	TRANSFERA	MK08_HUMA	Responds	NONE	Psoriasis
MAOB	2bk5_v	3	2.312	0.7707	-0.00573838	1	1	1	0	0	0	Amine oxi	OXIDOREDU	AOFB_HUMA	Catalyzes	NONE	Alzheimer
SULT2A1	1ov4_v	5	3.791	0.7582	1.85152	4	1	0	0	0	0	Bile salt	NONE	Q06520	Involved	NONE	NONE
PNP	1v3q_v	3	2.264	0.7546	-0.562938	1	2	0	0	0	0	Purine nu	TRANSFERA	PNPH_HUMA	NONE	Defects i	T-cell ly
AR	1xj7_v	3	2.263	0.7543	-0.257755	2	1	0	0	0	0	Androgen	HORMONE/C	ANDR_HUMA	Steroid h	Genetic v	Hypoactiv
CES1	1ya4_v	4	2.975	0.7439	0.866272	4	0	0	0	0	0	Liver car	HYDROLASE	EST1_HUMA	Involved	NONE	Arteriosc
PAH	4pah_v	4	2.967	0.7419	0.990883	1	0	2	1	0	0	Phenylala	MONOOXYGE	PH4H_HUMA	NONE	Defects i	Phenylket
MAPK14	1w84_v	4	2.966	0.7414	0.856479	3	0	1	0	0	0	Mitogen-a	NONE	Q16539	Involved	NONE	NONE
MMP13	830c_v	4	2.965	0.7412	0.764965	3	1	0	0	0	0	Collagena	MATRIX ME	MMP13_HUM	Degrades	Defects i	NONE
HSD17B1	1qyx_v	5	3.702	0.7405	1.73626	4	1	0	0	0	0	Estradiol	NONE	P14061	Lipid tra	NONE	Some evid
AMPM2	1yw9_v	4	2.962	0.7405	0.975637	3	0	0	0	1	0	Methionin	HYDROLASE	AMPM2_HUM	Removes t	NONE	NONE
CCNA2	1h1s_v	4	2.941	0.7353	1.02505	2	2	0	0	0	0	Cyclin-A2	TRANSFERA	CCNA2_HUM	Essential	NONE	NONE
TGFBR1	1vjy_v	4	2.917	0.7293	0.710506	3	1	0	0	0	0	TGF-beta	TRANSFERA	TGFR1_HUM	On ligand	Defects i	Skin burn
NR3C2	2aa5_v	5	3.643	0.7286	1.91889	4	1	0	0	0	0	Mineraloc	TRANSCRIP	MCR_HUMAN	Receptor	Defects i	Metabolic
SHBG	1lhw_v	4	2.909	0.7272	0.775848	2	2	0	0	0	0	Sex hormc	TRANSPORT	SHBG_HUMA	Functions	NONE	NONE
RORA	1n83_v	4	2.908	0.727	0.616297	4	0	0	0	0	0	Nuclear r	LIPID BIN	RORA_HUMA	Orphan nu	NONE	NONE
PPP5C	1s95_v	4	2.907	0.7266	0.752247	3	0	1	0	0	0	Serine/th	NONE	P53041	Involved	NONE	NONE
LSS	1w6j_v	5	3.628	0.7255	2.48351	2	2	1	0	0	0	Lanostero	ISOMERASE	ERG7_HUMA	Catalyzes	NONE	Hyperlipi
SRC	1o4i_v	4	2.899	0.7248	0.463792	1	2	0	0	1	0	Proto-onc	SIGNALING	SRC_HUMAN	NONE	NONE	Hypercalc
FABP4	1tow_v	4	2.892	0.7231	0.410519	2	1	0	0	1	0	Fatty aci	LIPID TRA	FABP4_HUM	Lipid tra	NONE	Non-insul
ADH1B	1u3v_v	4	2.891	0.7228	0.80849	2	0	1	1	0	0	Alcohol d	OXIDOREDU	ADH1B_HUM	NONE	NONE	NONE
LCN2	1l6m_v	4	2.886	0.7215	0.528964	1	1	1	0	1	0	Neutrophi	TRANSPORT	NGAL_HUMA	Transport	NONE	NONE
#N/A	2j7x_v	4	2.877	0.7194	0.77565	2	1	1	0	0	0	Estrogen	NONE	Q62986	Involved	NONE	NONE
DUSP6	1mkp_v	4	2.871	0.7178	0.692185	3	0	1	0	0	0	Dual spec	HYDROLASE	DUS6_HUMA	Inactivat	NONE	NONE
EGFR	1m17_v	4	2.866	0.7165	0.745371	2	2	0	0	0	0	Epidermal	TRANSFERA	EGFR_HUMA	Receptor	Defects i	Squamous
CHEK1	2brg_v	3	2.145	0.7149	-0.640584	2	1	0	0	0	0	Serine/th	TRANSFERA	CHK1_HUMA	Required	NONE	Cancer;Sc
REN	2g1y_v	4	2.849	0.7123	0.836759	2	1	1	0	0	0	Renin	HYDROLASE	RENI_HUMA	Renin is	Defects i	Glaucoma;
NOS3	1m9r_v	4	2.809	0.7022	0.531768	2	1	0	1	0	0	Nitric ox	OXIDOREDU	NOS3_HUMA	Produces	NONE	Coronary
CHIT1	1waw_v	4	2.792	0.6979	0.474624	1	2	1	0	0	0	Chitotrio	NONE	Q13231	Involved	NONE	NONE
AURKA	2w1f_v	4	2.79	0.6975	0.673058	2	1	1	0	0	0	Serine/th	NONE	O14965	Involved	NONE	NONE
TREM1	1q8m_v	3	2.08	0.6932	-0.698524	1	2	0	0	0	0	Triggerin	NONE	Q9NP99	Involved	NONE	For nutri
SEC14L2	1o6u_v	5	3.443	0.6885	1.23746	5	0	0	0	0	0	SEC14-lik	NONE	O76054	Involved	NONE	Vitamin E
ADAM17	1zxc_v	4	2.748	0.6869	0.167386	3	1	0	0	0	0	ADAM 17	HYDROLASE	ADA17_HUM	Cleaves t	NONE	Psoriasis
PPARD	3dy6_v	4	2.736	0.6841	0.26568	3	1	0	0	0	0	Peroxisom	NONE	Q03181	Involved	NONE	EPA can b
PTPN1	1c83_v	4	2.724	0.681	0.0768516	0	2	0	0	2	0	Tyrosine-	HYDROLASE	PTN1_HUMA	May play	NONE	NONE
MMP8	1jao_v	4	2.718	0.6794	0.520397	1	1	2	0	0	0	Neutrophi	COMPLEX (	MMP8_HUMA	Can degra	NONE	NONE
HSD11B1	3czr_v	5	3.388	0.6775	1.29299	4	1	0	0	0	0	Corticost	OXIDOREDU	DHI1_HUMA	Catalyzes	Defects i	Non-insul
ANXA5	1hak_v	4	2.703	0.6758	-0.308763	3	1	0	0	0	0	Annexin A	CALCIUM/F	ANXA5_HUM	This prot	NONE	NONE
PPARG	1fm6_v	4	2.7	0.675	0.0302989	3	1	0	0	0	0	Peroxisom	TRANSCRIP	PPARG_HUM	Receptor	Defects i	Asthma;Ce
ADH5	1m6w_v	5	3.374	0.6749	1.42976	3	0	1	0	1	0	Alcohol d	OXIDOREDU	ADHX_HUMA	Class-III	NONE	NONE
MIF	1gcz_v	4	2.689	0.6723	0.347889	2	2	0	0	0	0	Macrophag	IMMUNE SY	MIF_HUMAN	The expre	Genetic v	Insulin d
KDR	2p2h_v	4	2.65	0.6625	0.234968	2	1	1	0	0	0	Vascular	TRANSFERA	VGFR2_HUM	Receptor	NONE	Angiogene
TYMS	1juj_v	4	2.641	0.6602	-0.295069	2	1	0	0	1	0	Thymidyla	TRANSFERA	TYSY_HUMA	NONE	NONE	Small-cel
#N/A	2fb8_v	4	2.632	0.658	-0.00403994	3	1	0	0	0	0	B-Raf prc	TRANSFERA	BRAF1_HUM	Involved	Defects i	Melanoma;
PTPN11	2shp_v	5	3.29	0.6579	1.1287	4	0	0	1	0	0	Tyrosine-	TYROSINE	PTN11_HUM	Acts down	Defects i	NONE
#N/A	1ctr_v	5	3.289	0.6578	1.15637	5	0	0	0	0	0	Calmoduli	CALCIUM-E	CALM_HUMA	Calmoduli	NONE	NONE
RXRA	2acl_v	4	2.627	0.6568	0.108424	3	1	0	0	0	0	Retinoic	TRANSCRIP	RXRA_HUMA	Nuclear h	NONE	Acute pro
SORD	1pl6_v	5	3.277	0.6554	1.14436	2	3	0	0	0	0	Sorbitol	OXIDOREDU	DHSO_HUMA	NONE	NONE	Diabetic
HSP90AA1	2byi_v	4	2.591	0.6479	0.37281	1	1	2	0	0	0	Heat shoc	CHAPERONE	HS90A_HUM	Molecular	NONE	NONE
CTSK	1q6k_v	4	2.591	0.6476	0.225785	1	2	1	0	0	0	Cathepsin	HYDROLASE	CATK_HUMA	Closely i	Defects i	Multiple
PDPK1	2pe0_v	4	2.588	0.6471	-0.00280628	3	1	0	0	0	0	3-phospho	TRANSFERA	PDPK1_HUM	Phosphory	NONE	NONE
PDE4D	1xor_v	5	3.202	0.6404	1.36795	3	1	1	0	0	0	cAMP-spec	HYDROLASE	PDE4D_HUM	Regulates	Genetic v	NONE
IGF1R	3f5p_v	4	2.55	0.6376	-0.059969	3	1	0	0	0	0	Insulin-l	NONE	P08069	Involved	NONE	For treat
MTAP	1cg6_v	5	3.184	0.6368	0.40087	2	1	2	0	0	0	S-methyl-	NONE	Q13126	Nucleotid	NONE	For nutri
ESRRG	1s9p_v	6	3.814	0.6356	2.29365	4	2	0	0	0	0	Estrogen-	TRANSCRIP	ERR3_HUMA	Orphan re	NONE	NONE
TNNC1	1ih0_v	4	2.539	0.6346	-0.149965	3	0	1	0	0	0	Troponin	NONE	P63316	Involved	NONE	For short
IMPA1	1awb_v	6	3.78	0.6299	0.69288	0	3	2	0	1	0	Inositol	HYDROLASE	IMPA1_HUM	Responsib	The block	NONE
SULT2B1	1q22_v	7	4.4	0.6285	2.52112	6	1	0	0	0	0	Sulfotran	NONE	O00204	Involved	NONE	NONE
NQO1	1kbo_v	4	2.507	0.6267	-0.209214	3	0	1	0	0	0	NAD(P)H d	OXIDOREDU	NQO1_HUMA	The enzym	NONE	NONE
DHFR	1s3w_v	4	2.503	0.6257	0.114335	2	0	2	0	0	0	Dihydrofo	OXIDOREDU	DYR_HUMAN	NONE	NONE	NONE
F10	2bq6_v	4	2.462	0.6155	-0.104903	2	1	0	1	0	0	Coagulati	HYDROLASE	FA10_HUMA	Factor Xa	NONE	Thromboem
#N/A	1r7y_v	6	3.662	0.6103	2.73255	3	0	2	1	0	0	Histo-blc	NONE	P16442	Involved	NONE	NONE

CSNK2A1	3h30_v	4	2.437	0.6092	0.0584914	2	0	2	0	0	0	Casein ki	NONE	P68400	Involved	NONE	Being inv
HSPA8	3fzk_v	4	2.436	0.6091	-0.253067	2	2	0	0	0	0	Heat shoc	NONE	P11142	Molecular	NONE	NONE
TTPA	1oiz_v	5	3.044	0.6088	0.666902	4	1	0	0	0	0	Alpha-toc	NONE	P49638	Involved	NONE	Vitamin E
PYGL	1fa9_v	4	2.432	0.608	-0.287533	0	2	2	0	0	0	Glycogen	NONE	P06737	Carbohydr	NONE	For nutri
MMP3	1d7x_v	4	2.424	0.6061	-0.0557303	1	1	2	0	0	0	Stromelys	HYDROLASE	MMP3_HUMA	Can degra	NONE	NONE
GSK3B	3f7z_v	5	3.029	0.6058	0.913089	3	2	0	0	0	0	Glycogen	NONE	P49841	Involved	NONE	NONE
HDAC8	1w22_v	6	3.603	0.6005	1.6543	3	1	2	0	0	0	Histone c	HYDROLASE	HDAC8_HUM	Responsib	NONE	NONE
DPP4	2hha_v	5	2.992	0.5984	1.11623	2	2	0	1	0	0	Dipeptidy	HYDROLASE	DPP4_HUMA	Removes N	NONE	Rheumatoi
PNMT	1n7i_v	4	2.39	0.5976	-0.86091	3	0	0	1	0	0	Phenyleth	TRANSFERA	PNMT_HUMA	Converts	NONE	NONE
DHODH	1d3g_v	4	2.384	0.5961	-0.6868	3	0	1	0	0	0	Dihydroor	OXIDOREDU	PYRD_HUMA	NONE	NONE	NONE
LCK	2of4_v	4	2.384	0.5961	-0.234403	2	1	0	1	0	0	Proto-onc	TRANSFERA	LCK_HUMAN	Tyrosine	A chromos	Psoriasis
PDE3B	1so2_v	5	2.954	0.5908	0.797327	2	2	1	0	0	0	cGMP-inhi	HYDROLASE	PDE3B_HUM	May play	NONE	NONE
CFD	1dic_v	4	2.352	0.5881	-0.614332	2	2	0	0	0	0	Complemen	SERINE PR	CFAD_HUMA	Factor D	Defects i	Autoimmun
CTSG	1cgh_v	5	2.935	0.587	0.975307	1	2	0	2	0	0	Cathepsin	COMPLEX (	CATG_HUMA	Serine pr	NONE	Connectiv
PARP1	1wok_v	5	2.934	0.5867	0.494421	3	1	1	0	0	0	Poly [ADP	NONE	P09874	Involved	NONE	NONE
PGF	1fzv_v	5	2.931	0.5862	0.720473	3	0	2	0	0	0	Placenta	HORMONE/G	PLGF_HUMA	Growth fa	NONE	Retinopat
CPB1	1kwm_v	5	2.927	0.5855	-0.10423	0	3	0	0	2	0	Carboxype	NONE	P15086	Involved	NONE	NONE
AMY2A	3cpu_v	5	2.918	0.5837	0.907559	0	2	3	0	0	0	Pancreati	HYDROLASE	AMYP_HUMA	NONE	NONE	Bacillus
F2	1ktt_v	5	2.905	0.5811	0.571482	3	1	0	1	0	0	Prothromb	HYDROLASE	THRB_HUMA	Thrombin,	Defects i	Deep vein
ISG20	1wlj_v	5	2.899	0.5798	0.255112	0	4	1	0	0	0	Interfero	HYDROLASE	ISG20_HUM	Exonuclea	NONE	NONE
LTA4H	3cho_v	4	2.307	0.5767	-0.149397	2	1	0	1	0	0	Leukotrie	HYDROLASE	LKHA4_HUM	Hydrolyze	NONE	Inflammat
PDE5A	1rkp_v	4	2.296	0.5741	-0.424825	1	2	0	0	0	1	cGMP-spec	HYDROLASE	PDE5A_HUM	Plays a r	NONE	Anal fiss
ITK	1sm2_v	6	3.437	0.5729	1.3578	4	2	0	0	0	0	Tyrosine-	NONE	Q08881	Involved	NONE	NONE
ADK	2i6b_v	6	3.421	0.5702	1.04405	5	1	0	0	0	0	Adenosine	NONE	P55263	ATP depen	NONE	NONE
PLAU	1f92_v	5	2.847	0.5695	0.756844	1	2	1	1	0	0	Urokinase	HYDROLASE	UROK_HUMA	Specifica	NONE	Angiogene
MMP12	1utt_v	5	2.846	0.5693	0.373809	2	3	0	0	0	0	Macrophag	HYDROLASE	MMP12_HUM	May be in	NONE	Chronic c
HMGCR	2q6c_v	5	2.845	0.5691	0.516261	2	2	0	0	1	0	3-hydroxy	OXIDOREDU	HMDH_HUMA	This tran	NONE	Alzheimer
CYP2C9	1og5_v	6	3.414	0.5689	1.61603	3	3	0	0	0	0	Cytochrom	ELECTRON	CP2C9_HUM	Cytochrom	NONE	NONE
CBR1	1wma_v	5	2.834	0.5668	0.520288	2	2	1	0	0	0	Carbonyl	OXIDOREDU	CBR1_HUMA	Catalyzes	NONE	NONE
EPHX2	1vj5_v	5	2.818	0.5636	0.181816	3	1	1	0	0	0	Epoxide h	HYDROLASE	HYES_HUMA	Acts on e	NONE	NONE
ADH1C	1u3w_v	5	2.812	0.5625	0.25127	3	1	0	1	0	0	Alcohol d	NONE	P00326	Energy pr	NONE	Some evid
AHCY	1a7a_v	5	2.809	0.5617	0.123104	0	3	2	0	0	0	Adenosylh	HYDROLASE	SAHH_HUMA	Adenosylh	Defects i	Inflammat
ALDH2	1of7_v	5	2.806	0.5612	0.603611	2	1	2	0	0	0	Aldehyde	OXIDOREDU	ALDH2_HUM	NONE	NONE	NONE
SERPINA1	1iz2_v	6	3.359	0.5598	1.55942	3	1	2	0	0	0	Alpha-1-a	PROTEIN E	A1AT_HUMA	Inhibitor	The major	Psoriasis
EPHB4	2vww_v	4	2.238	0.5595	-0.727475	3	0	1	0	0	0	Ephrin ty	TRANSFERA	EPHB4_HUM	Receptor	NONE	Ocular di
FGFR1	1agw_v	4	2.219	0.5549	-0.81237	2	1	1	0	0	0	Basic fib	PROTEIN K	FGFR1_HUM	Receptor	Defects i	NONE
JAK3	1yvj_v	6	3.327	0.5545	1.19431	4	1	1	0	0	0	Tyrosine-	TRANSFERA	JAK3_HUMA	Tyrosine	Defects i	Psoriasis
CTNNA1	1h6g_v	5	2.771	0.5541	0.240449	3	2	0	0	0	0	Catenin a	CYTOSKELE	CTNA1_HUM	Associate	Abnormali	NONE
WAS	1t84_v	5	2.759	0.5518	-0.382247	5	0	0	0	0	0	Wiskott-A	SIGNALING	WASP_HUMA	Effector	Defects i	Wiskott-A
TRAPPC3	1sz7_v	6	3.298	0.5496	0.894174	5	1	0	0	0	0	Trafficki	NONE	O43617	May play	NONE	NONE
SOD2	1xil_v	5	2.748	0.5496	0.276764	2	2	1	0	0	0	Superoxid	NONE	P04179	Destroys	NONE	NONE
GLRX	1b4q_v	5	2.733	0.5466	0.21955	1	1	0	1	2	0	Glutaredo	OXIDOREDU	GLRX1_HUM	Has a glu	NONE	NONE
FABP7	1fe3_v	7	3.826	0.5465	1.33678	6	0	0	0	1	0	Fatty aci	LIPID BIN	FABP7_HUM	B-FABP co	NONE	NONE
REG1A	1qdd_v	5	2.725	0.545	0.392411	0	4	1	0	0	0	Lithostat	NONE	P05451	Involved	NONE	NONE
NR1H2	1upw_v	6	3.266	0.5444	0.979271	5	0	1	0	0	0	Oxysterol	RECEPTOR	NR1H2_HUM	Orphan re	NONE	Lipid met
FGFR2	1oec_v	5	2.703	0.5406	-0.243324	3	1	0	1	0	0	Fibroblas	NONE	P21802	Involved	NONE	For treat
NR1H3	1uhl_v	6	3.243	0.5405	1.01947	5	1	0	0	0	0	Oxysterol	DNA BINDI	NR1H3_HUM	Orphan re	NONE	NONE
PPARA	1i7g_v	6	3.233	0.5388	1.10437	4	2	0	0	0	0	Peroxisom	TRANSCRIP	PPARA_HUM	Receptor	NONE	Syndrome
MTHFD1	1dia_v	6	3.232	0.5387	1.69957	2	2	2	0	0	0	C-1-tetra	NONE	P11586	Nucleotid	NONE	For nutri
NR1H4	1osh_v	5	2.681	0.5362	-0.314068	5	0	0	0	0	0	Bile acid	TRANSCRIP	NR1H4_HUM	Receptor	NONE	Liver fib
#N/A	1ydt_v	5	2.671	0.5343	-0.254979	4	0	0	1	0	0	cAMP-depe	NONE	P00517	Involved	NONE	NONE
AKR1C1	1mrq_v	5	2.656	0.5311	-0.19207	4	1	0	0	0	0	Aldo-keto	NONE	Q04828	Involved	NONE	Some evid
#N/A	2vd1_v	6	3.181	0.5301	0.566682	5	1	0	0	0	0	Glutathio	ISOMERASE	PTGD2_HUM	Catalyzes	NONE	NONE
GLO1	1qip_v	7	3.71	0.53	1.05243	3	1	0	1	2	0	Lactoylgl	LYASE	LGUL_HUMA	Catalyzes	NONE	Cancer
SULT1E1	1g3m_v	7	3.708	0.5297	1.4124	6	1	0	0	0	0	Estrogen	TRANSFERA	ST1E1_HUM	May contr	NONE	NONE
THRA	1nav_v	7	3.68	0.5257	1.49423	4	2	1	0	0	0	Thyroid h	NONE	P10827	Involved	NONE	Used as r
SYK	1xbc_v	5	2.615	0.523	-0.274543	3	1	1	0	0	0	Tyrosine-	TRANSFERA	KSYK_HUMA	Positive	NONE	Systemic
#N/A	1gzr_v	6	3.129	0.5215	1.04478	3	2	0	1	0	0	Insulin-l	NONE	P01343	NONE	NONE	NONE
ADAM17	3edz_v	5	2.597	0.5194	0.413479	2	2	0	0	0	1	Disintegr	NONE	P78536	Cleaves t	NONE	NONE
RBP4	1rbp_v	8	4.134	0.5168	1.03618	8	0	0	0	0	0	Retinol-b	RETINOL T	RET4_HUMA	Delivers	Defects i	NONE
GSTT2B	1ljr_v	7	3.601	0.5144	2.02054	1	3	1	1	1	0	Glutathio	TRANSFERA	GSTT2_HUM	Conjugati	NONE	NONE
GSTA1	1gse_v	6	3.085	0.5142	0.400347	5	1	0	0	0	0	Glutathio	NONE	P08263	Involved	NONE	For nutri
XIAP	2opy_v	5	2.561	0.5123	0.124794	1	2	0	1	0	1	Baculovir	APOPTOSIS	XIAP_HUMA	Apoptotic	Defects i	Cancer;Sk
FABP6	1o1v_v	7	3.58	0.5114	1.04953	5	1	1	0	0	0	Gastrotro	LIPID BIN	FABP6_HUM	Ileal pro	NONE	NONE
FABP3	2hmb_v	7	3.559	0.5084	0.878363	5	1	0	0	1	0	Fatty aci	LIPID-BIN	FABPH_HUM	FABP are	NONE	NONE
PLA2G2A	1dcy_v	5	2.528	0.5056	-0.954748	3	1	0	0	1	0	Phospholi	NONE	P14555	Involved	NONE	NONE
CSNK1G2	2c47_v	4	2.02	0.5051	-1.20191	1	1	2	0	0	0	Casein ki	TRANSFERA	KC1G2_HUM	Casein ki	NONE	NONE
YARS	1q11_v	5	2.525	0.5049	-0.40966	2	1	1	1	0	0	Tyrosyl-t	NONE	P54577	Translati	NONE	Tyrosine
CDK6	1xo2_v	5	2.523	0.5047	-0.18693	2	3	0	0	0	0	Cell divi	CELL CYCL	CDK6_HUMA	Probably	NONE	NONE
RNASE4	2rnf_v	5	2.507	0.5013	-0.462335	0	3	1	0	1	0	Ribonucle	NONE	P34096	Involved	NONE	NONE
FHIT	2fhi_v	5	2.502	0.5004	0.0817782	0	3	1	0	0	1	Bis(5-ade	NONE	P49789	Nucleotid	NONE	NONE
MET	1r0p_v	6	2.975	0.4958	0.121518	4	2	0	0	0	0	Hepatocyt	TRANSFERA	MET_HUMAN	Receptor	Activatio	Alopecia,
PDK2	2bu5_v	6	2.967	0.4945	0.0375573	5	1	0	0	0	0	[Pyruvate	TRANSFERA	PDK2_HUMA	Inhibits	NONE	NONE
NR1I3	1xvp_v	6	2.966	0.4943	0.241254	6	0	0	0	0	0	Nuclear r	DNA BINDI	NR1I3_HUM	Binds and	NONE	NONE
PCK1	1nhx_v	6	2.956	0.4926	0.0905391	1	3	0	0	2	0	Phosphoen	NONE	P35558	Energy pr	NONE	NONE
F7	2flr_v	5	2.421	0.4842	-0.559078	3	0	2	0	0	0	Coagulati	HYDROLASE	FA7_HUMAN	Initiates	Defects i	Deep vein
BLVRB	1he2_v	6	2.901	0.4835	0.246847	4	1	0	0	1	0	Flavin re	NONE	P30043	Cell wall	NONE	For the t
RHOA	1a2b_v	6	2.894	0.4823	0.915877	0	4	2	0	0	0	Transform	NONE	P61586	Involved	NONE	For the t
DPEP1	1itu_v	8	3.841	0.4802	1.27229	4	2	1	0	1	0	Dipeptida	HYDROLASE	DPEP1_HUM	Hydrolyze	NONE	NONE
#N/A	2f57_v	5	2.393	0.4785	-0.523768	3	1	1	0	0	0	Serine/th	TRANSFERA	PAK7_HUMA	The activ	NONE	NONE
MDM2	1rv1_v	5	2.388	0.4777	-1.11008	4	0	1	0	0	0	E3 ubiqui	LIGASE	MDM2_HUMA	Inhibits	Seems to	Leukemia;
AMY1B	1q4n_v	6	2.859	0.4765	0.576842	2	1	2	1	0	0	Alpha-amy	NONE	P04745	Carbohydr	NONE	NONE
FABP5	1b56_v	5	2.363	0.4727	-0.99898	3	1	0	0	1	0	Fatty aci	LIPID-BIN	FABP5_HUM	High spec	NONE	NONE
NR3C1	1nhz_v	8	3.762	0.4702	1.03739	7	1	0	0	0	0	Glucocort	HORMONE F	GCR_HUMAN	Receptor	Defects i	Alzheimer
VDR	1s19_v	8	3.758	0.4697	2.13507	5	2	1	0	0	0	Vitamin D	GENE REGU	VDR_HUMAN	Nuclear h	Defects i	Pagets bo
SELP	1g1r_v	6	2.817	0.4694	0.796425	0	3	3	0	0	0	P-selecti	IMMUNE SY	LYAM3_HUM	Ca(2+)-de	Defects i	Sickle ce
AMD1	1i7b_v	8	3.748	0.4685	3.05423	2	3	3	0	0	0	S-adenosy	LYASE	DCAM_HUMA	NONE	NONE	Colorecta
TPI1	1hti_v	6	2.801	0.4668	-0.131938	1	3	0	0	2	0	Triosepho	ISOMERASE	TPIS_HUMA	NONE	Defects i	NONE
BST1	1isg_v	6	2.8	0.4667	0.0154672	0	5	1	0	0	0	ADP-ribos	HYDROLASE	BST1_HUMA	Synthesiz	Rheumatoi	Hematolog
FKBP1A	1fki_v	8	3.731	0.4664	1.35914	5	3	0	0	0	0	Peptidyl-	CIS-TRANS	FKB1A_HUM	May play	NONE	NONE
HNF4G	1lv2_v	7	3.259	0.4656	0.513709	5	1	0	0	1	0	Hepatocyt	NONE	Q14541	Involved	NONE	NONE
PIK3CG	1e8z_v	5	2.327	0.4654	-1.28562	3	1	0	1	0	0	Phosphati	PHOSPHOIN	PK3CG_HUM	3-phospho	NONE	NONE
CRABP2	3cbs_v	9	4.165	0.4628	1.25718	6	2	0	0	1	0	Cellular	NONE	P29373	Involved	NONE	For topic
CDA	1mq0_v	5	2.313	0.4626	-1.15029	1	3	1	0	0	0	Cytidine	HYDROLASE	CDD_HUMAN	This enzy	NONE	NONE
FECH	1hrk_v	8	3.688	0.4609	1.3933	5	1	2	0	0	0	Ferrochel	NONE	P22830	Coenzyme	NONE	NONE
RNASE3	1dyt_v	6	2.756	0.4593	-0.150687	0	5	1	0	0	0	Eosinophi	NONE	P12724	Involved	NONE	Used as a
ABL1	2hzi_v	8	3.663	0.4579	1.6674	6	1	1	0	0	0	Proto-onc	NONE	P00519	Involved	NONE	For nutri
JAK2	2b7a_v	7	3.196	0.4566	0.57146	3	2	2	0	0	0	Tyrosine-	TRANSFERA	JAK2_HUMA	Plays a r	Chromosom	Psoriasis
NOS2	2nsi_v	6	2.738	0.4564	0.359769	1	1	4	0	0	0	Nitric ox	OXIDOREDU	NOS2_HUMA	Produces	NONE	Asthma;Mu
GM2A	1tjj_v	6	2.736	0.456	0.0353107	4	1	0	1	0	0	Gangliosi	NONE	P17900	Involved	NONE	NONE
ELANE	1h1b_v	6	2.709	0.4514	-0.188153	3	3	0	0	0	0	Leukocyte	HYDROLASE	ELNE_HUMA	Modifies	Defects i	Blood clo

APRT	1ore_v	7	3.158	0.4512	0.746162	0	4	3	0	0	0	Adenine p	NONE	P07741	Nucleotid	NONE	For nutri
FNTA	1sa4_v	7	3.149	0.4498	0.603779	5	0	0	2	0	0	Protein f	TRANSFERA	FNTA_HUMA	Catalyzes	NONE	NONE
TNK2	1u4d_v	5	2.24	0.448	-0.954582	0	2	3	0	0	0	Activated	TRANSFERA	ACK1_HUMA	Downstrea	NONE	Cancer
CYP2C8	1pq2_v	7	3.135	0.4478	0.435702	5	1	0	0	1	0	Cytochrom	OXIDOREDU	CP2C8_HUM	Cytochrom	NONE	NONE
DCK	1p62_v	7	3.126	0.4466	0.858597	3	3	1	0	0	0	Deoxycyti	TRANSFERA	DCK_HUMAN	Required	NONE	NONE
PRKCQ	1xjd_v	7	3.12	0.4458	0.427984	4	1	1	1	0	0	Protein k	TRANSFERA	KPCT_HUMA	This is a	NONE	Inflammat
NR1I2	2o9i_v	7	3.109	0.4442	0.267555	5	2	0	0	0	0	Nuclear r	TRANSCRIP	NR1I2_HUM	Orphan re	NONE	NONE
BIRC7	3f7h_v	6	2.654	0.4424	-0.030016	2	2	1	1	0	0	Baculovir	NONE	Q96CA5	Apoptotic	NONE	NONE
AMY1B	1mfv_v	6	2.652	0.442	0.0341128	0	2	4	0	0	0	Salivary	NONE	P04745	Carbohydr	NONE	NONE
RARB	1xap_v	9	3.949	0.4387	1.35272	7	1	0	0	1	0	Retinoic	TRANSCRIP	RARB_HUMA	This is a	NONE	Psoriasis
RARG	1fcx_v	9	3.927	0.4364	1.97412	6	1	1	0	1	0	Retinoic	GENE REGU	RARG_HUMA	This is a	NONE	Emphysema
RARA	1dkf_v	8	3.488	0.436	1.01032	5	3	0	0	0	0	Retinoic	HORMONE/C	RARA_HUMA	This is a	Chromosom	Squamous
ZAP70	1u59_v	7	3.011	0.4302	0.251011	4	1	2	0	0	0	Tyrosine-	TRANSFERA	ZAP70_HUM	Plays a r	Defects i	Transplan
MMP2	1hov_v	7	2.985	0.4264	0.451343	4	1	2	0	0	0	72 kDa ty	HYDROLASE	MMP2_HUMA	In additi	-Leu bond	Defects i
FDPS	1yq7_v	8	3.408	0.426	0.430207	1	4	1	0	2	0	Farnesyl	TRANSFERA	FPPS_HUMA	Key enzym	NONE	NONE
IL2	1m48_v	7	2.966	0.4237	0.290016	4	1	2	0	0	0	Interleuk	CYTOKINE	IL2_HUMAN	Produced	A chromos	Cancer
MAP2K1	1s9j_v	9	3.811	0.4234	0.82871	7	2	0	0	0	0	Dual spec	TRANSFERA	MP2K1_HUM	Catalyzes	Defects i	Colon tum
SETD7	1mt6_v	7	2.961	0.423	0.571635	2	2	2	0	1	0	Histone-l	TRANSFERA	SETD7_HUM	Histone m	NONE	NONE
HCK	1qcf_v	7	2.945	0.4207	-0.0957678	3	3	1	0	0	0	Tyrosine-	TYROSINE	HCK_HUMAN	May serve	NONE	NONE
S100A9	1irj_v	7	2.939	0.4198	0.252421	4	0	3	0	0	0	Protein S	METAL BIN	S10A9_HUM	Expressed	NONE	NONE
TEK	2oo8_v	8	3.35	0.4188	0.714277	5	1	1	1	0	0	Angiopoie	TRANSFERA	TIE2_HUMA	This prot	Defects i	Retinopat
ACPP	1nd5_v	6	2.509	0.4181	-1.06311	2	2	0	1	1	0	Prostatic	HYDROLASE	PPAP_HUMA	NONE	NONE	Prostate
HSP90AB1	1uym_v	8	3.341	0.4176	1.40731	5	1	1	0	0	1	Heat shoc	NONE	P08238	Posttrans	NONE	NONE
KAT2B	1cm0_v	9	3.723	0.4137	2.8146	1	6	2	0	0	0	Histone a	NONE	Q92831	Involved	NONE	NONE
TK1	1w4r_v	6	2.482	0.4136	-0.113883	0	2	2	0	2	0	Thymidine	NONE	P04183	Nucleotid	NONE	NONE
ERBB4	3bbt_v	6	2.476	0.4126	-0.925959	5	1	0	0	0	0	Receptor	TRANSFERA	ERBB4_HUM	Specifica	NONE	NONE
CTSF	1m6d_v	7	2.88	0.4115	0.384493	3	2	2	0	0	0	Cathepsin	NONE	Q9UBX1	Involved	NONE	NONE
SDS	1p5j_v	7	2.877	0.411	-0.188348	2	4	0	0	1	0	L-serine	NONE	P20132	Amino aci	NONE	For nutri
PSAP	1n69_v	7	2.876	0.4108	-0.0666955	6	0	0	0	1	0	Proactiva	NONE	P07602	Involved	NONE	NONE
PAPSS1	1x6v_v	7	2.874	0.4106	-0.194749	0	5	1	0	1	0	Bifunctio	NONE	O43252	Involved	NONE	NONE
#N/A	1svh_v	7	2.871	0.4101	0.322527	4	1	1	1	0	0	cAMP-depe	NONE	P00517	Involved	NONE	NONE
NQO2	1sg0_v	5	2.045	0.409	-1.39821	2	2	0	0	0	1	Ribosyldi	OXIDOREDU	NQO2_HUMA	The enzym	NONE	NONE
CTSS	1nqc_v	7	2.841	0.4059	0.119659	3	2	2	0	0	0	Cathepsin	HYDROLASE	CATS_HUMA	Thiol pro	NONE	Psoriasis
MMP16	1rm8_v	8	3.236	0.4045	0.887687	1	2	4	0	0	1	Matrix me	HYDROLASE	MMP16_HUM	Endopepti	NONE	NONE
MAN1B1	1x9d_v	7	2.829	0.4041	0.183273	0	5	2	0	0	0	Endoplasm	NONE	Q9UKM7	Involved	NONE	NONE
NT5M	1q91_v	9	3.634	0.4038	1.09764	1	6	1	0	1	0	5(3)-deox	NONE	Q9NPB1	Involved	NONE	NONE
ACADM	1egc_v	7	2.823	0.4033	0.479859	3	2	2	0	0	0	Medium-ch	ELECTRON	ACADM_HUM	This enzy	Defects i	NONE
TGM3	1l9n_v	8	3.199	0.3998	0.0664704	3	4	1	0	0	0	Protein-g	TRANSFERA	TGM3_HUMA	Catalyzes	NONE	NONE
DTYMK	1nmx_v	7	2.785	0.3979	-0.28059	1	4	1	0	1	0	Thymidyla	TRANSFERA	KTHY_HUMA	Catalyzes	NONE	NONE
GSTM1	1xwk_v	9	3.58	0.3978	1.96215	2	2	3	1	1	0	Glutathio	TRANSFERA	GSTM1_HUM	Conjugati	NONE	NONE
CSK	3d7t_v	6	2.387	0.3978	-0.752325	3	1	1	1	0	0	Tyrosine-	TRANSFERA	CSK_HUMAN	Specifica	NONE	NONE
AGXT	1h0c_v	7	2.77	0.3958	-0.350289	2	2	1	0	1	1	Serine--p	TRANSFERA	SPYA_HUMA	NONE	Defects i	Hyperoxal
SHMT1	1bj4_v	6	2.368	0.3946	-0.676854	2	4	0	0	0	0	Serine hy	TRANSFERA	GLYC_HUMA	Interconv	NONE	NONE
IMPDH1	1jcn_v	7	2.753	0.3933	-0.363561	0	5	1	0	1	0	Inosine-5	OXIDOREDU	IMDH1_HUM	Rate limi	Defects i	NONE
HNMT	1jqe_v	8	3.136	0.392	0.00536757	7	0	0	1	0	0	Histamine	NONE	P50135	Inactivat	NONE	For treat
PPCDC	1qzu_v	7	2.73	0.39	0.134535	1	3	2	0	1	0	Phosphopa	NONE	Q96CD2	Involved	NONE	NONE
HAGH	1qh5_v	7	2.692	0.3846	-0.331195	0	4	1	0	2	0	Hydroxyac	NONE	Q16775	Involved	NONE	For nutri
RXRB	1h9u_v	10	3.781	0.3781	0.716892	8	1	0	0	1	0	Retinoic	NUCLEAR F	RXRB_HUMA	Nuclear h	NONE	NONE
LGALS2	1hlc_v	6	2.267	0.3778	-1.13758	0	3	3	0	0	0	Galectin-	LECTIN	LEG2_HUMA	This prot	NONE	NONE
CMA1	1t31_v	8	3.021	0.3776	0.267583	3	4	0	0	1	0	Chymase	HYDROLASE	CMA1_HUMA	Major sec	NONE	Asthma;At
MME	1r1i_v	8	2.997	0.3746	0.0692056	3	2	1	1	1	0	Neprilysi	HYDROLASE	NEP_HUMAN	Thermolys	Important	Hypertens
CCBL1	1w7n_v	6	2.242	0.3736	-1.64553	1	2	1	1	1	0	Kynurenin	TRANSFERA	KAT1_HUMA	Catalyzes	NONE	NONE
CD209	1sl4_v	8	2.956	0.3695	0.898312	0	5	3	0	0	0	CD209 ant	SUGAR BIN	CD209_HUM	Pathogen-	NONE	NONE
AKT2	2uw9_v	6	2.214	0.369	-0.979077	3	1	1	1	0	0	RAC-beta	TRANSFERA	AKT2_HUMA	General p	Alteratio	NONE
PPP1CC	1jk7_v	9	3.314	0.3682	0.736053	6	1	1	0	1	0	Serine/th	NONE	P36873	Signal tr	NONE	NONE
MMP1	1hfc_v	8	2.943	0.3678	0.176404	1	3	4	0	0	0	Interstit	NONE	P03956	Cleaves c	NONE	NONE
F11	1zpb_v	8	2.934	0.3668	0.346602	3	3	1	1	0	0	Coagulati	HYDROLASE	FA11_HUMA	Factor XI	Defects i	Thrombosi
BCAT2	1ekv_v	8	2.932	0.3665	-0.0374593	2	3	2	0	1	0	Branched-	NONE	O15382	Amino aci	NONE	For nutri
LGALS3	1kjl_v	7	2.558	0.3654	-0.403225	0	3	4	0	0	0	Galectin-	SUGAR BIN	LEG3_HUMA	Galactose	NONE	Colorecta
MMP9	1gkc_v	8	2.9	0.3626	-0.134847	2	4	2	0	0	0	Matrix me	HYDROLASE	MMP9_HUMA	May play	-Leu bond	Defects i
GNPDA1	1ne7_v	7	2.53	0.3614	-0.719854	0	4	3	0	0	0	Glucosami	NONE	P46926	Carbohydr	NONE	NONE
UCK2	1uj2_v	8	2.877	0.3597	-0.234299	1	3	3	0	1	0	Uridine-c	NONE	Q9BZX2	Nucleotid	NONE	NONE
CLEC4M	1k9j_v	8	2.857	0.3572	0.779822	0	5	3	0	0	0	C-type le	NONE	Q9H2X3	Probable	NONE	NONE
GP1BA	1p9a_v	8	2.806	0.3507	0.327505	0	5	3	0	0	0	Platelet	NONE	P07359	Involved	NONE	NONE
GSTO1	1eem_v	8	2.794	0.3492	-0.131799	0	4	2	1	1	0	Glutathio	TRANSFERA	GSTO1_HUM	Exhibits	NONE	NONE
STAT1	1yvl_v	8	2.79	0.3488	-0.108378	1	4	2	1	0	0	Signal tr	SIGNALING	STAT1_HUM	Signal tr	Defects i	Respirato
CDC42	1a4r_v	8	2.786	0.3483	-0.461036	0	5	2	0	1	0	Cell divi	HYDROLASE	CDC42_HUM	Plasma me	NONE	NONE
CTSB	1gmy_v	9	3.134	0.3482	0.396921	4	2	3	0	0	0	Cathepsin	COMPLEX (	CATB_HUMA	Thiol pro	NONE	Myocardia
PCTP	1ln3_v	11	3.806	0.346	1.13577	9	0	0	1	1	0	Phosphati	NONE	Q9UKL6	Involved	NONE	NONE
GPI	1nuh_v	8	2.753	0.3442	-0.645543	1	5	1	0	1	0	Glucose-6	ISOMERASE	G6PI_HUMA	Neurotrop	Defects i	NONE
#N/A	2qcf_v	6	2.065	0.3442	-1.58237	0	3	2	0	1	0	Uridine 5	LYASE	PYR5_HUMA	NONE	Defects i	Squamous
OTC	1oth_v	8	2.741	0.3426	-0.527379	1	4	1	1	1	0	Ornithine	NONE	P00480	Amino aci	NONE	Used for
TPSB2	2fs8_v	8	2.737	0.3421	0.0670373	2	2	2	1	1	0	Tryptase	HYDROLASE	TRYB2_HUM	Tryptase	NONE	NONE
ARF4	1z6x_v	8	2.735	0.3419	-0.438133	0	4	2	0	2	0	ADP-ribos	NONE	P18085	Involved	NONE	NONE
GRB2	1x0n_v	9	3.042	0.338	0.0405721	5	1	2	0	1	0	Growth fa	PEPTIDE B	GRB2_HUMA	Adapter p	NONE	Leukemia;
LYZ	1hnl_v	8	2.7	0.3375	-0.624927	1	3	2	1	1	0	Lysozyme	NONE	P61626	Lysozymes	NONE	NONE
CDK7	1ua2_v	8	2.693	0.3367	-0.520029	1	3	1	0	3	0	Cell divi	NONE	P50613	Involved	NONE	NONE
#N/A	1y0x_v	6	2.004	0.334	-1.68149	4	0	1	0	1	0	Thyroid h	NONE	P37243	Involved	NONE	Used as r
#N/A	3d2f_v	7	2.337	0.3339	-0.849303	0	5	2	0	0	0	Heat shoc	NONE	P32589	Has a cal	NONE	NONE
HINT1	1kpe_v	8	2.658	0.3323	-0.498287	0	3	5	0	0	0	Histidine	PROTEIN K	HINT1_HUM	Hydrolyze	NONE	NONE
TPH1	1mlw_v	7	2.301	0.3288	-1.46998	1	4	2	0	0	0	Tryptopha	OXIDOREDU	TPH1_HUMA	NONE	NONE	NONE
GSTM2	1xw5_v	9	2.958	0.3287	0.0717974	1	5	1	1	1	0	Glutathio	TRANSFERA	GSTM2_HUM	Conjugati	NONE	NONE
GSTA3	1tdi_v	9	2.941	0.3267	-0.189912	1	5	1	1	1	0	Glutathio	NONE	Q16772	Involved	NONE	For nutri
TGFB2	1tfg_v	9	2.914	0.3238	-0.0900954	4	2	2	1	0	0	Transform	GROWTH FA	TGFB2_HUM	TGF-beta	A chromos	NONE
CLK1	1z57_v	7	2.261	0.3229	-1.73491	0	5	1	1	0	0	Dual spec	TRANSFERA	CLK1_HUMA	Phosphory	NONE	Toxicity;
PIK3R1	1pic_v	9	2.902	0.3225	0.124348	5	1	1	0	2	0	Phosphati	COMPLEX (	P85A_HUMA	Binds to	Defects i	NONE
GCDH	1sir_v	8	2.576	0.322	-0.104138	1	4	1	1	1	0	Glutaryl-	NONE	Q92947	Lipid tra	NONE	NONE
ITPKA	1w2c_v	9	2.871	0.319	0.0846234	0	3	3	0	3	0	Inositol-	NONE	P23677	Involved	NONE	NONE
#N/A	1yzg_v	11	3.494	0.3177	0.723513	0	7	3	0	1	0	ADP-ribos	NONE	Q9D4P0	Involved	NONE	NONE
RNASE2	1hi4_v	9	2.849	0.3165	-0.145453	0	5	1	0	2	1	Non-secre	NONE	P10153	Involved	NONE	NONE
FKBP1B	1c9h_v	9	2.814	0.3126	0.207015	3	3	3	0	0	0	Peptidyl-	IMMUNE SY	FKB1B_HUM	Associate	NONE	NONE
EPHA2	1mqb_v	7	2.181	0.3116	-1.90465	0	3	3	0	1	0	Ephrin ty	TRANSFERA	EPHA2_HUM	Receptor	NONE	Cytomegal
SELE	1g1t_v	9	2.765	0.3072	-0.481055	0	7	1	0	1	0	E-selecti	IMMUNE SY	LYAM2_HUM	Cell-surf	NONE	Asthma;Lu
GART	1njs_v	11	3.366	0.306	0.471164	3	2	5	0	1	0	Trifuncti	TRANSFERA	PUR2_HUMA	NONE	NONE	Head and
KIT	1t46_v	9	2.733	0.3037	-0.483213	5	1	1	2	0	0	Mast/stem	TRANSFERA	KIT_HUMAN	This is t	Defects i	Pancreas
TRDMT1	1g55_v	10	3.024	0.3024	0.159893	1	6	2	1	0	0	tRNA (cyt	NONE	O14717	Specifica	NONE	NONE
BCL2L1	2yxj_v	9	2.698	0.2998	-0.329711	6	1	1	1	0	0	Bcl-2-lik	NONE	Q07817	Potent in	NONE	NONE
ARL5A	1z6y_v	9	2.684	0.2982	-0.71555	0	5	3	0	1	0	ADP-ribos	NONE	Q9Y689	Involved	NONE	NONE
ARG1	2aeb_v	7	2.082	0.2974	-1.32293	2	2	1	1	1	0	Arginase-	HYDROLASE	ARGI1_HUM	NONE	Defects i	NONE
NMNAT1	1gzu_v	7	2.076	0.2966	-2.15039	1	4	1	0	1	0	Nicotinam	NONE	Q9HAN9	Coenzyme	NONE	NONE
CASP1	1ice_v	10	2.963	0.2963	-0.227993	4	3	1	1	1	0	Caspase-1	CYTOKINE	CASP1_HUM	Thiol pro	NONE	Psoriasis

ease;Musculoskeletal disease;Periodontal disease;Bone injury;Osteoporosis

Fibroids;Menstruation disorder;Infertility;Acne;Breast tumor;Female contraception;Neoplasm;Planned abortion;Dysmenorrhea;Endometriosis;Hormone deficiency;Unidentif

disorder;Cancer;Breast tumor

city, drug-induced;Toxicity;Alzheimers disease
tumor
Cancer;Liver disease;Cognitive disorder;Neurodegenerative disease;Cerebrovascular ischemia
NONE
tional supplementation, also for treating dietary shortage or imbalance

Brain tumor;Inflammation;Myocardial infarction;Fibrosis;Brain ischemia;Psoriasis;Rheumatoid arthritis;Retinitis pigmentosa;Atherosclerosis;Genitourinary disease;B
y;Diabetic neuropathy;Diabetic foot ulcer;Diabetic complication;Diabetic nephropathy;Retinopathy;Ocular disease;Central nervous system disease;Diabetic retinopath
ment of transplant rejection, rheumatoid arthritis, severe psoriasis

bstructive pulmonary disease;Asthma

tional supplementation, also for treating dietary shortage or imbalance

Hodgkins disease;Acute leukemia;Breast tumor;Head and neck tumor;Hepatocellular carcinoma;Ovary tumor;Prostate tumor;Solid tumor;Non-Hodgkin lymphoma;Cancer;Non-s
ervous system disease;Inflammation;Pulmonary fibrosis;Ischemia;Cerebrovascular ischemia;Neurodegenerative disease

;Asthma;Cancer;Neurodegenerative disease;Inflammation;Liver disease;Acute myelogenous leukemia;Diabetes mellitus;Pulmonary fibrosis;Ischemia
s disease;Cocaine addiction;Major depressive disorder;Epilepsy;Neurodegenerative disease;Attention deficit hyperactivity disorder;Head injury;Parkinsons disease;D

mphoma;Autoimmune disease;HIV infection;Dermatitis;Chronic lymphocytic leukemia;Acquired Immune Deficiency Syndrome;Inflammation;Multiple sclerosis;Solid tumor;Ps
e sexual desire disorder;Hepatitis B virus infection;Cardiac failure;Andrology;Hirsutism;Female sexual dysfunction;Psoriasis;Reperfusion injury;Plasmodium infecti
lerosis;Cerebrovascular disease;Cancer;Melanoma;Multiple myeloma;Hypercholesterolemia;Atherosclerosis;Breast tumor;Hyperlipidemia;Solid tumor;Cardiovascular disea
onuria;Sickle cell anemia;Vascular disease;Genetic disorder;Diabetic nephropathy;Hypertension;Ataxia;Peripheral arterial occlusive disease;Coronary artery disease

lence suggests that NADH might be useful in treating Parkinsons disease, chronic fatigue syndrome, Alzheimers disease and cardiovascular disease.

s;Glomerulonephritis;Wound healing;Renal disease;Scleroderma;Glaucoma;Diabetic complication;Scar tissue;Inflammation;Urinary tract disease;Pulmonary fibrosis;Diab
disorder;Hypertension;Cardiovascular disease;Urinary tract disease

demia;Hypercholesterolemia;Atherosclerosis
emia;Cancer;Osteoporosis;Cerebrovascular ischemia;Bone metastases;Metastasis;Solid tumor
in dependent diabetes

cell carcinoma;Arteriosclerosis;Melanoma;Central nervous system tumor;Diabetes mellitus;Esophagus tumor;Ischemic heart disease;Brain tumor;Nasopharyngeal carcinoma
lid tumor
Renal disease;Cardiac failure;Atherosclerosis;HIV infection;Hypertension;Cardiovascular disease;Ocular hypertension;Renal failure
artery disease;Hypertension;Congestive heart failure;Angina;Myocardial infarction;Peripheral vascular disease;Atherosclerosis;Ischemic heart disease

tional supplementation, also for treating dietary shortage or imbalance
, known for its antioxidant activities, is protective against cardiovascular disease and some forms of cancer and has also demonstrated immune-enhancing effects.
;Inflammation;Rheumatoid arthritis;Cardiac failure;Multiple sclerosis;Metastasis;Immune disorder
e used for lowering elevated triglycerides in those who are hyperglyceridemic. In addition, EPA may play a therapeutic role in patients with cystic fibrosis by re

in dependent diabetes;Rheumatoid arthritis;Diabetes mellitus;Obesity;Atherosclerosis;Metabolic disorder;Periodontitis

erebrovascular ischemia;Conjunctivitis;Inflammation;Multiple sclerosis;Fibrosis;Unidentified indication;Psoriasis;Heart arrhythmia;Rheumatoid arthritis;Atheroscler

lependent diabetes;Glomerulonephritis;Cancer;Rheumatoid arthritis;Sepsis;Autoimmune disease;Atherosclerosis;Infection;Inflammation;Myocardial infarction;Immune dis
sis disorder;Melanoma;Cancer;Non-small-cell lung cancer;Renal cell carcinoma;Colorectal tumor;Ocular disease;Age related macular degeneration;Solid tumor
l lung cancer;Solid tumor;Breast tumor;Head and neck tumor;Brain tumor;Neoplasm;Hepatocellular carcinoma;Ovary tumor;Prostate tumor;Pancreas tumor;Urinary tract t
Cancer

myelocytic leukemia
complication

sclerosis;Asthma;Cancer;Rheumatoid arthritis;Autoimmune disease;Arthritis;Bacterial infection;Bone disease;Osteoarthritis;Bone metastases;Immune disorder;Osteopor

ment of diabetes (type I and II)
tional supplementation, also for treating dietary shortage or imbalance

term treatment of acutely decompensated severe chronic heart failure (CHF). Also being investigated for use/treatment in heart disease.

bolism

estigated for the treatment of Herpes labialis infections (cold sores).

, known for its antioxidant activities, is protective against cardiovascular disease and some forms of cancer and has also demonstrated immune-enhancing effects.
tional supplementation and for treating dietary shortage or imbalance.

d arthritis;Diabetes mellitus;Non-insulin dependent diabetes;Autoimmune disease;Immune disorder

;Cancer;Rheumatoid arthritis;Cardiac failure;Atherosclerosis;Delayed hypersensitivity;Transplant rejection;Inflammation;Multiple sclerosis;Autoimmune disease

e disease;Reperfusion injury;Age related macular degeneration
e tissue disease;Viral infection;Inflammation;HIV infection

hy;Vascular disease;Diabetic macular edema;Inflammation;Cancer;Gastrointestinal disease;Age related macular degeneration

anthracis infection
thrombosis;Thrombocytopenia;Cerebrovascular disease;Wound healing;Ebola virus infection;Bone injury;Cerebrovascular ischemia;Restenosis;Thrombosis;Diabetic foot

ion;Cancer;Acute myelogenous leukemia;Myocardial infarction;Non-small-cell lung cancer;Solid tumor
ure;Raynauds disease;Cardiac failure;Thrombosis;Diabetic complication;Prostate hyperplasia;Erectile dysfunction;Urinary tract disease;Female sexual dysfunction;Va

sis disorder;Ulcer;Cancer;Lung embolism;Restenosis;Metastasis;Skin ulcer;Cardiovascular disease
bstructive pulmonary disease
s disease;Renal disease;Lipid metabolism disorder;Cardiac failure;Hyperlipidemia;Intermittent claudication;Myocardial infarction;Coronary artery disease;Glioma;Hy

lence suggests that NADH might be useful in treating Parkinsons disease, chronic fatigue syndrome, Alzheimers disease and cardiovascular disease.
ion;Cancer

;Genital tract inflammation;Otitis media;Wound healing;Atopic dermatitis;Dermatitis;Cystitis;Inflammatory bowel disease;Cystic fibrosis;Alpha-1 antitrypsin defici
sease;Rheumatoid arthritis;Cancer

;Transplant rejection;Rheumatoid arthritis;Respiratory tract inflammation;Autoimmune disease

ldrich syndrome

abolism disorder;Atherosclerosis
ment of mucositis (mouth sores)

X;Lipid metabolism disorder;Non-insulin dependent diabetes;Atherosclerosis;Hypertriglyceridemia;Hyperlipidemia;Cardiovascular disease;Congestive heart failure;Dia
tional supplementation, also for treating dietary shortage or imbalance.
rosis;Non-insulin dependent diabetes;Atherosclerosis;Hypertriglyceridemia;Hyperlipidemia;Liver disease;Primary biliary cirrhosis;Non-alcoholic steatohepatitis

lence suggests that NADH might be useful in treating Parkinsons disease, chronic fatigue syndrome, Alzheimers disease and cardiovascular disease.

eplacement or supplemental therapy in patients with hypothyroidism of any etiology, except transient hypothyrodism during the recovery phase of subacute thyroidit
lupus erythematosus;Asthma;Rheumatoid arthritis;Allergy;Allergic rhinitis;Inflammation;Thrombocytopenic purpura;Acute myelogenous leukemia;Lymphoma

tional supplementation, also for treating dietary shortage or imbalance
in tumor;Leukemia;Breast tumor;Lung tumor;Head and neck tumor;Uterine cervix tumor;Ovary tumor;Prostate tumor;Prostate hyperplasia

is claimed to act as an effective antidepressant, however results are mixed. Tyrosine has also been claimed to reduce stress and combat narcolepsy and chronic fat

drug-induced;Mucositis;Cancer;Glioma;Metastasis;Renal cell carcinoma;Head and neck tumor;Brain tumor;Pancreas tumor;Solid tumor;Stomach tumor

thrombosis;Blood clotting disorder;Cancer;Ebola virus infection;Thrombosis;Lung embolism;Unstable angina;Angina;Myocardial infarction;Thromboembolism;Cardiovascu
reatment of ariboflavinosis (vitamin B2 deficiency).
reatment of hypercholesterolemia.

Cancer;Solid tumor

s disease;Immune deficiency;Breast tumor;Major depressive disorder;Glaucoma;Metabolic disorder;Psychotic disorder;HIV infection;Cocaine addiction;Inflammation;Pla
ne disease;Melanoma;Hyperthyroidism;Aging;Skin tumor;Acne;Breast tumor;Neurodegenerative disease;Acute myelogenous leukemia;Hepatocellular carcinoma;Prostate tumo
ll anemia;Inflammation;Transplant rejection;Myocardial infarction;Restenosis;Thrombosis
l tumor;Non-Hodgkin lymphoma;Neoplasm;Cancer;Trypanosomiasis;Pneumocystis carinii infection;Bacterial infection

ical neoplasm;Multiple myeloma

al treatment of cutaneous lesions in patients with AIDS-related Kaposis sarcoma.

n adjunct to the standard therapy of inhaled steroids with inhaled long- and/or short-acting beta-agonists.
tional supplementation, also for treating dietary shortage or imbalance
;Cancer;Rheumatoid arthritis;Pulmonary hypertension;Myeloid leukemia;Inflammation;Prostate tumor;Pancreas tumor;Myeloproliferative disorder;Solid tumor;Myelofibro
cositis;Migraine;Neuropathic pain;Irritable bowel syndrome;Reperfusion injury;Inflammatory bowel disease;Inflammation;Chronic obstructive pulmonary disease;Gastri

otting disorder;Chronic bronchitis;Lung injury;Cerebrovascular ischemia;Emphysema;Respiratory disease;Cystic fibrosis;Chronic obstructive pulmonary disease;Myocard

tional supplementation, also for treating dietary shortage or imbalance

ion

;Acne;Cancer

cell carcinoma;Psoriasis;Colon tumor;Cancer;Metabolic disorder;Leukemia;Breast tumor;Skin infection;Neutropenia;Carcinoma;Dermatological disease;Ocular disease;Ge
t rejection;Immune disorder
NONE

or;Melanoma;Cancer;Rheumatoid arthritis;Non-small-cell lung cancer;Neurodegenerative disease;Breast tumor;Colorectal tumor;Transplant rejection;Inflammation;Pancr

hy;Ovary tumor;Cancer;Renal cell carcinoma;Breast tumor;Solid tumor
tumor

tional supplementation and for treating dietary shortage or imbalance.

;Arteriosclerosis;Pain;Rheumatoid arthritis;Autoimmune disease;Allergy;Inflammation;Asthma;Multiple sclerosis;Immune disorder

uria

ment of acute malarial attacks in non-immune subjects.

tional supplementation, also for treating dietary shortage or imbalance

opic dermatitis;Atherosclerosis;Thrombosis;Bacterial infection;Fungal infection;Inflammation;Congestive heart failure;Chronic obstructive pulmonary disease;Viral
ion;Neuropathic pain;Renovascular hypertension;Congestive heart failure

s
tional supplementation and for treating dietary shortage or imbalance.
l tumor;Chronic lymphocytic leukemia;Cancer;Prostate tumor;Pancreas tumor;Metastasis;Multiple myeloma
NONE

ry disease;Asthma

l infarction;Arthritis;Inflammation;Cancer

cell carcinoma;Biliary cancer;Cancer;Non-small-cell lung cancer;Gastrointestinal tumor;Renal cell carcinoma;Breast tumor;Lung tumor;Head and neck tumor;Colorectal
nutritional supplementation, also for treating dietary shortage or imbalance. It has been claimed that ornithine improves athletic performance, has anabolic effec

Breast tumor;Cancer

eplacement or supplemental therapy in patients with hypothyroidism of any etiology, except transient hypothyrodism during the recovery phase of subacute thyroidit

tional supplementation, also for treating dietary shortage or imbalance

Metabolic disorder;Neurodegenerative disease;Cancer;Cardiovascular disease

ovirus infection;Metastasis;Cancer;Solid tumor
ng injury;Atopic dermatitis;Cerebrovascular ischemia;Shock;Ischemic heart disease;Dermatitis;Respiratory disease;Inflammatory bowel disease;Inflammation;Chronic o
neck tumor;Carcinoma;Melanoma;Cancer;Sarcoma;Non-small-cell lung cancer;Breast tumor
tumor;Ovary tumor;Cancer;Mastocytosis;Gastrointestinal tumor

;Septic shock;Alzheimers disease;Rheumatoid arthritis;Sepsis;Neurological disease;Osteoarthritis;Inflammation;Diabetes mellitus;Metastasis;Cardiovascular disease

ied indication;Gynecological disorder;Musculoskeletal disease;Estrogen deficiency;Cancer;Contraception;Amenorrhea;Female infertility;Carcinoma;Menopause;Premenstru

reast disease;Atrophy;Crohns disease;Testosterone deficiency;Uterine fibroids;Fibrocystic breast disease;Arthralgia;Prostatitis;Prostate hyperplasia;Inflammatory l
y;Diabetes mellitus;Diabetic cataract;Cataract

mall-cell lung cancer;Renal cell carcinoma;Colorectal tumor

ementia;Nicotine dependence;Cerebrovascular ischemia

oriasis;Non-Hodgkin lymphoma;Viral infection;Cancer;Rheumatoid arthritis;Leukemia;B-cell acute lymphoblastic leukemia;Cutaneous T-cell lymphoma;Ocular disease;Tran
on;Atherosclerosis;Prostate hyperplasia;Fatigue;Male sexual dysfunction;Autoimmune disease;Testosterone deficiency;Uterine fibroids;Metabolic disorder;Myalgia;Obes
se;Prostate tumor;Alzheimers disease;Dementia;Osteoporosis

etic nephropathy;Fibrosis;Solid tumor;Angiogenesis disorder;Glioma;Cancer;Atherosclerosis;Glomerular disease;Lung tumor;Nephritis;Immune disorder;Bone marrow trans

a;Liver tumor;Pancreas tumor;Lung tumor;Psoriasis;Bladder tumor;Esophageal disease;Mesothelioma;Atherosclerosis;Multiple myeloma;Bacterial infection;Uterine cervi

It may be of limited benefit in some with asthma and rheumatoid arthritis. It may be helpful in some neurological diseases including Alzheimers, some eye disorders

ducing disease severity and may play a similar role in type 2 diabetics in slowing the progression of diabetic nephropathy.

osis;Corneal disease;Cardiovascular disease;Ulcerative colitis;Alzheimers disease;Infertility;Syndrome X;Lipid metabolism disorder;Non-insulin dependent diabetes;N

order

umor;Stomach tumor;Retinopathy;Bladder tumor;Colon tumor;Cancer;Mesothelioma;Non-small-cell lung cancer;Cholangiocarcinoma;Gastrointestinal tumor;Liver tumor;Lung

osis

It may be of limited benefit in some with asthma and rheumatoid arthritis. It may be helpful in some neurological diseases including Alzheimers, some eye disorders

ulcer;Ischemic heart disease;Marburg virus infection;Myocardial disease;Embolism and thrombosis;Myocardial infarction;Cartilage disease;Thromboembolism;Coronary a

scular disease;Viral infection;Cancer;Atherosclerosis;Pulmonary disease;Metabolic disorder;Hypertension;Cardiovascular disease;Pulmonary hypertension;Angina;Male

percholesterolemia;Atherosclerosis;Peripheral arterial occlusive disease;Cardiovascular disease;Congestive heart failure;Angina;Hypertension;Heart disease;Cerebro

ency;Dermatological disease

betes mellitus;Obesity;Inflammation

is.

igue, however these claims have been refuted by some studies.

lar disease

nned abortion;Diabetes mellitus;Weight gain;Endometriosis;Obesity;Insulin dependent diabetes;Cushings disease;Glioma;Endometroid carcinoma;Neurodegenerative disea
r;Pancreas tumor;Alzheimers disease;Multiple sclerosis;Psoriasis;Colon tumor;Cancer;Renal disease;Leukemia;Hyperparathyroidism;Sarcoidosis;Carcinoma;Insulin depen

sis;Hematological neoplasm;Cardiovascular disease
c motility disorder;Uveitis;Insulin dependent diabetes;Multiple sclerosis;Rheumatoid arthritis;Sepsis;Osteoarthritis;Transplant rejection;Gingivitis;Allergic rhin

ial infarction;Lung inflammation;Pulmonary fibrosis;Psoriasis;Pancreatitis;Bronchitis;Rheumatoid arthritis;Arthritis;Pulmonary disease;Lung tumor;Respiratory dist

nital system disease;Lymphoma;Immune disorder

eas tumor;Solid tumor

infection;HIV infection;Cardiovascular disease

tumor;Adenocarcinoma;Prostate tumor;Pancreas tumor;Hepatocellular carcinoma;Stomach tumor
ts, has wound-healing effects, and is immuno-enhancing.

is.

bstructive pulmonary disease;Myocardial infarction;Injury;Psoriasis;Reperfusion injury;Sepsis;Skin burns;Respiratory distress syndrome;Transplant rejection;Hypert[illegible]

ual syndrome;Anesthesia;Age related macular degeneration;Osteoporosis

bowel disease;Solid tumor;Gynecological disorder;Vascular disease;Contraception;Cancer;Sepsis;Hypercholesterolemia;Neurological disease;Ataxia;Cognitive disorder;(

nsplant rejection;Immune disorder
sity;Gynecological disorder;Contraception;Cancer;Hypercholesterolemia;Skin burns;Seborrhea;Growth disorder;Bone tumor;Muscular dystrophy;Erectile dysfunction;Syste

splantation

x tumor;Nasopharynx tumor;Carcinoma;Restenosis;Stomach tumor;Thyroid tumor;Small-cell lung cancer;Renal disease;Nervous system tumor;Hepatobiliary system tumor;Bre

s including cataracts, and diabetes and premenstrual syndrome. It may also help protect skin from ultraviolet irradiation although claims that it reverses skin agi

Metabolic disorder;Diabetic complication;Hyperlipidemia;Lacrimal gland disease;Myocardial infarction;Obesity;Coronary artery disease;Insulin dependent diabetes;Vas

tumor;Colorectal tumor;Uterine cervix tumor;Carcinoma;Biliary tumor;Gallbladder disease;Keratosis

s including cataracts, and diabetes and premenstrual syndrome. It may also help protect skin from ultraviolet irradiation although claims that it reverses skin agi

rtery disease;Hemophilia;Blood clotting disorder;Bleeding;Atrial fibrillation;Skin burns;Disseminated intravascular coagulation;Connective tissue disease;Lung embo

sexual dysfunction

vascular ischemia;Osteoporosis

se;Hypertension;Hepatitis C virus infection;Carcinoma;Non-insulin dependent diabetes;Psychiatric disorder
dent diabetes;Transplant rejection;Osteoporosis

itis

ress syndrome;Transplant rejection;Pulmonary hypertension;Pneumonia;Inflammation

ension

)steoporosis;Gynecomastia;Major depressive disorder;Acne;Female contraception;Dysmenorrhea;Neoplasm;Central nervous system disease;Multiple sclerosis;Alcoholism;La

emic lupus erythematosus;Asthma;Wound healing;Cerebrovascular ischemia;Acne;Hypogonadism;HIV infection;Neoplasm;Dermatological disease;Prostate disease;Multiple sc

east tumor;Head and neck tumor;Ovary tumor;Prostate tumor;Renal tumor;Solid tumor;Colon tumor;Glioma;Cancer;Non-small-cell lung cancer;Metastasis;Renal cell carcin

ing, enhances male fertility and exercise performance are poorly supported. It may help relieve some muscle cramps.

scular disease;Cancer;Cognitive disorder;Hypertension;Diabetes mellitus;Osteoporosis;Dermatological disease

ing, enhances male fertility and exercise performance are poorly supported. It may help relieve some muscle cramps.

olism;Cardiovascular disease;Sepsis;Angina;Infarction

crimal gland disease;Carcinoma;Cardiovascular disease;Menopause;Unidentified indication;Alzheimers disease;Breast tumor;Scar tissue;Hyperlipidemia;Postmenopausal

lerosis;Lacrimal gland disease;Alopecia;Mycobacterium tuberculosis infection;Ovary cyst;Carcinoma;Cardiovascular disease;Cachexia;Muscle wasting disease;Cervical

oma;Glioblastoma;Colorectal tumor;Neoplasm;Sarcoma;Hypercholesterolemia

osteoporosis;Papillomavirus infection;Ovary tumor;Prostate tumor;Adrenal disease;Urinary dysfunction;Estrogen deficiency;Endometriosis;Hormone deficiency;Musculosk

dystonia;Anemia;Endocrine disease;Non-insulin dependent diabetes;Breast tumor;Female contraception;Fungal infection;Cystic fibrosis;Prostate tumor;Estrogen deficie

keletal disease;Glioma;Vagina disease;Non-small-cell lung cancer;Parkinsons disease;Uterus tumor;Psychiatric disorder

ency;Endometriosis;Hormone deficiency;Turners syndrome;Keratosis;Premenstrual syndrome;Hepatitis C virus infection;Benign tumor;Osteoporosis;Male contraception

Table 3S

GENE symbol	T1	T2	T3	T4	T5	T6	T7	T8	T9	T10	T11	T12	T13	T14	T15	T16	T17	T18	T19	T20	T21	T22	T23	T24
OTOP2	0.2691471	0.0304698	0.2206358	0.0685794	0	0	0.6324332	0.0149801	0.0277323	0	0.0343345	0.028042	0.0140016	0.2835252	0.0350011	0	0	0.1932984	0.0420733	0.014998	0.2297307	0.6681331	0.013023	0.9296122
OTOP3	0	0.0168214	0	0	0.0176911	0	0.009188	0	0	0	0	0.0232216	0	0	0	0	0	0	0	0	0.0724724	0.0207605	0	0.1139235
AQP8	0.0619026	35.278701	23.723399	25.821897	0.1586179	0	3.5952697	0.2196419	0.6505857	2.1453469	0.6880073	0.2192841	2.8193736	23.931885	0.5816199	2.30127	0.5882218	7.0077969	9.9831054	0.0585939	4.1061916	17.017059	0.4964596	9.9174678
KRT24	0.1169107	0.01125	0.1396506	0.0101283	0	0.0138376	0	0	0	0	0	0	0	0	0	0	0	0.0519023	0	0	0	0.1945701	0.0096167	0
CA1	1.2541519	0.0572207	9.8366115	16.452168	0.0109417	1.2540754	1.8639153	0.1278725	0.2777588	0.9853154	0.5216898	1.0388671	1.5872182	6.5260231	0.0597549	0.4019303	0.2739637	1.631937	19.83047	0.0972209	4.0789066	23.822015	0.0800396	4.2158618
CA7	0.4960354	0.1363776	3.9501162	4.6957531	0.8366696	0.5172144	1.7877683	0.2011457	0.3034164	0.39415	0.5506708	0.7739863	0.2088958	1.1445983	1.4099331	0.9756846	0.2394166	0.3145915	0.3389636	0.2682986	0.8323818	2.2603884	0.2040107	2.565625
KRTAP13-2	0	0.0170655	0.1765341	0.184368	0	0	0.0186426	0	0	0.0455277	0	0	0	0	0	0	0	0	0.2827734	0	0.0367622	0.3162325	0	0.0385258
[illegible]	0	0	0	0	0	0	0	0	0.0056802	0	0	0	0	0	0	0	0	0	0	0	0	0	0	0
SST	0	0.3766568	3.1678972	0.770696	0.2160719	0.0421173	1.6458774	0.0673379	0.0415335	0	0.2700927	0.6302643	0	3.2237022	0	0	0	0.5529107	0.7565027	0.4041844	0.4425742	1.2690234	0.1463597	1.3914157
TMIGD1	0	0.1033938	3.3869244	4.4370426	0.0724932	0	3.2378995	1.5249729	0.0209121	0.5516717	0.1359261	1.5066295	1.0769363	6.7157816	0.0791801	1.1835341	0.1361341	1.6695381	4.5114793	0.2881624	2.1901657	9.4732392	0	3.8513286
BEST4	0.3008038	0.8682665	1.6896218	0.4467343	0.1304665	0.3178855	1.5019901	0.3760987	0.1881763	0.882531	0.815424	0.2663921	0.8550749	1.0411539	0.4275029	0.1775012	0.2450014	0.2384666	0.3996862	1.1897475	1.5365778	1.251541	0.4506773	2.6371417
DAO	0	0.0054497	0.0450996	0.0883143	0.011463	0	0	0.0053586	0.0066135	0	0.0491276	0.0050155	0.0200342	0.0357955	0	0.0233933	0	0.0125712	0.0240803	0.032164	0.0117397	0.0605915	0.0139755	0.0492114
MYOC	0	0.2020168	0.2925663	0.0363749	0	0.0869686	0	0.1291152	0.0245156	0	0	0.009296	0.0371326	0	0	0	0.3058847	0.4194053	0	0.0794864	0	0.0623911	0.0172687	0
PYY	0.0744823	0.0179181	9.6754434	3.6779929	0.0753782	0	3.5820734	0.1409477	0.065233	1.147253	0.1211448	1.7809637	0.691638	2.8638343	0.0823312	0.4614881	0	0.2066643	4.9087484	0.0332506	1.157963	7.6588412	0.1072165	6.6338678
PNLIPRP3	0	0	0	0	0	0	0.0098203	0	0	0	0	0	0	0	0	0	0	0	0	0	0	0	0	0
GUCA2B	0	0.248122	16.793508	3.3507413	0.5964599	0	2.9428799	0	0.0960304	3.4988493	0.2795934	0.7502996	1.6939808	11.641042	0.2443044	2.8909356	0.6067125	2.3712049	12.216569	0	12.904337	12.041906	0.1617985	19.124779
MS4A12	0.2869939	2.126784	4.5708794	1.8647332	0.3388534	0.2830719	9.7546856	0.0678871	0.097749	0.3990795	2.2691312	0.5189042	3.8071523	10.102703	1.0838934	5.6309578	0.2272602	1.0617534	18.062645	0.0339568	4.0156569	25.374258	0.629523	19.5348
CCDC169-SO	0	0	0	0	0	0	0	0	0	0	0	0	0	0	0	0	0	0	0	0	0	0	0	0
SLC30A10	0	0.047677	0.0910512	0.3598957	0.0154284	0.009022	0.1883022	0.0360614	0	0.0097841	0.0041326	0.027062	0.0606703	0.2408909	0.0168515	0	0.0386303	0.1015198	0.1053335	0	0.0474822	0.1585729	0.003135	0.1655873
PCSK2	0	0.0657739	0.2536025	0.0161498	0.0062686	0.0073548	0.0261284	0.0058795	0	0	0.0101068	0.0302666	0	0	0	0	0.0118093	0.0068966	0.0033026	0.0117635	0.0064404	0.0664812	0.0127783	0.0134967
HSD3B2	0	0.0090588	0.1874163	0.0733999	0.0381086	0	0.3265686	0.0089073	0.0219864	0.0966685	0	0.1634134	0.1332069	0.3768389	0.1248714	0.1166561	0.0238545	1.5045446	0.0900614	0	0.5463973	0.0559544	0	1.0020655
LCE2A	0	0	0	0	0	0	0	0	0	0	0	0	0	0	0	0	0	0	0.0394864	0	0	0	0	0
KRT25	0	0	0	0	0	0	0	0	0	0	0	0	0	0	0	0	0	0	0	0.0124644	0.0272965	0	0	0
GCG	0.0368511	0.0068652	1.1168135	1.205176	0.3356493	0.0961383	2.0337621	0.0174339	0.0215166	0.212857	0.149645	0.4242601	0.4399677	1.7080681	0.0407345	0.3044365	0	0.2044999	1.4591597	0.0174407	0.3628478	3.5702781	0.0833592	2.1614451
DHRS7C	0	0	0.130558	0.0378755	0	0	0	0	0.0255269	0	0.0237031	0.019359	0	0.046855	0	0	0	0	0.046473	0	0.0453133	0	0.0179811	0
CD177	0.2989567	0.8545732	9.4002815	14.29804	0.2135665	0.0208144	2.7544561	0.0998356	0.1129471	2.4152825	1.6589712	1.5184558	4.0747135	3.1122216	0.5831657	1.4164798	0.5124577	2.6544261	33.021662	0.0832287	2.3338261	52.398744	0.600313	12.11012
LINC01289	0	0	0	0	0	0	0	0	0	0	0	0	0	0	0	0	0	0	0	0.0112152	0	0	0	0.0128696
LINC00307	0	0	0	0	0	0	0	0	0	0	0	0	0	0	0	0	0	0	0.0551319	0	0	0	0	0
CLCA4	0.1786322	0.3069519	4.1405362	12.861186	0.0129129	0.6418391	41.043489	0.0241455	0.1713494	0.8352697	2.4627026	1.2373258	3.0918524	47.63513	1.2975705	2.6879305	0.24249	2.9890413	13.569842	0.1690844	15.221497	23.722802	0.1311934	45.263461
CHAT	0	0.0212037	0.0584909	0.0063632	0	0	0.0077211	0	0	0	0	0.0065047	0	0.0389495	0	0	0.009306	0	0	0.0069524	0	0.0087314	0.0120835	0
SLC17A8	0	0	0.0102482	0.0089192	0.0416768	0	0.0324678	0.0243532	0.0781463	0	0	0.0273526	0	0.0542268	0	0	0.0260881	0.0226531	0	0.0048725	0.0106707	0.0183581	0	0.0335477
ABCB11	0.1280775	0.0205409	0.0339976	0.0776701	0	0	0.040391	0.0121185	0	0	0.0046293	0.0604936	0.0151025	0.0449732	0.0188766	0	0.0594997	0	0.0181525	0	0.0796479	0.0507511	0.0140469	0.0741944
LGI1	0	0.0329187	0.1078336	0	0	0	0.0179806	0	0	0	0	0.0126233	0.0050423	0	0	0	0.0144476	0.006328	0	0.0053968	0	0.0237222	0.0070349	0
PABPC1L2B	0	0.0096319	0	0	0	0	0	0	0	0	0	0	0	0	0	0	0	0	0	0	0	0	0	0
LINC00682	0	0.0092691	0.0383534	0.0166898	0	0	0	0	0	0	0	0.0085305	0	0	0	0	0	0.0213816	0	0.0273526	0	0.0114507	0	0.0209251
BMP5	0.0076704	0.3985726	1.7162933	0.2358993	0.0310505	0.1906515	0.9312956	0.0217727	0.0044786	0.0984555	0.170502	0.4143653	0.2509883	0.6383286	0.0763079	0.2376253	0.699711	0.5448384	0.4117491	0.1125358	0.4610984	1.1306569	0.0220826	0.6998331
PLP1	0.1519606	0.2820109	1.5126514	0.0332861	0.0678792	0.0599537	0.1255124	0.1027019	0.0126753	0.0092883	0.0196161	0.1954566	0.0255982	0.1600787	0	0.0696705	0.6417727	0.2650322	0.0269219	0.1472633	0.0225801	0.4301067	0.220234	0.0550187
SCNN1G	0	0.0543061	0	0.1684038	0	0.0074219	0.5900697	0.0296655	0.0073225	0.2092693	0.1835825	0.072192	0.0443643	0.0264222	0	0.2331127	0.0317788	0.0278381	0.026662	0.1839978	0.6239198	0.1043588	0.0206318	0.0272438
ABCG2	0.0544329	0.2618968	0.668266	0.3969033	0.1101753	0.2040185	0.8773815	0.9184788	1.843371	0.1280937	0.2016622	3.1615011	0.3770897	1.1372587	0.8423665	0.2810527	0.7758631	0.4531013	1.1957986	0.3950136	0.5829783	0.8465915	0.425359	0.9755408
LINC01445	0	0	0	0	0	0	0	0	0.0224064	0	0	0	0	0	0	0	0	0	0	0	0	0	0	0
SPIB	0.1438321	0.0543737	0.623828	0.3204153	0.280727	0	1.0421863	2.1871637	0.0659847	0.065936	0.1002607	1.5922235	0.3270896	0.7575787	0.2157709	0.1697477	0.1887412	0.2290501	0.1638117	0.0680722	0.2662862	0.5373711	3.6127197	0.5914302
RIT2	0	0.01485	0	0	0	0	0	0	0	0	0	0	0	0.0325132	0	0	0	0	0	0	0	0	0	0
PPY	0	0	0	0.0579069	0.0450971	0	0.0702647	0	0	0.057198	0	0	0	0.1408248	0	0	0	0	0.0710515	0	0.1385567	0.132431	0	0
HTR3E-AS1	0	0	0	0	0	0	0	0	0	0	0	0	0	0	0	0	0	0	0	0	0	0	0	0
CA4	0.1632988	0.1745979	11.17991	5.6273813	0.3121633	0.0107376	7.9441177	0.0858391	0.127129	3.0742499	0.2951154	4.5072481	1.2355707	23.938217	0.7019721	6.1631599	0.2068968	8.1356858	24.629595	0.0856725	17.698213	14.623924	0.2238732	13.4802
GUCA2A	1.5459335	4.3140713	69.633236	48.448708	19.791279	0	15.072834	1.1336198	5.4609628	38.595368	2.3887234	11.842545	31.787046	103.08521	3.9303389	17.241325	11.60513	120.27661	102.91163	9.6578053	72.022923	136.78359	4.6414531	83.118135
PRIMA1	0.0678054	0.6415799	2.2947617	0.0832151	0.3659677	0.1337537	0.2375858	0.1122702	0.0197945	0.1015367	0.0122535	0.1801403	0.0099939	0.1071363	0	0.0233392	0.1002234	0.1755903	0.0600615	0.1818414	0.0937002	0.5440626	0.1719659	0.0736464

[illegible]

NBGPMD	0	0.0436121	0	0	0	0	0.0478615	0	0	0.1168828	0	0	0	0	0	0	0	0	0	0	0	0	0	0
LINC01634	0	0	0.0184829	0	0	0.2516413	0.0097171	0	0	0	0	0	0	0	0	0	0	0.0205188	0	0	0	0	0.0152072	0
NESN1	0	0.1162985	0.1569292	0.0091046	0.0106358	0.0373168	0.0331426	0.0049719	0	0	0	0.032575	0	0.0110708	0	0.0217052	0.0266304	0.0116641	0.0055856	0.0198953	0.021785	0.0374794	0	0.011415
NECAB1	0.0686358	0.2113485	0.3621043	0.0743265	0	0.6052186	0.2200598	0.0616949	0.024045	0.00881	0.0483754	0.139803	0.0303499	0.1301442	0	0.0567018	0.4261058	0.0914122	0.0656627	0.1591697	0.0071138	0.1183075	0.1072691	0.0298282
CNTN1	0.2406906	0.186418	0.5434573	0.1169731	0.0631756	0.3543651	0.151193	0.0833185		0	0.1241261	0.2157553	0.0778759	0.055657	0.0064891	0.2061153	0.8590692	0.0325775	0.1497657	0.1139129	0.0304225	0.3384627	0.2849034	0.0063764
RNF150	0.6872782	0.6459693	1.0699502	0.3911699	0.0444825	1.1374299	0.7602788	0.2495301	0.0233307	0.0410316	0.2361352	0.3432527	0.1802232	0.2188816	0.0883376	0.1485466	0.954303	0.2084368	0.1571563	0.313925	0.0628294	0.6555065	0.7263847	0.0520817
TRPV3	0.611606	0.0259647	0.1701081	0.0779197	0.0728193	0.0638735	0.0969122	0.2978566	0.0157546	0.1962628	0.3267151	0.1075316	0.0357942	0.1942316	0.0745653	0.1393194	0.0911644	0.4242536	0.188824	0.4107762	0.3635625	0.1737446	0.1683123	0.0390773
KIAA1644	0.3257429	0.7064055	1.7704139	0.524693	0.3955931	0.2159079	1.0033046	0.2065022	0.0636856	0.0636238	0.6216527	0.2855935	0.10947	0.4392247	0.165632	0.2152836	0.5200156	0.1518436	0.2596941	0.3329986	0.3713795	0.7976934	1.2657514	0.1202967
CPB1	0.0231361	0	0.1074744	0.0167029	0.5853606	0.013692	0.0040535	0.0036485	0.0225145	0.0098991	0.0041812	0	0.0136407	0.0406202	0.0255742	0.0477834	0.009771	0	0.0286922	0	0	0.0091678	0.057093	0
MAPT-IT1	0	0	0	0.0063168	0	0	0	0.020697	0	0	0	0	0	0	0	0	0.0092381	0	0	0	0	0	0	0
CTSG	0.1920603	0.0924073	5.9266117	0.2218499	2.8507658	0.0378871	1.6992917	0.3937355	1.2522302	0.0410676	0.2429655	1.0347069	0	1.2813619	0.2476827	0.338054	1.0747391	0.8881753	0.4423394	0.2272434	0.3981235	0.9132535	7.5568106	0.3824537
C10orf105	0	0	0.0177464	0.0115837	0.0090212	0	0	0	0	0	0.0096657	0.0039471	0	0.0093902	0.0098534	0	0.005647	0	0	0	0	0	0.0256633	0
EPHX2	0.0397546	0.1147643	0.1055269	0.063141	0.0536437	0.0392113	0.2925333	0.0940377	0.0618983	0	0.0933964	0.1232234	0.0585966	0.0837566	0.1171837	0.3264227	0.1594998	0.0147075	0.5845744	0.0689878	0.0624076	0.055135	0.1635036	0.0863606
NCR2	0	0	0	0	0.0365142	0	0.018964	0	0.0210665	0	0	0	0.0319085	0	0	0	0	0.0400444	0.0191763	0	0	0	0	0
NBGPMX5	0	0	0	0	0	0	0	0	0	0	0	0	0	0	0	0	0	0	0	0	0	0	0	0
LMNTD1	0.0142659	0.0068639	0.0142006	0	0.0433125	0.0337703	0	0	0	0	0	0	0	0	0	0	0	0.0158333	0.0227466	0	0	0	0	0
LOC389895	0	0.0258379	0	0	0	0.1271231	0	0	0	0	0	0	0	0	0	0	0	0	0	0.0254157	0	0.0319193	0.0441733	0
FAM129A	4.1239512	3.6170292	4.9208362	1.1673649	0.4678634	2.1825109	3.3052036	1.866503	0.4232978	0.1753242	0.8516188	2.2703971	1.5796438	1.5993501	0.9581625	1.0416005	7.7942006	1.1602973	0.8711528	1.4420674	1.0127612	3.091312	2.9795345	0.8958665
LINC01324	0	0	0	0	0	0.0308394	0	0	0	0	0	0	0	0	0	0	0	0	0	0	0	0	0.0214324	0
HLF	0.1398997	0.4102759	0.5702998	0.1731417	0.0337101	0.8673526	0.483211	0.2363762	0.1129925	0.0427555	0.1264142	0.3126883	0.2592308	0.1052666	0.3902876	0.0550356	0.4979898	0.1922398	0.3753179	0.1229634	0.2209519	0.205904	0.2137137	0.1374836
TSPAN8	0.466688	17.786348	6.6867254	2.95878	3.5064795	7.0553037	7.5966792	6.6637229	5.3259298	8.8402882	0.7667308	13.770384	1.4507984	2.0409629	1.6570282	5.95839	0.7615045	2.3700744	2.4052409	1.2248347	5.8044105	5.1191018	6.531798	2.3194735
CYP4B1	0	0.006116	0.1138798	0.3138518	0.0900508	0	0.0133625	0.4450139	0.1707056	0.0489489	0.2687768	0.0281433	0.0674504	0.0267811	0.0562041	0.2625325	0.0563684	0.1692975	0.1060968	0.1323529	0.0790494	0.0679991	0.0993324	0.0138069
HMGCS2	3.7121947	60.95695	21.149484	33.03421	252.83393	7.9978553	28.682823	5.6085311	6.9618845	33.749679	37.40498	24.330704	120.27477	53.990166	21.481306	170.64918	69.357243	109.05806	36.414067	0.1052514	110.00009	97.602463	5.2416446	27.035461
[illegible]	27.948776	21.161264	10.016263	12.684237	4.0305074	7.6476164	15.576568	15.115559	8.6573796	4.8815199	20.190991	17.498686	8.8931999	12.971156	15.127995	7.2451769	2.1979292	5.3953192	8.3680256	4.3798137	11.107141	21.296981	4.106809	15.645345
ZNF536	0.050958	0.022066	0.1268117	0.0573903	0.0154713	0.0603139	0.1285622	0.0265185	0.0148766	0	0.0249648	0.0586669	0.0135196	0.0912563	0	0.0315733	0.1646353	0.0452454	0.051459	0.0192937	0.1478837	0.0617786	0.0062874	0.0166048
CP	0.5847362	0.5597758	0.4800488	0.0861698	0.0183021	0.1391324	0.2122669	0.8926424	0.0422369	0.2869184	0.0392193	0.1654962	0.0906303	0.1714565	0.5663939	0.0747009	1.0196247	0.2475497	0.2050521	0.2111221	0.1874389	0.6377817	0.6495758	0.1113107
CD163L1	0.7652371	0.1971084	1.6234931	0.9743313	0.3441962	0.6998866	1.1335096	0.7752496	2.9245487	0.8235001	1.6511527	0.3936096	0.5537098	0.9261129	1.1961901	1.516598	0.9744392	0.8493141	0.8668405	0.8267667	1.8025759	2.003139	2.2094588	0.6464744
JPH2	1.3543111	2.802274	7.1530913	0.4744407	1.3481319	0.4116903	0.7974095	0.4481488	0.3024743	0.1994855	0.1324072	1.0125908	0.1308982	0.6314722	0.1799705	1.1310587	2.7144807	0.5749601	0.3500687	1.3975069	0.429542	0.7345944	0.3317677	0.1286137
KRT9	0.1920603	0.0092407	0.0191181	0.0083194	0	0	0.0201896	0.0090862	0.011214	0	0.0208256	0.0340178	0	0	0.0424599	0	0.0486674	0	0.0306235	0.0090897	0	0.068494	0	0.1251667
ADCY5	0.426286	0.9396526	2.3437074	0.500278	2.287494	0.5280282	0.6513326	0.2274674	0.0376243	0.3053997	0.1236205	0.6255407	0.1578125	0.2610905	0.104104	0.2866465	1.070774	0.4621194	0.3740993	0.4527658	0.2722881	0.8632449	1.0193255	0.166903
PIK3C2G	0.0890771	0	0	0.0064308	0	0.0615019	0	0.0245826	0.0086684	0	0	0	0.0065648	0.0078196	0.0082053	0	0	0	0.0039453	0.0070263	0	0	0.0264728	0
LGALS9C	0.9344053	1.4199502	0.653852	0.5410041	0.149805	0.0383255	1.3177815	0.196957	0.0648214	0.4037547	0.0832694	0.6800332	0.1390911	0.2923726	0.7976638	0.171966	0.0234431	0.1129481	2.0110975	0.1357342	1.6972171	2.2160662	1.7388832	0.6129761
LINC01625	0	0.0125215	0	0	0	0	0	0.0123121	0	0	0	0	0	0	0	0	0.0329729	0.0966523	0	0	0	0	0.0107035	0
UGT1A9	0.0546132	0	0	0	0.0184234	0.0646402	0.086115	0	0.0106292	0	0	0	0	0	0	0	0.069194	0	0.0096755	0.0086157	0.018868	0.0216406	0.0224614	0
LOC339166	0.0968549	1.5401448	1.1280144	0.9607489	2.8768831	0.0286394	1.3999563	0.2130686	0.1470341	0.5221504	0.6406364	0.9263678	0.10707	0.7958256	0.0695899	0.5100912	0.435633	0.4998583	0.3577687	0.233779	1.0088762	1.4104297	0.6054879	0.5207467
MYOCD	1.4173293	1.3114023	1.8844094	0.6461362	0.0852602	2.0382853	1.0627352	0.2743061	0.1996335	0.0626938	0.1665814	0.3840174	0.0876539	0.8196081	0.1314702	0.153526	1.0642539	0.3850127	0.2949977	0.7247311	0.2722268	0.7304999	0.2140101	0.1022723
KCNQ5	0.0232401	0.0223633	0.0578342	0.0050334	0	0	0.0152689	0.016492	0.0033923	0.0074577	0.0125999	0.0385992	0.0154147	0.0061204	0.0192668	0.0599975	0.0036806	0.0064484	0.0092639	0.0164984	0.0240873	0.0483469	0.1481526	0
CHAD	0.6589949	0.7045926	0.9475237	0.8140702	0.5187166	1.2566461	0.5003145	0.5657952	0.0570035	0.5952462	0.3572829	0.3242259	0.172681	0.8227542	0.2697924	0.2520428	0.5257001	0.5959548	1.1156106	0.139616	0.8348001	2.1325509	0.7127167	0.4506787
PLAC8	38.354237	8.5897266	6.9198798	12.119096	1.1436726	0.2634597	10.307597	5.1464987	10.850544	3.0915857	4.4311586	8.8707294	3.2304306	21.428098	5.0849943	8.4164386	0.7954403	5.9418167	10.999369	0.4916196	16.066604	24.323353	1.492937	91.439929
BTNL3	1.2437479	6.8340475	11.411627	10.899875	6.1658432	2.8481966	3.9981208	1.9442988	8.0092333	6.3626893	5.7658783	8.1971303	2.1679964	6.8167843	4.0248155	4.6534959	1.0619573	0.4201215	5.8727246	4.0607281	16.178989	15.610132	0.1260296	4.072362
GABRA1	0	0.0038517	0	0	0	0	0	0	0	0	0	0	0	0	0	0	0	0	0	0.0075775	0	0	0	0
LINC01486	0	0	0	0	0	0	0	0	0	0	0	0	0	0	0	0	0	0	0	0	0	0	0	0
CCDC169	0	0.0071839	0.0396338	0.0043117	0	0.0412355	0.0261594	0	0.0058119	0.0127768	0.0026984	0.0088153	0	0	0	0	0.0441405	0.0110477	0.0052965	0.009422	0	0.0088747	0.0163756	0
[illegible]	0	0	0	0.0064254	0	0.0087786	0	0	0	0	0	0	0	0	0	0	0	0	0	0	0	0	0	0
GPT	0.2872542	0.5221212	4.3208576	3.5323817	32.866299	0.0188886	6.2322215	0.7096866	1.8263036	6.7597802	0.5969984	2.2683342	0.8326879	9.212474	6.8973455	3.526661	1.3344695	2.178569	3.1382786	1.4048195	8.58443	3.8131483	0.7351098	9.793594
GPR26	0	0.0056325	0.0407856	0	0	0	0	0	0	0	0	0	0	0	0	0.0120889	0.0074161	0	0	0.0027702	0	0.0034791	0.0048147	0
DCAF12L2	0	0.0744591	0.0616192	0	0	0	0.0325364	0.0292856	0	0	0	0.0137053	0	0	0	0.1917725	0	0.1030558	0	0.029297	0.0320796	0.0183968	0.0636487	0
DIRAS2	0	0.0337808	0.0299524	0.029968	0.0203014	0.0949727	0.0263593	0.0047451	0.0234254	0.0128744	0	0.0310892	0	0	0	0.0414304	0.0635396	0.0333962	0.1492646	0.0189879	0	0.0894249	0.0742534	0
MAB21L2	11.947439	8.3744251	25.159547	4.3298541	8.0296474	9.6902504	6.3713786	2.334453	0.5465815	1.2626888	1.6096133	2.845398	1.3189979	2.4963935	0.5787689	5.7673903	11.749928	2.5710176	1.9395617	6.7207323	1.8069342	5.3000498	3.4781424	0.4653111

[illegible]

ARHGAP20	0.6543259	0.1039501	0.4239791	0.1550847	0.0187414	[illegible]
SIGLEC11	0.0970289	0.0266767	0.2621584	0.0960674	0.028056	[illegible]
SCRG1	1.2715275	0.125493	1.2738197	0.0105919	0.0907374	[illegible]
ANKS1B	0.2865005	0.0191453	0.0633753	0.0103418	0	[illegible]
PRKACB	1.3331782	4.2416857	5.7376465	5.2511544	0.1851862	[illegible]
FAM163A	0.0151192	0.24733	0.40635	0.2292188	0.0765054	[illegible]
KCNIP4	1.4766839	0.3424649	0.5075995	0.3175226	0.0430056	[illegible]
NAAA	5.0797559	7.1897992	8.5616857	4.2768354	4.3522579	[illegible]
CSRP1	24.795714	38.969294	87.420886	23.095017	19.701582	[illegible]
SMPX	0	1.0173684	1.7680568	0.5861956	0	[illegible]
FAM151A	1.1406753	0.3271816	0.5022197	1.0737174	1.9535921	[illegible]
NCAN	0.0191087	0.0061293	0.0190212	0.0055181	0	[illegible]
PGM5P4-AS1	0.0612987	0.0442396	0	0.0265524	0	[illegible]
KIR2DL4	0.4336953	0.0736471	0.0507894	0.1768107	0.077455	[illegible]
UGT3A2	0	0.0157099	0.0487533	0.0070718	0	[illegible]
RERG	0.3174054	1.1250034	2.580277	0.7332733	0.3747603	[illegible]
KRT1	0	0	0.1935631	0.0536011	0.2325726	[illegible]
MIR5707	1.0859706	0	0	0	0	[illegible]
NMRAL2P	0.2343019	0.1771491	0.3998215	0.8989225	0.0677496	[illegible]
ZNF726	0.0069935	0.0942146	0.0208843	0.0333225	0.0070776	[illegible]
[illegible]	0.1184266	0.1205333	0.299245	0.0552442	0.0092193	[illegible]
TARID	0.0335654	0.0242243	0.0278431	0.0290786	0	[illegible]
LOC1053694	0	0	0	0.0243935	0	[illegible]
C14orf39	0	0	0.0126387	0	0.0256991	[illegible]
CPNE5	0.6625301	1.3077645	1.3781796	1.3061476	0.060173	[illegible]
FGFBP2	0.0703709	0.2200771	0.4202924	0.0304822	0.0356087	[illegible]
CASQ1	0.262759	0.252646	0.985865	0.0612865	0.122732	[illegible]
LOC1053738	0.309731	0	0.0770783	0.0670822	0	[illegible]
CPEB1-AS1	0.0175436	0.0042204	0.0087316	0	0	[illegible]
LOC1019296	0	0.024881	0.0257381	0.0448004	0	[illegible]
NLGN4X	0.2364296	0.091004	0.2765329	0.0896113	0.0418728	[illegible]
TRIM55	0	0	0.0142098	0	0	[illegible]
STON1-GTF2	0.0102236	0.0049189	0.0407071	0.0044285	0	[illegible]
PRAMENP	0.0723623	0	0.4609985	0.0125379	0	[illegible]
[illegible]	0.4019737	0.212273	1.0052133	0.3227604	0.36712	[illegible]
KCNG1	0.1941445	1.1676254	1.0951153	0.1086246	0.2537862	[illegible]
ERBB4	0.0033772	0.0048747	0.0033618	0	0	[illegible]
ATP6V0D2	0.0730595	0.1406064	1.8617602	0.1898806	0.1478765	[illegible]
OR2W3	0	0.0223929	0.2779711	0	0	[illegible]
PLIN4	0.4577826	2.8967003	20.823506	0.0660984	1.1652415	[illegible]
SLITRK2	0.1285854	0.0053798	0.0500857	0.0048434	0.0056579	[illegible]
TCEAL7	0.672619	1.078739	2.3061922	1.4729597	0.0756344	[illegible]
DMD	1.0248516	1.5535354	1.1641823	0.2778475	0.0390467	[illegible]
ETFDH	5.1979391	2.7646699	2.8355469	3.6593193	0.9411369	[illegible]
[illegible]	7.8292225	3.944985	37.305073	13.501243	18.010736	[illegible]
GPR55	0.1703896	0.0461141	0.1696095	0.0738067	0.0538872	[illegible]
[illegible]	0.0413557	0.0099489	0	0.0179138	0.0289266	[illegible]
FBXL22	0.6084921	0.9017251	2.6893362	0.3162926	0.1970595	[illegible]
NOL4	0.030935	0	0.0153967	0.0067	0.0704409	[illegible]
STAB2	0.3646209	0.0432885	0.0648458	0.057433	0.0575075	[illegible]
[illegible]	6.0761266	73.266097	117.19762	79.465015	101.98228	[illegible]
BTNL2	0	0	0	0	0.0303207	[illegible]
NECAB2	0	0.025192	0.3995835	0.0302403	0.035326	[illegible]
CES2	13.829204	126.45271	56.702367	120.85828	74.584574	[illegible]
CELA3A	0.4671271	0.0172886	0.2146001	0.0311297	0.2545553	[illegible]
CNTNAP4	0.0067852	0	0.0067542	0	0.0137337	[illegible]
LRCH2	0.4639996	0.3992691	0.6128731	0.2203139	0.0361216	[illegible]
SH3BGR	0.5295469	1.4720879	3.6312885	1.2106214	0.238185	[illegible]
DPYSL5	0	0.0777218	0.0865838	0.0080738	0.0125755	[illegible]
LRRC55	0.146335	0.0743187	0.1699426	0.0633871	0.0493651	[illegible]
ACKR1	0.8771301	4.7225992	24.010648	2.6957762	3.0857454	[illegible]
LOC1019285	0.1749939	0.1157695	1.4298325	0.1358104	0.1475823	[illegible]
LINC01354	0.1072727	0.0442396	0.1830542	0.0132762	0.015509	[illegible]
LOC1005073	0	0.001965	0	0	0	[illegible]
YWHAEP7	0	0.0070632	0	0	0	[illegible]
LOC1019291	0.1334805	0	0.0664347	0.0289095	0	[illegible]
ZBBX	0	0	0	0	0	[illegible]
CNR2	0.0418555	0.0080553	0.1333246	0.0326346	0	[illegible]
NACAD	0.1431175	0.7187169	1.1129869	0.2673467	0.2172584	[illegible]
OMD	0.8799954	0.5789321	0.965351	0.1711433	0	[illegible]
LOC1019292	0	0.0089213	0.0553716	0.0321271	0	[illegible]
GNG13	0.0893939	0.1290321	0.133477	0.0774445	7.7351122	[illegible]
[illegible]	0.4028429	0.6022342	0.6158193	0.3240663	0.2402449	[illegible]
[illegible]	0.4838805	0.7266578	0.5546449	0.6669087	0.1558136	[illegible]
ADAMTS9-AS	0.5351769	0.0380388	0.1210743	0.0052686	0	[illegible]
GGT3P	0	0	0	0	0	[illegible]
NOG	0	0	0.0410955	0.005961	0	[illegible]
SLCO2A1	4.9368186	3.9975281	8.1130063	6.5307607	2.8645995	[illegible]
GALNT15	1.3010995	0.290646	1.0638674	0.67765	0.2586471	[illegible]
SLC13A2	0	9.7016457	4.2070493	3.5787734	1.2555749	[illegible]
PHF24	0.1210578	0.0400437	0.1581609	0.0098321	0.0306285	[illegible]
PTX3	0.6801311	0.1308944	1.015526	0.1865855	0.0688312	[illegible]
LINC00334	0.0978461	0	0.0486991	0.0635752	0	[illegible]
FAM189A2	1.7526997	0.1686576	0.5343069	0.2372523	0.0443445	[illegible]
CERS1	0.0117191	0.0620234	0.12832	0.0152289	0.0237201	[illegible]
S100B	0.4252385	0.8330055	15.631557	0.9341481	0.952925	[illegible]
SPARCL1	24.577697	33.978526	82.159179	17.06239	4.6853496	[illegible]
BLOC1S5-T1	0	0	0.0132588	0.0173089	0	[illegible]
CXCL13	4.9483377	0.7098118	2.1721959	1.2780785	0.6220939	[illegible]
SLC9A9	0.7102519	0.4142161	2.0781522	0.4894526	0.4356332	[illegible]
ADRA1B	0.101224	0.0121756	0.2770915	0.2192331	0.0512207	[illegible]
PIGZ	3.6434634	7.5919576	8.0852724	15.242273	14.582804	[illegible]
CD160	0.8639086	0.0286448	0.0661503	0.1439287	0.0336269	[illegible]
FLVCR2	1.6650959	1.2103226	1.5327169	0.5118623	0.4892295	[illegible]
TMEM132B	0.1736529	0.0296471	0.0306683	0.0181985	0.0085036	[illegible]
LOC1019273	0	0.0064634	0.0133722	0	0	[illegible]
KCNB2	0.0122786	0.0118153	0.0122223	0.0319118	0	[illegible]
FAM216A	0	0.029188	0.1811605	0.0262777	0.0204647	[illegible]
EML1	1.3245449	1.9094836	2.5582466	1.0083368	0.8152898	[illegible]
CD36	0.3087206	0.2217592	3.3024703	0.4840533	0.0704076	[illegible]
KCNS2	0.2384952	0.0158274	0.024559	0.0178117	0	[illegible]
SYT5	0.1398337	0.0712367	0.278387	0.060571	0.0499467	[illegible]
LPAR1	2.8870002	0.7479456	3.1158967	2.0292742	1.0782289	[illegible]
EDIL3	3.1579828	3.702668	5.234122	3.9344474	0.4731893	[illegible]
GDNF-AS1	0.018974	0.0091291	0.0472179	0.0246566	0	[illegible]
NUDT10	0.0428255	0.1751419	0.2557768	0.1020277	0.1536922	[illegible]
TCF21	0.2765978	1.1531517	2.4434708	1.8461105	0.6063878	[illegible]
PPP2R2B	0.0965828	0.0801199	0.2486396	0.0346231	0.0235935	[illegible]
RYR3	0.3232748	0.0804059	0.1908952	0.0545884	0.0055451	[illegible]
ERICH1-AS1	0	0.0078339	0.0162075	0.0070528	0.0041195	[illegible]
CCL21	17.230968	24.894257	78.74796	5.1133515	19.750473	[illegible]
TRANK1	8.9128386	1.7986222	3.5174777	3.2760767	1.8199378	[illegible]
BRINP2	0	0.0481789	0.0221505	0.0337363	0.01126	[illegible]
ADAMTSL1	0.4941776	0.3164872	0.6448078	0.250219	0.2635776	[illegible]
LIFR-AS1	0	0.0290563	0.0601144	0.0261592	0	[illegible]
SFTPD	0.1664716	0.2082486	0.1988514	0	0.0336948	[illegible]
LIPI	0	0.0242675	0.0251035	0.0109239	0	[illegible]
GRIA1	0.086397	0.0166275	0.0057334	0.0174646	0.0058291	[illegible]
LINC01352	0.3657531	0.1539801	0.0455098	0.0396078	0.046269	[illegible]
FAM153A	0.0051573	0.0049628	0.0359361	0.0111699	0	[illegible]
ZNF229	0.2077252	0.0691921	0.1908685	0.0899792	0.0080655	[illegible]
TEX33	0	0	0	0	0.0385709	[illegible]
CA14	0.917961	0.0552081	0.0913759	0.084496	0.0348376	[illegible]
[illegible]	0.1852299	0.029707	0.1229213	0.044575	0.0624859	[illegible]
NKAPL	0.0536691	0.1291108	0.1335584	0.1162377	0.0271573	[illegible]
PGM5P3-AS1	0	0	0	0	0	[illegible]
NLRP7	0.0337111	0.0054066	0.0671136	0.0292049	0.0113722	[illegible]
SLC37A2	0.9388677	0.9798419	4.5628978	0.7475811	1.816483	[illegible]
KIAA0513	1.8166075	2.8208633	3.5521767	1.679417	0.5893138	[illegible]
MEI4	0	0	0	0.003969	0	[illegible]
P2RX1	0.6647125	0.8421863	0.6506415	0.3935041	0.6839194	[illegible]
[illegible]	18.532911	16.558561	15.732849	19.147725	5.7755409	[illegible]
MIR4432HG	0	0	0	0.0182484	0	[illegible]
BRINP3	0	0.0124259	0.5784264	0.2461127	0	[illegible]
MIR8071-2	0	0	2.0206364	0.8792934	0	[illegible]
RHBDL2	7.3643959	0.0605689	2.9134757	3.4012979	1.0032851	[illegible]
KLK15	1.0760076	0.0092448	0.4781614	0.6075793	0.330574	[illegible]
[illegible]	0.2448064	0.0275668	0.051648	0.0135372	0.0263565	[illegible]
RGS9	0.8642713	0.3465272	0.7766729	0.2859784	0.2186667	[illegible]
KCNN3	0.7762637	0.2396554	0.582752	0.1737291	0.1898535	[illegible]
ENTPD3	1.5815153	0.2515455	0.6765479	0.1472022	0.0661379	[illegible]
MIER3	7.7538639	9.543315	3.3881802	7.0182253	0.7646315	[illegible]
LINC00484	0.8939392	0	0.1779693	0.0387223	0	[illegible]
MIR6076	0.3892195	0.1872678	0	0	0	[illegible]
PAPSS2	20.057342	13.397469	14.968777	19.382645	10.016478	[illegible]
ZNF728	0.0401294	0.0193077	0.0399457	0.0086913	0	[illegible]
STXBP5L	0.0476122	0.053452	0.0236971	0.0137493	0	[illegible]
LRRC66	0.5552257	0.5597208	1.0527311	2.3134201	1.1104293	[illegible]
TDP2	18.223481	42.343126	24.447165	26.011718	9.0641358	[illegible]
HIF3A	0.688283	0.2148737	0.3817911	0.0705522	0.0744417	[illegible]
LOC1019289	0.7508534	0.0437895	0.1132448	0.2759638	0.0230268	[illegible]
PNLIPRP2	0	8.0262226	4.3669095	3.5222015	8.9926563	[illegible]
POU3F3	0	0.0255931	0.0441266	0.0038402	0.0089721	[illegible]
[illegible]	11.816008	31.167067	21.694195	27.115485	3.5606657	[illegible]
KCNIP1	0.3798813	0.0067694	0.1120421	0.0182835	0.0142389	[illegible]
DPEP3	0	0	0.1518648	0.0283221	0.1543983	[illegible]
KCNA3	0.7704093	0.0576601	0.391962	0.1038221	0.0346523	[illegible]
EEF1A2	0	1.3397211	3.2518882	0.4463678	0.5103434	[illegible]
PDE2	2.0244972	2.4880058	3.272794	1.9714162	0.9608937	[illegible]
[illegible]	0.026828	0.0851924	0.1709131	0.0185935	0.0271507	[illegible]
TMEM132D	0.0371992	0.005966	0.0185144	0.0080567	0.0188233	[illegible]
PAX5	0.457113	0.0226736	0.1923282	0.1061471	0.1001529	[illegible]
ITLN1	0.6524611	0.1569615	222.62199	27.350196	11.135209	[illegible]
ENAM	0.0472053	0.0032446	0.0738401	0.0146055	0	[illegible]
C1orf94	0	0	0	0.0073957	0	[illegible]
IL2	0	0	0	0	0	[illegible]
RETSAT	21.699689	27.07902	38.489633	22.338371	29.162645	[illegible]
CAND2	0.2890371	0.3978843	0.6713323	0.2156233	0.2600121	[illegible]
KIT	2.1030309	0.6314524	1.6212068	0.924657	4.3366646	[illegible]
TMEM255A	0.1710024	0.3784674	1.6681511	0.1678969	0.1153728	[illegible]
SIGLEC6	0.3076846	0.0657948	0.102092	0.1554911	0.1210943	[illegible]
MYO1A	7.4921693	8.5589158	16.464529	18.866768	35.223525	[illegible]
CALY	0.0146119	0.1265458	0.5963451	0.0189881	0.0687259	[illegible]
NEU4	0.1406968	1.1192136	6.8345687	5.2696974	18.406159	[illegible]
ERICH3	0.0200396	0	0.004987	0.0043402	0.0050701	[illegible]
SEC14L6	0	0.0311929	0.1161626	0.0224662	0.0656115	[illegible]
PPP1R3C	0.4182105	1.7271079	5.2210447	0.9963468	0.2645255	[illegible]
CD300LG	0.0092691	0	0.6827721	0.0080301	0.0093806	[illegible]
STBD1	0.5015469	1.0890007	1.945798	0.5292044	0.5075795	[illegible]
LY9	0.0820016	0.0371332	0.3265048	0.0731298	0.1073961	[illegible]
C7orf31	1.2379555	3.3641832	0.6846044	1.0277902	0.081203	[illegible]
RCAN2	1.9550104	1.6589245	4.1398005	2.3506255	0.5036246	[illegible]
CHRM4	0	0.2800961	0.8402602	0.1008675	0.3240364	[illegible]
TGFBR3L	0.2611057	0.0977103	0.2887893	0.0628343	0.0587214	[illegible]
LGALS4	92.320227	514.90275	470.19712	461.6623	724.49144	[illegible]
FAM46C	4.9480491	1.8876245	4.2326435	2.1764462	3.0881269	[illegible]
MYH15	0.6288015	0.1815237	0.1479454	0.1238066	0.2256202	[illegible]
GNG2	3.495654	0.9653437	1.9285637	1.2006029	0.2302645	[illegible]
SLITRK1	0	0	0.0064372	0.0036715	0.0171558	[illegible]
PPP2R3A	2.6601221	1.7389121	0.8658888	0.831385	0.1760668	[illegible]
LOC1005071	0.2317882	0.0464674	0.0576818	0.0334675	0.019548	[illegible]
ITGA8	0.5842429	0.2895338	0.6688034	0.3618948	0.2003319	[illegible]
NAP1L3	0.1333287	0.5693254	1.3935423	0.4981219	0.1180658	[illegible]
UGDH	5.7736317	16.536651	9.0699681	14.261437	8.4069882	[illegible]
AKR1C2	1.1351414	0.2241459	0.5290492	0.2472726	0.0199213	[illegible]
GGT6	3.4506449	19.226558	15.23521	21.60078	60.141005	[illegible]
LINC02232	0	0	0	0	0	[illegible]
ACAA2	8.0471448	20.752391	23.323105	18.02592	31.145289	[illegible]
NAT2	0.465768	2.9452894	2.4837625	4.813278	0.6397168	[illegible]
KIAA1211	4.4656526	1.1579626	1.9317961	2.1030915	0.689872	[illegible]
RDH5	1.061579	0.2553823	1.5116952	0.555636	2.0144019	[illegible]
C14orf132	1.0711111	1.0121066	1.7477008	0.7676985	0.3743692	[illegible]
PDE5A	2.8018895	13.957161	2.7994885	3.3189017	0.9540305	[illegible]
BMP2	9.2946838	5.717831	4.1579882	3.9732213	1.6934136	[illegible]
RSPO1	0.3132405	0	0.2692874	0.0493399	0.1296851	[illegible]
CYP2C8	0.3592446	0	0.0681143	0.2371231	0	[illegible]
TAS1R1	0.0912486	0.0292687	0.0302769	0.0461132	0.2636473	[illegible]
MFSD4A	11.804444	1.5094304	4.7636764	4.1722145	0.1921883	[illegible]
NEXN	8.6754018	4.5890748	3.7404852	2.5596504	0.140476	[illegible]
LGI3	0.0194868	0.1500134	0.1551811	0.0379845	0	[illegible]
ADAMTS1	4.4648105	1.519009	10.779302	4.2321365	1.0335232	[illegible]
BDKRB1	0.7614027	0.3682889	1.206423	0.6700441	0.1452492	[illegible]
[illegible]	0	0.005145	0.0053222	0	0	[illegible]
TEF	1.529802	3.4603042	4.3641174	4.9012295	1.7370076	[illegible]
EMP1	30.783899	18.631523	17.572507	15.835621	6.9168365	[illegible]
AXDND1	0.1155705	0.1390131	0.0215703	0.0876068	0	[illegible]
TACR1	0.5551585	0.0717601	0.2556884	0.0502485	0.0167712	[illegible]
PACSIN1	0.0182991	0.0968479	0.2185835	0.1070078	0.0740767	[illegible]
LOC1019292	0	0	0	0	0	[illegible]
KLF9	8.7538672	2.7151844	4.9490949	4.9146981	0.3956517	[illegible]
C9orf135	0.0687753	0.0165452	0	0	0.0349013	[illegible]
[illegible]	1.1882253	2.3876833	1.4958757	1.6273508	0.1945248	[illegible]
HIPK4	0.0538113	0.043151	0.17855	0.0310789	0.1089172	[illegible]
RGMA	0.3213038	0.9742458	3.9579317	1.0061367	1.9035904	[illegible]
PNOC	0.351689	0.0696749	0.2471145	0.0179223	0.2931098	[illegible]
LINC00977	0	0	0	0.0163812	0	[illegible]
CCR2	0.5066214	0.2040733	0.9499643	0.3266239	0.2146249	[illegible]
TLR5	1.6745007	0.4878043	0.8334173	1.1588348	0.2714074	[illegible]
CCDC26	0	0	0	0.0093988	0	[illegible]
FOSB	2.811731	3.8908068	43.859296	4.2267734	1.186314	[illegible]
HOXB13	2.3722522	6.7750224	11.074548	11.330905	1.6818337	[illegible]
OSTN	0.0409896	0	0	0	0	[illegible]
PDLIM3	3.7001258	2.8015406	9.5321051	2.5389273	2.8707209	[illegible]
AQP12A	0	0.1214768	0.0251323	0.1640473	0	[illegible]
SEMA3G	0.3861903	1.005065	2.2977968	1.3306676	0.7372577	[illegible]
FGFR2	5.3170634	1.9109023	3.2458125	0.669317	0.7432344	[illegible]
CYS1	0.0926909	1.345342	2.3220403	1.4186469	0.5628344	[illegible]
PIGR	127.41257	1.2282618	595.62828	445.81745	1097.7064	[illegible]
GNG3	2.8922278	0.4798473	1.0920295	0.3456029	0.3027947	[illegible]
ABCB1	0.9923557	11.101827	8.9807994	16.436144	1.4872717	[illegible]
BFSP2-AS1	0.2951799	0	0	0	0	[illegible]
AKAP6	0.4276133	0.3032529	0.3724487	0.0742837	0.0495867	[illegible]
PDE7B	0.2440968	0.113885	0.5743147	0.2338966	0.1122876	[illegible]
C10orf142	0	0	0.0510857	0	0	[illegible]
RPL10L	0.0541648	0	0.3235009	0.2346226	0.6577954	[illegible]
BEND4	0.0501789	0.0096572	0.0249746	0.015215	0.0050782	[illegible]
SLC1A1	11.760018	0.4669525	5.2343815	7.2002505	5.9563005	[illegible]
APPL2	27.656008	15.544691	8.9115952	13.68991	2.0413823	[illegible]
SCN3A	0.2421758	0.0237355	0.1026767	0.0233115	0.0045387	[illegible]
FAM95B1	0	0	0.0165772	0	0	[illegible]
LINC00964	0	0.025202	0	0.0302523	0	[illegible]
PCDH8	0	0	0.0935117	0.0073986	0.0086429	[illegible]
LOC1019293	3.031256	0.5090811	0.5408509	0.2972904	0.1447036	[illegible]
LINC01266	0	0.0103859	0.032231	0.0187007	0	[illegible]
LOR	0	0	0	0	0	[illegible]
SLC22A3	1.5877909	0.3467133	0.2917886	0.3962119	0.1606889	[illegible]
[illegible]	0.3938869	0.3340576	0.3787926	0.30075	0.1959341	[illegible]
SYPL2	0.2441584	0.2296075	0.2761825	0.2019069	0.0224632	[illegible]
CSMD1	0.0211621	0.0022626	0.0210652	0.0050926	0.0047592	[illegible]
AOC1	12.789689	58.719187	47.982187	86.318992	60.166431	[illegible]
[illegible]	0	0	0	0	0	[illegible]
WDR64	0	0	0	0	0	[illegible]
OR13C7	0	0	0	0.0198659	0	[illegible]
[illegible]	0	0	0	0	0	[illegible]
LOC154449	0	0	0	0	0	[illegible]
LRRTM1	0	0.0167613	0.8853304	0.0528785	0.0441225	[illegible]
KIAA1683	0.8552285	0.1271433	0.5691363	0.3496426	0.3355087	[illegible]
SCUBE1	0.0761119	0.4045664	0.2092515	0.3626587	0.25309	[illegible]
LRP1B	0.0154847	0.0049669	0.0128449	0	0.0078355	[illegible]
LINC00582	2.5048266	0.035446	0.5866726	0.0319118	0.0745575	[illegible]
MAOA	12.125231	17.58551	28.717064	20.502266	30.782357	[illegible]
[illegible]	0.794852	17.830914	12.494572	4.5906886	0.904964	[illegible]
[illegible]	1.8443984	3.4875748	4.416562	2.6426077	0.5743332	[illegible]
AGTR2	0	0	0.060764	0	0	[illegible]
MAG	0	0	0	0	0	[illegible]
LOC389332	0.2902219	1.155174	4.8849217	1.3371379	3.2842417	[illegible]
MS4A8	5.108224	0.8520215	5.1865347	5.0597102	1.6715252	[illegible]
FUCA1	20.905862	29.898617	50.047027	57.089925	18.765432	[illegible]
KBTBD13	0	0.0153677	0	0	0	[illegible]
SYN2	0.2244365	0.1226791	0.3466691	0.0335234	0.0156646	[illegible]
[illegible]	0.3031769	0.0508847	0.1122936	0.0427571	0.0285417	[illegible]
LINC00890	0.036975	0.01779	0.1012158	0.0480488	0.0093549	[illegible]
[illegible]	4.4548793	1.2081016	3.265393	2.5963176	1.803385	[illegible]
SLC36A1	4.7384413	6.9062119	4.5160354	4.2224141	2.2191641	[illegible]
SGK2	4.8667571	7.8326671	3.6533758	6.9210351	3.2930978	[illegible]
ADGRG7	0.1935157	0.7655505	0.2675413	0.6705929	0.5440091	[illegible]
PPP1R14D	3.1235649	40.892385	46.137478	40.154065	17.182299	[illegible]
BHMT2	0.4975943	0.4721715	0.9493562	0.2335019	0.1678598	[illegible]
SORBS1	8.4841515	13.797167	14.163194	3.2119121	1.1643001	[illegible]
PPARGC1A	4.3172576	1.0259417	0.6323396	2.5255756	0.2252157	[illegible]
ADRA2A	1.1744141	2.5088387	4.3605899	0.9309475	24.032278	[illegible]
CLIC5	28.490161	9.4857593	9.1288355	5.566969	1.187351	[illegible]
PTH1R	0.2073109	0.1150903	0.8730693	0.0483537	0.4034701	[illegible]
SSTR4	0	0	0	0.0133506	0	[illegible]
RGS22	0.0622091	0.0033257	0.0412828	0.0149704	0	[illegible]
CD27	1.4036747	0.7203834	3.1360467	1.1619977	2.9673881	[illegible]
IL1RAPL1	0.0719441	0	0.0119358	0.0103879	0.0121349	[illegible]
FBXO32	24.479936	10.853911	10.514413	4.0318289	1.4867036	[illegible]
ANK3	1.6913439	1.0640942	0.5623397	1.1201431	0.3336487	[illegible]
FENDRR	1.4757693	2.3201091	3.1893046	2.6987788	0.8515683	[illegible]
FNDC9	0.5004495	0.1574361	0.1724394	0.0416879	0.0389591	[illegible]
CDH12	0	0.0035625	0	0	0	[illegible]
ABCA6	0.3698562	0.0508433	0.2475045	0.1211662	0.0094363	[illegible]
KLK13	0.109571	0.0105437	0.4144637	0.0094925	0.5766225	[illegible]
[illegible]	0.0638713	0.0983387	0.8646735	0.0830004	0.0775675	[illegible]
[illegible]	2.043905	0.5515353	1.8730758	1.0024565	2.0903779	[illegible]
[illegible]	9.4830665	25.743582	83.898975	41.156953	27.757213	[illegible]
PLPP1	5.1268477	15.220155	19.740294	11.793698	8.544488	[illegible]
GPX3	8.0093335	6.3423579	91.228889	5.9870533	10.56833	[illegible]
IGLL5	2.864511	0.3081737	26.052204	5.6105941	11.037675	[illegible]
LOC1027248	0	0.060692	0.1674205	0.0546406	0	[illegible]
LOC151484	0.1359261	0	0	0.0168224	0.0393032	[illegible]
SGCA	0.8968558	2.0022077	7.1777091	0.3418678	2.0149678	[illegible]
LINC00640	0	0.0134957	0.0558424	0	0.056774	[illegible]
ASAP3	3.2095787	2.7703098	3.82684	2.0433994	1.2046834	[illegible]
CDC14A	1.1279468	1.9716676	0.759292	0.5659167	0.1806712	[illegible]
XKR7	0	0.0053955	0.0167442	0.0072863	0.0056745	[illegible]
CKM	0	0.1021666	0.1321076	0.04599	0.4297967	[illegible]
ZNF835	0.0253278	0.1218616	0.3403606	0.0383989	0.2691412	[illegible]
LOC1019291	0	0	0	0	0	[illegible]
FOLR2	0.0931818	1.5841059	27.455539	2.7446871	5.8153262	[illegible]
OR2T8	0	0	0	0	0	[illegible]
AQP7	1.2056692	0.2578186	1.2668245	0.4352109	0.2457289	[illegible]
SLC6A1	0.0332754	0	0.0082808	0	0.0084189	[illegible]
PEX26	7.9494184	4.3409886	4.4031627	3.1373675	1.8230673	[illegible]
PTCHD1	0.3779571	0.0994487	0.1087531	0.0076743	0.0029883	[illegible]
FNBP1	6.5173044	7.5859924	11.290051	4.5145945	1.9422904	[illegible]
[illegible]	0.1075859	0.0851594	0.221096	0.0225495	0.028098	[illegible]
GPR142	0.1914631	0.03948	0	0	0.0830426	[illegible]
MPEG1	8.9508228	3.4252473	9.4322141	4.8764957	1.4229033	[illegible]
[illegible]	13.366187	5.9485392	13.457246	10.000491	2.1929631	[illegible]
FAM189A1	0	0.2862209	0.4974154	0.8039707	3.3955954	[illegible]
LIMS2	1.6320261	3.7765714	10.092867	1.6764477	38.098201	[illegible]
MCOLN2	1.7633613	3.5043348	3.3579431	2.1475674	0.6983104	[illegible]
UCN3	0.219909	0.1763439	1.9336367	0.4286556	0.4080159	[illegible]
[illegible]	1.070117	1.1327197	3.7282625	1.2515491	1.6244825	[illegible]
GLIPR2	7.0399687	6.4903778	17.139582	3.9225218	11.277046	[illegible]
SEMA3D	0.3109604	0.0367474	0.1629141	0.1394232	0.0276953	[illegible]
CCNA1	0.0183258	0.0176344	0.0729674	0.0317523	0	[illegible]
SLC22A5	4.7703135	2.0783975	1.9685456	1.8935155	1.2460802	[illegible]
HAGLR	7.2532358	0.1526787	1.3988807	2.1914363	1.0460181	[illegible]
[illegible]	0.0075557	0	0.0075211	0.0032729	0	[illegible]
[illegible]	0.0548981	0.0487634	0.0714611	0.0914612	0.0128212	[illegible]
LOC1019294	0	0	0	0	0	[illegible]
CASP7	24.459271	9.5909469	15.946112	16.497137	7.8726622	[illegible]
[illegible]	1.8137948	0.4337288	1.2919532	1.3290551	0.2967754	[illegible]
KCNIP3	0.2682499	0.1693977	0.2781477	0.1936606	0.0678691	[illegible]
LINC00689	0.0048026	0.0069321	0.0382446	0.0166424	0	[illegible]
ARHGEF26	1.931065	2.0124211	1.2283093	0.5657445	0.3324729	[illegible]
LINC00675	5.3642861	10.863124	9.0589411	9.0401467	19.014331	[illegible]
SLC5A1	0.1277564	3.6136877	0.7831086	1.5087301	2.5246162	[illegible]
RGS2	9.8833167	7.3499046	38.28701	8.098645	3.1217036	[illegible]
ARL5C	0	0.0061911	0	0.0055738	0	[illegible]
LY6H	0.0233821	0.0675	1.1404797	0.1316681	0.2129704	[illegible]
VIT	0.2015339	0.0285192	0.6018337	0.0513514	0.0239951	[illegible]
MEIS2	2.4235207	0.4960153	1.0217331	0.372276	0.1978625	[illegible]
PRKAA2	0.0705289	1.1220854	0.3276279	0.3829009	0.0047585	[illegible]
CCDC158	0.2476791	0.0144445	0.0448264	0.0520174	0	[illegible]
LINC01133	16.745049	9.3771477	9.1295735	7.7048145	2.1798348	[illegible]
SCN4B	0.6695761	0.198539	1.4415232	0.5328579	0.3309355	[illegible]
BMP6	0.9334269	0.4262701	1.7008234	0.4660044	0.3042106	[illegible]
MIXL1	0.0216766	0.0417176	0.1078868	0.0281686	0.0438746	[illegible]
ANKRD33B	0.3191974	0.1038271	0.2192827	0.0993171	0.0636974	[illegible]
B4GALT1-AS	0.7304215	0.8032745	1.7657597	1.0395759	0.7920075	[illegible]
PCDH11X	0	0.0036344	0	0	0.0076446	[illegible]
MIR4436A	0	0	0	0	0	[illegible]
NEFH	0.406916	0.3188454	0.6596579	0.1309372	0.1411921	[illegible]
NRG4	0.3759129	0.0168247	0.0174043	0.0492283	0.0265419	[illegible]
C10orf107	0	0.0879103	0.4936868	0.0678387	0.0264159	[illegible]
NR4A3	1.3025971	0.3820691	3.1479789	0.4857885	0.3313286	[illegible]
CLDN5	0.2565285	1.2897941	13.763584	1.1723058	3.115368	[illegible]
VPREB3	0.216304	0.7285025	2.7990782	0.6558664	1.9701511	[illegible]
CLECL1	0.3577835	0.135255	0.3307065	0.0442797	0	[illegible]
[illegible]	3.822419	8.0797552	20.537013	14.520599	5.2603698	[illegible]
RASD2	1.0054458	1.9598357	4.5679489	1.7867628	5.5834207	[illegible]
GPER1	0.963717	0.3897597	2.3356991	1.0829448	1.5407048	[illegible]
CLMP	5.6864846	2.4536604	5.2911046	2.7323019	0.5501632	[illegible]
[illegible]	0	0.0038736	0.024042	0.0034873	0.0244431	[illegible]
[illegible]	8.2116459	6.7081654	3.6406416	11.352107	1.7799613	[illegible]
APOBR	3.2823976	6.5291874	62.599584	28.180133	32.631878	[illegible]
SORBS2	4.7767451	1.5191081	1.7219091	0.9519985	0.5666949	[illegible]
GPA33	22.906277	40.490383	107.81101	94.787325	225.35549	[illegible]
TPSG1	0.1752263	0.7419088	7.8839703	4.3112236	4.9298838	[illegible]
SNCA	0.5916835	0.4217492	1.9414352	0.3037586	0.2661335	[illegible]
LINC02206	0.1223416	0	0	0.026497	0	[illegible]
TMEM108	0.1983749	0.0226055	0.1247158	0.0655774	0.0528318	[illegible]
CHRNA4	0	0.001987	0	0	0.0125383	[illegible]
LOC1002407	0.3164159	0.0608957	0.4094575	0	0.0320222	[illegible]
RAB6C-AS1	0	0	0.0332594	0	0	[illegible]
LRRN4CL	0.2381994	0.9495964	4.5220044	0.9654653	0.3443777	[illegible]
TDRD10	0.0162534	0.062561	0.3721177	0.0774445	0.0822447	[illegible]
SPINK5	2.3391375	1.1399357	0.6995279	2.269986	0.1219196	[illegible]
FGF10	0.1314634	0.3935672	0.9160308	0.3290171	0.0591309	[illegible]
[illegible]	0.1770604	0.1277854	0.2291248	0.2607674	0.035838	[illegible]
[illegible]	0	0.0459314	0.1900547	0.029537	0.0276036	[illegible]
STK32A	0.0395927	0.0435417	0.2364685	0.0171501	0	[illegible]
CD79B	0.2537412	0.3886	4.7148182	0.7785411	1.4765609	[illegible]
[illegible]	0.0044688	0.0129006	0.0355866	0.0425858	0.0045225	[illegible]
KNG1	0.0352795	0.5262025	0.140472	0.8048435	0.0129013	[illegible]
PRRG3	0.0276441	0.0232761	0.1651054	0.1915913	0.0069942	[illegible]
SLC20A1	20.945972	7.4407814	8.4624942	9.3360877	3.7750795	[illegible]
[illegible]	0.1744756	0.819319	1.7715836	0.9915654	0.3743373	[illegible]
SPIC	0	0	0	0	0	[illegible]
[illegible]	36.814249	14.470693	10.767607	8.0993019	2.2357033	[illegible]
ST8SIA6	0	0.1047189	0.2999803	0.0797735	0.5591386	[illegible]
PLCG2	1.4752396	0.2681436	1.1813152	0.4444749	0.577287	[illegible]
C16orf96	0.0115045	0	0.0114519	0	0	[illegible]
ARMCX1	0.9449618	1.8680424	4.9894586	1.6372956	0.623692	[illegible]
MS4A2	0.6716288	0.2185983	0.4030987	0.2395859	0.0099957	[illegible]
FGF10-AS1	0	0.0111728	0.0462307	0	0	[illegible]
MOCS1	2.3206482	2.4294145	3.1561866	2.8683819	0.7398397	[illegible]
NRAP	0.2151451	0	0.1147267	0.0033283	0.0155523	[illegible]
LAMC3	0.6320944	0.2671358	0.3188516	0.2146007	0.0172891	[illegible]
TMEM156	0.5493434	0.1156353	0.2734142	0.1115419	0.0173735	[illegible]
ACADL	0.0401385	0	0.099887	0.0043466	0.0101553	[illegible]
[illegible]	0	0.0187351	0.0193805	0	0.0098519	[illegible]
ENTPD8	1.7989738	0.4999311	67.600356	5.1860537	7.2824474	[illegible]
GZMA	4.6244855	1.0178236	4.4074283	3.0473648	1.4439833	[illegible]
LINC01798	0.4379023	0.0790091	0.0544872	0.0355657	0	[illegible]
ALAS2	0.5152588	0.0198325	0.061547	0.0178551	0	[illegible]
SYP	0.6662633	2.0775776	1.3767967	0.3543589	0.4694334	[illegible]
MAPK10	0.1254575	0.1573729	0.4995325	0.2057298	0.0861558	[illegible]
MIR9-3	0.0302697	0.0291277	0.0361573	0.0268458	0.024507	[illegible]
FAM133A	0	0.0187766	0.012949	0.0056348	0	[illegible]
NBEA	1.877205	0.3258576	0.447001	0.0701531	0.1043019	[illegible]
HHIP	0.5682592	0.3838261	0.326341	1.1960352	0.081103	[illegible]
[illegible]	4.0232839	8.2533328	21.644474	8.0483075	7.7917071	[illegible]
TINAG	1.0204596	4.5170215	1.665892	4.1727336	1.2599351	[illegible]
MIR7704	0.3340391	0.0214291	0.4433464	0.2122177	0.360594	[illegible]
TLR7	0.0928777	0.3981194	0.8656915	0.2962488	0.0683599	[illegible]
EPB41L4A	1.9289588	0.0996309	0.3162885	0.3507474	0.0363057	[illegible]
[illegible]	0	0	0.3908969	0.1701014	0	[illegible]
DSC2	15.852792	10.542144	4.6183407	7.8509273	3.4236952	[illegible]
IQGAP2	13.160043	1.4269931	4.1099866	10.571768	0.5011973	[illegible]
CNGA1	0.4912796	0.242282	0.5257078	0.5107317	0.5966265	[illegible]
[illegible]	0	0.0102774	0.021263	0.0092527	0.0236177	[illegible]
GIMAP7	2.5562675	2.6620061	6.0302696	3.2915164	1.0631566	[illegible]
TPPP2	0	0.0342	0	0.0076975	0.0179842	[illegible]
[illegible]	0.1454885	0.7304334	1.6371233	0.2219416	0.4417153	[illegible]
IGSF9	4.6118523	3.5375196	3.8377254	2.6386763	2.6660827	[illegible]
SVIL	12.592155	15.449831	15.200017	7.9219292	4.2060643	[illegible]
STYK1	10.224743	1.6501336	4.5007944	3.7891281	0.2599931	[illegible]
CHRNA3	1.0430104	1.4477591	2.8298804	0.2718767	0.7566373	[illegible]
LINC00483	1.6569723	2.5245624	3.0955877	2.6577242	1.2880554	[illegible]
ADAMTS8	0.0194481	0.4163945	3.4265489	0.593907	0.6101417	[illegible]
GRIK2	0.1908363	0.0344319	0.053427	0.0335821	0.0603537	[illegible]
COL2A1	0.0400077	0.0866213	0.0066374	0.0462131	0.0134963	[illegible]
GNAI1	6.8699557	30.857069	21.981407	27.743907	23.852723	[illegible]
CACNA2D1	0.6687578	0.7985383	0.91351	0.5687919	0.0197606	[illegible]
NXPH2	0	0.0201153	0	0.0181097	0	[illegible]
LINC02106	0	0	0.072245	0.0314379	0	[illegible]
APLP1	0.3609011	0.3299212	0.9819467	0.2553382	0.3774167	[illegible]
HS6ST3	0	0.0108436	0.0224343	0.0048812	0.0741277	[illegible]
ANKRD35	0.0523126	0.2894493	0.950334	0.1926096	0.3176508	[illegible]
ZDHHC15	0.1741215	0.0868791	0.1283884	0.0670429	0.013053	[illegible]
COLEC12	0.7849614	0.6371443	3.1731883	1.7831448	0.2001168	[illegible]
RAB6B	0.4174793	0.8386979	1.5820747	1.4498702	0.4225007	[illegible]
ATP2B2	0.082209	0.0041636	0.0559907	0.0056226	0.026273	[illegible]
CAPN13	0.1186179	8.0226097	0.8265237	0.9432092	1.1661475	[illegible]
DDX25	0	0.0068765	0.0284535	0.0041272	0	[illegible]
CLIP3	7.9988125	4.6395454	9.2621428	3.8638093	3.2922903	[illegible]
KCNJ16	0.0069372	0	0.0552435	0.165272	0	[illegible]
MGP	8.1163853	21.281544	106.32396	12.741814	7.1345499	[illegible]
CD19	0.2271285	0.1170856	1.0496974	0.24596	0.082093	[illegible]
LOC1019276	0	0.166101	0.1374583	0.2990794	0	[illegible]
PRDM8	0.3339795	0.259309	0.5337938	0.2791482	0.0904498	[illegible]
C1QTNF12	0.0735482	0.7077346	2.0255171	4.4070867	1.6871437	[illegible]
CBX7	3.2657716	4.0274755	4.7060615	2.1270314	1.330373	[illegible]
GRAMD3	7.0531113	4.0847817	4.7238859	4.7486952	0.8019174	[illegible]
BFSP2	0.2974615	0.0102228	0.0423	0.0460178	0.0215028	[illegible]
FLNA	53.580106	134.23058	281.99411	49.164562	162.20273	[illegible]
TPSB2	11.058717	2.1550884	10.202005	3.8913878	8.5826424	[illegible]
MIR22	7.6445273	1.4953204	7.7854727	4.6958471	2.7577032	[illegible]
HRK	0	0.0092637	0.0191655	0.00834	0	[illegible]
SMIM2-IT1	0.5520729	0.0442704	0	0.0797128	0	[illegible]
DUPD1	0	0	0	0	0	[illegible]
NTRK2	0.1490323	0.0415681	0.1505	0.1309822	0.0721336	[illegible]
PID1	7.5438477	18.827486	3.3707306	2.0671063	3.9165268	[illegible]
OR6M1	0	0	0	0	0	[illegible]
SLC41A2	11.407435	3.7795307	2.6263173	4.2274708	0.820914	[illegible]
GSN	23.95709	28.097361	66.516766	30.918521	45.780164	[illegible]
ADAM33	0.5955713	0.6492743	4.8027924	0.5355541	0.8850283	[illegible]
PAG1	12.680231	4.9797984	1.5370621	2.0739934	0.4118755	[illegible]
TLCD2	7.8962483	1.7684593	2.1629835	2.6705722	0.730541	[illegible]
AMHR2	0	0.0060151	0	0.0054154	0.0126523	[illegible]
RAB39B	0.2007729	0.0645243	0.0624543	0.0543548	0.0761954	[illegible]
SUSD5	0.0614762	0.1985981	0.2972315	0.0380418	0.0711035	[illegible]
EIF4E3	5.8511841	1.9690832	1.9008491	1.5894034	0.3014463	[illegible]
OR51E2	0.214396	0.405247	1.3719502	0.4776106	0.4804441	[illegible]
NKX3-2	0.172724	0.6497211	2.7040409	0.5305269	0.4608403	[illegible]
TTC22	6.4065987	3.4249444	4.2732037	6.0001551	4.7782054	[illegible]
PCP4	0.0397666	6.8114013	14.408757	0.3617346	0.4024487	[illegible]
[illegible]	0.0644895	0.2004902	0.1036983	0.0193393	0.0150612	[illegible]
[illegible]	0.1204981	0.029988	0	0	0	[illegible]
[illegible]	0.5638693	0	0	0	0	[illegible]
ACOX1	10.746263	15.72108	10.712569	13.249599	4.628663	[illegible]
[illegible]	0	0	0	0	0	[illegible]
SETBP1	1.4987972	0.4535505	0.7616723	0.6065135	0.1197493	[illegible]
KCTD4	1.2970507	0.0340396	0.3051721	0.0408608	0.0239864	[illegible]

DAAM2	1.1460673	1.446754	2.8814537	1.4023026	0.8549426	0.7866225	2.7107085	0.6679611	0.8278326	0.6672908	0.8775849	1.5930662	0.6060421	1.2819688	0.5354681	0.6954554	2.3764186	0.8982584	0.8634496	1.2469727	1.7572782	1.9839038	3.1026985	0.3079991
SH3RF4	7.3750183	6.9613977	7.4063944	8.9941911	2.695418	15.76579	9.6513275	6.4301422	16.173741	3.9394609	9.3522217	7.1948881	25.058985	13.290706	12.448912	7.7172286	8.7636497	8.1998416	10.569776	14.96201	5.0393931	16.845781	7.8077736	13.149544
FER1L6	27.465035	0.031436	0.5203355	1.0598065	0.1028642	0	0.5876581	0.5461172	4.1457747	0.3075265	1.1060642	0.9901536	0.4301848	0.7416522	0.4975616	0.0749721	0.1655721	0.3384273	0.9955416	0.3298593	0.5267349	1.7433675	0.0119436	0.7412615
ANKRD7	0.0539323	0	0	0	0	0	0.0708679	0.0127575	0	0.0346134	0	0	0	0.0284067	0	0	0.0170628	0	0.0573291	0.0255248	0	0	0.0554536	0
SV2B	0.1836401	0.1138433	0.358568	0.0489516	0.0393142	0.0543392	0.0983765	0.1052567	0.0350539	0	0.011488	0.154813	0.0187392	0.0595232	0.0351332	0.0437624	0.2639898	0.0391955	0.0244008	0.106969	0.0329425	0.1490343	0.3544095	0.0153435
SLC2A13	2.3817814	1.601815	0.7466299	1.7879054	0.0976601	3.4316973	1.3717635	1.624833	0.9757759	0.636217	0.8156923	1.3421104	1.8426043	1.2290953	1.7018471	0.5073132	1.9089724	0.9054977	0.834605	1.2829338	0.6501122	2.5132916	1.2934854	1.7485092
EPB41L4A-A	0	0.181902	0.2822522	0.2865892	0.0637691	0.0372901	0.4802262	0.1192402	0.0551864	0.1617605	0	0.1255562	0.111451	0.3650751	0.1393025	0.1952068	0.2195438	0.1049014	0.1339595	0.7008083	0.5224658	0.2247153	0.4016887	0.2053235
PTPRH	12.265639	12.374936	1.1494285	0.7076642	0.2683461	1.0022629	1.2677987	3.9373022	2.2174151	1.5480497	1.4654071	1.3900013	1.2783922	1.4686911	5.304143	2.9501463	1.3058668	1.8891499	0.872847	3.0927732	0.6205679	2.4657357	0.2251445	8.8817528
PXMP2	3.3423676	5.786283	7.6491419	12.340084	3.9199871	3.0502105	8.3241249	8.2017836	20.719143	9.7431967	13.548481	11.881513	25.111387	12.633822	15.365326	10.838415	14.683013	13.624962	11.007276	4.4055526	7.9968255	7.7423047	4.5093904	7.9393704
CACNG6	0	0	0	0.0102814	0	0	0	0	0.0138586	0	0.025737	0.0315302	0	0.0250034	0.0262367	0	0.0451066	0.0263433	0.0252304	0.0112334	0	0.0141079	0.1464297	0
SYNC	1.4126649	0.7464398	2.5612884	0.7539683	0.3574141	0.1791463	1.4651214	0.4654367	0.0810097	0.0485699	0.6017748	0.8042513	0.3569501	0.8104996	0.3067292	0.4949494	2.5329186	0.2239822	0.3821131	0.9073575	0.2876034	1.3044702	1.2916974	0.2740002
THEMIS	0.5511761	0.0540204	0.1524035	0.075162	0.0309892	0.1268303	0.5472137	1.0478551	0.131112	0.052406	0.210286	0.4062052	0.1720356	0.3836073	0.0421612	0.6724667	0.0906273	0.1301978	0.048307	0.0211581	0.2366056	0.9067564	0.2882503	[illegible]
CNTN6	0	0.0078333	0	0	0.0046571	0.0124085	0.0037229	0.018379	0.010101	0	0.0104537	0	0	0	0.0086986	0	0.0149555	0	0.0041625	0.0037244	0	0.0046774	0.0609132	0.0085475
LINC01797	0.1724777	0.0553236	0.1144567	0.0498075	0	0.3062172	0	0	0	0	0	0	0	0	0	0	0.5098947	0	0.0305568	0	0	0	0.0945827	0
OR5A2	0	0	0	0.0413669	0	0.0386755	0	0	0	0	0	0	0.0430589	0	0	0	0.0258941	0	0	0	0	0	0	0
CPED1	0.9304393	1.4903272	2.6989897	1.4069075	0.4101581	2.9558605	2.6642416	1.336751	0.4732731	0.2837714	0.9346254	0.7936668	1.267099	2.1647352	0.3028677	0.3890455	6.0859862	1.2607409	0.7797035	0.7915562	0.9347765	1.6692805	1.9673642	0.1736034
LINC01140	0.2946526	0.0094512	0.0977679	0.0397081	0	0.0620002	0.1376632	0.1084203	0.0649935	0.0084047	0.0213	0.0347926	0.0289537	0.0344881	0.0289514	0.0676167	0.1037	0.0218017	0.031321	0.0154946	0.0135731	0.1362165	0.4281887	0.0142242
EPCAM	0.0075765	0.0145814	0.0452511	0.0229732	0	0.022419	0.0836277	0.017922	0.0088476	0.0194503	0.0246463	0.0268392	0.0067005	0.0239438	0	0.0782397	0.0047997	0.0252269	0.0080537	0.0107574	0.0157055	0.02702	0.0218127	0.0246883
GPIHBP1	0.4047406	0.0834581	2.609177	0.2504561	0.1950518	0	0.4558586	0.2005973	0.1237865	0.024739	0.0522466	0.2048221	0.0340898	0.1015148	0.1491306	0.517472	0.2930283	0.1069547	0.1024362	0.0091216	0.2197356	0.3665821	1.4109699	0.0418685
SHE	0.7346801	0.2933145	0.798743	0.5123396	0.0738244	0.2960233	1.3337313	0.3130602	0.2616379	0.0802574	0.3644174	0.786759	0.3133462	0.4775296	0.1843064	0.0430453	0.9473314	0.4395063	0.2990879	0.3501719	0.194416	0.6534696	1.5193776	0.1697854
C4orf19	26.690765	3.3078799	5.2473325	7.6136654	0.9480644	2.3585358	5.0907685	3.9135955	6.3041314	3.5054722	11.681628	6.2503338	7.5124828	8.1790718	12.022513	6.0010946	1.9614117	5.445329	8.3967708	6.4174854	6.7801729	7.2574396	1.8154254	4.1218387
ELOVL4	0.3903711	0.1739091	0.21588	0.2442482	0.0438963	0.1454582	0.5395509	0.2599216	0.1181857	0.0371166	0.1332579	0.3137026	0.0767188	0.3503026	0.0479453	0.298607	0.2472968	0.2567475	0.1844255	0.2258091	0.1048968	0.3351523	0.3627304	0.1099289
ARHGEF37	3.7174707	1.4030665	2.8205665	1.5172381	2.2155086	1.3297787	2.4228662	0.7034811	1.2782151	0.7210535	2.3469105	2.2972212	4.7780334	2.4192552	2.9403719	0.6483426	2.7736505	2.6405832	3.3281289	4.5665854	1.8922458	2.7889912	1.9842125	1.1216176
ROBO1	1.1799614	0.3119829	0.6128612	0.2808763	0.0132571	0.3139696	0.6231116	0.2169052	0.6998439	0.0504433	0.1811037	0.6438552	0.5676616	0.7382657	1.0497996	0.2570199	0.6472805	0.210813	0.3237471	0.1487926	0.0678855	0.5722795	1.1179322	0.4553096
SGCE	0.7943318	2.1933936	6.7898661	2.6329321	0.7514587	1.1752153	5.9902959	2.6060318	1.5022888	0.5319614	1.3013351	2.8903077	2.0463755	2.9832513	1.0116525	2.924449	4.5616402	2.0698565	1.6336539	2.3455232	1.8792462	3.3638864	7.5707976	0.8065149
SLIT2	0.9555356	0.6558423	1.798841	0.5727962	0.3345644	0.9808914	1.430537	0.3299362	0.0951897	0.0697542	0.7144759	0.6978335	1.0332859	0.2480673	0.085099	1.0381819	1.3368761	0.3267007	0.1708908	0.7715794	0.2252972	1.2381864	3.2333627	0.1278983
ZNF804B	0	0	0	0	0.0095007	0	0	0	0	0	0	0	0	0	0	0	0	0	0	0	0	0	0	0
ENPP1	1.8559963	3.1433177	0.5331355	1.0244641	0.1985653	2.6674938	0.8696086	0.5443944	0.5573199	0.6874723	0.4987516	0.2888167	0.3892454	0.4859932	0.6916755	0.6790266	0.5610045	1.2006365	0.6087777	0.9160425	0.3957234	2.8083455	1.1253796	0.5126239
ANKRD20A4	0.0097521	0.0703811	0	0.0211212	0	0.0403989	0.0358932	0	0.005694	0.0125176	0	0.0129547	0	0	0	0	0.0926678	0	0	0.0046154	0	0	0.0040109	0.0105924
FASC	0.3959947	0.598901	0.4242147	0.0343062	0.0381674	0.7454559	0.1288468	0.0285473	0.0308264	0.0484089	0.1778892	0.0417492	0.0333531	0.0437013	0.0875449	0.2180942	0.1959092	0.0209267	0.0601336	0.1874144	0.0820861	0.0717321	0.0418798	0.1597587
GIMAP1	0.3358083	0.4672421	1.2919119	0.5071451	0.5327337	0.1879893	1.3834003	1.0605509	0.2755599	0.2795926	0.4526971	0.9645151	0.5056693	0.8031022	0.4614863	0.224835	0.7531853	0.6446767	0.400372	0.4553122	0.4138973	0.7228668	2.3815003	0.4731847
NUDT32	0	0	0	0.0212964	0	0	0	0.0232485	0	0	0	0	0	0	0	0	0	0	0	0	0	0	0	0
NGFR	0.2649507	0.7291713	4.9899069	0.3443016	1.2119814	0.1818856	0.7185796	0.6116876	0.1546992	0.1632417	0.1493924	1.1356804	0.0374905	0.3967463	0.0351445	0.4596533	1.2689007	0.3881604	0.1126551	0.5868465	0.2526406	1.2724483	8.368864	0.2532488
SLC22A17	0.8635979	1.9093613	5.3420751	1.0598932	1.1757183	0.0790954	2.1344678	0.8803508	0.3841805	0.7521976	0.7469083	1.8983509	0.4637019	1.6461816	0.4091157	2.1870343	1.1006769	1.0839974	0.8469542	1.2942782	1.1508171	1.7049054	3.1247627	0.591845
LOC1001282	0.3163374	0.1522015	0.1194407	0.0094501	0.0662363	0	0.0459673	0.036124	0.0063691	0.0140036	0.0650542	0.0289809	0.0096469	0.0344727	0.0602884	0.0901153	0.1174742	0.0242134	0.0173928	0.1290643	0.0226117	0.0064836	0.0607542	0.0118482
MXI1	17.031375	13.724735	7.868508	7.5676112	2.1931748	14.836281	9.6743568	12.605486	8.4894158	4.4972362	9.3683616	8.8688742	5.4963231	5.7826188	11.650251	6.4869945	8.4971255	6.6659749	9.0672445	9.0635744	4.6604303	13.204289	10.569884	11.136762
SLAMF7	3.3398146	0.753101	2.8806506	0.9775992	0.8288793	0.4254601	6.7538465	15.804675	3.2199709	0.7008617	2.437325	3.4695055	3.0663213	4.5439897	5.2710717	1.7103451	3.2399137	1.919032	2.0991425	0.357477	2.3202866	3.2075598	10.796749	1.1762592
ACACB	1.8223417	1.5522637	2.819093	1.6010758	0.7855753	1.1735688	1.6160452	0.7029045	0.086751	2.6777409	1.9431352	1.3064005	0.4746407	1.2169764	1.72278	0.5135175	2.0169052	1.4235347	1.0523602	0.9471374	1.8007781	2.0527621	3.7262985	0.5236634
EGGNTN	1.6663332	9.1691281	13.877049	10.379635	16.507482	1.1805867	10.102936	19.7415	6.0705809	22.714313	8.7034171	10.776814	12.453492	17.776008	6.8488677	8.1432702	7.2108435	13.102188	12.660925	19.882465	14.83826	25.193298	8.3108767	4.792489
PDE5A	4.8908671	2.5285275	3.6412797	1.5708843	0.9744568	8.6752669	3.5048119	3.7314973	1.8264731	0.4042771	1.5685468	2.6079791	4.094568	1.592566	2.5265703	1.1615898	7.8697976	1.8926393	1.781593	1.6545925	0.8394195	2.8241256	2.3057629	1.1729172
NIM1K	0.1336833	0.0292363	0.1209739	0.0579069	0.0369976	0.0503452	0.1405295	0.0977413	0.0141918	0.0155994	0.0527114	0.0753391	0.0214957	0.0768135	0.0403011	0	0.046193	0.0134983	0.0710515	0.0575173	0.0251921	0.1372467	0.0749749	0.0264006
SPAG6	0	0	0.0112633	0.0147039	0	0.0066963	0.0118946	0.0107062	0	0	0.0122693	0.0050093	0	0	0	0	0.007168	0	0.0060139	0.0107103	0.0234552	0.0201764	0	0.0245804
MEGF10	0.1220767	0.0469885	0.06805	0.0401883	0.0148254	0.0173388	0.0615977	0.1478488	0	0	0.0317691	0.01946	0.0475031	0.0051439	0.0161929	0.0302552	0.0618677	0.0216783	0.0493108	0.0231104	0.0660384	0.0754623	0.0341415	0.0530387
UBE2QL1	0.3362056	0.3333254	1.0547063	0.1632847	0.4227342	0.1085273	0.6747172	0.2699131	0.0178458	0.1961585	0.1712312	0.4060146	0.162181	0.1502522	0.1238783	0.3787474	0.4130588	0.0791519	0.9096959	0.2362658	0.2006295	0.5207788	0.8087022	0.132792
SQRDL	16.891977	27.808422	32.42419	15.436042	23.768894	2.9585462	27.039836	26.979553	22.129097	16.819318	45.088363	25.573772	62.413113	45.656182	31.597631	39.829103	51.591812	26.936266	22.755201	16.764294	28.623572	25.557754	14.480533	40.980526
TSHB	0.6552225	0	0	0.0354774	0	0	0.0430486	0	0	0	0.0888094	0	0	0	0	0	0	0	0	0	0	0	0	0
CCR10	0.1343469	0.0646392	0.6017932	0.0800172	0.2889209	0.0198766	0.3001074	1.0725468	0.0980529	0.1077786	0.0910474	0.1189777	0.0594065	0.3891904	0.3526976	0.4162027	0.1808537	0.1677461	0.0714042	0.1271661	0.3829222	0.0698714	0.4351294	0.1641647
HCN4	0.0060849	0.0058554	0.0423994	0	0.0184743	0	0.0063965	0.0172723	0.0142114	0.015621	0.003299	0.0215553	0.0215254	0.0256399	0.0336307	0	0.015419	0.0270139	0.0032341	0.0057597	0.0063067	0.0180838	0.0173184	0
CCDC80	7.3992806	3.3172236	11.086627	3.2925574	1.8218414	2.0012704	10.118041	2.9799914	0.4597303	0.5657966	2.6432241	4.8229761	2.2169553	2.4908474	0.8071149	6.3795936	8.4453874	2.5581569	2.0548504	2.069688	1.1957308	10.251954	28.18782	0.7382703

[illegible]

[illegible]

[illegible]

[illegible]

FLRT2	0.3087637	0.1485575	0.317386	0.2516598	0.0318855	0.5563837	0.533232	0.1097041	0.0198677	0.0226471	0.1339203	0.1735516	0.1159124	0.2243617	0.0376128	0.1587726	0.4095607	0.158056	0.1111896	0.0906604	0.0587791	0.3573076	0.8096204	[illegible]
VCL	44.259116	21.862262	21.180416	16.403006	9.622035	19.766211	25.354389	12.998383	13.199841	13.932803	22.556648	26.819287	22.706439	23.077574	13.497038	12.963619	40.153554	13.112198	13.108023	13.845055	14.941087	29.979967	23.56886	[illegible]
GSK3B	13.174319	8.2754581	18.338518	9.7238264	5.4578629	7.7851721	13.489613	20.26524	66.581496	6.10652	21.072857	18.70759	18.745564	15.48098	39.089138	16.656535	23.359755	13.146784	18.518531	10.897713	11.788056	17.252383	10.016851	[illegible]
WDFY3-AS2	0.2198027	0.1432812	0.2329123	0.0798542	0.0861083	0.4783564	0.2198789	0.5199336	0.2276968	0.0182023	0.2767795	0.103608	0.1442233	0.0746918	0.1959398	0.0878635	0.1706851	0.1573987	0.0904437	0.2080543	0.0955355	0.1306462	0.2041313	[illegible]
BAD	3.862571	16.639097	19.04501	13.061557	5.9938459	0.8229139	17.378426	23.146414	19.771244	18.179112	22.798255	14.663388	28.147516	38.62754	16.423752	22.70906	15.611261	9.4884579	22.650569	19.999964	8.3270264	14.494358	13.586713	[illegible]
MGST3	7.6330767	9.7909573	11.511847	12.498225	10.066725	3.6290598	10.712398	12.557262	15.238081	11.397057	10.666675	15.239823	21.024709	17.057833	16.984844	14.575319	12.931121	9.4595142	10.988164	12.411002	16.969844	10.153668	8.1498249	[illegible]
PPM1A	7.576293	6.5196676	5.1052587	3.9082592	2.026631	13.558503	4.1770695	6.2071339	5.4737945	2.3276733	5.2375276	4.5026064	4.4274999	4.2854543	6.0258329	5.3000572	8.5183804	4.7044294	6.4530194	3.5944467	3.8551348	6.0152781	5.3183956	[illegible]
SHROOM3	36.357939	9.3849039	9.1160761	6.1863707	4.5698092	7.7952864	14.222662	12.619798	8.4910676	4.3244959	15.225202	11.320432	25.512188	12.442813	13.895203	4.8942089	10.150538	5.6199851	10.461348	12.525048	6.8971821	10.759513	6.5158163	[illegible]
SALL1	0.2020366	0.3701352	0.2707272	0.8919805	0.1966026	0.3816895	1.0088203	0.3970318	0.2858382	0.3790225	0.5982397	0.261504	0.4947953	1.2851628	0.2233271	0.1604881	0.2855127	0.5433373	0.520383	0.1397507	0.9906312	0.378734	0.044743	[illegible]
CYB561D1	2.0406322	2.5215003	2.0159614	3.1841549	1.8304977	0.9880752	2.0638814	3.4922802	2.2611094	4.1274224	3.977015	2.4301177	3.2129842	3.5175997	2.1360538	2.777769	3.2807751	2.9768889	4.0877074	2.3047035	2.0829766	3.8592586	3.44612	[illegible]
THSD4	1.3441432	2.8220363	1.5093849	2.5334444	0.9584006	1.9804444	1.7399494	0.9827582	0.3421414	0.7628492	4.0367066	0.9001593	1.8494029	1.489086	0.598613	1.2159679	1.8402325	1.3099702	1.3476181	1.2841283	2.0609309	2.4331082	1.4859964	[illegible]
UNC5B	6.5384446	4.4636316	9.2539888	8.268306	7.326295	1.7425868	15.625413	3.6346809	3.3259081	4.7286033	5.7269218	9.3945411	3.5934116	5.804117	3.6101775	5.5901268	6.2354747	5.2374741	8.0847042	3.8673435	6.1904104	11.739199	13.009449	[illegible]
SPATA24	6.0639371	1.8103074	1.4545802	2.6741524	0.2218147	1.1025346	1.7856229	1.8664452	1.5570139	1.9693398	2.3171954	2.2483791	1.2276274	2.4627959	1.9382027	1.5899076	1.8975852	1.0135821	2.640471	2.1956546	1.4765952	1.563302	1.8479576	[illegible]
KLC4	4.0755608	7.5700705	12.572988	14.992448	20.525859	1.9457503	7.5172666	2.6766732	4.5113087	18.850374	11.553156	6.3845553	8.8684533	15.89596	9.335656	13.778928	11.229891	15.219726	8.4060167	21.794516	18.756572	11.595091	5.6652983	[illegible]
SLC31A2	1.4578635	0.3464903	1.1189893	0.3880269	0.5155007	0.041579	0.5262278	1.6280949	0.9947948	0.2254569	0.2475952	0.5599889	0.4504775	0.4070646	0.2912335	1.4510582	0.589733	0.214439	0.5414554	0.2576998	0.6007627	0.9709246	0.9391228	[illegible]
CYTL1	0.4385026	0.0843919	1.0912377	1.1016737	0.0887554	0	0.6914386	2.2197334	1.2545595	0.1125711	0.5230281	0.7378429	0.8143814	1.201012	0.2423359	0.8150809	0.4166815	0.3406768	0.3728963	0.1037662	0.454488	0.8601018	3.5348299	[illegible]
CD320	1.0828418	0.9348349	1.2842878	0.755135	0.9404242	0.4044933	0.9929816	1.8529384	0.9012909	1.2420561	0.9493163	1.5982364	0.9100958	1.092148	0.628187	0.7137473	0.5838052	0.6733569	2.2163668	0.6869441	1.0108806	1.2644144	1.9330257	[illegible]
TCP11L1	7.7725766	1.3437599	1.7120848	1.4829549	0.563638	2.3459546	1.472262	1.7397628	1.1572767	0.8200081	1.6962598	1.0952516	1.5283318	1.3200551	1.77446	1.5900571	1.9716201	1.3817031	1.3407426	1.209392	0.9592404	2.4097266	1.8526658	[illegible]
METTL7B	5.4320162	27.821582	24.826367	20.265787	13.122712	5.686165	21.704897	35.079687	45.289676	18.030923	19.206097	21.376131	22.039966	12.582683	10.943574	26.358192	6.4197546	27.259409	27.396099	48.293059	19.281365	16.899809	12.455672	[illegible]
OLFML3	1.9795413	5.1431222	19.256951	8.1927075	10.5267	2.0341393	16.524373	7.2025642	2.6898922	11.272402	6.2930589	7.0282727	9.6445263	21.326827	2.8048029	10.518272	6.2815138	62.275945	9.9950294	6.4132853	7.0690545	9.7229834	22.877638	[illegible]
BCL2L2	21.481685	10.456867	9.1866841	7.9771023	5.5997142	9.189806	9.2821571	7.261979	7.1602567	6.7518878	13.655989	9.8979789	5.5805061	5.5964817	8.0544065	6.8306077	10.322747	9.6602772	12.27669	7.8263089	8.4553965	10.552858	11.987331	[illegible]
EGFR	10.156435	6.9323915	4.6546923	7.3715423	1.3359084	7.5187537	4.681921	3.3279651	1.8624536	3.5891082	7.1677644	5.3732893	4.6155908	4.1573456	2.5094124	2.7052534	5.468806	6.7415528	4.802879	1.7023707	3.351797	8.7112716	4.6642446	[illegible]
OAF	5.8162341	29.976236	31.718774	31.210395	43.922202	4.5156434	27.950142	32.986555	41.722019	27.622843	39.033959	29.108509	30.384756	39.577124	38.498334	31.975729	16.4225	27.726909	23.913228	22.224607	46.087498	36.249638	23.670554	[illegible]
ATG4A	4.1772951	4.4823996	4.9808307	4.3804454	7.7451419	4.5174101	5.9155125	8.1324477	10.773871	4.1853797	6.2403555	4.1452101	4.9168672	6.1732919	2.4748449	6.9205606	10.832619	2.001262	6.0216791	3.1433036	4.3918723	5.9303758	5.0490654	[illegible]
CD4	7.1975467	4.9082311	17.691774	5.1463192	3.8168782	1.7776367	14.537087	18.292177	6.298937	3.3505561	8.9387458	9.5569831	8.1221603	10.604753	8.2636523	7.2145737	8.2680477	6.6604912	5.1617818	2.6479238	9.031488	15.634934	20.090063	[illegible]
[illegible]	0.2663412	0	0.0176748	0	0	0.4413385	0.0186654	0.033601	0.0103674	0	0.0385068	0	0.0628121	0.0748185	0.0588816	0	0	0.019707	0.0566232	0.0756316	0.0184034	0.0211077	0.0438166	[illegible]
FITM1	0.1298356	0.0780858	0.1938618	0.1546605	0.3941918	0.192092	0.2559088	0.3992565	0.2274249	0.4166377	0.0703921	0.2738835	0.0661175	0.2051573	0.179397	0.4692647	0.3701239	0.1441007	0.2587742	0.1382579	0.4037061	0.4244439	0.1735471	[illegible]
RPS6KA1	16.040952	14.889128	25.81142	30.390218	28.254235	3.3530889	20.256925	20.531579	30.947882	14.727654	37.049933	22.142909	33.462544	25.677519	29.464095	15.345211	26.053246	13.929613	22.231769	11.614085	23.513071	22.911912	11.617632	[illegible]
EGLN1	5.8238816	8.8140377	7.0566982	6.707986	3.4003449	12.449953	6.1444065	6.647487	8.423344	5.8902189	9.213191	9.6665461	12.12133	8.252273	11.369522	6.5517123	11.716274	7.1289437	9.4340317	8.8734473	7.9875962	7.4017691	7.6513997	[illegible]
SLC35F3	0.1514241	0.0198697	0.0411084	0.0059629	0	0.0570264	0.0072354	0.0325625	0	0	0.0149267	0.0243821	0.0121741	0	0.0304329	0	0.0610437	0	0.0219492	0.0716652	0	0.0409106	0.0736014	[illegible]
4-Mar	0.2571457	0.052344	0.0787595	0.1028182	0.1000918	0.0526773	0.3663474	0.0187159	0.1443674	0.0634747	0.139415	0.0919673	0.0962133	0.1041856	0.098592	0.0617056	0.0313269	0.1297455	0.0683353	0.0702119	0.1640122	0.0293927	0.0386091	[illegible]
WASHC2A	6.5900248	5.7999664	3.7424269	6.2150485	1.3890758	1.9928752	8.0477428	4.392182	4.126531	2.5825238	7.2612926	6.3117816	4.342766	4.1580566	2.2522511	3.0460689	8.2738408	3.5955347	6.2755708	5.8827282	2.2001746	7.4032352	6.2657405	[illegible]
RCVRN	0	0.0289781	0.1199654	0.0391332	0	0.044554	0.0553987	0.0142468	0.0087915	0	0	0.0066673	0	0.0951688	0	0	0.1049241	0.0501343	0.0400136	0.0285046	0.015606	0.0626474	0.0805054	[illegible]
CALML4	4.3650068	4.0533187	4.1799124	4.4130478	3.7107076	1.0591177	4.1893428	4.8982901	7.1769613	3.9892356	7.3836265	7.0200289	4.7250101	5.9224668	5.6934864	5.7696699	4.6123453	4.0694578	4.8162486	5.8463534	7.9443738	2.6774933	2.2809779	[illegible]
C1QC	13.408686	31.131627	214.93416	39.900821	182.97782	4.1918499	93.819047	198.66009	94.756553	40.258124	63.998357	74.749635	52.590272	100.43664	69.811229	69.40297	61.651429	37.698391	39.695314	27.364193	111.24824	106.4474	142.232	[illegible]
MXRA7	6.9462465	10.989878	12.807332	3.7909259	1.7790865	3.7874489	9.5195389	3.9942491	1.0763601	0.8903963	4.2090596	8.2638592	3.0379282	2.7127136	4.3538023	8.0660462	4.8759436	2.9319053	2.7019823	5.3909533	3.969064	8.7883265	12.988793	[illegible]
RBFOX1	0.0107103	0.0180359	0.186572	0.0603111	0	0.4848843	0.1519936	0.0025335	0	0.0068738	0	0.0260839	0.0094719	0.0225649	0.0236779	0.0331602	0.0067849	0	0.0682326	0.0050689	0.0277518	0.0954896	0.0352396	[illegible]
SHC4	0.0507789	0.1047069	0.3538252	0.1539694	0.0440483	0.077274	0.2135176	0.1098197	0.0211777	0.0279339	0.0904573	0.2858799	0.0898152	0.1526331	0.05613	0.0898924	0.3032985	0.056358	0.0655436	0.3055549	0.0601496	0.1983385	0.1223234	[illegible]
KCNK5	5.7963246	25.245906	27.426893	16.493411	12.075753	2.9590399	12.851038	4.4598216	3.6031401	20.531606	15.090613	13.27381	12.375188	11.570502	8.1415951	19.492548	13.389537	17.64898	11.67552	11.699019	15.801879	28.863255	10.156975	[illegible]
TLR9	0.0113648	0.0109361	0.0226256	0.0098457	0	0	0.0239936	0.0161298	0.0132714	0.0145877	0.0123232	0.0050323	0	0.0119719	0	0	0.0378404	0.0120806	0.0107574	0.0117791	0.01351	0.0140224	0.0123442	[illegible]
LCP1	23.602962	6.472562	24.9828	7.2651888	3.9732798	4.2635045	24.921335	30.637582	9.9097345	3.1902708	11.100492	18.797905	17.991797	16.368395	15.475463	12.581744	26.920706	12.239698	8.9697817	4.2116319	8.6468558	20.656271	39.709197	[illegible]
DOC2B	0.402439	0.5644754	1.3104261	0.5791045	2.5817381	0.0403667	1.4699146	1.8490454	0.2827675	0.3764795	1.0021842	1.2987473	0.4403594	0.5892014	3.1449936	0.6902878	0.9765564	0.8100357	0.4096589	0.7329036	1.2018415	0.7703098	2.1741527	[illegible]
ZCCHC24	2.4729427	8.006172	13.547585	12.152737	2.4233767	7.4926521	12.279384	5.4953637	0.9812418	5.5996155	3.2054993	3.3274738	2.0329937	6.751113	2.7046644	4.8915972	6.1394818	3.044238	3.012816	6.6307929	4.7742111	10.019934	13.949013	[illegible]
EBF4	0.9584414	1.0361447	2.9681664	0.9635876	2.0237635	0.3221171	2.4566191	0.9906289	0.2348997	0.5923384	1.0264332	1.1474474	0.4918297	1.5082306	0.7324532	1.7595398	0.9444781	1.1686072	0.7420932	1.2824183	1.250924	1.5960545	1.7957554	[illegible]
TYRP1	0	0.4016171	0.5266294	0.2648144	0.0237962	0.1878558	0.9145497	0.0834299	0.027458	0.1056351	0.0828627	0.2811171	0.3431118	0.7183155	0.0129956	0.8255633	0.5436872	1.2135025	0.7935696	0.2336944	0.2315204	0.4402402	0.1982483	[illegible]
BRPF3	9.5771669	11.734214	5.7001318	12.235161	3.6413661	7.949691	7.8583446	4.8014674	5.7977221	10.534514	9.2922135	10.749186	9.9167308	10.186871	7.5060535	4.9449639	3.5726209	14.593392	10.017753	13.021883	7.9686111	12.582733	10.219314	[illegible]
MYL6	67.886003	155.52542	343.74638	132.35093	183.51901	40.485583	161.06733	219.17979	217.23385	171.13929	127.73728	142.68128	157.86422	253.54073	156.68274	415.55264	297.53633	154.43858	202.4339	200.46828	170.93687	144.48651	134.67222	[illegible]
HSBP1L1	14.460945	3.1494877	5.3873808	4.8184368	2.2082217	3.5953714	4.396296	2.487383	6.907138	3.349921	3.7925204	7.8525849	7.6809051	6.1294399	9.3402483	4.4181051	1.9649831	4.6578985	3.3437205	4.4649026	3.7026843	4.0433457	4.5838246	[illegible]
TUBB2A	7.1562757	5.1585941	18.607327	6.034217	4.7423226	1.1002188	5.3141212	3.6265372	14.572359	5.8840725	2.8995476	10.994856	3.9864875	7.5653966	5.0953107	12.097473	7.8407544	2.8547919	6.7948577	5.496159	9.5551949	7.1750159	6.0959969	[illegible]

[illegible]

LOC1019284	0.0906841	0.0436315	0.090269	0.0130937	0.0611833	0.2683347	0.2065445	0.0143006	[illegible]
ANKS4B	6.2758969	13.274414	14.938873	12.94897	8.683292	14.096757	10.443063	9.4044511	[illegible]
CCDC107	0.5727583	2.7847585	5.5513256	2.4156965	2.0440199	0.0178399	2.8203174	6.2179779	[illegible]
NDUFB1	5.7333776	14.428559	17.102042	11.819735	9.3034107	4.0486165	15.905134	20.945327	[illegible]
TNIP1	45.181381	25.449771	46.969709	31.571385	25.117534	4.4571441	35.687242	23.041519	[illegible]
EGR1	22.88792	60.97519	281.18269	84.765793	52.142058	77.537946	35.309191	18.400432	[illegible]
SNORD38	31.027809	101.09202	105.2005	145.66211	49.714545	34.530485	101.7825	111.69974	[illegible]
ZNF883	0.3235278	0.1297176	0.1789148	0.0622848	0.0363799	0.0850951	0.3684376	0.0680259	[illegible]
ZNF682	0.1327953	0.149083	0.1321874	0.5292044	0.0089595	0.094306	0.628182	0.0628243	[illegible]
ATP6V0D1	7.1071432	12.817356	29.876482	14.715388	41.860335	2.4564125	17.222406	11.338669	[illegible]
MTF1	6.9612284	2.1519929	1.8278231	3.1506919	1.0095924	4.1975114	2.3393672	1.9033696	[illegible]
FAM118B	7.559016	5.5076508	6.3860725	6.0730537	2.2683622	3.539492	7.4412286	6.9412127	[illegible]
TTC30A	0.272356	1.9337322	0.6521273	1.2435196	0.5363647	3.1844028	1.5591949	0.9785576	[illegible]
SERBP1	25.306745	7.6457482	5.8072936	6.9071335	4.7882373	15.962335	6.7698893	10.721883	[illegible]
ABI3	1.7370665	2.1712697	8.1464445	2.3426344	4.8207974	0.3603289	7.6711863	6.3709978	[illegible]
ST3GAL3	1.6927502	1.2383364	2.1776909	1.4364629	0.9717645	0.6326973	1.2019017	1.6250665	[illegible]
CCDC87	0.0756221	0.1309844	0.0451655	0.0655136	0	0	0.0635959	0.1073284	[illegible]
LAMA3	13.072244	12.608938	3.5744433	5.9808471	6.2477103	0.979923	5.9203154	0.7847787	[illegible]
KIAA1614	0.5176868	0.315499	0.4680794	0.5792958	0.1091487	0.1914796	0.3809389	0.1836848	[illegible]
ARNTL	3.7468891	1.3434225	1.523138	1.4389072	0.5955953	1.1957769	1.3301095	2.4253654	[illegible]
ATOH8	0.484987	0.4423108	0.9367115	0.2633828	1.6116492	0.1113792	0.4375356	0.1746507	[illegible]
PHKG2	10.024122	23.491744	34.949591	18.271576	22.976287	3.0197923	23.287275	16.187624	[illegible]
MRPS36	4.0244792	14.605421	13.735045	17.482422	4.5674503	5.0695961	13.462309	16.727524	[illegible]
SIPA1L3	14.762738	9.4925675	7.7478908	9.4565521	7.696259	6.5707074	7.5200462	13.267617	[illegible]
HIST1H2AC	15.135227	9.8828684	9.0290626	8.7033181	13.102406	23.282055	13.290944	31.622769	[illegible]
GIPC1	14.452581	38.056912	66.0651	42.998571	92.380003	2.5383614	34.920352	27.991587	[illegible]
PRKAR2A-AS	0.1347413	0.2539136	0.2235497	0.2820982	0.1477255	0.139545	0.2419711	0.2018589	[illegible]
FGD4	12.546133	1.8037543	1.0832872	2.4502029	0.259636	14.328021	2.0922614	2.5155254	[illegible]
LOC1001298	0.1644521	0.3340789	0.2546433	0.5540486	0.1849224	0.1730185	0.2209946	0.4149379	[illegible]
LOC1019283	0	0.0381284	0.3944185	0.0686535	0	0.5627776	0.0416524	0.0374908	[illegible]
ALAD	8.1026089	8.7354444	12.010412	7.768886	9.2987687	4.82647	10.350039	13.040645	[illegible]
LOC1053693	0.14877	0.2147361	0.0538505	0.1698922	0.0136872	0.2961418	0.0995201	0.0575851	[illegible]
ANKRD6	0.591423	0.2769333	0.5992282	0.194425	0.1496342	0.2406281	0.3885693	0.1823677	[illegible]
SETD9	1.4114413	3.7545437	3.2621364	2.0098445	0.9851159	2.3138545	2.9930287	6.8923099	[illegible]
PUS10	25.271943	6.4965917	4.7690634	7.0258234	1.577909	6.7232358	6.8901757	3.4482075	[illegible]
RAB9A	8.2021875	16.997373	17.657692	7.7399055	2.7233738	11.071334	10.172428	17.894574	[illegible]
TRIP4	5.513509	8.9167558	7.1640744	6.3261447	1.476595	2.7066281	8.2661132	7.2411106	[illegible]
OSBPL11	5.9083174	6.1396465	3.6686522	5.2776489	1.6143271	3.9561277	5.2180322	3.9618973	[illegible]
SLC5A10	0.1431744	0.0262425	0.0339331	0.0206727	0.0965977	0.0605219	0.0179175	0.0677345	[illegible]
CTDSP2	47.141839	56.426148	41.978392	57.297606	21.936424	26.721228	50.868656	37.412805	[illegible]
MSH2L2	21.572202	11.313492	9.6096097	16.23987	5.5489764	7.5093821	12.290417	3.4194222	[illegible]
SPAG16	1.3050191	1.5479476	0.9703068	1.3751495	0.0916415	0.2017466	1.6294235	0.1159187	[illegible]
SH3GL2	17.278922	8.4711945	6.6723429	8.1040321	0.8140385	23.084173	12.051789	8.1228286	[illegible]
ZNF569	0.4559518	0.5189066	0.3578546	0.1139435	0.0177475	0.472207	0.5576493	0.2322996	[illegible]
UPP1	10.99305	2.4827613	9.8427109	5.1988521	6.1072308	1.6776047	4.4345227	12.247139	[illegible]
AK1	2.0943718	1.7535205	1.7400556	3.1430812	5.4240783	0.2439863	2.2016262	4.9541447	[illegible]
HERPUD1	16.876046	22.885363	26.174852	17.650698	11.138498	13.546919	28.269675	34.349377	[illegible]
ZNF429	0.6004639	1.0007008	0.485102	0.7652207	0.1937551	4.4136116	1.0565986	0.1687977	[illegible]
ZNF658	0.208875	0.1835171	0.162719	0.2202924	0.0643353	0.3977093	0.2482116	0.4597135	[illegible]
[illegible]	[illegible]	[illegible]	[illegible]	[illegible]	[illegible]	[illegible]	[illegible]	[illegible]	[illegible]

BRD4	21.018016	13.846777	7.4614667	8.60328	10.828648	6.3107132	9.5102583	8.3518694	5.7463206	12.711928	8.6582007	8.4468087	7.9735542	8.9114954	9.3671247	7.9771841	3.1748929
GDI1	20.409367	27.707728	37.100104	21.128304	19.58781	12.613108	20.416583	53.796017	12.766744	36.776996	30.732162	23.425041	15.897215	21.120607	23.290821	33.769789	10.156734
ARID5B	28.622654	4.580102	5.4001614	5.3182318	1.7773797	15.274665	9.9898476	4.4194078	3.8406858	2.2404738	9.0340025	12.022887	9.0904389	6.8952213	4.9783763	4.6397247	14.142646
ITPRIPL2	19.46668	9.1272171	10.803886	5.9381853	3.2605373	16.806551	7.8672699	4.3740303	6.4096613	5.3267465	12.454696	7.0440426	10.152799	8.1328951	9.0045231	5.8359843	17.514926
NBN	10.781116	23.01064	6.2015199	9.1171703	1.2436422	19.500207	13.349785	11.181109	17.808349	3.7691295	10.597923	12.353888	14.646178	9.9647017	6.7365064	8.1599157	20.557126
DIDO1	8.9414113	15.528776	6.1841829	10.405734	3.0799973	8.8956551	7.1591459	3.5877542	2.811446	15.28575	5.5497256	7.7630857	7.110946	5.7595854	5.9155485	3.8096368	9.4635912
ERCC8	1.4246222	1.9558998	1.4271326	1.4110661	0.4224258	3.2542292	1.4546553	1.69138	1.4728818	1.1996707	1.8005755	1.7196971	2.6562225	2.0455763	1.3741432	1.2743685	3.5812031
PATL1	39.581572	20.55284	9.5891271	16.432798	10.612678	26.874175	19.804619	13.472772	15.661358	16.931044	25.054873	22.739239	17.293353	25.223401	17.319232	12.400272	5.0539956
TPBG	3.3636146	0.4842815	1.9848323	1.9812971	1.0186428	0.3204547	4.7379333	4.0234486	4.8763854	0.7522893	2.5005818	2.242578	1.2732601	2.7916918	4.633504	2.4603675	1.3761555
PPL	9.4590072	5.5835341	8.4872336	3.3710443	12.561016	0.589247	9.7449118	1.8814921	2.2018166	4.7743123	8.5658272	7.0086281	5.6629038	4.4486774	4.3138837	4.6446321	8.4137321
ZNF75D	4.5536232	2.7370926	1.964631	1.950117	0.4765053	11.710709	2.6578389	3.942392	1.4385958	2.2850002	3.9829634	2.2990923	2.0991168	1.5831066	1.7110964	1.9315523	6.1330135
ALDH3B1	7.6931657	3.3948722	9.1908561	7.133477	6.0861461	0.695297	5.7426412	10.5984	3.654739	4.3537884	5.0346533	14.96055	3.070151	4.7687864	8.9220896	5.360243	3.3765877
RSPH1	6.0791456	0.7365366	0.4835191	1.4919738	1.8173762	0.3832839	1.0676571	1.5180773	3.6698064	0.5668113	3.1761848	2.6788133	0.7550173	1.7986735	6.6546796	1.2160139	0.2704153
GXYLT1	4.1242112	2.9242456	1.0569911	3.1536261	0.2909204	13.418702	2.9509219	1.5264872	2.8533405	1.4061598	2.9802683	3.0655715	4.2751286	2.6697535	1.6536253	1.7568701	3.229581
ALG8	7.6279355	15.06083	6.831173	9.5333056	3.2402019	8.0697295	10.901508	3.8018175	14.284908	5.583223	10.560271	11.81614	15.815766	12.926553	9.7571566	11.170118	6.7796711
SNX22	1.5710513	0.1871379	0.4479013	0.264281	0.9030286	0.1895605	0.4730046	0.577282	0.5389049	0.773348	0.3431875	0.7024163	0.4991042	1.1809794	1.2139393	1.1340748	0.9324408
LINC01806	3.0160012	2.4927025	0.3131122	0.8175172	0.3370613	1.9381733	1.2642922	0.3238848	1.1343739	0.7125083	1.4245	1.9172021	0.6709098	0.3313559	1.0635517	1.6050156	1.4651927
ADAT3	0.4325435	2.4405928	6.4193067	2.359064	6.7054746	0.1396252	1.3950863	2.3811785	1.5841971	10.977947	2.0679452	2.5160142	2.9559182	4.5150799	5.2376382	3.9389088	1.2455121
TXNL4B	2.9136214	5.0132836	3.3284194	2.8306592	2.5133871	2.9969682	4.667174	8.3360908	5.4762535	3.1165682	5.3257071	4.5646846	9.067648	5.3785286	4.0028165	3.5532157	3.5069337
NSUN4	5.7444356	1.3619377	2.4816507	3.1227308	3.0854809	3.2550948	2.467862	4.1821219	4.6459446	2.3133773	3.486126	3.1025038	3.5506521	4.0487951	2.9107945	4.2476922	3.2047343
HSD11B1L	0.1617519	1.2239715	1.7272128	1.9873031	1.2946979	0.165343	0.9815685	1.6765581	0.8070632	1.7931022	0.9487108	0.6771707	0.5071748	1.037847	0.6339173	1.5489627	0.4005585

[illegible]

SNRNP48	5.237724	3.8362949	1.5903798	3.7737097	0.8084556	9.696004	3.0975958	4.870274	6.6532313	2.1475117	2.8723895	4.2213996	4.7054254	4.0170771	4.0152833	2.1790736	5.0510807	[illegible]
BDP1	14.171366	3.5989123	1.2110386	2.6441495	0.2970729	11.381697	2.4927651	1.0708219	1.9803138	1.1167404	2.6384022	3.1320631	2.755327	2.0937775	1.7357481	0.7231254	10.901978	[illegible]
C22orf24	0.4262775	0.2774852	0.0998414	0.0217233	0.0253768	0.3116293	0.2635929	0.0474513	0.0585634	0.1609305	0.2854914	0.1221362	0.2439341	0.2905615	0.1663053	0	0.2382734	[illegible]
POM121C	7.8160896	7.890518	3.7553213	8.0164776	3.2290273	4.4147741	5.2762986	4.4999253	3.4886457	4.9390362	6.8323784	8.0770745	3.7978013	3.4350833	3.9150574	2.8700398	1.878237	[illegible]
C11orf49	6.4398883	5.7479721	4.9255464	4.6511159	6.5505989	2.5316793	5.0641724	4.8933004	6.6454313	6.8237883	9.3485938	4.5858158	9.9841664	12.903277	7.5761387	10.111011	7.3433069	[illegible]
MRPL37	27.866073	36.727121	28.580101	47.736768	31.858619	9.2003317	35.55988	52.471564	54.845928	62.434277	53.528035	35.061415	62.777487	65.616384	66.508876	60.830703	39.645294	[illegible]
SLC35B4	4.0399664	2.6909245	2.4633819	2.4923003	0.876567	6.791785	3.0046697	2.8127611	2.8681912	2.4697314	3.4112671	3.40247	1.9314153	2.2680136	2.0926536	2.3127528	2.8375529	[illegible]
[illegible]	17.362625	6.1070633	10.7636	8.4980326	13.637099	3.2530017	11.639571	11.732404	11.112369	13.039819	11.802397	14.843614	7.4481065	15.905855	14.529285	5.7617106	11.708848	[illegible]
GTF3C4	6.7604461	4.5841994	2.4158406	3.8574628	1.7178233	11.769367	4.2512143	4.820471	5.4921204	3.9267745	6.5394682	5.9849047	6.3357742	4.7198963	5.9890384	2.9230726	6.2761371	[illegible]
ARFRP1	6.4585847	8.6620513	11.250779	8.6401792	13.580265	3.3546584	7.2288285	3.431981	3.0168268	13.44928	4.855355	6.9249583	3.2791909	5.1330783	5.9089515	6.9910677	4.7636272	[illegible]
ARTN	0.7115191	0.2308791	0.8400313	0.9747873	0.7703152	0.1077175	0.1913379	0.6967115	0.1545828	0.6584224	1.1124231	0.373677	0.5268136	1.4293343	1.8107757	1.6403836	0.9119651	[illegible]
[illegible]	0.3286312	1.2385796	2.0718023	1.1862614	4.4898836	0.0648281	2.3030731	8.8101104	4.0294996	1.1248699	1.3659839	3.6622048	0.4359498	1.6732367	2.6033787	4.5248438	2.1512459	[illegible]
ZNF30	0.6729457	2.1353989	0.8134074	1.4505405	0.5188875	2.0576235	1.5074536	2.6681995	1.9458847	1.7070083	1.2161551	1.220302	1.2186124	1.19837	1.310611	0.4301898	1.6747713	[illegible]
VASN	0.8307327	3.2426166	8.1288727	5.7303443	9.6604027	0.8348402	14.829214	4.0339379	2.2513528	4.1043118	4.6653901	5.6773655	2.4951365	7.0008878	2.7028464	5.8917305	4.6668529	[illegible]
[illegible]	0.0820556	0.2763598	1.2251993	0.4620665	0.6643406	0	0.4312891	0.5822969	1.0061219	0.5266289	0.4448755	0.6903511	0.6531113	2.160977	1.088431	0.847353	0.67576	[illegible]
ATP13A1	5.7025124	12.695242	11.844139	11.563647	23.63176	1.2709696	14.235671	10.001268	12.079687	23.74509	12.087782	16.347888	9.5104871	13.535435	14.586136	11.560582	6.5397443	[illegible]
[illegible]	7.6874623	7.9586133	11.286531	9.1573232	9.9601425	3.6502924	9.3088179	3.0280437	4.9056272	15.921918	10.819225	6.1227037	22.361172	9.085887	15.415931	2.166113	12.182039	[illegible]
SRD5A1	2.0066878	5.1511151	2.8941065	3.2472502	3.3750914	4.063366	3.4728081	7.2576454	2.5496992	3.8343628	4.8246993	5.8867262	1.5225813	2.5943152	4.8333013	2.2606038	3.0624841	[illegible]
DYRK4	2.232136	1.5959445	2.1412329	1.4422207	2.1643339	0.8080853	2.5135828	6.223934	13.648223	2.0248037	2.8292931	2.208695	2.0567564	1.9639606	1.9573447	3.039035	1.8919688	[illegible]
ZNF286A	1.1395938	0.7300229	0.1750181	0.4654241	0.0856738	1.3912164	0.3662318	1.18609	0.5399125	0.3510638	0.5225212	0.4094572	0.2821919	0.3704313	0.4318915	0.1882895	0.7301736	[illegible]
PAQR7	1.9609724	3.9087685	2.5893808	5.8072838	1.8900561	1.0736642	2.678414	7.16931	0.9268813	2.0718784	5.6774588	1.665756	2.5128707	2.4732536	2.1086356	2.9755274	1.8422623	[illegible]
G2E3	3.0283025	4.4421014	0.957416	1.3736283	0.2505752	4.7762526	1.5616616	2.5116661	2.0878779	0.660071	3.4075816	2.698735	1.9917722	2.0464642	2.2579316	1.2784141	6.1533716	[illegible]
[illegible]	4.4741697	5.1532121	4.408041	4.6535935	1.7303134	6.1911611	4.5518024	3.7685147	4.0275705	2.8588767	7.5932841	5.8970833	7.5197401	5.9691116	4.2287883	4.2888127	8.6109424	[illegible]
FAM208B	16.493178	5.2632851	2.684349	4.660282	1.1493214	14.113792	6.1209684	4.3627211	3.6551917	4.4607223	5.9896154	5.259717	7.8822006	5.2630043	5.2342672	3.2074475	13.933595	[illegible]
ZBTB41	2.4708304	3.5417065	0.8232484	2.3675128	0.1195689	11.506393	2.1383719	2.8287576	2.4774274	0.8176065	3.6065987	2.7636451	4.6746557	1.8236001	1.7432015	1.6866678	6.4757753	[illegible]
EIF3M	12.759935	24.933142	14.917654	23.157608	5.5548642	8.6831626	21.355636	24.491002	26.36459	14.197204	22.391109	26.653818	31.429218	24.856007	23.640697	21.327195	33.5298	[illegible]
EIF2AK2	26.169033	9.5267997	4.2913233	8.8143314	0.9313619	23.506222	8.8766382	6.7532645	5.0247239	4.2998381	14.879768	8.7151967	9.4446682	9.3240059	8.0161074	3.8520716	12.644571	[illegible]
[illegible]	15.377954	4.8940202	4.0919909	4.4730407	2.5344085	3.9961661	5.7437145	3.9769603	4.4668615	4.1573027	3.9614124	2.9101482	2.2375216	2.3937184	4.0312022	4.1075475	5.913112	[illegible]
ELAVL1	8.668084	17.489078	11.343371	12.316084	11.444379	8.9439446	14.211303	11.951813	11.245709	14.65113	15.941899	13.949437	19.935578	19.042886	16.23765	12.74511	8.4103083	[illegible]
TUBB	142.80012	278.91415	156.13161	205.31681	212.6422	102.57958	209.389	97.50801	211.33493	220.72513	164.76631	158.86264	117.4998	258.47071	215.55427	179.88767	140.35586	[illegible]
RPLP1	39.087471	322.62032	395.59007	257.87186	1294.7056	46.558404	369.17388	979.48757	652.44322	690.1383	433.09412	508.51564	337.64126	745.66415	846.93329	461.34591	243.09282	[illegible]
PRKRIP1	5.0098326	6.1605843	6.4201857	6.9329211	3.2756881	3.7253942	6.8988521	7.6732946	4.0854648	4.9947487	7.7355376	6.6866852	4.7778594	5.8164895	3.6304592	7.1273024	6.5509115	[illegible]
[illegible]	2.7862186	8.4822354	7.1528504	5.781397	9.5461544	1.6728214	6.4105241	4.0288176	6.8929733	7.2738334	4.9946787	5.7130079	3.3066118	5.9648	5.5536263	6.784642	2.8411546	[illegible]
IRF5	7.5466941	12.923207	17.920676	18.960972	18.278297	2.7441711	18.425435	30.558481	19.209264	19.135574	22.336925	17.611835	12.148719	17.572696	18.362563	18.986067	16.038702	[illegible]
TMCC1	9.8043602	4.3112793	3.482727	5.2629406	1.7419505	14.307499	3.6276605	3.3876237	3.7933847	3.7111607	4.699533	2.7921299	5.0094233	3.205686	3.3078427	6.5638158	7.4225303	[illegible]
[illegible]	[illegible]	[illegible]	[illegible]	[illegible]	[illegible]	[illegible]	[illegible]	[illegible]	[illegible]	[illegible]	[illegible]	[illegible]	[illegible]	[illegible]	[illegible]	[illegible]	[illegible]	[illegible]

[illegible]

SNX4	11.753445	10.374199	1.5478723	7.5489062	0.3789499	10.513566	5.1717567	4.9453576	5.8726788	2.8170462	[illegible]
GPR3N1	1.242896	3.5427905	1.3307761	1.6422804	0.7504793	0.2410615	1.25165	1.2303597	1.7685155	1.7428337	[illegible]
MAP3K10	1.8397997	3.9254469	3.7586374	3.7969198	2.3688413	0.8778763	3.0326633	4.3200663	1.8897288	3.4743076	[illegible]
STBC	0.1223638	0.0102389	0.0105916	0.004609	0	0.0472272	0.0111852	0.0679569	0.0529077	0.0546311	[illegible]
CRYBB2P1	4.5519634	0.8352698	2.0342433	1.6816117	4.4407066	1.9293826	1.3112261	1.9853533	0.8579975	1.4212298	[illegible]
NEK3	8.9466741	7.9107065	5.4008192	18.565263	2.0734207	11.19639	9.1072269	4.7293991	2.1700616	10.981651	[illegible]
FNBP4	18.870026	6.1204972	4.0606427	8.3218016	2.0392003	10.732115	8.3904469	11.789769	4.0027905	6.3470331	[illegible]
TOMM5	7.7125071	5.2728945	6.9323058	6.8227283	5.3989525	4.7856944	5.5255189	6.9948064	22.445391	7.0955319	[illegible]
MTHFD1	11.181895	25.449032	8.6701826	7.1252119	8.5110272	4.1692174	10.736371	9.7102715	13.53975	8.8019988	[illegible]
TAF6B	4.7038932	3.1575194	1.0630657	0.9181377	0.5610261	2.8609599	1.9710592	2.6419044	2.8988247	0.899922	[illegible]
C16orf95	0.0119841	0.495877	0.5010297	0.2907019	0.9460065	0.2482279	0.5102138	0.515933	0.7137239	0.3691841	[illegible]
LRRC14	2.8886357	8.7010868	6.3572987	5.5111082	6.4851164	1.4846744	5.4251334	3.0858444	6.8636418	6.8543894	[illegible]
ZNF215	2.709935	1.5509498	1.4894709	1.4962426	0.243289	3.2980165	1.5794289	0.1395776	2.048025	0.7153229	[illegible]
POLR2J3	0.2195597	0.1824661	0.5463864	0.2031799	0.1616004	0.9479393	0.2412949	0.382437	0.2243437	0.3586847	[illegible]
PKN1	5.9298096	39.575147	24.428087	21.545489	10.456081	0.599005	18.429435	28.26573	18.459986	21.71889	[illegible]
LSM14B	9.2620668	29.241474	12.987813	22.62384	10.55832	16.959688	33.462014	9.2374797	8.888624	21.055607	[illegible]
SLC2A3	17.074282	2.3913362	5.4592286	4.0088585	1.0117738	3.934486	6.0429494	16.101332	3.2272073	0.8249548	[illegible]
GPSN	3.8091794	2.4235884	4.7990964	4.4722422	0.8745663	1.8303366	11.851397	4.6639148	5.6233234	1.2405958	[illegible]
NNX2	5.3902745	0.8369136	3.4663302	2.3231939	0.4366816	1.8293938	3.5897323	1.2168354	0.2558756	0.5408754	[illegible]
FAM219A	9.8158637	5.2203752	6.6909314	6.1858983	3.4483529	5.560814	5.6845319	8.0654838	2.9536827	5.1349328	[illegible]
C9orf33	10.363979	72.285323	34.837733	34.245069	13.914573	22.776832	40.608896	33.607228	37.417765	23.414039	[illegible]
LY6G5B	2.4668121	0.2722826	1.0110959	1.1125339	0.7342597	1.2022385	0.7931963	0.4393514	0.2541742	1.3782996	[illegible]
ACPP	4.4999275	1.2112338	0.522066	0.4089249	1.9850998	0.7138739	0.5899187	3.5729391	3.9749061	0.1615676	[illegible]
TMC6	4.4037617	5.1774184	8.1712741	5.6061845	22.68414	0.4383615	5.7626076	7.8942859	7.2368867	11.468014	[illegible]
DTX2	6.1236076	12.729495	11.157851	9.5294574	19.236231	1.7035686	9.2019065	9.92912	11.627844	20.131897	[illegible]
NMNAT3	3.0077389	2.4772966	3.9200697	4.4109055	7.5066061	2.2626888	1.3866239	4.4738037	2.3514635	3.3862829	[illegible]
UPF3A	5.448197	5.3783045	3.1174232	8.5849934	3.06918	7.6344748	9.6161033	1.9610585	2.4959314	6.5041893	[illegible]
NFS1D10	3.3136025	20.23684	44.168505	27.24121	54.514836	1.4748639	26.549169	15.985938	27.224608	31.470785	[illegible]
TTL	3.3436463	2.4682761	2.187353	1.816491	1.5702711	2.8986694	2.3363961	2.1307357	2.7227326	2.2822911	[illegible]
PKP4	17.805129	14.154164	9.0180842	12.48289	4.1076128	12.900827	9.3414912	5.8632107	7.1068399	7.6635746	[illegible]
COPS6	14.27411	59.571181	59.92323	44.17716	56.530487	5.2668925	38.319861	51.147529	35.917326	58.163928	[illegible]
TMEM237	1.3746113	1.7793504	0.8416015	1.0289278	0.244469	1.8312166	1.5659286	1.7795272	2.2331951	1.0778532	[illegible]
SACS	3.2760351	0.5945744	0.3192239	0.7676108	0.0783395	2.5768326	0.9154422	0.1373297	0.6279179	0.1383947	[illegible]
NCL	107.2727	230.75214	65.465333	103.72253	57.885266	55.396413	140.36738	35.449769	98.747242	90.022333	[illegible]
AARSD1	2.7310518	0.9826505	0.5909889	1.1881361	0.5167293	1.8551605	1.3489812	2.6233962	1.4559432	1.0059371	[illegible]
NIBZ	1.1067006	2.3751207	6.5718123	3.3878256	14.550931	0.3711142	2.1437435	13.139022	3.7844922	7.1541971	[illegible]
YIF1A	10.370448	44.095619	64.315792	35.15878	114.58491	1.3808785	30.621636	44.33073	47.759514	91.9672	[illegible]
TMED3	3.7024234	5.8918958	5.9610675	3.7955941	9.6131485	1.5935248	4.7184048	7.2959513	11.419517	7.7099265	[illegible]
RASL11B	0.124544	0.2225699	0.3365003	0.3930499	0.18006	0.4527605	0.6639619	0.6397099	0.1558256	0.2055379	[illegible]
CATSPER2	2.6222642	0.2593648	0.2709251	0.4103648	0.1430194	2.3293353	0.3521348	0.3318092	0.2016999	1.0883749	[illegible]
RILPL1	1.3130146	1.223241	2.3226368	1.0904086	1.5404011	0.6731072	1.3406949	1.5628306	0.3173995	1.5458138	[illegible]
YIF1B	2.8237616	10.974962	13.300044	10.292387	25.022525	0.7662623	9.7739054	13.991076	17.307704	27.475775	[illegible]

[illegible]

DEGS2	2.33401	2.857197	5.5681366	2.8581303	115.09426	0.2272974	2.9142242	4.0021413	19.199872	10.694229	23.412843	15.737994	7.1069117	5.4568492	27.999624	25.363366	9.9195581	6.8532132	26.80957	1.7944394	18.811782	3.7228476	6.4360374	6.1613606
FAM151B	0.7639169	0.1701613	0.2816369	0.2696236	0.0572671	1.0213807	0.2097596	0.2007791	0.3964757	0.0726335	0.2607723	0.3883758	0.4128599	0.3725583	0.5003958	0.1753031	0.7975964	0.1884109	0.4210525	0.1941619	0.4105449	0.277479	0.3723682	0.2765825
ACSS1	4.119341	23.515762	4.2616804	10.732418	6.8627891	7.6549019	6.8385749	7.050721	8.497971	5.4174405	6.3818008	15.096561	10.110112	6.4238032	8.6313875	4.7032917	6.1873044	7.868896	10.484339	5.1038804	9.6195089	11.469221	14.103179	2.4020639
PGK1	78.777389	213.03902	129.45755	80.469558	81.689481	85.684836	92.216509	164.56104	206.63086	162.08618	213.55109	123.49379	150.62211	120.27327	195.3821	366.43155	239.56265	97.997829	214.342	88.236181	142.45462	127.42659	84.194629	155.58742
CUL4A	12.96706	19.087896	9.9478273	24.51328	7.4289549	14.960826	22.843886	8.562001	12.202028	17.541114	16.212035	21.405608	31.565949	23.404131	12.708643	12.674824	17.172602	21.655874	16.079291	41.337371	25.072118	13.285219	18.594678	14.145205
PIP5KL1	0.3554351	0.13301	0.104832	0.0342138	0.0399676	0.0077906	0.0761114	0.6912967	0.0614907	0.0337949	0.1784296	0.0874372	0.0582107	0.1525426	0.4219937	0.2446951	0.166789	0.0876638	0.1189437	0.3987404	0.2319507	0.0312983	0.2057408	0.1143896
MORC2	8.2228693	9.6913042	7.8634118	7.3398568	5.4980608	3.6933965	7.3990962	8.8010679	7.5956855	9.6977449	8.8705531	8.6048003	7.7444746	6.5003073	6.7121649	7.8382182	7.89827	7.4102603	8.8714997	7.8332527	8.551767	7.9578598	8.917292	6.4276082
XAB2	3.3976959	22.82319	19.599653	13.33962	45.082327	0.707386	12.654298	15.165853	10.117055	35.385183	12.338541	14.286513	11.028833	13.464149	11.839433	15.997908	10.630887	9.3700537	11.455309	13.692682	25.33375	16.027497	12.786394	13.085401
KIF27	0.8237348	0.4363996	0.2640455	0.5745059	0.1298954	2.1268394	0.4542464	1.4937614	1.0092165	0.3954005	0.6634011	0.6820698	0.749172	0.4777357	0.690469	0.2474139	1.8159154	1.1966106	1.4371197	0.7491959	0.2749298	0.5492847	1.400662	0.2509421
ANKS3	2.2248042	1.5249665	3.077096	1.5811562	2.6532251	1.1776986	2.4856461	2.7688556	0.9628392	3.7669756	1.6789923	1.834151	1.0431039	1.9844515	1.6312631	2.3090046	2.2948929	1.6000662	1.6726806	1.2619461	2.6419452	1.847342	2.0254659	0.6860993
FN1	325.20121	50.204075	97.568892	56.015283	32.917185	9.9589125	140.75726	25.25249	10.137063	103.13077	25.032429	165.26055	42.290716	44.80347	15.844261	59.044868	116.06502	92.951209	15.599707	37.556655	37.242127	87.377949	341.14936	24.797816
RSL1D1	32.934651	51.779385	29.759634	29.452837	9.469466	30.135111	44.033814	36.975577	47.778995	17.576206	37.243099	39.16192	38.505361	33.078794	31.698927	24.156224	42.007759	41.199524	37.984473	47.537775	16.669343	39.762969	28.270035	28.098381
SAP25	0	0.0609835	0.0630842	0.054993	0.0641366	0	0.0333099	0.2398548	0.1110089	0.0613464	0.2233352	0.1262799	0	0.0333799	0.1050791	0.065444	0.0200736	0.1759434	0.185256	0.0449903	0.0328423	0.2448447	0.2867125	0.0344178
DPM2	15.610524	13.360032	16.433391	14.855559	18.071422	4.4280788	18.483347	23.842513	25.221053	22.722313	17.124796	20.507048	12.737859	15.986015	16.650365	26.47095	6.3674647	12.776117	30.232776	16.370694	23.163596	13.367155	16.801419	11.634213
DDX51	2.7432487	6.712426	4.0444473	4.0976133	5.7762668	1.4312588	4.7251127	3.6776644	3.2478322	6.483609	4.6491832	6.2938604	3.574925	4.2800993	5.3543166	3.3965476	3.3224411	5.8438893	5.4721355	1.592639	6.0589208	2.9037409	4.5869919	2.7770019
HNRNPA1L2	1.6067748	1.9397239	0.5816068	1.5058861	0.561744	2.9391158	2.1904236	1.0365696	0.5202337	1.5561992	1.7739466	1.901594	2.0410602	1.1694395	0.8073162	0.9653932	0.5552063	1.7671001	2.0262766	3.5464464	1.8773625	1.441233	3.6526344	0.5077058
ADK	2.4725954	10.367956	3.9737913	8.1075973	3.3970256	5.9921054	13.97626	11.488512	12.372242	15.337017	15.202894	12.367711	17.444745	17.170537	8.1841987	12.838036	17.790094	12.310846	14.367684	17.108717	9.8214221	8.6373306	10.186397	12.213052
SMARCC1	30.111297	34.098837	15.525824	24.900087	15.027133	26.530673	29.826951	10.775216	14.884024	23.172522	27.354286	25.217095	33.91034	31.386533	22.263786	15.779009	33.434526	37.98498	27.953276	32.847372	28.734441	30.802626	29.311435	21.498561
RPS18	202.80758	875.53892	1151.2091	1011.1875	3042.4952	117.19665	858.59673	2426.766	2746.7116	1611.4491	473.85363	1229.5727	446.16104	1143.1979	1642.2057	1916.6542	1192.9786	1656.5052	1126.5033	1324.5697	1422.8425	898.24501	951.58536	1296.0341
LOC1027243	4.5695386	0.4122324	0.7581031	0.2474202	0.3853751	0.5633867	0.8005921	0.4954145	0.3335073	0.3665871	0.4645194	0.5058485	0	0	0.4209228	0	0.723691	0	0.1011945	0.1802208	0.3946766	0.6790099	2.2317537	0.5170123
LOC1019297	0.3460657	0.4389676	0.062633	0.0953931	0.1910336	1.8990724	0.1157508	0.2083718	0.1653223	0.4038227	0.7646102	0.1671688	0.3616976	0.1325647	0.0347758	0.2599034	2.5709667	0.4539224	1.2875157	0.0148895	0.4238962	0.2430933	0.6726365	0.0341716
MIIP	1.7773577	6.5496647	9.4513636	9.4007409	13.159172	0.5351474	9.628709	13.475765	11.388075	10.306281	10.270143	7.8977641	8.9898819	14.469308	7.427773	13.202006	10.212484	6.4716338	12.562183	7.3344928	8.4997466	10.684718	8.515465	8.7212153
COL18A1	3.5237038	13.543209	19.063114	23.592397	9.1580035	0.4732448	46.261309	14.600576	5.3137401	8.3572389	12.889672	40.31417	7.7017166	15.77168	10.470387	9.8028553	13.628736	8.6977877	10.855757	11.622495	18.330307	17.972736	44.863808	8.7418444
NAA15	9.9267014	6.5497074	3.1555105	7.426734	1.0357749	15.4745	7.1407835	5.028314	9.1329225	2.9122382	9.4379569	6.7958552	12.467516	8.8940707	6.4725332	4.1527276	7.6167513	7.3784103	7.6587957	7.282652	3.3419091	5.2596004	7.9083284	8.971995
MEMO1	1.3649527	0.7299987	0.3270954	0.5146056	0.2131744	6.3475544	0.5579989	0.7772882	0.8905999	0.4326008	0.648666	0.6305179	0.4694394	0.54142	0.2421516	0.3132284	1.371755	0.5330277	0.6358971	0.7058128	0.5580655	0.6009626	0.7623696	0.1738825
OBSCN	0.4590377	0.3322729	0.2386373	0.5947465	1.9244048	0.0846415	0.3085716	1.1451195	0.1415668	0.41787	0.5280253	0.5458524	0.4770348	0.2927187	0.3869572	0.4810616	0.3218609	0.6077387	0.8224182	1.2906137	0.336005	0.1764976	0.4055994	0.0724961
CCL4L2	3.9983462	1.4390265	8.0540994	0.8318775	1.6586094	0.1676852	3.6073923	11.364389	7.2426238	0.4849341	1.433792	4.1822122	2.6729098	1.5919146	2.3316472	5.4618784	4.7068409	1.0133209	0.6023863	0.5066048	2.2841517	9.5435649	2.8636921	3.6247856
XKR6	3.2169455	0.2356217	0.3530004	0.2852766	0.0512699	0.1698921	0.053255	0.6311327	0.0147898	1.0079209	0.3112847	0.1645052	0.485365	0.0978393	0.6533233	0.052315	0.1444187	0.2998755	0.2288679	0.3716339	0.306293	0.1957254	0.5278404	0.3301574
ATG101	2.1035376	8.6325255	15.218464	7.7241673	50.549488	1.1635106	8.6262629	11.322208	13.068817	13.376818	10.129978	8.4256433	8.5963611	10.876787	8.7841154	22.772949	7.5860873	7.5838121	12.422347	18.198719	15.318546	7.5835158	7.888117	12.663521
UBN2	11.898655	2.3865504	0.8282644	2.0230296	0.2499082	20.330667	1.9228977	1.6329993	0.8744951	1.1300528	2.567018	1.5403262	1.8776429	1.3119581	1.1066756	0.5820704	2.977331	1.3517963	1.4979697	1.424031	1.3269934	2.2677737	2.3425335	0.7900766
ZFAND1	7.3163853	15.943762	7.5218436	8.7119047	1.5949535	13.057468	8.9144	32.549224	20.256415	3.684139	7.4184204	12.672262	10.291764	9.279463	9.1141673	12.243708	16.225345	14.935513	15.371314	20.484543	6.3823527	9.4108296	10.246399	9.8060576
FBXW9	1.6644015	5.8298541	5.2395719	6.6598644	17.682198	0.3693727	5.7300655	4.9114931	8.1996422	12.043963	7.1626219	5.6196087	6.9365871	10.169227	13.614483	7.3047118	4.0461997	4.0870853	8.4038365	7.7771985	8.1078589	4.6991085	5.2452823	8.1352482
GTF2IRD2P1	0	0.0156403	0.016179	0.0070404	0.0164489	0	0.0170858	0.0153787	0.0569403	0.0208627	0.0440601	0.007197	0.0143741	0.0684988	0	0.0335685	0	0	0.0172771	0.0076923	0	0.0096607	0.0088848	0
LOC10537113	5.5798898	3.9020636	3.6976603	3.717546	8.1120643	2.6105312	4.3578529	1.7555145	2.9615821	6.2177127	4.6978089	3.1677423	1.2145339	2.8933775	3.7885877	3.5434017	3.734225	2.9217717	3.7666689	2.7730406	5.9749695	3.3422878	4.6630162	2.25674
GDF11	2.3776697	2.3515231	1.0822476	0.7394312	0.9306294	2.0866066	2.267115	1.1416794	4.6335059	2.034628	1.129327	0.8391172	0.6335212	1.6436194	0.3706474	2.2769394	0.9076024	3.8286686	1.6228413	5.0806452	1.0057482	1.3286853	2.7936846	0.6290864
ACAP3	2.1369297	1.8761434	7.7233314	5.3873235	9.231612	0.9484773	3.4482761	3.4084561	4.224136	8.4062395	3.5765586	4.9898348	2.8303549	5.7013487	8.2618941	4.6402893	6.7496573	5.3419011	7.3691646	5.1253052	4.4400005	4.2793274	3.8379213	3.2484267
CSNK2A2	17.446224	16.280512	10.442637	8.4703358	7.1636976	21.987549	13.512035	11.264025	9.4426769	14.888266	12.518365	12.094665	22.425285	13.989987	10.145763	10.866145	7.6948637	15.425402	11.026129	12.266221	16.722254	14.072321	11.817416	10.896568
[illegible]	3.7729059	3.9118083	5.8362185	7.2507181	4.1947371	1.1426108	5.3998856	5.3799258	3.888725	10.322312	3.0923058	7.1296529	1.3315322	9.2923926	7.0878789	4.463158	4.4996794	4.9148323	6.9666499	7.0167423	6.9396633	5.4115698	5.930087	4.0980358
AMN	2.4203687	1.9932984	2.2070464	2.0394404	3.1646577	0.2613824	1.7762382	2.0895167	2.2023273	3.3788739	2.5334762	3.0431173	0.9062027	1.5819098	6.606865	2.1162892	3.0329907	1.500012	2.5023847	2.1195937	3.4699275	2.5097047	3.487727	1.2281197
FAM86A	3.0507613	21.038944	29.709973	10.968296	26.636152	6.409321	15.700918	39.979089	22.518299	35.863233	29.869884	17.95543	13.490083	24.317262	14.978404	30.821314	20.324842	8.0980641	27.699807	13.909084	24.162761	16.652228	9.964607	17.231034
OSBPL10	8.8025432	2.4578589	2.9866927	1.7860533	1.3305673	3.2839853	2.5093708	1.0858909	1.7668982	1.7064542	3.155982	1.9530459	2.5723074	3.6597741	2.6873896	2.0479112	3.6898212	1.6630059	2.5472274	4.9535877	2.9918105	2.5539414	3.3607144	6.5498933
TAS2R50	0.5063064	0.0609249	0	0.201118	0	0.37469	0.0221853	0.0599062	0.024645	0	0.0696526	0.0186902	0.1493144	0.2223193	0	0	0.1871734	0	0.0224337	0.0799059	0.0874955	0.0752645	0.104159	0
TRIM65	5.843973	11.582693	6.3216017	8.7501628	6.6093897	2.2190165	7.8832494	9.428856	9.2108987	7.8914762	12.899431	6.9554173	10.326568	4.3769245	11.71229	5.9301693	7.4612805	6.7609923	14.554601	5.0466656	6.4304694	16.330311	12.735436	9.2828728
PCBP4	3.2336056	7.8536649	9.3119578	7.4995985	10.03486	1.3063662	7.9711963	3.0426459	6.8833163	23.586486	6.0292579	5.50546	4.6958494	8.698126	5.4604627	6.6537669	5.1778464	5.6150799	8.4853582	13.819644	10.413101	6.5602934	8.4310411	2.9160841
ALDH1A3	1.165838	3.2702438	3.5345866	3.2214354	0.9780614	3.1205296	5.6431027	4.9314854	0.7563429	0.6357478	3.6809055	1.5790677	5.8897192	2.889689	0.901243	2.3448718	5.9518322	1.6322114	1.2190153	5.1966617	1.6664007	3.0571328	8.659004	1.0924975
BBS5	1.0039746	1.0589921	0.7591431	1.1248497	0.7815772	0.5998658	0.8676522	0.6074155	0.8849364	2.3419409	0.73797	0.9361418	0.4695571	0.7220206	0.693608	0.6579447	0.7032796	0.9107111	1.1133841	1.5442577	1.1906567	0.6942862	1.0878781	0.5557315
LAMC2	43.02729	30.867049	47.119358	29.81306	17.483236	6.293295	24.23709	7.4955005	21.507986	12.657591	65.777825	26.904485	50.304354	36.62322	22.390124	37.567303	88.560017	28.163424	27.884636	48.890908	34.075643	46.870002	17.630023	34.848764
ACTL6A	9.7059175	32.67184	16.094529	28.667758	9.4714182	10.213073	23.928554	28.484917	28.034944	17.2866	21.567855	18.54248	37.38347	32.728212	23.984181	24.989235	31.228302	20.149935	34.717239	29.254977	23.27013	21.055775	22.317089	23.450497
DPH6	0.8942262	1.6349312	0.9604059	0.7604234	0.2476793	2.8604849	1.0191807	0.7904366	1.1789822	0.803471	1.1737668	1.2754283	0.4869855	1.2493646	0.9000181	0.9720304	1.4728603	1.3790183	1.3282634	1.1538178	0.990391	0.934338	0.62134	0.966174

[illegible]

UBL4B	0	0.0143175	0	0.01289	0.0301156	0	0	0.0563123	0	0	0	0	0.0789508	0.0313473	0.0328935	0	0.0188512
TBX3	12.847123	2.2192653	2.472308	3.2179184	2.9399584	7.9047787	2.8556413	7.2318856	15.744685	7.1920088	4.636814	14.860044	4.3080221	5.4741071	6.2752162	2.9617194	20.575813
FOXD4L1	0.3178131	0.0169902	0	0.0076481	0.0714746	0.020898	0.0185605	0.1670607	0.0618548	0	0.0095726	0.0234546	0.0156148	0.0185995	0.0390338	0	0.0111851
ACSL4	10.846332	14.098713	6.1081071	11.058618	0.9505818	20.20604	13.553141	16.876219	11.897296	6.7850893	13.853708	4.9575916	10.137925	11.772423	10.39781	6.0463094	14.823595
TRADD	4.0731646	17.060556	34.963268	17.728095	108.46072	1.0566582	14.311631	18.972819	34.582161	35.792058	29.040931	25.651879	19.947824	19.23493	52.224665	35.838973	17.187375
RNF148	0.438469	0.016228	0.1007219	0	0.0341341	0.499012	0.0709113	0.0957396	0.2363196	0.2597595	0.0731452	0.0597398	0.2087997	0.0355302	0.0372827	0	0.363233
TIMELESS	5.9569724	11.166222	3.4464504	7.8229106	4.0853865	2.666374	7.4580251	6.2193335	7.2299666	9.6354806	12.730077	6.5843342	8.838269	9.8507866	9.0414478	6.4325423	9.0367466
JMJD7-PLA2	1.0405261	0.215327	0.3563914	0.7245429	0.4585826	0.307892	0.2910955	0.415513	0.3462334	0.7324281	0.5590692	0.5573535	0.1880009	0.3123329	0.5812718	0.2657382	0.9355884
ATP7B	5.9685458	5.6814167	2.8776788	7.5441257	18.323002	3.2768963	4.0474377	1.0874315	3.7179892	11.735508	4.9359233	4.7241039	6.291982	5.8193963	5.3591403	4.999916	5.5308175
ANTXR1	34.981255	16.161839	22.898488	21.665246	5.9003348	17.219808	63.595316	11.927716	3.3539388	2.110574	17.339937	29.513743	12.864089	18.181587	4.3062948	15.337286	21.305915
ADAM19	16.653029	8.1607106	8.4730864	7.0354508	7.1526994	2.1791743	7.570669	3.2113198	1.1833363	2.007854	10.319356	6.4361083	4.0895754	6.9280519	2.6125734	5.8246499	15.525963
NQO1	57.876492	89.951629	104.74211	77.546169	3.9668525	42.690199	58.751809	26.581559	21.994573	52.099749	72.620526	107.23911	80.828979	91.367011	78.303477	40.756273	117.69898
LOC1053718	0.5393533	0.1887292	0.0488076	0.2336287	0.2481094	0.3191896	0.2319441	1.3454065	3.6072332	0.1258738	0.4519181	0.0434229	0.0433628	0.2066058	0.3251945	0.2025334	0.0621229
PHF6	6.2494112	8.062188	2.5888143	4.7129295	0.3997978	27.600042	5.2659453	4.898151	5.6799844	3.2959908	9.3673821	5.5782369	7.5599552	4.9533389	4.1120314	2.8556303	16.068676
FPR2	2.6389085	0.2459282	0.5087999	0.2317057	0.1323295	0.0351736	0.2936496	1.0178759	0.4997202	0.0762897	0.244898	0.3473949	0.3574264	0.3756596	0.6569809	1.7676241	0.1355454
[illegible]	5.205866	1.9617018	2.8508203	2.9486063	2.7127686	2.141556	3.1329505	3.0869049	2.4341489	7.5061272	2.6771017	2.3426425	2.0337175	2.1549464	2.8070521	1.850233	3.1146571
NMRAL1	4.570829	20.450111	27.732155	13.407507	29.992324	4.0575294	12.80221	14.049848	15.635758	20.598445	15.515268	10.664318	9.8751396	11.304796	15.706685	19.342142	8.8598503
BIK	10.2455	4.5742081	14.103462	3.4784221	13.171093	2.7039012	15.839941	281.5441	22.715944	31.988856	34.62952	35.88499	25.305112	8.2647734	32.700136	27.165088	33.44622
SLC39A4	2.9361986	30.415375	43.763857	43.742796	86.350816	1.2877185	23.231924	26.789589	23.690153	50.902066	8.637002	5.7345916	12.257044	24.12765	17.995767	51.762038	2.0316695
HCG25	1.952613	0.2002157	0.1593175	0.3189092	0.2591605	0.3409842	0.3028441	0.1211494	0.24297	0.2876134	0.1214827	0.2834813	0.1981622	0.5059007	0.424599	0.3966648	0.3650057
KLHL11	4.5587523	0.9146497	0.8267396	0.4876764	0.4482836	4.0195025	0.7566653	2.5408962	0.9159906	1.1845239	0.8605531	0.6129407	2.5789476	0.9915647	1.7341207	0.7242503	6.3955398
ICA1	22.103309	15.437209	13.167256	9.8898789	4.9051999	10.829671	12.903225	11.694861	11.251764	7.9527881	21.602667	19.39155	23.985189	11.897807	12.007531	15.526293	15.534647
MCM3AP-AS1	2.122462	0.3506008	0.3396511	0.5185577	0.2224078	2.8099104	0.4134024	0.4541788	0.6145656	0.2598165	0.7211604	0.8297113	0.6239792	0.60313	0.4347045	0.6449911	0.6081669
MIR7705	280.93936	735.4786	298.42939	371.70193	284.76809	201.3317	327.71111	641.64931	510.28861	277.41306	352.79743	570.43555	375.48655	385.90931	381.14706	385.70994	140.82324
ZRANB3	1.3558846	0.5989694	0.3698404	0.2947056	0.2588122	2.1188283	0.6238953	0.4542703	0.3944278	0.52026	1.2112327	0.7200364	0.302978	0.9606829	0.7253818	0.637797	1.3969262
RABEPK	4.5520719	8.4309063	14.404684	8.5649886	16.189322	1.920948	7.3545129	12.113692	18.605022	18.591278	11.064817	8.612365	11.356698	12.355934	8.2909787	20.058223	12.009952
AP1S1	28.863955	53.886285	92.112483	133.09954	107.27137	17.470697	67.323474	83.391872	142.51685	70.210354	119.38158	101.05828	83.024493	105.99914	111.19582	142.36323	38.199507
DPH2	7.4904172	16.950678	15.823721	20.813446	22.209836	3.2158033	14.619788	12.406328	20.982278	15.760991	20.752759	13.233809	21.811193	26.913295	18.74974	21.422472	16.346402
LUC7L	9.1154007	3.5839835	7.9536383	4.185993	5.7440292	15.091957	6.8011276	6.9504019	4.1746737	7.0476599	4.2933944	4.6259832	6.3231852	6.2940415	7.3129544	8.3099864	7.2090392
OSER1	18.368571	57.550727	53.70266	46.315275	16.379566	19.597365	47.962574	71.160848	29.850075	55.66299	22.661418	24.00269	28.33167	27.089766	19.736288	49.768921	25.502289
GNA15	1.8683432	3.8710616	6.4506219	2.289371	5.1784499	0.3984457	5.0516119	9.6432341	6.8008437	0.8426031	9.2442848	3.5700749	1.8458302	2.9965548	5.2467955	7.4740879	5.587349
CABP4	0.6040819	0.0223574	0.3931684	0.5132695	0.1763503	0.3299964	0.2259199	0.0604547	0.0610461	0.0447341	1.7005384	0.1183119	0.5239611	0.4895018	0.2439818	1.1036627	0.1471849
WDR35	1.1337832	3.5970908	0.9218702	2.0933154	0.1022454	5.7497792	2.4869464	0.2788129	0.4784698	1.4336941	1.3937162	1.0214771	1.2657713	2.7336338	1.2346478	0.5216476	2.9049693
BCL2L1	46.344139	76.280162	77.718894	87.858148	26.311617	21.056823	75.664195	29.578666	31.143084	63.183282	26.781022	49.827988	35.058408	26.18546	27.227634	39.853921	10.509243
PTCD1	0.5682404	2.4278039	2.202221	1.8181658	3.1820828	0.3721555	1.748218	1.6452369	1.8093275	2.0422783	2.2140622	2.2175859	1.2261916	1.4525935	1.5158659	1.4083138	1.3247099
ALDOC	7.9311458	17.027301	10.661019	7.0726865	56.292203	1.3778729	10.366376	34.636375	6.9374765	24.679522	8.7709758	35.065945	6.9742532	5.2692413	17.002521	28.448372	6.6229593

[illegible]

LOC574538	0.0404319	0.0077813	0.0160967	0.0035027	0	0.0526406	0.0085005	0.019126	0	0	0.0219207	0.0071613	0	0	0	0	0.0204906	0	0.0171913	0.0036271	0	0.0144191	0.0399094	0
LOC730101	2.8510748	3.0498959	0.8211536	3.2097056	0.5712149	4.4793966	2.9059015	1.2050358	5.940469	4.4398168	4.1976447	3.1721646	6.5787275	0.9909633	2.0636852	1.0059365	1.7969637	3.3410121	1.1384134	2.5479977	2.6250082	1.3247285	4.3689667	0.7073831
SLC3A2	9.3348798	48.505801	45.970323	24.360572	78.740657	7.5501645	39.032981	32.569339	36.699976	57.14128	30.103689	33.981875	30.475612	31.370742	20.900061	42.014831	24.27307	40.631293	55.342909	47.331337	54.21269	41.944141	43.622389	32.890491
HSP28P	0.3918715	0.3881783	0.2982945	0.3195196	0.0233285	0.0954923	0.1575064	0.3925926	0.4710704	0.0591765	0.2624486	0.1428996	0.3669472	0.4126055	0.1520823	0.2856486	0.1022195	0.307007	0.2695338	0.240011	0.1194579	0.2740239	0.227534	0.1502262
NTG2	2.0855263	24.240595	7.5642943	10.499402	5.29789	3.0530603	18.656992	3.8786702	5.2602474	15.064624	6.8757307	9.1188204	6.003592	6.1638748	4.8439253	7.917	5.4718628	5.2759352	13.34165	13.914295	14.124481	10.378463	9.945624	3.4024552
CDCA8	10.046877	17.8358	11.951408	19.051358	19.902773	3.2469691	13.154929	23.672745	24.733574	13.742041	30.113297	20.153526	24.024714	32.857914	27.862481	22.736653	12.390681	21.668901	28.886179	7.587666	23.041822	8.9998381	21.891367	19.774371
RBM11	0.1304545	0.2151993	0.1484063	0.0968713	0	1.1911331	0.7150627	1.9484903	1.6974958	0.1196068	0.6971727	0.2558179	0.4120382	0.2944789	0.3708045	0.6158389	0.1062538	0.2688913	0.0990506	1.3141988	0.3283676	0.2658497	1.2187047	0.0202423
IPO5	18.077519	30.495764	14.939045	24.324906	12.969858	14.04369	22.73225	16.76469	23.149499	36.712001	23.209189	36.421042	46.988596	42.225674	23.310423	24.251802	29.353249	40.541306	28.57757	58.133157	40.687721	18.427175	32.055469	28.670079
[illegible]	3.9983462	0.1923751	1.1940124	0.6927766	0.4046438	0	0.2101554	0.1891583	0.4669103	0.5132219	0.4335514	0.177047	0	0.8423881	0.4419689	0	3.0395023	0	0.2125685	0.3784636	0	0.475307	0.98667	0
UNQ6494	0	0.0435417	0.0360333	0.0313603	0.0366344	0	0.1141585	0.214068	0.1690968	0	0.3434512	0.1041881	0.0480203	0.0571992	0.1600546	0.0747623	0.103193	0.0803325	0.2020143	0.0856605	0.1125559	0	0.1798563	0.0196592
LPCAT1	8.3439464	8.1399502	6.9978614	7.9676916	5.5402653	3.1423337	9.7822268	8.4011431	29.685902	8.7628645	9.8113203	8.4300429	2.6054722	4.9370638	19.105254	6.5322643	5.6033121	8.4413891	8.941457	5.2984319	6.6826387	8.0188836	8.6935041	41.23217
[illegible]	3.6220313	0	0	0	0.5236567	0	0.2719658	0		0.6641695	0.2935333	0.6873589		0.5450747	0		0	0.3277895	1.7226511	0.275011	0.2448882	0	0.2128112	0.5620228
NID2	5.3532301	2.2313348	3.7944887	4.3425889	5.0833697	0.6311658	9.2113962	2.94598	1.7074511	1.8988888	3.6223111	6.4287555	3.2007994	4.4287317	1.8634329	2.9700836	8.316786	1.8557306	2.2893283	1.5989376	5.291642	5.7706815	9.0076962	1.9954799
CDKN3	5.1962857	14.683796	6.5336844	6.3089464	3.0304208	4.3239005	7.982701	13.327621	15.217816	6.4572034	14.403305	11.78481	9.3215788	10.598674	15.093384	13.902496	20.714473	10.47536	21.631652	2.6983052	5.2636439	5.3821523	13.231733	16.236214
GRAMD2	2.0813529	1.5969887	2.0103718	0.6570751	1.0085668	0.49583	1.0429811	1.8024464	0.4788943	4.5960476	2.1660183	1.6753253	0.6005661	1.1241414	3.3827861	1.9853921	0.5139991	4.9529659	1.4952723	3.3266395	1.9104261	1.5463917	1.001113	0.6897092
FPGS	4.7998463	8.1878103	27.486686	11.21951	41.000682	1.5096641	14.125481	21.664899	24.909205	23.854177	17.943138	21.278516	16.148292	32.388334	26.247336	21.952284	20.333329	19.582767	30.934015	16.102754	26.70633	11.178963	17.127213	28.923229
RCN3	6.9345422	7.7345278	34.242847	14.982578	27.837871	2.3503964	35.08983	34.079213	3.8403537	17.59837	10.11689	21.391979	8.9760938	21.782153	8.4318298	35.501216	13.085738	12.524214	9.6050186	9.1596143	18.011903	25.230102	37.189085	9.1298497
LMOD3	0.0720802	0.0296931	0.1331024	0.663857	0	0.346965	0.1189374	0.0194644	0.048045	0.0528105	0.061342	0.0592089	0.1182538	0.2275294	0.125066	0	0.17593	0.0456633	0.0710681	0.3407588	0.0746249	0.116159	0.1099889	0.0223442
ARHGEF38	3.0696478	1.1615102	0.8029401	1.2719339	0.135077	8.2079466	1.3176653	1.2930847	0.9927773	1.5046563	2.4666495	1.5135076	2.5814609	1.3815654	1.943637	0.659189	2.735111	1.404074	1.3694307	2.3592176	0.842853	1.2279336	1.5871688	0.5294686
[illegible]	7.8773389	3.1583976	0.6534296	5.6869725	1.3286812	1.5539381	6.2105632	2.1739083	3.0662764	6.7408247	8.8975101	6.6855056	3.4832603	4.8405885	3.6281032	1.3557648	10.396308	15.299946	9.7690473	3.1067906	2.7215011	2.3410641	8.6394989	0
LINC01933	0.1056973	0.0056505	0	0.0101743	0.0237708	0.0556015	0	0.0055561	0	0	0	0.0052003	0	0.024743	0	0	0.0669583	0.0130345	0	0.0333493	0.0121723	0	0.028981	0
[illegible]	10.13349	37.725738	31.076047	21.931652	62.133353	2.6620019	21.525702	20.265977	20.520368	52.475078	19.560865	23.962283	15.425321	27.843064	21.762166	24.309765	17.500999	39.552939	37.100442	26.866115	33.610785	31.011196	19.611259	15.074281
CDC25A	3.418552	5.3198708	2.3607629	2.9199434	2.1514825	4.0082696	4.2393509	1.8295559	7.1108267	8.0766689	6.8750068	3.9830196	7.4164875	9.1379776	8.4786755	4.4127394	8.4730034	9.473519	5.9504444	3.2762794	5.3701225	2.1398626	4.4420523	6.5096083
SYN3	0.008079	0.824061	0.8444063	0.5004306	0.8176124	0.0956226	2.4543868	0.0152883	0.0047171	0.8296019	0.0744619	0.0500831	0.9288267	0.0936159	0.0535818	0.5839949	0.0665333	0.0986325	0.1288167	0.1911783	0.2177101	1.5462319	0.1827502	0.1842782
DDX55	6.4502547	5.5921435	2.666363	3.6575673	1.7137523	3.6897192	3.8474551	5.3675392	5.0694982	3.8532059	4.8158046	3.7593823	3.9345682	3.2595733	3.8372557	4.2604327	6.9679642	6.4498874	7.9119807	2.1784507	2.616718	4.1450027	5.2804288	3.0181431
[illegible]	4.5251692	9.7913102	6.5577641	13.034111	13.353899	1.4343694	9.8289164	8.8407649	7.7043958	12.477392	9.7296483	8.9312329	6.1055329	7.9787972	9.369897	7.5885471	6.8603746	6.438913	8.5240108	7.3218808	10.181996	10.70503	9.3878023	6.0563867
FAM173A	0.5717864	4.7426521	39.727974	3.1702726	15.947975	0.5414137	6.707925	21.683851	11.043897	12.917285	5.3196283	6.0359856	7.9087337	16.648446	13.753209	14.312731	7.6790032	6.5237793	4.6922853	8.5513482	26.398856	3.3442034	3.7626523	17.812002
TRIM28	26.897155	76.063045	95.223031	96.900298	307.10701	12.665809	86.82951	99.379357	80.955226	237.27924	100.7072	113.14329	123.42908	120.78998	119.25163	115.24538	78.938026	107.8898	92.564993	90.073207	158.27107	97.400222	88.314796	80.610705
FGF4	0	0	0	0.1001773	0	0.0563561	0.0071504	0.0193078	0.0079431	0.0174619	0	0.0120477	0.0120311	0.057323	0.0300752		0.008618	0	0.0216912	0.0064384	0.1409995	0.009086	0.0055951	0.0147764
HOXB4	14.72694	0.3999068	2.0975464	5.2788032	2.3457925	2.1892453	1.1383186	3.2288312	1.6131209	0.3305821	4.7635674	1.5602943	0.8903622	2.3554132	6.1466269	3.5541462	0.9492546	1.4032271	2.5136752	10.489085	0.5217357	0.7097344	1.386643	2.6320977
SETD6	3.447176	3.8836712	2.9054772	2.7537804	1.9196278	5.60755	3.5508734	2.614243	2.8168169	3.4575267	1.934674	2.7421712	3.7149974	3.5035914	4.9880347	2.1465831	2.7598737	3.0665031	2.4397884	3.1973391	3.3843243	3.8764942	3.1881965	4.8543364
DLX5	0.3249316	0.100502	0.2772377	0.0301605	0.0939542	0	0.1585869	0.0878413	0.0406545	0	0.0377499	0.2158198	0.1231548	0	0	0.0958694	0.1029207	0.0257594	0.0616779	0.153782	0.0240555	0.2759038	0.4399985	0.0252095
GTPBP3	2.1350393	7.8864462	4.5789796	4.1575006	4.9598333	1.3554098	4.6111772	4.513169	5.0770825	13.25408	5.2878538	4.3187432	3.3643847	4.232387	8.3459184	5.9729037	3.7809343	3.7375326	5.1321834	4.2531223	5.3611829	4.7299964	5.9072478	4.6064292
SLC2A8	1.5363827	8.5873356	14.812884	8.298593	15.343088	2.7830842	5.1083604	5.9858378	14.933095	62.476402	7.8189361	7.417223	7.892168	8.8699524	5.6191265	14.823327	8.5827767	44.265753	25.02439	5.2568669	14.61092	8.5852924	8.924742	5.0350261
CD46	40.395786	41.027267	36.550747	51.903355	8.7049608	111.23372	37.919683	38.513298	65.792632	39.371568	71.878272	59.693583	146.69234	88.863822	59.067921	51.697796	126.64331	80.284308	71.265999	149.46378	42.601274	49.960176	86.244495	25.449474
CCT6A	37.002896	109.99489	52.119654	102.83631	31.092537	41.661555	75.490564	68.012114	71.332781	61.085376	68.80364	79.013968	72.199148	76.81906	61.166417	77.354009	80.843672	112.08101	91.96372	73.844056	68.465355	74.411552	63.763436	93.951887
GPR180	3.6144651	3.3581693	0.8883135	3.7942911	0.4742708	5.6500235	2.6046694	2.0378146	5.0737194	1.651013	2.4498626	2.1016051	3.4169933	2.0797113	2.4633431	2.2961326	4.4278702	2.1192253	1.5076974	5.7029082	1.8188586	2.6224854	3.2396897	2.8699986
MGAT5	22.913815	17.049903	38.671544	54.0092	9.9472189	36.530272	19.088178	9.3388342	7.7567067	16.769157	49.052768	20.369691	36.030642	28.350809	26.461976	16.254795	38.892614	19.308264	28.417376	63.892753	14.938195	25.178957	44.585531	28.820212
GARS	34.723596	50.094909	23.471891	41.351358	12.714314	18.989491	45.103949	31.892664	38.612872	32.91814	57.735336	51.394369	39.692696	47.670404	27.141917	35.004332	31.619999	41.768052	40.07464	35.013351	45.167938	31.241816	53.173325	38.889487
HSPD1	57.171171	279.80724	79.402996	145.27053	32.01214	74.167324	158.53046	218.91158	219.62051	149.70725	168.8721	188.44347	160.90926	155.8676	164.66265	128.90401	161.96776	224.51043	157.40347	163.79761	99.836295	133.78239	134.89372	186.68756
[illegible]	0.0405923	0.2441309	0.2828456	0.3252885	0.4313462	0.0120113	1.7815206	0.5857184	0.3199639	0.2805187	0.3081076	0.4223964	0.251292	0.5772711	0.783224	0.3353438	0.1157171	0.2477874	0.5393617	0.4706781	0.5890097	0.6031814	0.6761444	0.5731751
BCL2L12	1.7664174	6.7036133	9.8169918	10.10369	9.6011608	1.4799578	10.244128	27.361366	12.897975	15.183586	15.409074	11.55679	11.88551	16.440545	17.134224	14.838772	6.6009048	9.3619128	12.858874	10.943165	14.87277	7.0191427	7.2323152	13.969412
EME2	2.6418368	1.1271306	3.1049968	2.0543149	3.0150621	0.6178417	1.6528941	1.1143061	1.2897644	2.6964762	2.5229315	2.5820313	1.199151	2.4749923	3.6450123	0.8808941	2.2059012	1.3565364	1.8913226	1.2714054	3.5958754	1.8351155	1.8929384	0.7121884
RCC1	14.966032	19.370612	17.172118	20.026466	36.313869	6.3330572	20.911221	27.957278	40.587448	16.915994	29.634933	20.597195	22.169351	23.082258	30.710476	14.765562	26.365026	23.339876	34.865495	10.498763	18.466204	17.696871	13.036327	38.630395
SLC22A14	0.1081815	0.0057833	0.0717909	0.0260336	0.0608238	0.0213406	0.0126357	0.0454931	0.021055	0.1234313	0.0521352	0.0053225	0.031891	0	0	0	0.0761468	0.0266817	0.0127772	0.0113777	0	0.0214336	0.0148311	0
LMCD1	9.5430142	1.1266168	2.1021655	1.645823	1.082031	2.4237454	3.8640732	2.9763685	0.5391395	1.1568758	1.1257965	3.9478543	2.3013544	4.053702	1.631134	4.0422335	2.8883669	0.9218537	1.2938363	1.2064834	2.092658	2.4500629	3.9830216	0.9405625
FLJ41278	0	0.0033303	0	0.0431895	0.0112118	0.0065563	0.0058229	0.0157235	0.0258741	0.0142203	0	0.0049056	0	0.0116704	0.012246	0	0.0140363	0.0122957	0.0176644	0.0052432	0	0.0131697	0.0091128	0.0120332
MCM8	12.413389	58.649323	10.412614	13.018242	15.778382	7.7543766	23.625239	9.1809942	29.631423	18.936714	29.677368	27.146513	17.977618	19.764149	27.397644	17.65077	27.706952	27.082391	36.166928	12.939528	24.35572	13.120743	15.027295	27.715285
XPO6	8.3365436	6.0521214	1.5010442	6.5826977	0.7969557	15.671614	4.2491425	3.075533	3.516946	2.3872149	4.8894275	6.2665227	9.5203585	5.6303618	3.7087506	1.963793	6.8203463	5.7879593	4.6239825	8.6354576	6.5969921	4.2673511	5.7505636	3.962796

[illegible]

KCTD14	1.4486976	11.395129	3.7104826	5.8803969	5.7162305	1.8495429	5.1418856	1.4246375	12.935073	8.1484895	8.2967365	5.766149	10.767769	9.5509106	10.74171	13.716205	7.1150453	4.7513369	8.1063105	5.5158756	8.1992522	8.2044103	6.7281257	5.0919016	[illegible]
ATP5L2	0.770645	0	0.2191762	0	0.0557082	0.651526	0	0	0.0642805	0.0706563	0	0.0243744	0	0.5798667	0.1216936	0	0.5928107	0.3054701	0	0.0521039	0.0570527	0	0.0226395	0.0597897	[illegible]
RELT	2.9636587	1.4761337	1.7243488	0.961307	2.8162493	0.4158296	1.7515168	3.903446	2.0675661	2.7414589	2.3611475	2.2333909	0.9536449	1.6855757	2.7914932	1.3649862	2.4856417	1.0114915	2.0447472	0.7603342	1.6872704	1.6622962	3.8827438	1.7606551	[illegible]
WFDC3	1.005821	1.9709451	0.8555848	1.2991363	0.4071676	1.3961213	1.268797	0.1816863	0.3203334	0.5868441	1.4872545	0.5911382	0.0323463	0.3082335	1.3139617	1.6240991	2.9310239	0.1826724	0.3207569	3.2370045	1.1751679	0.8261052	0.5415403	0.1787723	[illegible]
GCNA	1.0810221	0.1928533	0.205542	0.2314995	0.208971	2.1708357	0.3192087	0.7987378	0.1347476	1.7149917	0.5070672	0.2474062	0.3329988	0.2047229	0.3356572	0.3261174	1.1080207	0.2696168	0.4970856	0.229942	0.2517821	0.2526832	0.4645878	0.1847023	[illegible]
RPL13AP6	5.2461961	0.1037317	0.8584403	0.5603341	1.3818719	0.9761939	0.6043685	3.16191	0.377648	1.2914407	0.038963	1.1774203	1.2711244	7.797596	2.2242881	0.8905514	1.6844737	1.9940406	0.4201557	0.7142573	1.266254	0.0427155	0.5024709	1.7953356	[illegible]
TAL2	0	0.1612901	0.0667385	0.0580834	0.0678519	0	0.1057184	0.6660908	0.2348786	0.2581756	0.1090488	0.0690633	0.1185866	0	0	0.6923494	0.0849454	0	0.0712681	0.1586547	0.1389791	0.0797009	0.0275746	0.0729231	[illegible]
SERPINE2	1.563182	17.272218	14.156879	19.970606	6.6583502	11.850548	15.470332	11.04377	10.317084	1.4286629	16.502114	7.3072952	4.2610617	5.5080682	4.3744536	19.912252	6.2079439	7.2636457	4.7068403	11.698913	7.4343108	14.716019	15.002578	7.5921436	[illegible]
TGIF2-C20c	0	0.0230515	0	0.0622593	0.0969735	0	0.3525483	0.0906641	0.0839218	0	0.1298765	0.0648591	0	0.1009398	0	0	0.242807	0.0531744	0.025464	0.272098	0	0.085431	0.1182284	0	[illegible]
SLC9B2	2.3570452	1.8074098	1.5478761	0.9997177	0.2319135	5.1436971	2.2239559	2.687075	1.0656223	0.8509131	2.2984517	1.5293117	0.709313	1.284587	1.9812117	1.8255143	0.9280455	1.1626734	0.9656601	2.3743766	0.8906662	1.7852756	2.2383983	10.605121	[illegible]
MROH5	0.0092321	0	0	0.0159961	0	0	0.0048525	0.0131029	0.0107809	0	0.0100106	0.0204399	0	0	0	0	0	0.0102465	0.0961357	0.0043693	0.0478433	0.0109748	0.0673312	0	[illegible]
UTP6	8.4154596	18.564995	12.422829	10.218181	18.318307	5.6675249	12.712165	12.335742	13.321449	13.2565	11.353918	12.62235	20.232156	14.166089	10.894017	11.624353	9.8188822	9.0565177	12.504747	11.274298	17.78952	14.720627	10.141403	13.076107	[illegible]
UBD	2.8727768	8.0595797	1.6513327	6.7301945	2.7844856	1.6282755	15.46102	15.753907	7.4181603	7.4787807	8.0946878	16.214377	34.208799	15.600067	23.404066	3.1755079	18.583281	4.4907189	8.1503949	27.556118	3.6484889	18.325772	24.296107	34.587411	[illegible]
NPRL3	5.1042589	14.895173	13.632951	7.9041948	18.743171	2.7410819	11.221008	7.1467417	6.7376656	12.804533	6.8227437	9.0878225	6.3856258	9.2957129	9.60709	7.5318898	1.568271	5.4582586	6.5894033	9.3069744	11.5601	7.7250865	6.6208461	8.7101204	[illegible]
MYO7A	2.2370245	2.0128753	0.8113096	1.0985751	0.8599429	0.5747044	0.8362715	0.9414096	0.1290964	7.7516992	1.0765066	0.4549645	0.7169668	0.6713364	0.9344735	1.3072539	0.6399084	2.6030987	0.592175	1.3849641	1.8647446	1.0049615	1.7135024	0.6121589	[illegible]
LOC155060	0.6899511	0.247732	0.3382709	0.4951301	0.0729515	0.3839066	0.3680549	0.3556405	0.0781646	0.6212496	0.6811355	0.3647889	0.2732129	0.1844142	0.4439351	0.1063407	0.3587955	0.2971592	0.5254249	0.1705789	0.6190443	0.3978512	1.3849419	0.0559259	[illegible]
QTRT1	2.603005	18.582427	27.034112	14.702316	148.04065	2.0261224	13.122154	23.482646	12.592965	47.369033	22.858144	20.105414	16.726589	21.544752	16.186629	38.082365	18.146922	15.142338	17.998788	20.578819	31.444445	14.839139	15.093463	10.417904	[illegible]
APOC1P1	0	0.1127999	0.4667426	0.1015531	0.0474529	0.0277489	0.271096	0.1552792	0	0.0601859	0.1016858	0.1245746	0.0829348	0	0.05183	0.3873614	0.0891112	0	0.024921	0	0	0.1114792	0.1735614	0	[illegible]
MIR6832	0	0	0	0	0	2.1690386	0.6421416	0.5779835	0.3566676	0.784089	0	0	0	0	0	0	1.9348684	0.6779738	0	0	0	0	0.2512354	0	[illegible]
CYTOR	7.0630509	1.7022081	3.3570062	2.6901223	0.5022923	1.3179922	3.8060266	3.2933021	2.1028525	1.6335187	2.5735842	3.9671639	2.5773371	1.6757576	2.1382294	4.5207851	4.168029	2.5424018	1.8533004	1.2407385	1.9125711	2.2163112	3.9935965	4.1192238	[illegible]
RBBP8NL	0.3149431	5.8339324	5.1570961	5.6546995	9.2113335	0.4566397	4.8998643	4.656151	6.5004675	19.343689	7.4276529	9.7131792	2.4928237	3.8319159	8.1985001	10.14712	3.2221482	9.9096323	6.3189512	14.920381	6.7080178	3.3788828	5.0126349	0.7867917	[illegible]
TENM4	0.4174125	0.2902353	0.458392	0.4467714	0.2289315	0.1912451	1.8797149	0.188556	0.025158	0.0898735	0.3869098	0.4113968	0.254832	0.3092161	0.1369314	0.1835413	0.8120476	0.2211759	0.1345419	0.1325503	0.1897985	0.8179342	1.2781441	0.0468006	[illegible]
TBC1D30	3.98725	2.263433	0.8302491	2.1345129	1.1477698	1.2881802	1.6306	1.3401626	2.8625789	1.9192489	1.7398706	2.0402986	1.7541164	1.2215018	2.4004719	1.7331151	0.4800478	2.0320399	2.6462812	2.0964729	1.9134374	1.6958367	1.3426661	2.3587671	[illegible]
OR7A5	0	0.0109986	0	0	0	0.0135283	0.0480605	0	0.0133472	0.0293422	0.0123936	0	0.0606493	0.0240907	0	0	0.0868881	0	0.0242993	0.0432755	0.0236929	0	0.0094017	0	[illegible]
SERPINE3	0.5565183	0.0405699	0	0.1972341	0	0.2195653	0.0975031	0.0718047	0.0098467	0.2381129	0.0091431	0.0149349	0.2087997	0.0177651	0	0.1044895	0.1388832	0.0187171	0.0268895	0.0718326	0.0873948	0.0501186	0.0971033	0	[illegible]
TREX2	0.0161996	0.8339798	0.0645016	0.1333244	0.1147609	0.1246298	0.1958354	0.528807	0.0189172	0.1039676	0.553317	0.1865825	0.0429795	0.2559743	0.0895333	0.2007431	0.1026229	0.1078765	1.08485	0.1916712	0.2014813	0.308118	0.2065405	0.0703822	[illegible]
SNHG12	7.905525	1.4600457	3.2151474	4.3270333	2.2275181	2.8749235	3.7581539	17.578778	4.8135963	3.2598784	4.2931474	4.209912	2.5109318	6.3247708	7.2125876	2.7167487	8.9435954	4.0473652	5.89763	2.4717247	3.5096645	3.8241309	4.0870209	2.716083	[illegible]
SNHG11	0.9068414	7.8160515	4.1710493	7.6530517	2.468428	2.1281715	6.9112968	4.0904716	1.6523611	10.275384	3.6026534	3.4754833	3.0558499	3.969376	3.1627568	5.0697795	2.5950485	4.7373812	4.3128724	7.7771374	4.958782	9.6370893	4.7842799	1.0868838	[illegible]
NPIPB13	0.118367	0.0081358	0.3871397	0.0256362	0.3337026	0.0350248	0.0133317	0.0919974	0	0.5534746	0.0045839	0.0299503	0.1420668	0.3117257	0.5794364	0.506392	0	0.2158256	0.0404428	0.0320115	0.2190751	0.0251268	0.1077965	0.1010172	[illegible]
VASH2	0.2323494	0.3749685	0.2168303	0.2830655	0.0538872	0.8049745	0.539382	0.2587758	0.2261067	0.4162938	0.188957	0.2657848	0.3125069	0.3365473	0.1979764	0.1699555	0.2606514	0.4405412	0.2238286	0.4352789	0.1755976	0.2877161	0.6330934	0.0683508	[illegible]
DUS3L	1.3887721	16.913904	11.191544	8.6154822	32.790371	0.7932892	11.02537	7.5871053	9.3779476	31.373893	9.1727905	12.903235	7.0701321	12.237989	13.989615	13.218893	8.7365523	10.105942	14.467149	8.4930886	13.179889	8.1684411	8.2795939	9.2605679	[illegible]
RRH	1.0277536	0.0581753	0	0.0523749	0.1223665	0.805004	0.2224325	0.114405	0.1059972	0	0.0819427	0.040155	0.0534659	0.2547428	0.0668289	0	0.1914921	0.0335492	0.1927912	0.0715309	0.03133	0.0718677	0.0497291	0.2298306	[illegible]
CENPN	4.7581217	6.9094373	3.7056326	4.1861726	1.7465008	2.9982339	4.729675	7.9835598	6.7726852	5.1897549	6.0949939	5.3491214	4.5459085	9.1026554	7.2897624	4.1242912	7.3480616	5.404029	6.2829376	3.774559	6.6052463	2.9160508	4.69461	5.8242133	[illegible]
CD8B	0.1890691	0.1061856	0.2824545	0.1365689	0.2233518	0.1679256	0.3148565	2.4461755	0.5890768	0.0809382	0.1538408	0.1116856	0.6691855	0.232487	0.2788048	0	0.9187504	0.0699844	0.1675694	0.029843	0.4901628	0.2248764	0.2962408	0.0684902	[illegible]
SPATA25	0.1743004	3.2706312	1.2145172	2.5418588	0.4115926	1.3065807	3.0537776	0.7696267	0.2035408	3.5050953	1.511989	1.2348852	0.8734992	0.3060195	0.5137816	1.7999255	1.5826559	2.0634765	3.3350079	2.0348042	1.9871991	3.8332219	1.8877505	0.3155346	[illegible]
PSRC1	3.3737359	5.0846329	2.6528962	4.7578347	4.349744	0.6290592	2.866358	4.8975755	5.0370705	6.2089961	8.5525796	3.6835695	5.6675674	9.1364443	7.6288019	3.7543525	4.5769972	6.5313457	6.38642	1.5894145	4.758099	2.7378014	3.3896983	6.1743766	[illegible]
LINC00173	1.7038957	0.0204952	0.0636036	0.1014842	0.1939939	0.0504183	0.0559736	0.1914483	0.5471781	0.1093548	2.2632853	0.3489497	0.565081	0.044873	0.0706294	0.351908	0.0269851	0.0236388	0.4641227	0.070561	0.0220751	0.0632975	1.2964478	0.0694023	[illegible]
NTMT1	4.3996329	9.0751012	13.572085	10.038286	13.519299	1.2803701	8.4256436	13.58767	23.63685	9.8781234	11.210216	10.087996	7.7176428	10.018829	9.1487133	16.433892	15.454999	9.8788552	26.416581	11.565692	13.58976	7.9487203	7.7993297	11.323952	[illegible]
NKRF	3.2733714	7.9751116	3.2255829	3.2259792	0.9337934	9.6079093	4.809326	6.4646392	4.2586321	3.6799701	7.0332995	5.3649128	5.1777684	3.2399544	3.6061752	3.3575833	6.0957052	3.6817839	5.9257177	3.6910597	3.3813155	4.8836484	3.0765673	2.899246	[illegible]
LOC1005071[illegible]	[illegible]	0.3311895	0.036063	0.0627722	0	0.171522	0.1332946	0.188535	0.6557512	0.0465028	0.2749873	0.1283372	0.4805981	0.1526568	0.3203729	0	0.275408	0	0.2118083	0.0171462	0.3379459	0.064801	0.1639832	0.0393309	[illegible]
EIF5A2	1.5019713	2.0317537	1.7299268	3.0758763	0.334249	4.7607935	1.657421	4.0550821	1.4692688	0.6056257	2.2169802	2.4165505	2.3714777	2.1123715	2.4338715	1.7540343	4.139712	0.6720341	2.775187	2.0208859	0.9861974	2.0939681	1.9599328	1.7424415	[illegible]
RGL3	7.4682282	0.6943691	0.7333578	0.3803277	0.3881164	0.0597257	0.275835	0.0859416	0.6069405	0.3756718	4.9682324	0.5362598	0.3480862	0.9355573	0.423917	6.2322248	0.1726197	2.4082227	0.745584	6.2379622	0.5439255	0.7558231	1.0791939	1.6552461	[illegible]
VSIG1	1.7737423	0.0168437	0.0232319	1.1373191	0.0119097	0.0759653	0.1226697	0.1214548	0.0340675	0.194722	0.3163349	0.1136784	0.0309603	0.1352202	0.0386972	0.1205044	0.30309	0.025903	0.1674583	0.0331369	0.0967581	0.0277441	0.1535809	0.6717731	[illegible]
FRMPD3	0.2881534	0.0092427	0.066928	0.0020803	0.0729048	0.0170529	0.0227183	0.2135724	0.0588864	0.012329	1.8512806	0.0935693	0.0467199	0.1871871	0.164568	0.3868326	0.0395509	0.2345295	0.0357352	0.0181834	0.1393737	0.0627999	0.0928348	0.0260821	[illegible]
SDR16C5	10.207428	0.0580822	5.0469691	1.6210213	0.4751079	0.0476276	0.9729062	11.428527	28.93021	0.2754713	2.2979943	9.6514634	1.3522996	1.7238257	14.174277	3.8506366	1.0366504	0.133982	3.7783609	1.3711947	3.0445704	2.591068	0.4854613	14.816731	[illegible]
RBL1	3.6639196	9.1234404	1.5882731	4.6556476	0.2636525	10.784719	4.7795004	1.5516441	1.1904094	4.1909385	3.1036173	3.143372	3.3794753	2.0654553	2.4496095	2.1989126	5.6107934	3.3941961	1.2210137	4.4120184	2.6107715	3.3676224	3.417882	1.9015703	[illegible]
MTFR2	1.9746934	5.9597026	3.2701319	2.9704566	0.4965274	1.5298362	4.0476507	5.9874377	4.8215632	3.2490067	2.3650671	2.4244597	2.27928	5.2004573	3.9885442	3.4651455	2.410925	4.184067	4.1599623	2.1070381	3.9817227	2.4968165	3.49275	4.2312288	[illegible]
[illegible]	14.899709	3.8325619	1.2648594	4.7110534	0.3871709	5.0981584	2.5637786	2.7924201	0.2114078	1.7537871	2.7260268	4.9822309	5.4496052	1.0219092	3.2244923	1.2416261	1.4541266	1.0387595	2.8720665	5.4156504	2.1593944	5.0716389	1.9302748	1.1204664	[illegible]
GNMT	0.0820556	0.03948	0.2450399	0.2310333	0.9865109	0	0.1293867	0.0970495	0.1197764	0.1053254	0.200194	0.1635042	0.3265557	0.5186343	0.2267963	0.4236765	0.3898615	0.0910711	0.1308355	0.0194174	0.1700938	0.1650887	0.231153	0.0891286	[illegible]

[illegible]

LOC1001313	0.1518359	0.1948103	0.2519013	0.0986549	0.1280518	0.1198088	0.1729127	0.0478882	0	0	0.01372	0.3025486	0	0.0533157	0	0.1045296	0.7534631	0.0561727	0.0806994	0.0598835	0.3409706	0.1203309	0.1769345	0.0824682	[illegible]
GPR84	1.4639075	0.3625278	0.5785963	0.3170564	0.9586276	0	1.2786255	1.6295571	0.691338	0.4144964	0.291793	0.5433601	0.8948268	1.5194323	1.2612232	2.7566554	0.422773	0.5973338	0.2860491	0.1120756	0.4016312	0.9980748	2.5765466	0.5845807	[illegible]
TMEM97	15.221127	37.85174	12.005715	12.520899	12.117976	24.029573	20.617951	69.616347	31.359472	23.556783	32.27843	60.27417	19.076418	17.393344	38.217057	28.741856	12.590292	22.074148	35.295042	36.815683	24.956138	17.99947	23.663496	14.501633	[illegible]
CCDC175	0.3117355	0.2673688	0.0134917	0.0645809	0	2.0534011	0.1353543	0.0769457	0	0.2087682	0.1690116	0.162043	0.0719194	0.0656666	0.07491	0.0279927	0.0772755	0.2256431	0.0648331	0.1475367	0.0140476	0.1288968	0.2062531	0	[illegible]
TFAP4	2.5940713	6.2539644	3.9289438	3.6857972	3.4173609	3.004175	5.6095763	3.790138	2.9802846	2.8704414	3.6736413	3.6143061	3.5796362	3.6690517	4.6410391	2.3323911	3.5628928	3.2017393	4.2490894	4.1832064	4.5254858	5.8052458	3.7617292	5.3498256	[illegible]
ADGRF2	0.0412652	0.0529445	0	0.0148955	0	0.1831557	0	0.0032537	0.0120469	0	0.0894899	0.0030454	0	0	0	0	0.1263489	0.137397	0	0.003255	0	0.0735815	0.0026286	0	[illegible]
C2	1.8670024	9.9537447	5.353932	15.468904	2.8462483	1.7396191	15.137188	5.7506022	4.3372219	5.2416122	13.404382	7.3805822	5.4873723	7.1723202	11.43403	7.6298504	9.9701643	8.9410246	18.642477	7.3132482	8.9177582	22.505819	16.677385	2.6060559	[illegible]
ZP2	0.0636864	0.1041823	0.329653	0.7227709	0.7605382	0.1206068	0.0669479	0.0301295	0.007437	0.0490481	0.1173966	0.0056401	0.2476196	0.737974	0.0563181	0.4998229	0.1210343	0.2403243	0.0338487	0.0120565	0.4752578	0.0529954	0.0366703	0	[illegible]
PDCD1L	3.3193816	11.519654	7.0366908	6.4569592	7.6829601	1.9826013	5.3632956	18.611797	13.692445	7.2195363	7.2485795	8.9142105	10.682242	8.3208639	7.3383522	9.3311861	8.6837867	5.0826716	8.7557659	7.9276351	8.3461268	6.9602076	6.1181357	10.382301	[illegible]
LINC01759	2.4111854	0.4511538	0.2222358	1.0637814	0.4066979	0.158549	1.2438641	0.697101	3.6760296	0	0.5567944	0.3163479	0.2369324	0.3762952	0.8390679	1.1066344	0.4808688	0.5451322	0.261051	0.2324573	0.1851173	0.1592399	0.5509325	1.9884766	[illegible]
ANP32AP1	1.515202	0.0768129	0.0615276	0.0709548	0.2901078	1.066343	0.0645729	0.077495	0.0239107	0.1051292	0.0444047	0.2538663	0.0724328	0.776503	0.5432021	0.2537325	0.0778271	0.3181553	0.0217653	0.2325754	0.1697771	0.0243407	0.1010556	0.5782451	[illegible]
SLC9A7	2.1830899	1.8977037	0.4843646	0.246853	0.1685848	1.6499654	0.9308589	2.5425976	1.5024617	0.8496577	4.4895667	0.7201522	0.6280492	0.3140169	2.2532355	0.4345798	2.1522108	0.7639609	1.5377372	0.244815	0.3453076	0.7608322	1.8101571	3.256853	[illegible]
IRAK2	3.0811258	7.369685	8.0069335	6.076962	1.8274612	2.1148594	7.5663192	2.9630991	0.6783135	2.6419949	5.6070407	1.5488434	2.9482203	3.3521388	1.3120755	4.4857403	7.1275152	2.7329177	5.8657959	0.9143758	2.0609755	7.5956317	3.6718025	1.9613555	[illegible]
KCNN4	12.677031	35.541143	36.658976	59.384166	25.022958	10.572627	28.718898	18.042787	22.541303	34.071754	30.558849	25.639246	60.703938	63.827101	51.469427	14.250988	28.387325	1.3209049	23.91609	25.644389	22.730202	23.89252	27.823655	17.981654	[illegible]
FAM27E3	1.4632993	0.1328391	0.1099321	0.227229	0.0638245	0.0816964	0.1306051	0.7183976	0.8705107	0.1063172	0.164657	0.2200584	0.1220854	0.087253	0.1525944	0.1140442	0.2448647	0.1225715	0.3962023	0.091468	0.2575432	0.0820524	0.6586059	0.0299887	[illegible]
GRAMD1A	5.5291839	17.614206	13.417066	13.766745	12.839372	4.8622676	12.861851	12.51526	10.169167	24.920887	13.590338	7.9513225	9.976123	13.801159	9.1309504	16.968736	11.253627	14.261411	18.875418	11.956124	17.368891	16.630144	20.320388	2.2430782	[illegible]
PRSS33	0.6955858	0.196187	0.3024282	0.245891	0.1941937	0.2271162	0.1849032	0.4463323	0.0899968	0.3284023	0.1517154	0.0885074	0.0494955	0.168447	0.2829087	0.1616395	0.496232	0.0976105	0.2167193	0.0794629	0.2071675	0.3136456	0.4669389	0.0955265	[illegible]
C11orf94	3.6351192	13.396354	10.293944	7.2078881	10.164388	2.4758104	9.2464856	17.89881	5.8642399	13.515289	11.816477	7.5000559	6.5266147	12.032616	10.3399	10.387757	5.1019315	10.225401	11.334039	15.461458	10.052442	10.384111	9.5310147	5.8544676	[illegible]
LINC00630	1.9957406	0.7214657	0.322152	0.2429901	0.0545877	5.8926347	0.3515477	0.7451267	0.2897432	0.4015643	0.479597	0.4108081	0.3339157	0.3409223	0.3458133	0.6015645	3.0001138	0.3352453	0.4070864	0.2910187	0.2907073	0.3077779	0.439246	0.691328	[illegible]
MIR6814	0.6283116	0	2.5017403	0.5443245	0	3.3465167	0	0	0	0	0	0.5564334	0	0	0.6945226	0	0.3980301	0	0	0	1.3024327	0.3734555	0	0	[illegible]
PSPH	1.0674279	0.8771744	0.7240324	1.2234442	1.5391414	0.3410086	1.2760081	1.6177581	1.4836635	3.1282726	1.8437109	1.4054245	1.4619569	3.4330573	3.8216644	1.6192686	1.5199688	2.6315169	2.399849	0.5580353	1.9972872	0.4660172	1.2743075	1.1388929	[illegible]
EPHA1-AS1	0.9597234	0.0477681	0.1317696	0.0143351	0.016746	0.4455587	0.0652288	0.0391411	0	0.0106197	0.0089712	0.1648575	0.3438922	0.0261464	0.0182907	0	0.0157235	0.1377373	0.2374531	0.0274094	0.1715017	0.0098352	0.0340274	0.1707424	[illegible]
B3GNTL1	2.2466401	0.9151149	1.2452429	1.2496319	1.6148172	0.5779067	0.9359556	0.8907539	1.3527592	1.523802	1.875514	0.8648094	1.5240211	0.8471557	1.8413769	1.3050048	1.1523291	0.8571336	0.7225474	0.7461076	1.1047335	0.671472	1.1392548	0.4159507	[illegible]
CD55	635.67593	22.956006	68.822529	25.326657	6.2771671	4.2931563	32.076681	57.420443	129.45133	11.218304	17.701745	93.678633	35.61668	21.383699	41.10115	31.555292	21.439258	18.100697	15.347661	42.83702	55.981163	24.027645	19.872871	239.64882	[illegible]
PCSK4	0.220373	1.0379734	5.7727393	0.1456987	4.7656626	0.0480483	1.6886697	2.2223102	0.3318363	0.759276	0.5596609	0.4211402	0.3692697	0.3909818	2.1538993	1.4851917	0.5876861	0.4505522	1.5904513	0.5599105	2.3802369	0.7376533	0.4340967	0.302354	[illegible]
GCKR	0.2236837	0.0134526	0.0695811	0.0121115	0.0282968	0	0.0514366	0.0529114	0.0326511	0	0.0454774	0.0557141	0.0494551	0.0294541	0.0463604	0.0288736	0.0265691	0.0155162	0.0297215	0.013233	0.0724494	0.0997147	0.1954941	0	[illegible]
NXT1	5.9530142	44.338763	29.044024	16.150394	18.668385	5.2149637	16.818048	38.557627	26.297484	13.67373	20.044514	23.689297	28.644103	31.520169	23.63727	47.723403	21.609678	17.778248	10.990644	36.811804	38.684453	23.860579	22.534677	27.480563	[illegible]
RNVU1-3	7.1026523	0	0	0.8283199	0	3.8800194	0.7179222	0	0.1595035	0	0.5924305	0.3628913	0	0	0.9058991	1.1264005	3.2880745	0.9095798	0	0.5171552	1.1325501	0.3247439	0.2247075	0.2967281	[illegible]
1-Dec	0.0093439	0.0339656	0.0093011	0.0121424	0.0378252	0.2543678	0.0245561	0.0618873	0.0654686	0.5037339	0.0303956	0.0289624	0.4875479	0.1574892	0.0103286	0.4245596	0.5860099	0.4355625	0.0496621	1.1542054	0.1549527	0.0166615	0.011529	0.0507456	[illegible]
KRT81	1.0944151	0.0877606	0.6127902	0.138268	1.9613374	0	0.0599199	0.1078663	0.0399379	0.2633954	0.0865305	1.1004631	0.0806561	0.0960733	0.073609	0.6121675	0.1588819	0.0253054	3.5506215	0.0539541	0.9925225	0.1355204	0.0375095	0.1733559	[illegible]
CCNF	3.2346741	8.100246	5.2322813	4.6956509	8.029739	1.6461177	4.7487331	3.1793218	4.9414485	8.7424244	7.5893829	3.6579608	6.3378516	7.7475941	9.2470915	3.3547066	7.8167579	5.1529879	7.3992299	2.6988551	8.2514238	3.6546427	4.2376715	7.7007547	[illegible]
KLHL32	0.0639891	0.4207623	0.1486242	0.3187545	0.2266555	0.8709816	0.3307247	0.5903169	0.7347836	0.2874739	0.0520388	0.1641735	0.235793	0.2920987	0.2121985	0.2863412	0.4526579	0.2248953	0.2947499	0.3331287	0.3316067	0.6085404	0.5080066	0.2548455	[illegible]
ATAD2	8.143864	16.178645	2.6816912	6.0488998	7.4441399	7.6132707	7.1288915	6.6239935	3.9469939	2.6331642	7.8423589	9.4694518	9.5628984	10.798497	5.361621	5.1173549	12.889844	11.063898	16.623901	6.7172235	4.1115007	4.2419043	6.472683	7.1745156	[illegible]
MPP5	3.4178693	0.7444845	0.9553517	0.9756874	0.515378	0.9562896	0.7875564	1.3899404	0.4117044	0.5656754	0.9132479	0.6721557	0.4157265	0.3713936	1.6779271	0.9708616	1.0368658	0.4456421	0.6870682	0.4773964	0.4060133	0.5529902	1.1157068	0.8190691	[illegible]
NME1	13.406904	25.199015	20.935156	20.093142	25.090742	5.7311073	20.64939	21.963375	58.956588	22.84121	22.139117	16.308612	27.661929	16.184976	20.150853	44.097058	13.623094	12.934747	27.251526	21.62075	32.127051	14.743779	16.999826	24.400094	[illegible]
PPP1R3A?	1.6855087	0.09156	0.1127549	0.0451407	0.1604902	0.4156188	0.0714446	0.0986031	0.0502649	0.4071091	0.1572165	0.086271	0.088155	0.0620488	0.5008405	0.3089077	0.5748621	0.0905176	0.1661625	0.0879196	0.0469611	0.0996446	0.5870011	0.0393711	[illegible]
RNASEH2A	5.0690167	39.977042	24.735205	20.733184	33.421318	0.8169797	16.768115	16.939822	23.513621	43.219877	25.657993	18.176187	21.63243	29.697526	33.356842	38.401589	18.378877	15.561145	24.465054	15.609071	37.60383	15.359478	16.057382	24.483411	[illegible]
BTBD19	9.3961137	0.5170081	2.6616526	1.1311744	0.6069657	3.1870079	1.8191579	0.9930808	0.4231374	1.0745583	0.6571013	1.0622819	0.3094032	0.5528172	1.0082416	1.6257623	0.7757063	1.8721322	0.7836251	1.4783734	0.556864	1.9977746	3.9261246	1.017868	[illegible]
FOXP3	1.6449562	1.7507808	2.2824718	1.3315078	2.3373646	1.0423185	4.7072591	4.197641	0.931351	0.7891247	0.8827629	2.1189454	1.0139222	1.2952473	2.3325675	1.166767	0.642081	1.4199799	1.9163498	0.1887314	1.9115795	4.4047146	2.9043461	1.3355208	[illegible]
RBM12B-AS1	6.6679924	0.8101556	0.1340902	0.7293782	0.2044908	3.0293464	0.6018233	0.7647439	0.5112417	0.4322696	0.1825629	0.596483	0.4765257	0.3547576	0.3722556	0	3.2000886	0.598029	0.5011686	0.6375344	0.6980879	0.6005027	0.8310391	0.2194729	[illegible]
NUP62?	2.5640793	6.8418482	1.6017239	3.6890047	0.5426149	2.8335567	3.6580295	6.7645737	7.1647533	2.6363301	7.5591926	7.6348221	6.5945722	5.691507	5.914591	3.3502956	7.0691182	5.9819796	10.457289	3.702459	2.8202587	1.9750262	4.5625908	4.2201861	[illegible]
NOTCH3	8.3571991	8.4991961	12.326943	10.162482	18.886537	3.4634425	24.273689	5.7655612	3.124581	8.4735697	6.9880972	20.696254	6.5627852	10.332571	5.5996533	9.8920027	8.7703049	18.722297	5.8726002	5.5484357	13.291218	15.550441	24.239271	3.9157326	[illegible]
POC1B-AS1	4.1624371	0.2526404	0.3811257	0.4857018	0.0332129	2.971537	0.3766123	0.3648607	0.5365316	0.308916	0.4448202	0.1986026	0.5853084	0.2938563	0.5804243	0.338899	1.5315322	0.3399565	0.3546653	0.2226256	0.2891235	0.2860945	0.2879475	0.1544672	[illegible]
IL17A	0	0.0227663	0	0.0307445	0.0239434	0	0.3108808	0	0.0966974	0.0303682	0.2052314	0.0104762	0.1464631	0.1993818	0.052304	0.4397686	0.0749384	0.2888409	0.0754468	0.0111971	0.0980853	0	0.0973047	0.0256977	[illegible]
MIR4435-2HG	9.5725113	1.336894	1.9932983	1.7303116	0.4451082	1.8250546	3.0514567	2.036678	1.1691982	1.3814725	1.6775889	2.93044	1.8761796	3.1232779	0.8007437	3.3769708	3.5565157	1.4127379	1.5483119	1.4056583	2.0754647	1.6853826	3.0283032	2.4785286	[illegible]
CDCA7	13.572424	67.249141	24.728055	23.235489	9.911408	43.158024	38.06623	9.4305053	32.566813	41.271702	68.486151	70.019517	36.926845	51.901944	54.993339	31.910443	54.838911	80.194617	52.741433	13.429012	34.6118	16.925108	64.488443	17.51071	[illegible]
SNORD3A	10.648227	0.6682504	0.4608469	1.2032437	3.2797447	12.053286	1.2166893	1.0951267	1.8922153	2.9712846	1.0040138	0.4100035	0.4094359	0.9753968	2.5587676	0.956171	13.491124	1.5414984	1.2303124	0	1.4395308	0.8255331	1.3326701	2.01145	[illegible]
SCLY	2.069203	0.4689277	0.3171686	0.3442332	0.3641898	1.2001533	0.3842013	0.5444366	1.0374457	0.6736229	0.5580907	0.6373873	0.232059	0.3593414	0.5593828	0.5187103	0.2944602	0.4410037	0.5000733	0.4417505	0.5089622	0.3252974	0.4455559	0.3623713	[illegible]
THY1	7.2375128	13.925362	38.231236	27.43074	22.83762	2.956482	59.45288	20.751219	11.069516	8.3752614	21.531224	39.009582	15.053073	33.663536	11.085402	30.202892	13.702983	12.967665	17.414578	13.803222	17.387751	40.984295	48.139507	5.0714851	[illegible]

[illegible]

LOC1005060	0.4967537	0.0183851	0.0190184	0.008276	0	0.3392067	0.0502109	0.0451942	0.0111555
SLC39A10	7.916344	5.4884064	3.4811696	6.1558168	2.1557214	14.1149	4.6421349	3.3841743	9.5005473
SNORD1B	3.1415578	0.2519198	0.5211959	1.1340094	0.5298907	3.7183519	0.2752035	0	0.3057151
LDLRAD1	0.2962985	0.0980101	0.0553016	0.0401081	0.2436382	0.0767155	0	0.1051322	0.2703165
LINC01649	0.1890336	0	0	0	0.0318845	0.1305152	0	0	0.0367909
S100A5	0.6731909	0.4588539	0.4467393	0.5103042	1.0787061	0	0.1769166	1.5924037	2.849701
CRTP18	0.8177821	7.1932558	8.1551827	3.545562	15.589321	0.6115545	4.5523018	4.5546979	3.8719773
LCN1	0	0.0541902	0	0.0731806	0	0	0.0295994	0.053284	0.164405
ALMS1-IT1	2.9223793	0.733162	0.1662286	0.1265871	0.0211252	4.5460249	0.1316588	0.6221484	0.0853158
LOC1053764	0	0.1550276	0	0	0	0	0	0	0
PRELID3A	0.7821207	1.7214044	1.4411273	0.807322	4.1260504	0.5120999	1.1108102	3.5190735	1.2242483
SLC2A12	1.9884345	3.2963831	1.2248337	2.6948205	0.2889019	0.6291571	2.0799178	0.3958437	0.1839217
FBXO41	2.6737162	3.1407743	1.6231176	2.5065699	1.7613195	2.2613689	2.65193	2.6960541	0.0609064
RNF224	0.1223076	0.141232	0.2191458	0.1589379	0.3218246	0.1158107	0.0899998	0.0462901	0.1142606
SOD3	62.658709	59.165495	50.411497	75.411423	188.14447	151.48764	47.29752	37.685996	59.648231
BRDT	0.0202962	0.0048826	0.0202033	0.0175832	0	0	0.0053339	0.004801	0.0059253
NECTIN4	14.367558	3.5953603	2.9878723	3.6829724	9.5325859	0.1337842	1.3598292	3.8323181	5.2210754
KSPH14	0.1624944	0.3214149	0.5032236	0.3753962	0.3471698	0.0961643	0.170816	0.1708326	0.0210838
JAG2	1.1069362	4.7238213	12.770725	6.6204734	22.231057	0.4312986	5.384473	6.1475693	1.5896992
LOC1019295	7.0243179	0.0384052	0	0.8298232	0.0807819	5.9520624	0.3775932	0.2265779	0.1398189
ZNF695	0.7260043	0.2760655	0.1049052	0.2789736	0.0118506	0.5613167	0.1230942	0.4542618	0.3418539
NR5A1	0.0128527	0	0	0.0055673	0.0260145	0.0380311	0.0067554	0.1033682	0
SYNPR	0.6559787	0.0250488	0.1140116	0.0270616	0.0105376	1.5035381	0.0437824	0.004926	0
HSF4	0.9295609	1.4472676	3.0774197	1.1536681	3.0336276	0.7540899	2.5472175	2.016011	1.6282539
MIR6719	0	0	0	0.2189811	0.5116186	0.8975332	0.2657138	1.6741592	0
SLC24A2	0.2946032	0.005906	0.0448028	0.0141791	0.0082819	0.0387436	0.167749	0.2303546	0
BMP4	13.928048	24.353992	34.220349	14.767584	79.316492	4.7293769	17.97913	16.837662	51.218481
KIAA2012	0.0808914	0.0555997	0.0287575	0.0225253	0	0.1333566	0.042517	0.0191345	0.0573517
SLC16A1-AS	2.1686733	0.4848479	0.3097129	0.883069	0.061096	2.5750892	0.4491126	0.4152254	0.0515174
IQGAP3	12.042483	11.120492	8.5456954	10.05975	7.0245496	5.2968039	5.2289487	6.8360143	6.981839
COL8A1	7.0918796	5.4297715	4.6395741	11.755222	1.0597615	5.2455321	15.118994	4.0163959	0.7861244
TRPV4	0.1872064	0.4953954	1.2345646	0.6588658	1.5748708	0.0207729	1.3406563	0.6919197	0.3689076
BFSP1	0.3953727	0.7477942	2.3206627	1.8602535	0.1517728	0.7503556	0.4657815	0.6449909	0.8676773
LINC01091	1.6622512	0.0843295	0.0956765	0.2375603	0.1831015	3.4530626	0.1842473	0.5670614	0.2872047
PYDC1	0.0510822	0	0.0508484	0	0	0	0	0.4349981	0
PTPRG-AS1	0.4108621	0.1553207	0.0389506	0.0593236	0	1.0941681	0.1131175	0.004626	0.0399823
MET	43.475696	38.567673	32.721831	30.088641	3.4905488	83.140214	20.240185	16.1369	15.067292
MIR548AA1	0	0.2181574	0	1.1784345	0	4.8300241	0.2383206	1.0725468	0
THEG	0.0424945	0.0102226	0.02115	0	0.0430056	0	0.0223354	0	0.0124058
NPIPA3	0.4221289	0.0870436	0.1200561	0.087072	0.2849042	0.1189601	0.1690464	0.0380391	0
FZD2	1.8341787	2.5240469	2.3747928	4.3169743	0.4283631	6.6873946	10.038308	4.0109934	1.4753493
SLCO5A1	0.4181617	0.33038	0.1314465	0.0991464	0.2004591	0.9534045	0.9138564	0.2228175	0.6630761
XKR9	4.6741231	0.0325141	0.4933009	0.7610786	0.0113984	1.3397493	0.1183974	2.6055883	10.567955
MIR6761	0.6108585	0	0	0.2646022	0	0	0	1.4449589	0
PGF	0.7524563	2.3106311	1.8431557	1.6296868	0.8958904	0.0698516	2.7064296	11.251775	1.5936974
TNNT2	0.1090608	0.0466428	0.0361872	0.098609	0.0429227	0.1039848	0.0509538	0.7194737	0.0389145
KRT42P	0.041679	0	0.0103721	0	0.115996	0	0	0.059154	0.0182516
CD8T	0	0	0	0	0.0980145	0	0	0.0014318	0.0441785
FAM83C-AS1	1.2494832	0.3005861	0.1243763	0.4329854	0	0.2218335	0.3283678	0.9457913	0.1459095
[illegible]	[illegible]	[illegible]	[illegible]	[illegible]	[illegible]	[illegible]	[illegible]	[illegible]	[illegible]

SPAG17	0.2164097	0.0088615	0.0137501	0.0179504	[illegible]
NDUFA4L2	1.0188231	1.7496127	14.822321	2.8040666	[illegible]
LINC01555	0.0152503	0.4035609	0.0607219	0.1321176	[illegible]
HCRT	0	0.403421	0.455256	0.0990539	[illegible]
DUSP13	0.0187077	0	0.0186221	0	[illegible]
CCL26	0.0622972	0.3297081	1.7983473	0.377789	[illegible]
MIR6851	0	0	0	0.2843486	[illegible]
C2orf78	0.0288879	0.0069495	0	0	[illegible]
MIR5165	2.3456965	0.2821502	0	0	[illegible]
SLC7A11	17.743206	1.2023196	1.2573547	2.6962264	[illegible]
SLC11A1	6.2150412	0.4414694	4.1681253	0.56873	[illegible]
SPACA3	0.0357576	1.8408579	0.5695018	0.9912902	[illegible]
MIR1204	12.979157	2.8084036	1.5701346	1.7688317	[illegible]
SNORD46	15.224472	0.6104211	0.8419318	2.1982336	[illegible]
SNORD88A	0.4833166	0.2325414	0	0.2093556	[illegible]
SBF1P1	0	0	0.0077749	0	[illegible]
NXF4	0	0	0.0242082	0.0052672	[illegible]
LRRN4	0.016338	0.1336335	0.0162632	0.0849243	[illegible]
RDH16	0.3174207	0.1099604	0.0884709	0.1759941	[illegible]
CASP14	0	0	0	0	[illegible]
EFNB	0.5172302	0.0082953	0.1029725	0.0746819	[illegible]
MYADML2	0	0.4218478	0.7658908	0.031003	[illegible]
SPTBN5	0.3422154	0.0874716	0.2270991	0.3999272	[illegible]
LOC1019283	0.0151296	0	0	0	[illegible]
XKRX	2.2303942	4.8070961	3.5185359	0.3333423	[illegible]
KRT7	1.2357459	0.5893926	0.9198923	1.3265666	[illegible]
LMLN-AS1	0	0.0390429	0	0.0351501	[illegible]
SNORA26	14.420265	0.346906	0.3588362	3.4354908	[illegible]
FUT1	1.3063904	1.5397097	4.2439722	0.5834286	[illegible]
LINC01929	0.0138744	0.0600793	0.0414326	0.0901484	[illegible]
C1QTNF8	0.249613	0	0.024847	0.032437	[illegible]
CCDC187	0.1097031	0.1011659	0.0819007	0.095039	[illegible]
MIR652	14.029154	4.367868	5.6298964	3.1791904	[illegible]
KCNK9	0.0692004	0.0665897	0.0984052	0.0685147	[illegible]
SIX2	0.0797856	0.0863725	0.6155075	0.1641614	[illegible]
PRDM12	0.2309223	0.1880242	0.1591374	0.0769441	[illegible]
WISP1	3.7071035	1.3982729	1.8564553	2.6812655	[illegible]
TH	0	0.091353	0.4184996	0.1762383	[illegible]
MAS1	0.0312847	0.0064509	0	0.0019359	[illegible]
SIX4	1.7043116	0.0476748	0.2169953	0.0858427	[illegible]
COL1A1	464.69139	166.00137	604.85157	464.35138	[illegible]
TMEM74B	3.8409453	1.3213821	1.485762	1.8965153	[illegible]
MATN3	0.2821155	0.7940564	1.9798089	1.5580809	[illegible]
DNASE1L2	0.0400928	0.0578704	0.6385481	0.1042007	[illegible]
MIR17HG	3.0228047	0.9695883	0.3008966	0.5728501	[illegible]
LINC02154	0.0847434	0.1087284	0.1968293	0.0367076	[illegible]
LHX3	0	0	0	0	[illegible]
FJX1	1.113009	1.1228426	2.8412622	1.6485257	[illegible]
SPATA21	0.2342573	0	0.009716	0	[illegible]
CYP26A1	0	0.6841743	0.0294619	0.0064103	[illegible]
PDCL2	0	0	0	0	[illegible]
CYP2S1	0.2909147	0.2247214	0.7361315	0.4046306	[illegible]
PGK2	0	0.016875	0.0698253	0.0151925	[illegible]
EDAR	3.5078126	0.7076066	0.5934992	4.0848935	[illegible]
BRSK2	0.2548562	2.8601273	0.6215391	0.3808616	[illegible]
PCSK9	5.2361398	16.449792	5.0715363	2.0309719	[illegible]
PENK	1.2716703	0.5253234	0.4603085	0.4896377	[illegible]
TAS2R38	0.1154378	0.1481103	0.1149093	0.3666926	[illegible]
SNORD105	0.517433	0	0	0	[illegible]
C17orf99	0	0	0	0.0137903	[illegible]

[illegible]

C10orf62	0.2548615	0.0175176	0	0	0	0.0646402	0	0.0172247	0	0.1869351	0	0	0	0	0	0	0
KRT74	0	0	0	0	0.0744826	0	0	0	0	0	0.0478822	0	0	0	0	0	0
TNNI3	0	0.5513997	1.0116426	0.187329	0.0656502	0.0895768	1.0910724	7.0585601	0.0126254	0.1665322	1.3364638	0.4500062	1.4916026	0.1594491	3.6809016	1.5184032	0.2054727
FEZF2	0	0	0.1397461	0		0		0	0.02342	0.102972	0	0.0088806		0	0.0886759	0.041421	0.1016401
TNNT1	1.2560443	0.0097472	0.5646489	0.1228554	0.1230147	0.2757506	0.1277776	15.114363	0.4649759	0.7021046	5.4039157	0.4126475	0.2687453	0.1493871	20.131879	0.4184074	0.2695091
CCAT2	0.5020754	0.1932536	0	0.2718516	0.0254057	0.9508114	0.1055575		0.0596303	0.0644457	0.0816621	0.0444639	0.155408	0.0526897	0		0.8587635
TDGF1P3	0	0.0078086	0.0323103	0.01406	0.0656986	0.1728828	0.0065303	0	0	0.0208329	0.017598	0.0071864	0.1004704	0.4957967	0.0538191	0.1005567	0
LINC01704	0	0.0253732	0.1049891	0.0685301	0.1601109	0	0.2771834	0.0498979	0.1231658	0	0.0285915	0	0.0466384	0.0555532	0	0.1089164	0.0668156
CST5	0	0.0279911	0.6370172	0.2772023	0.8242745	0	0.3057617	0.0825691	0	0	0.1261658	0.4121729	0	0		0.2403075	0.2211278
PRR36	9.7912823	1.657379	5.669058	0.4788492	7.1520978	0.2102838	0.695173	1.438214	0.2017061	0.1900395	2.777317	0.7998106	0.2094959	0.3535157	2.7930533	5.7893834	0.2751195
CTB-113P19	3.3924809	0.0096583	0.7193302	0.0434764	0.3250448	0.5464671	0.0316526	0	0.0117207	0.103066	0.0108833	0.0444436	0.0532584	0.7401174	0.3106491	0.1243764	0.4196483
LOC1019295	1.9403739	0.0388994	0.724309	1.0856472	0	1.6746254	1.3173345	0.0764978	0	0	0.4383332	0.0357999	0	0.2555038	0	0.1669784	1.0243421
ST8SIA6-AS	0	8.7910294	0.0216521	0.0188441	0.0220133	6.30759	0.0342984	0.020581	0.0127003	0.1396004	0	0	0	0.0229136	0.0480876	0.1796958	0
MSX2	2.7512649	1.7991735	7.058875	1.9554918	6.8825102	0.6765107	4.2873558	1.2557775	13.282042	3.5563789	3.5820513	4.7272325	3.9410456	5.9189827	6.1252612	5.2020754	3.9154235
CLDN1	9.3294746	41.294216	15.729932	31.050125	6.3795198	63.674873	25.830815	19.199847	16.061433	16.259732	20.922435	22.729005	57.181859	18.264391	26.1996	15.994915	13.56975
MC2R	0	0	0.0117248	0.0204085		0	0.006191	0.0111448	0		0	0.0104313		0.012408		0.0243268	0
EPHA4	2.5986586	7.1446292	2.8590523	7.2532269	0.4547974	0.6126425	2.4236494	3.3739244	3.7989611	1.1787459	7.5953352	3.4434105	13.426258	0.9262153	6.6521136	2.5019311	6.1393176
DBN	0.3167535	0.3556041	1.1210786	1.3263282	0.7954721	0.3263104	0.696781	0.7328161	0.5068884	0.6776336	0.5024755	1.6623243	0.7573853	0.6302744	1.3745953	0.4845892	0.178365
LINC01729	0.6142711	0	0		0	0.1454104	0	0	0	0	0		0.1086492	0	0	0	0.2334813
DCAF8L2	0	0	0.0096405	0	0.030014	0.029252	0	0	0	0	0	0.0043774	0	0	0	0	0
MMP8	1.7270344	0.0307755	0.3438256	0.0166242	0.0647336	0	0.1680999	0.2602439	0.0821643	0.0328414	0	0.9686602	0.3167828	0.0673813	0.0707047	0.1585275	0.1377707
C6orf223	6.6360094	13.090054	4.202566	3.0398516	20.249753	4.3856997	5.8545212	7.7662574	9.5259469	32.630272	15.907031	17.078684	16.136497	5.7603692	18.679591	21.056754	5.7758563
RXFP3	0	0.0226523	0	0.0411476	0	0	0.0249645	0	0	0	0.0126754	0.042063	0.0210024	0	0.0525017	0	0.0150443
LOC1019274	1.1726482	0.2257201	0.3891596	0.406429	0.2176085	6.3625133	0.4623419	2.1639704	1.449497	0.3010902	0.688965	1.9128943	1.9880385	1.5443782	4.1270078	1.9782115	1.4240631
LINC01594	1.799583	0.1039015	0.5015764	0.1247225	1.3112844	1.1075942	0.0378349	1.055695	0.8826209	0.0923967	2.107443	1.4343414	1.591506	0.6824585	0.1591378	0.7433408	3.1008562
KRTAP10-3	0.0905907	0	0	0	0.1833607	0.0268058	0	0	0	0	0.0245575	0	0	0	0.0500686	0	0
LAMC2	4.306405	8.0840783	7.4349472	11.112782	29.892982	2.4399073	7.6438553	8.4699175	2.6545685	89.294017	12.416411	1.4129657	14.43475	47.848457	12.111226	13.822272	3.9803807
EVX1-AS	0	0.0777416	1.1580429	0.2239689	1.1119529	0	0.0169854	0.1375949	0	0	2.6631078	1.9747048	0.1143171	0.238295	0	0.6674228	0.6346255
MMP11	0.7307642	38.606224	97.499478	21.635217	101.57686	1.9813011	152.85153	14.178464	2.103622	15.94597	30.820177	29.735753	25.986046	51.048623	6.3400684	15.426015	68.454866
MMP13	0.3103868	0.0074669	0.0926897	0.2083952	0.07853	0.0734748	6.6398435	0.7268643	0.4349482	0.0199204	0.8329678	0.1236865	0.3568478	0.1798323	0.1029285	1.4744062	0.0589682
KRTAP5-2	0	0	0	0.0271387	0.0634057	0.0370776	0.0658607	0	0	0.0804194	0	0	0	0	0	0	0
CLDN2	16.676973	1.1790733	32.418078	25.453955	46.351298	13.693567	4.1081995	1.0312176	95.166855	10.959771	0.0769204	43.028129	27.090606	75.054065	20.915111	6.8992921	27.028693
PAEP	1.2704511	0.0291077	0.1806621	0.0524109	0.0612253	0.0179013	0.0953938	0.1144837	0.10597	0.038827	0.0819991	0.2946724	0.0267513	0.127459	0.2674915	2.4989337	0.0383248
ADAM2	0	0	0	0	0.0306443	0.090639	0	0.0071626	0.0618797	0	0	0	0.0535579	0	0	0.1250757	0
MAP3K20-AS	1.2693995	0.229033	4.3651224	0.1124711	0.058394	3.7305472	0.1213098	0.1160138	0.1263368	0.12961	0.3832145	0.2235588	0.0765426	0.1539559	0.2232313	0	30.822853
OPCC1	0.0548972	0	0	0	0	0.4440054	0	0.0043286	0.0373956	0	0	0.1215426	0.0404581	0	0.0505685	0.0377933	0.0231846
LINC00967	0	0.0105648	0.0218574	0	0	0.0389842	0	0.0415525	0	0	0	0.029069	0	0	0	0	0
MIR3661	0	0	0.9120928	0	0	0	0	0	0	0	0	0	0	3.8609456	2.025691	0	0
LINC00348	0	0	0	0	0	0	0	0.0270887	0	0	0	0	0	0.0977455	0	0	0
KRT16	0.746338	0.3078002	0.8844536	0.0307901	6.348412	0.1367152	0.4296511	0.9584018	0.5914197	0.0456197	0.3179377	0.4878628	0.11001	0.0187197	1.7285896	1.584762	0.1463464
KRT83	0	0.045144	0.0466992	0.0203214	0.8071296	0	0.0493165	0	0	0.0903271	0.0127175	0.0519338	0.1037238	0.1235503	0.0259288	0	0.0148598
TACSTD2	51.483597	2.6372821	4.7858685	1.1750093	5.1875648	0.6089177	3.2448498	36.950628	0.3495372	2.9295779	33.217119	72.640473	58.88231	8.4749448	2.2747019	34.000805	30.125679
MIR566	0	0	0	0	0.4735194	0	0.2459266	0.2213554	0	1.2011576	0	0	0.4137916	0	0	0	1.4820268
PD[illegible]	14.174065	11.972496	4.3762949	10.580702	7.2790597	0.2914022	6.6550617	2.8453153	7.6804032	5.9592017	11.24096	9.4299996	0.590989	6.0762423	8.8641192	4.0678364	0.3007904
PTTG3P	0.5806179	0	0.2889799	0	0.367251	0.8590252	0	0.0343357	0.0647527	0.0931591	0	0.0642745	0.0641855	1.6055417	2.3265362	0	0.3218395
SPRR3	1.5871296	0	0.030382	0	0.1235554	0	0	0	0.071264	0	0	3.7706953	0	0.0321522	1.2145711	0.5673325	0
AKAP4	0	0.0626073	0	0.0062628	0	0.0342255	0	0.1983612	0.0253255	0	0.0078387	0.0256064	0	0	0.0479453	0	0
SALL4	1.7653373	2.4855757	1.2721235	0.6051773	0.6137912	1.3716058	2.3168329	0.4150209	0.0505887	1.5986843	1.4562032	0.2301916	0.3448093	0.4905936	0.1077442	0.7605103	1.873025
EIF3IP1	0	0	0.5432942	0	0.2761788	0	0.023906	0.043035	0.0265564	0.116762	0	0.0201398	0.4826835	1.1019965	0.7038595	0.1878723	0
[illegible]																	

Table 4S

List 1	List 2	Intersection
OTOP2	MAPKAPK2	CA1
OTOP3	TTR	CA2
AQP8	ALB	NR1H4
KRT24	STS	BCHE
CA7	APOA2	ADH1B
KRTAP13-2	GC	TPH1
MIR122	ANG	ADH1C
SST	CASP3	CLEC4M
TMIGD1	PIM1	PCK1
BEST4	BACE1	CMA1
DAO	AKR1B1	ELANE
MYOC	#N/A	CPB1
PYY	PDE4B	CTSG
PNLIPRP3	GSR	NR3C2
GUCA2B	MAPK1	MAOB
MS4A12	MAPK8	CFD
CCDC169-SC	SULT2A1	PGR
SLC30A10	CES1	THRB
PCSK2	MAPK14	LGALS2
HSD3B2	HSD17B1	CD209
LCE2A	AMPM2	ERBB4
KRT25	TGFBR1	IL2
GCG	SHBG	KIT
DHRS7C	RORA	AKR1C2
CD177	LSS	BMP2
LINC01289	SRC	CYP2C8
LINC00307	FABP4	FGFR2
CLCA4	DUSP6	CASP7
CHAT	REN	MAPK10
SLC17A8	CHIT1	TPSB2
ABCB11	PTPN1	PDE5A
LGI1	HSD11B1	FKBP1B
PABPC1L2B	ADH5	PIK3CG
LINC00682	KDR	NR3C1
BMP3	PTPN11	KAT2B
PLP1	PDPK1	EPHX2
SCNN1G	ESRRG	AR
ABCG2	TNNC1	HSD17B11
LINC01445	F10	GSTM2
SPIB	CSNK2A1	AKR1C3
RIT2	TTPA	ACADM
PPY	PYGL	LGALS3
HTR3E-AS1	GSK3B	BST1
CA4	DPP4	GP1BA
GUCA2A	PNMT	ITPKA
PRIMA1	LCK	CTSS
OR8G5	PDE3B	ITGAL
POU3F4	PARP1	CDA
PGM5-AS1	AMY2A	CTSF

FRMPD4	ISG20	IMPA1
DPP6	LTA4H	FGFR1
HS3ST6	HMGCR	GSTM1
NPTX1	CYP2C9	TEK
FUT9	ALDH2	VDR
SLC26A3	SERPINA1	PPARD
SLC6A19	JAK3	RXRA
LINC01976	CTNNA1	PDE4D
RBFOX3	TRAPPC3	PPARG
CWH43	SOD2	AKR1C1
CLDN8	GLRX	WAS
CEACAM7	FABP7	HNF4G
LINC01264	REG1A	NMNAT1
SERPINA9	NR1H2	ITK
RHAG	NR1H3	EGFR
CMTM5	PPARA	CBR1
GBA3	SULT1E1	FECH
SLC4A4	THRA	PDK2
UGT1A8	SYK	HCK
PHOX2B	GSTT2B	CASP1
NRXN1	GSTA1	ESR1
SCGN	XIAP	CDC42
LINC00974	FABP6	ANXA5
SCGB1D2	FABP3	MDM2
CDKN2B-AS1	PLA2G2A	DHFR
NGB	RNASE4	IGF1R
VSTM2A-OT1	FHIT	FDPS
TMEM72	NR1I3	PNP
SFRP5	F7	PPP5C
SCN7A	BLVRB	CTSB
VSTM2A	RHOA	ADAM17
LINC01855	AMY1B	AKT2
STMN4	FABP5	PAPSS1
ALPI	SELP	TYMS
SPHKAP	AMD1	SDS
ZG16	TPI1	APRT
FAM135B	FKBP1A	TNK2
CDH10	RNASE3	HSP90AA1
MAMDC2	ABL1	CTSK
RERGL	JAK2	FNTA
GPM6A	NOS2	GLO1
SLC26A2	GM2A	CDK7
XKR4	DCK	GPI
P2RX2	NR1I2	PRKCQ
ATP1A2	RARB	HSPA8
TEX11	RARA	PPIA
PMP2	ZAP70	MTHFD1
ASB5	MMP2	ACPP
C2orf40	MAP2K1	CSNK1G2
LOC1019281	SETD7	DHODH
CHGA	PSAP	YARS

GABRG1	MMP16	ADK
DCAF12L1	MAN1B1	EPHA2
SLC51B	NT5M	CLPP
CHST9	TGM3	CDK6
GABRG2	CSK	HDAC8
CNTFR	AGXT	GART
OR8D1	SHMT1	KIF11
USP2	HNMT	S100A9
TRPM6	PPCDC	GNPDA1
SLC5A7	HAGH	RARG
SLC51A	RXRB	NQO1
ASTN1	MME	BCL2L1
MORN5	CCBL1	DTYMK
CADM3	PPP1CC	TK1
TMEFF2	F11	NQO2
LOC1027241	BCAT2	HSP90AB1
OR1E1	UCK2	RNASE2
VEGFD	GSTO1	CDK2
TAT	STAT1	MTAP
SLC7A14	PCTP	CCNA2
MT1M	OTC	CHEK1
SCARA5	ARF4	CRABP2
LOC1027249	GRB2	LCN2
MT1H	HINT1	GSTP1
EPHA6	GSTA3	LYZ
OR2S2	TGFB2	NOS3
PI16	CLK1	AURKA
PABPC1L2A	PIK3R1	EPHB4
FEV	GCDH	RBP4
MT1JP	SELE	SORD
MYH11	TRDMT1	IMPDH1
HAND2-AS1	ARL5A	MMP9
LINC02000	ARG1	SEC14L2
SCNN1B		MIF
TCEAL2		MMP12
MMP27		AHCY
KHDRBS2		MET
DUSP21		PGF
PKIB		PLAU
LINC00838		BIRC7
TMEM257		TREM1
RXRG		SULT2B1
FAM180B		DPEP1
LINC02023		CYP19A1
LINC00507		MMP1
SMYD1		F2
NPY2R		MMP3
TCEAL6		MMP8
SLC25A34		MMP13
PCDH11Y		PAH
SH2D7		

CADM2
PRKG2
UGT1A10
SLC9A3
LOC105378137
ZDHHC22
ABCA8
LOC100128006
HAND1
UMOD
ANPEP
HMP19
SYNM
CHP2
GPR149
UG0898H09
KCNA1
CLEC3B
CNGB1
ADCYAP1R1
AICDA
NPY
HTR3B
SULT1A2
CCBE1
LOC101929106
ELAVL3
LOC101929538
KIF1A
SGCG
SYT4
LDB3
NEFM
FMN2
PLSCR5
SLC6A15
PRPH
KCNB1
LYVE1
CDH19
B4GALNT2
EPHA7
ZMAT4
GCNT2
PDZRN4
AGTR1
TMEM174
OLFM3
LOC105379252
TMEM35A
LRRN2

LOC107986321
PDE6A
MYOT
GPR119
MAL
LOC101928509
CASQ2
LOC105369438
NLGN1
ST8SIA3
GLP2R
SLC4A10
MGAT4C
C11orf86
NAP1L2
TMEM236
SLCO4C1
TNXB
TCEAL5
CISTR
GPR12
SYT10
UGT2B17
AMPD1
GREM2
MAPK4
SVOPL
LINC01532
PHOX2A
SPOCK3
SPATA4
LINC01497
OGN
PP7080
AKR1B10
HAND2
PYGM
CACNG5
LONRF2
MT1G
PTPRZ1
ANGPTL1
JCHAIN
GPR15
LMX1A
RIMS4
DPT
B3GALT5-AS1
SORCS1
FOXD3
COL6A5

ACTG2
LINC01616
ATCAY
HTR3E
CLVS2
CASR
SCN11A
ADTRP
PADI2
TFAP2B
LOC101928942
PCP4L1
ASPA
PIRT
GRIK3
CNTN2
SOHLH2
PKHD1L1
DHRS9
NEFL
AVPR1B
TMEM100
CNN1
C2orf88
LRRC3B
C2orf71
FRRS1L
GLYATL3
LINC01014
ENPP6
ABCB5
MYT1L
STMN2
TMEM151B
CTD-2270F17.1
ANGPTL5
MCHR2
HSPB8
INA
KLHL1
B3GALT1
VIP
OR10G4
BEST2
PGA5
FAM107A
HBM
SCG3
ARPP21
OR6F1
LINC01028

C7
ABCC13
GSG1L
NPY6R
FABP1
TAGLN3
LINC01284
HRASLS2
ELAVL4
NTNG1
CHRM2
TMEM37
TNFRSF13B
CACNG7
ADAMDEC1
MUSK
LOC105375115
PCDH10
KCNA4
CTXN3
HSD17B2
RALYL
MIR1270
GDPD2
PLN
TACR2
MAGEE2
SYNPO2
GALR1
LOC100506289
FCRL4
SCN2B
SFRP1
PCOLCE2
AQP4
SLITRK3
LINC01954
LINC01687
BTNL8
SLC17A4
C16orf89
STUM
CTNND2
HNRNPA1P33
FRMD6-AS2
SNAP25
DNER
TMEM82
PLAC9P1
EDN3
RBPMS2

PENK
HMX2
CELA3B
CLCNKB
SNAP91
UGT1A1
LMOD1
LINC01854
HMGCLL1
HTR3A
HTR3C
MOBP
SLC27A6
GFRA2
LYPD8
ATP2B3
NAALADL1
PLCXD3
KCNE2
IGSF11
POPDC2
PLD5
KRT27
ARHGAP36
PDK4
UGT2A3
HHLA2
GNAO1
HPSE2
PDZD4
PROKR1
FOXD3-AS1
FXYD1
TINCR
CPEB1
RIPPLY2
ANO5
CRYGD
MMRN1
LINC00836
PPP1R1A
LOC286178
MMD2
MAS1L
FABP2
TMEM196
LINC00461
TUBB4A
NOS1
TCL1A
NRG2

CDKN2B
MT1F
ABI3BP
MYOM1
MSGN1
CA10
SCN9A
ANGPTL7
SPINK2
ADGRB3
DNASE1L3
RPH3A
LOC105379030
DES
NOTO
DAND5
TMEM179
CUX2
LOC100508631
TRIM9
HSPB6
TNFRSF17
PNCK
HTR4
MASP1
CTXN2
LIFR
FLNC
HBB
HEMGN
MYLK
C7orf72
OSTN-AS1
KIAA2022
GFRA1
LOC101928535
WISP2
KLHL40
METTL24
LOC101927392
SDPR
SLC17A1
LDHD
TMEM59L
NOVA1
NXPE4
RASGEF1C
GAP43
CYP2B7P
CLDN23
FHL1

CA3-AS1
MAGEB2
LINC00488
KCNK3
KRT20
LINC01995
LINC01799
CBLN2
TM6SF2
NCAM1
ACTL6B
SGK1
EDN2
FAM138B
PSD
ALK
PGM5
CACNG3
DMRTA1
MYL9
C8orf88
SEMA6D
TMEM253
LOC105375009
BVES-AS1
NEUROD1
HCG23
LOC102724050
BMP5
SECTM1
KCNMB1
LOC101927188
TRIM40
HPGDS
SEZ6L
UBXN10-AS1
KRT222
ENHO
CCL23
UNC80
UNC5D
SLC5A11
RNF152
SELENOP
MOGAT2
B3GNT7
CHGB
OTOR
LRRC19
HPGD
MTHFD2P1

SVOP
ANK2
VSIG2
LVRN
PLA2G2C
B3GALT5
ASPG
MRGPRD
LINC01634
NRSN1
NECAB1
CNTN1
RNF150
TRPV3
KIAA1644
MAPT-IT1
C10orf105
EFHC2
NCR2
MRGPRX3
LMNTD1
LOC389895
FAM129A
LINC01324
HLF
TSPAN7
CYP4B1
HMGCS2
AHCYL2
ZNF536
CP
CD163L1
JPH2
KRT9
ADCY5
PIK3C2G
LGALS9C
LINC01625
UGT1A9
LOC339166
MYOCD
KCNQ5
CHAD
PLAC8
BTNL3
GABRA1
LINC01486
CCDC169
PAUPAR
GPT
GPR26

DCAF12L2
DIRAS2
MAB21L2
IL6R
CCDC69
SLC16A12
NMRK2
SLC32A1
OR6T1
ADRA1A
SCGB2A1
SMPDL3A
LOC100996693
KIAA0408
PSMA8
DPF3
SLC17A7
GPAT3
EYA2
ADAMTS9-AS1
P2RY12
FAM46B
LINC01776
CABP2
DEFB1
GSTM5
SLC16A9
CASC18
GRIN2A
MAB21L1
ADAMTSL3
PDE9A
SLC52A1
GRIK5
GRIA4
C1QTNF9
RIC3
EVX2
LINC00092
ENTPD5
NAT8B
IRX1
SLC18A3
KCNMA1
LINC01644
AFF3
PTN
KCTD8
LINC01016
MMP28
SULT1B1

MIR147B
CTNNA3
EPB41L3
LINC02237
LINC01537
NWD2
CR2
NEGR1
LMO1
JPH4
FDCSP
OR2L13
CHRDL1
FAM163B
CNTN3
MT1X
SNCB
SH3GL3
CPM
MRGPRE
MIR650
CNR1
EPHA5
NUGGC
ASB2
ZBTB16
ADHFE1
LIPC
TUBAL3
CELF4
ZNF492
TMEM220
LINC01314
LINC01158
LINC01505
XDH
SYT9
TAGLN
MEF2C-AS1
PTGS1
SERTM1
BVES
SFTPC
SEMA6A
DCLK1
GLRA4
LOC105378325
PHLPP2
NTN1
IL1R2
SH2D6

SCTR
ARHGAP44
AADACL2
SNTG2
LOC100507657
AARD
SLC8A2
LINC02185
PKNOX2
SLC2A4
HTR1E
GPR88
SCIN
BMX
UGT2B15
MIR30C2
ST6GALNAC6
HBA2
MBNL1-AS1
MTRNR2L1
NKX2-3
GNG8
APOBEC3A
GKN2
PEG3
SLC10A4
MIR2682
PIANP
SLC6A5
NPAS4
LRMP
RNF112
P2RY14
C1orf115
CYP2C18
C2orf80
FCRLA
LGALS9B
ARL14
LCN10
NXPE1
MYPN
GNG7
ATP6V1G2
KLF4
PTGDR
PPP1R12B
HRCT1
TBPL2
ACKR2
REEP2

STAP1
SORCS3
LINC00940
SFTPA1
PGPEP1L
TP53INP2
C1QTNF7
SEC14L5
SFTA1P
LOC101928307
CRAT8
ADRB3
FGL2
CEACAM1
EMILIN3
CHST8
ACSM5
TMOD1
PRELP
BEX1
SPAM1
RBM20
ROS1
LINC01502
WSCD2
HHATL
LOC105374428
CLEC10A
SRPX
METTL7A
RBM46
BCAS1
CRHBP
KIF5C
LINC01829
TRHDE
TMEM130
LINC01781
LEXM
OSR1
NR5A2
LAMA1
NTRK3
LINC00908
TRPC7
CALB2
NRG3
ATRNL1
KCNA5
MAPT
ABCA9

CARTPT
GPR27
MORC1
CASP5
MIR7844
MRGPRX2
CXCL12
PCSK5
SDCBP2
JAM2
GLDN
ASCL4
PDE2A
MT1E
KCNG3
CHL1-AS1
COL4A6
SCG2
AOC3
DACT3
LMO3
LOC105375650
MIR6843
PLAC9
P2RY1
NAP1L6
ANXA8L1
TNS1
LINC01082
MEP1A
CFL2
RAB9B
LOC101928847
HEPACAM
TTLL6
C8orf46
CRABP1
SPATA31E1
TNNT3
SSTR2
LOC101928932
CXCR5
HPCAL4
SEMA3E
LOC105372028
GAL3ST3
CNTN4
MFAP5
TLL1
JPH3
OR2L1P

HSPB3
FAM19A4
CHL1
MADCAM1
ATP1B2
NCAM1-AS1
C10orf99
CLEC9A
F13A1
LOC101927043
RGS13
PBLD
WDR78
GHR
UCHL1
WDR17
MT2A
MIR133A2
HSD11B2
DISP2
TPM2
MS4A1
FXYD6
FILIP1
SLC38A4
IGSF10
KLRF2
SLC23A1
HTR7
CRYAB
MRGPRF
SLC39A12
UGP2
DHRS11
CCL28
RUNDC3B
SLC28A2
BEND5
GCNT3
GAS1RR
CDH18
FCER1A
TMEM155
UNC5C
KY
RELN
FCGBP
GGTA1P
HSPB7
PCDHB1
STOX2

SLC30A8
GH1
PLA2G5
HLX-AS1
GFRA3
SELENBP1
L1CAM
LOC105376609
FETUB
OR1I1
SCUBE2
MIR5196
DUSP26
LOC401554
IRF4
LOC400553
C1orf186
C14orf180
ITM2C
SYT6
OPN4
TMEM220-AS1
GALNT16
LGI4
LINC01752
FAM205C
MPZ
PKD1L2
SLC24A5
KLHL34
MIR497HG
NUPR2
CHODL
IGFBP6
TSPAN1
RND2
DNAJB5
CA12
ISX
BAALC
CORO2B
PRKCB
LINC01114
DIRAS1
TLR10
CCL8
SERPINI2
GDPD3
GPM6B
SPX
VWC2

DSCAML1
LUZP2
ACSM2B
LOC105371899
LOC100507175
ACADS
SLC22A18AS
AADACL4
LOC285804
FCER2
FMO5
MIR342
CES3
GRIN3A
RBM24
MSTN
DPP10-AS1
PLCE1
SH3GL2
CNTNAP3
LOC100505851
PPP1R14A
TMEM171
PTENP1-AS
FOXF2
SUGCT
KCNH5
LILRB5
KIF5A
HAPLN1
CIDEC
ADGRL3
GCNT4
FGF14-AS2
CCL14
MIR4322
MIR3714
LOC285423
MIR1268A
RPRM
HMCN2
SLC7A3
MIR149
ASB11
ARL4D
CAP2
MALL
LOC283856
HSPB2-C11orf52
BLK
LYNX1

MIA2
ZNF676
LINC01475
SCN3B
CD79A
FGF16
MSRB3
SALL2
PTPRH
FRMD3
SLC9A2
CCDC152
KLRF1
SLIT3
SULT1A1
CDHR5
CXorf67
GDNF
PAQR5
ZNF575
LINC01331
ASXL3
DYNC1I1
CEND1
NMBR
SLC35F1
SIGLEC8
TEX101
ATP13A4
CNKSR2
SSTR3
MAP6
PLCD1
LINC00473
CCL13
XCL2
CCDC68
ARHGEF25
LINC01586
PTGDR2
ZBTB7C
CCL19
MT1HL1
ARHGEF26-AS1
CCDC129
CHST5
CYP4F29P
TMEM74
GCSAML
NPY1R
KLRB1

FTMT
WFIKKN2
VAT1L
ABHD3
CPXM2
ATP2B4
FOXP2
CDKL2
NPY5R
UTS2B
CILP
SKIDA1
LINC01736
FLJ22763
CCL15
MAFA
ARHGAP20
SIGLEC11
SCRG1
ANKS1B
PRKACB
FAM163A
KCNIP4
NAAA
CSRP1
SMPX
FAM151A
NCAN
PGM5P4-AS1
KIR2DL4
UGT3A2
RERG
KRT1
MIR5707
NMRAL2P
ZNF726
RAB3C
TARID
LOC105369486
C14orf39
CPNE8
FGFBP2
CASQ1
LOC105373878
CPEB1-AS1
LOC101929080
NLGN4X
TRIM55
STON1-GTF2A1L
PRAMENP
TMIGD3

KCNG1
ATP6V0D2
OR2W3
PLIN4
SLITRK2
TCEAL7
DMD
ETFDH
CDHR2
GPR55
LILRP2
FBXL22
NOL4
STAB2
PHGR1
BTNL2
NECAB2
CES2
CELA3A
CNTNAP4
LRCH2
SH3BGR
DPYSL5
LRRC55
ACKR1
LOC101928583
LINC01354
LOC100507387
YWHAEP7
LOC101929163
ZBBX
CNR2
NACAD
OMD
LOC101929268
GNG13
MPP2
RMDN2
ADAMTS9-AS2
GGT3P
MOG
SLCO2A1
GALNT15
SLC13A2
PHF24
PTX3
LINC00334
FAM189A2
CERS1
S100B
SPARCL1

BLOC1S5-TXNDC5
CXCL13
SLC9A9
ADRA1B
PIGZ
CD160
FLVCR2
TMEM132B
LOC101927359
KCNB2
FAM218A
EML1
CD36
KCNS2
SYT5
LPAR1
EDIL3
GDNF-AS1
NUDT10
TCF21
PPP2R2B
RYR3
ERICH1-AS1
CCL21
TRANK1
BRINP2
ADAMTSL1
LIFR-AS1
SFTPD
LIPI
GRIA1
LINC01352
FAM153A
ZNF229
TEX33
CA14
MRGPRF-AS1
NKAPL
PGM5P3-AS1
NLRP7
SLC37A2
KIAA0513
MEI4
P2RX1
MIR4680
MIR4432HG
BRINP3
MIR8071-2
RHBDL2
KLK15
SLC8A3

RGS9
KCNN3
ENTPD3
MIER3
LINC00484
MIR6076
PAPSS2
ZNF728
STXBP5L
LRRC66
TDP2
HIF3A
LOC101928994
PNLIPRP2
POU3F3
HIGD1A
KCNIP1
DPEP3
KCNA3
EEF1A2
P3H2
MPPED2
TMEM132D
PAX5
ITLN1
ENAM
C1orf94
RETSAT
CAND2
TMEM255A
SIGLEC6
MYO1A
CALY
NEU4
ERICH3
SEC14L6
PPP1R3C
CD300LG
STBD1
LY9
C7orf31
RCAN2
CHRM4
TGFBR3L
LGALS4
FAM46C
MYH15
GNG2
SLITRK1
PPP2R3A
LOC100507506

ITGA8
NAP1L3
UGDH
GGT6
LINC02232
ACAA2
NAT2
KIAA1211
RDH5
C14orf132
PDE3A
RSPO1
TAS1R1
MFSD4A
NEXN
LGI3
ADAMTS1
BDKRB1
MDGA2
TEF
EMP1
AXDND1
TACR1
PACSIN1
LOC101929217
KLF9
C9orf135
FMO4
HIPK4
RGMA
PNOC
LINC00977
CCR2
TLR3
CCDC26
FOSB
HOXB13
OSTN
PDLIM3
AQP12A
SEMA3G
CYS1
PIGR
GNG3
ABCB1
BFSP2-AS1
AKAP6
PDE7B
C10orf142
RPL10L
BEND4

SLC1A1
APPL2
SCN3A
FAM95B1
LINC00964
PCDH8
LOC101929340
LINC01266
LOR
SLC23A3
GPR37L1
SYPL2
CSMD1
AOC1
RFPL4AL1
WDR64
OR13C7
OR8S1
LOC154449
LRRTM1
KIAA1683
SCUBE1
LRP1B
LINC00582
MAOA
BEX4
TSPAN2
AGTR2
MAG
LOC389332
MS4A8
FUCA1
KBTBD13
SYN2
ABCD2
LINC00890
SMIM6
SLC36A1
SGK2
ADGRG7
PPP1R14D
BHMT2
SORBS1
PPARGC1A
ADRA2A
CLIC5
PTH1R
SSTR4
RGS22
CD27
IL1RAPL1

FBXO32
ANK3
FENDRR
FNDC9
CDH12
ABCA6
KLK13
ADRB2
CPNE5
MFAP4
PLPP1
GPX3
IGLL5
LOC102724804
LOC151484
SGCA
LINC00640
ASAP3
CDC14A
XKR7
CKM
ZNF835
LOC101929199
FOLR2
OR2T8
AQP7
SLC4A1
PEX26
PTCHD1
FNBP1
ADD2
GPR142
MPEG1
CYBRD1
FAM189A1
LIMS2
MCOLN2
UCN3
MT1L
GLIPR2
SEMA3D
CCNA1
SLC22A5
HAGLR
MPPED1
ATP8A2
LOC101929445
PER3
KCNIP3
LINC00689
ARHGEF26

LINC00675
SLC3A1
RGS2
ARL5C
LY6H
VIT
MEIS2
PRKAA2
CCDC158
LINC01133
SCN4B
BMP6
MIXL1
ANKRD33B
B4GALT1-AS1
PCDH11X
MIR4436A
NEFH
NRG4
C10orf107
NR4A3
CLDN5
VPREB3
CLECL1
SMPD1
RASD2
GPER1
CLMP
SLC6A2
SLC46A3
APOBR
SORBS2
GPA33
TPSG1
SNCA
LINC02206
TMEM108
CHRNA4
LOC100240735
RAB6C-AS1
LRRN4CL
TDRD10
SPINK5
FGF10
DNAJC28
TRIM58
STK32A
CD79B
KCNC1
KNG1
PRRG3

SLC20A1
OLFM1
SPIC
MXD1
ST8SIA6
PLCG2
C16orf96
ARMCX1
MS4A2
FGF10-AS1
MOCS1
NRAP
LANCL3
TMEM156
ACADL
ROPN1
ENTPD8
GZMA
LINC01798
ALAS2
SYP
MIR9-3
FAM133A
NBEA
HHIP
HMOX1
TINAG
MIR7704
TLR7
EPB41L4A
MIR378A
DSC2
IQGAP2
CNGA1
GPR31
GIMAP7
TPPP2
SYNGR1
IGSF9
SVIL
STYK1
CHRNA3
LINC00483
ADAMTS8
GRIK2
COL2A1
GNA11
CACNA2D1
NXPH2
LINC02106
APLP1

HS6ST3
ANKRD35
ZDHHC15
COLEC12
RAB6B
ATP2B2
CAPN13
DDX25
CLIP3
KCNJ16
MGP
CD19
LOC101927686
PRDM6
C1QTNF12
CBX7
GRAMD3
BFSP2
FLNA
MIR22
HRK
SMIM2-IT1
DUPD1
NTRK2
PID1
OR6M1
SLC41A2
GSN
ADAM33
PAG1
TLCD2
AMHR2
RAB39B
SUSD5
EIF4E3
OR51E2
NKX3-2
TTC22
PCP4
AP3B2
P3H2-AS1
MIR4756
ACOX1
MIR4530
SETBP1
KCTD4
BTLA
CMBL
ZZEF1
CNNM4
PAK3

ITGA7
PLCD3
SLC44A4
GRIA2
LOC101928812
CLEC19A
AQP12B
ACSF2
ENDOD1
ZNF667-AS1
TUB
DDR2
SYNPO
POPDC3
FCRL2
C1orf210
PPP1R16B
THEGL
PALM
SERTAD4
SRI
IRX6
C2orf74
SLC22A4
DAAM2
SMIM14
FER1L6
ANKRD7
SV2B
SLC2A13
EPB41L4A-AS2
PTPRR
PXMP2
CACNG6
SYNC
THEMIS
CNTN6
LINC01797
OR3A2
CPED1
LINC01140
EFCAB1
GPIHBP1
SHE
C4orf19
ELOVL4
ARHGEF37
ROR1
SGCE
SLIT2
ZNF804B

ENPP1
ANKRD20A4
FAXC
GIMAP1
MIR4732
NGFR
SLC22A17
LOC100128239
MXI1
SLAMF7
ACACB
B3GNT8
NIM1K
SPAG6
MEGF10
UBE2QL1
SQRDL
TSHB
CCR10
HCN4
CCDC80
CLEC4F
IL16
RTN1
JAZF1
CD48
ANKRD36BP2
SLC25A23
AOX1
BARX2
KCNK10
CD1C
C20orf194
FSIP2
NDN
LOC102724190
P2RY8
IL5RA
COL19A1
LINC01783
SLC25A20
TCN2
SLC17A3
RCSD1
MIR378I
ATP8B1
HCG2040054
SOSTDC1
LINC02012
TUBB2B
ZCCHC5

RIOK3
IL25
TEX15
PRDX6
HDAC9
CLIP4
LINC01629
MOB3B
FHL5
STON1
PRKAR2B
GLTP
C15orf38-AP3S2
MIR6887
PYY2
MEI1
OPN3
AKR1B15
CPT2
TRAPPC3L
TNFSF10
MAF
CAMK2A
SDR9C7
PAPPA2
SPTBN4
GDF5
LOC101928673
DCN
LOC100506700
MICALCL
PWRN1
TESPA1
SSBP2
SNX24
SPPL2A
MIR6883
VLDLR-AS1
KANK2
CCNJL
DLG2
UNC13C
SERPINA5
KCNC2
MESP2
LINC01018
MZB1
LINC01697
C5orf64
CLEC17A
CFP

NCCRP1
AMOTL1
TMIE
CTSW
HRASLS5
FAM124A
SSMEM1
IL22RA2
ADH6
MICU3
CYP11A1
LINC01489
PDGFD
TMTC1
SH2D1B
CFAP99
ARRDC4
CCDC178
AHRR
EXTL1
ITIH5
ACSM2A
MYL7
LINC01135
ECI2
CYSLTR1
TEX26
KCNT2
JAML
CDK5R2
REEP1
CAV1
PAQR8
CITED2
RADIL
CR1
SLC35G1
FIGN
OTUD7A
LINC01907
GPD1L
VSIR
CD40LG
FAM30A
LIMA1
TMPRSS2
EPB41L4B
PRICKLE2
ZNF471
B3GALT4
CDO1

LRTM1
TMEM200B
DPP10
LOC100128108
PGM1
TIFAB
DACH2
FOXI2
ID2-AS1
ITM2A
MEF2C
BCL2
KCNAB1
PTPRN
ZNF135
CALD1
GOLM1
RASL12
LOC101928834
PTGER4
LOC102723766
SNTG1
C10orf90
GPR33
IP6K3
SEC1P
TST
G6PC2
LRFN5
ST8SIA1
CHI3L2
CHRNA1
MIR7-3HG
HOXD4
FMO2
ETHE1
VEPH1
SIGLEC17P
GPRASP1
KIR3DL2
GIMAP8
MROH7-TTC4
TNNI3K
PLA2G2D
PLSCR4
ZNF831
LOC643201
LINC00892
CORO6
ZSCAN1
EPHA5-AS1

FERMT2
TRIM50
RIMS3
AMN
C1QTNF2
MEIS1
MIR4786
CYP17A1-AS1
A2M-AS1
SERTAD4-AS1
PTPRQ
CNNM2
TXLNB
FAM13C
VIPR1
NPR1
HSPC324
ZSCAN18
RHOU
SUCLG2
LOC102724488
BCAR3
TEPP
CCDC160
CCR9
ZFR2
MIR6071
GZMM
CCDC13
CLCN2
GRID2
OR14I1
SMIM10
MAP1LC3C
CELA1
12-Sep
ZCCHC12
FAM238A
PARM1
RORB
SPG20
TP73-AS1
GRAMD1C
PDE1C
KLB
NBPF1
FCRL3
IKZF1
MATN2
MIR6084
COL21A1

NCF1B
SPATA22
RIMKLA
MIR8071-1
FFAR1
FRMD1
TSPYL5
RAB27A
CD96
HSPB2
KRT12
FAM170B
TMEM63C
HEPACAM2
TMEM25
APOBEC3B
OASL
PLCD4
LOXL4
PI15
ACVRL1
LINC00704
TMEM72-AS1
MFSD6L
AFF2
BCL2L15
SLC46A1
NUDT11
DPYD
MB
CPA3
CD207
SVEP1
LINC01399
LINC01559
NXPH3
GPC5
MAP1A
LINC00996
RILP
CDKL1
PCYT1B
PLPP3
NPAP1
SNCG
CIDEB
ZIC4
CCDC85A
CCL16
GLI3
MYZAP

CGN
PAMR1
ZNF582-AS1
LOC107986927
PABPC5
CHP1
GNAI1
NEURL1B
PDZD3
CFAP221
PAK5
C5orf30
VILL
DIXDC1
ADH1A
ENPP2
CRB1
KL
KLHDC7A
SIAE
SLC6A9
8-Mar
FEZ1
CKB
KBTBD11-OT1
SHOX
TOX
GRIA3
TMIGD2
LOC101927943
NAT1
LOC101928161
TCEA3
ZNF418
SLC9A1
PLEKHO1
EPM2A
TLX2
PEBP4
RGAG4
ZBTB7B
FAM184A
TIAM1
GAPT
FAM181A
IFNG-AS1
SCN4A
LINC01539
LINC00494
ZNF257
APBB1

CACNA1A
CD1D
CROT
MIR4507
MIR4795
ZIK1
WBSCR17
OSBPL1A
MYOZ2
CACNB1
SAMD13
TSC22D3
LINC00570
CPT1A
ACAT1
FOXD2
DLG1-AS1
CACNB2
MAN1A1
SYNE3
ZNF677
C5orf66-AS1
TRAT1
CPEB3
AHNAK
SYNDIG1L
MAP4K1
HGD
POU2AF1
PTPRT
TXNIP
MYL2
TMEM56
IL21-AS1
SRL
LTK
CD180
MIR10B
LRRC4C
SYT15
LINC01563
AKAP12
KIF6
PDE6G
MIR770
MT1A
FLJ31104
KANK4
MGLL
PLXNB3
CRTAC1

LOC105371907
IGSF3
FAM161B
TRIM36
LINC01990
PRRT4
CNRIP1
SMIM10L2B
MIR6717
SLC22A23
NIPAL1
MMP23A
FBXO27
BANK1
WFDC1
SHISA8
CIPC
ZNF415
PLS1
GRXCR2
MRVI1
ZEB1
SLC6A10P
GYPC
ACVR1C
VSX1
SSPN
KIAA1161
ZNF781
CT62
TCF7L1
IL12A-AS1
FAM184B
CASP16P
LINC00877
MGAT4A
FAM107B
TRG-AS1
PPARGC1B
PTPN21
C16orf62
P2RY10
EAF2
DKFZp779M0652
CYP4F12
SLCO1C1
REP15
SMIM2
GPR171
TNR
MARVELD3

TDH
ADA2
PP14571
GCGR
SIGLEC1
SMIM2-AS1
ADAM28
FAXDC2
CPNE4
A1CF
NCR3
MIPOL1
MS4A4A
NRG1
DUSP1
CH17-360D5.1
EHHADH
CCL5
HK2
ZFYVE28
LHFPL1
TPM1
LOC101929331
MTM1
AGR3
DDX60
LOC105369203
RSAD2
ICAM3
RBM47
DSEL
TSPAN18
RNF125
NEDD4L
P2RY13
CCR7
TESK2
CX3CR1
GOLGA8O
RCAN1
AKAP5
FCMR
GAS7
LINC01915
LEAP2
PHYHD1
CAMP
ZNF98
PEX11A
SLC5A8
SLC2A5

ATP2A3
NYAP1
RNASEL
TF
LOC150051
GALNT12
MIR936
LOC107985092
TNFAIP8L3
LINC01215
ZNF439
LOC400043
H1FNT
NCF1
NR0B1
LINC00930
FBLIM1
POU2F2
CD69
GBP3
LOC100506725
LINC00840
GABRG3
PLA2G10
GRIK1
UNC79
GLOD5
LOC729970
ZER1
SLC6A11
EVPLL
OLFML2A
CLMN
TRIM63
SLC35D1
SNAP25-AS1
PROM2
VWA5A
PRUNE2
PCDH15
C1QA
JAM3
EVI2B
ATP8A1
LRRC31
EBF1
CMKLR1
TTLL7
NBPF25P
NEU2
GPR174

GIMAP6
MUC12
LGR4
HSD17B6
CAPN5
FOXF1
SLC6A18
NCF1C
MIR4775
MARCKS
PHYH
RBMS3
DENND2A
KLHL6
ST6GALNAC1
SOX15
LINC01336
NAPSB
SLC39A5
PJA2
CKMT1B
SLITRK4
LOC105369187
RASGRP2
FOS
PAFAH2
VSIG4
SOWAHB
CYP1B1-AS1
BNC2
EDNRB
BDKRB2
RGS18
BTK
ZIM2-AS1
ZC3H12C
LOC106699570
LEPR
CACNA1H
COL28A1
C15orf59
VIPR2
CAMK4
PTGIS
ACTA2
GIMAP4
STMND1
LOC101929897
LINC01531
FAM162A
SMTN

MYBPC2
LPCAT3
CCDC136
ABHD11-AS1
CECR7
VWA3B
LOC100506444
EPHA10
DTNA
ADGRA1
CASC16
OTOA
LOC105371592
CLIC2
F2RL1
NDRG1
TGFB1I1
LOC400464
CLCNKA
GABARAP
RIPOR2
EPAS1
LINC00551
TSPAN11
BIN2
LOC283683
LINC00882
RBKS
FAM180A
ASCL1
RSPO3
LMO2
ARHGAP28
KIR3DX1
CD8A
LINC01197
SLC24A4
LINC00294
GYPE
KCTD12
GABRB2
KCTD9
CYP26B1
UMODL1-AS1
DCHS2
LRRC1
MIR339
CPAMD8
LINC00671
KIRREL3
KCNMB2

LRRC75A
ZNF304
OCEL1
ZNF157
FAM134B
TP53TG1
OR2A7
ITGB3
STK32B
RAP1A
CACNA2D3
CYP8B1
SEZ6
KIAA1462
SFTPA2
IKZF3
ZNF582
ABCA10
ANO10
SCARA3
RHOF
CAMK2N1
C19orf33
RANBP3L
SDHD
RECK
CORO2A
MAGI2
SPAAR
PELI2
ANKRD55
ADCY9
PRKN
MIR7847
FAM110B
FAS
UST
KCND3
PRSS36
NECTIN3
NRXN2
PALLD
SCP2
ST6GALNAC3
RD3
ABHD5
MIR4697
LITAF
KIF13B
CD3E
ZNF542P

SOX5
NAALAD2
SMPD3
ZNF568
SLAMF1
LOC100505501
LAX1
CCL11
APBB1IP
C5orf52
DCLK2
MYCT1
CCDC13-AS1
FGF9
LINC01344
FBLN1
AP3S2
ZNF454
WDR86-AS1
KLHDC8B
ARHGAP15
CD244
MPC1
LOC105378828
XCR1
FAM43B
PITPNM3
S100Z
ZNF667
LINGO4
ANGPT1
ITPR1
A2M
SCGB3A1
YPEL4
RAB44
GPRIN2
GPR156
HACD4
LINC01122
CKMT1A
C3orf70
CD3G
CD52
CLDN19
SPECC1
SESN2
PHF21B
TMEM61
BACH2
KCNJ2-AS1

LINC00982
ANKRD20A2
RPS6KA6
RNF180
MS4A6A
ANKRD20A1
SLC8A1
FAM49A
SERF1A
GVINP1
LTBP4
USP51
SYTL2
SLC4A9
SIPA1L2
LILRA4
PTGR1
STAB1
CHRNB4
DENND5B
PDE1A
TMEM30B
SIAH3
MISP
TAGAP
TRIM61
CDKN1A
TNFSF12
ANKRD9
ST3GAL6-AS1
LOC101927495
PCDH7
LRRC7
FGF2
RAB37
PDE1B
C16orf45
ADRA1D
SATB2-AS1
MS4A3
ZFP3
TSHZ1
MAP2K4P1
RAVER2
FCN1
GAREM2
PBX1
SH3BP1
EFHD1
PPP1R36
CSPG4

SP140
SAMD9
ENKUR
KCNS3
ADRB1
MIER1
JAKMIP3
FBXL7
WASHC2C
CFAP54
TPSAB1
ABCC6P2
LOC100507547
TAX1BP3
SYCE1
ARHGEF18
IL17B
WNT9A
LINC02139
IL10RA
CASP12
HFM1
MIR3615
ADAMTSL4
ADGRB1
FST
STPG1
LIPH
CDON
IL10
ENOX1
PCDHB5
RGL1
GPR34
BDH2
ZNF528
GIMAP5
DENND1C
SSC5D
NSG1
OR5K2
LOC100505622
LINC01268
PLCL1
CD200R1
LARGE1
MS4A7
CFH
UNC45B
FAM3D
SPECC1L

FAM187B
FOXH1
MIR3622A
ZNF549
PTGDS
RAPGEFL1
GALM
APBA1
LY75-CD302
GNG12
SOCS2
LOC102724927
GALNT13
KLHDC1
SLC25A42
ACKR4
TAL1
LXN
PREX2
LRRC3-AS1
CCDC96
TWIST2
PTRF
LOC151174
TTC28
C2orf72
HDC
KLRC1
ADGRV1
B4GALNT3
MTUS2
NOL12
NEO1
TGFA
RASGRP4
STAC
FOXN3
POLD4
SLC47A1
HVCN1
SGSM1
ZSCAN5B
KRCC1
ECE1
DOCK3
NFASC
MAP2
PHYHIP
PDE8A
CD101
COX7A1

LINC01588
PRR5L
CAPN2
ABCD3
ATL1
SSTR1
MPDZ
RPS6KA5
EVI2A
CXorf21
PQLC2L
CLIC6
RYR1
CASD1
PRX
NCS1
ITGB7
COL23A1
MYO15B
C22orf23
PBXIP1
RELL1
MEOX1
SLA2
IGLL1
ADAD2
SLC6A8
FLT3LG
DHDDS
TKTL1
LOC100130451
ATP13A5
LOC101927143
SIGLEC16
TOM1L2
AKAP7
ABCA5
ESR2
MAP1B
TFCP2L1
TSKS
VAMP2
CSF2RB
SAG
EFNA5
GJC1
DMPK
FLI1
SH3BGRL2
USP44
KBTBD12

MED12L
PLEKHG6
NPIPB6
SLC26A7
HEXA-AS1
AGFG2
STX19
CAPN9
TMEM140
NHSL2
CYSLTR2
OLFML1
TNFRSF11A
CD33
ZNF396
TRAM1L1
EBI3
GNAZ
SOWAHA
B3GNT6
CD247
ETNK1
TJP3
MAGI3
2-Mar
LINC00694
BCL2L10
C17orf97
PTK2B
NUTM2D
EML5
LY86
ZNF385D
C8orf4
SLC30A4
ZNF710-AS1
KBTBD11
PACSIN2
HCLS1
FKBP5
SPESP1
GNAL
STOM
ZFPM2
IGF1
PMP22
STK17B
LOC102723548
TEX43
NELL2
POM121L2

ZNF540
SELL
LOC100505715
AIF1
GNG11
TGM4
NDRG4
PDGFRA
TRAF3IP2
HADHB
MRC1
LY96
PIP5K1B
STK33
DZIP1
AQP11
GINM1
CD7
HADHA
ZNF772
SCAMP5
TP53I3
HSPA2
CALCOCO2
C1QTNF3
LAMC3
TSHZ3
NXPE2
PLXNA2
CLDN7
PNMAL1
SLC1A2
RAI2
ANKRD53
COX6B2
DOCK2
CBX6
SH3BGRL
PHLDB2
LOC100996583
CD37
FBLN5
SLC18A2
CD38
GDF6
IL10RB
ZNF626
SPN
NPPC
TMEM169
MYOZ3

C10orf128
RGS5
SNPH
P2RX4
ZEB2
LOC100129503
TMEM47
PANK3
SASH3
TRPC6
LOC100506274
LDOC1
TMEFF1
MAFTRR
KRBA1
LINC00668
HS3ST5
SOCS6
LAMA2
SCAMP2
OR2C1
PIK3CD
FTH1
C6orf136
PLOD2
ZNF671
LAG3
ADORA2A
PDZRN3
LTB
RGS8
GNGT2
RARRES1
NUTM2B
PTCRA
LOC105375431
MYO1D
CPTP
HES5
ELOVL6
LOC101929552
MUC4
CD24
URAD
SOS2
KITLG
VSTM4
FAM214B
SIRPG
MDGA1
TBC1D9

RIMKLB
TMEM54
TNS2
CD163
PROB1
6-Sep
LOC100130111
SLMAP
ARHGEF6
DERL3
AFG3L2
RASSF6
MBOAT1
FAM110D
CD302
TNFSF8
ZNF134
SMCHD1
SDK1
TMC4
NCKAP1L
BMF
GNA13
ME2
LOC100996286
VPS4B
TMEM133
SCN1B
LURAP1
SUCLG2-AS1
ANTXR2
GRAMD4
ACOT4
ARHGAP25
DNM1P46
CACFD1
C1orf21
STEAP4
FASLG
BIRC3
SYNE1
WDR7
KIF1C
BHLHE41
BTBD3
RUNDC3A
EFS
ZFP82
TBX21
BCL2L11
MPP7

ZNF132
MPPE1
MAGI2-AS3
LHFPL3
THCAT158
C1QB
LINC00865
PRSS8
STRADB
KCNQ4
LOC100422737
HHIP-AS1
MMP25
PYGO1
C11orf54
ADAM11
SV2A
HADH
SMAD7
ESPN
COL25A1
EMCN
BOC
PKD2L1
TAT-AS1
RHOH
CYP21A1P
RHOC
GAB1
MTNR1A
ZNF793
LOC729966
CSF1
SENCR
GABRA2
MATK
WWTR1
MAN1C1
MIRLET7D
PRR15
MAST2
TMEM45B
A1BG-AS1
TMEM59
LRRK2
TBX1
TMEM120A
ACSM1
CCM2L
EHD2
DDT

RFK
HCFC2
TMEM131
ANKRD13A
CD3D
CPQ
COL14A1
SLC26A4
APCDD1L
WASL
SCIMP
HEATR5A
GRAP2
MIR4477B
FAM214A
ALMS1P1
KLKB1
LPP
TGFBR3
GSTM4
MGARP
CSF1R
ACOXL-AS1
LRFN1
CDH23
CDIP1
CARD16
UQCRFS1
LOC100506474
ATP5A1
JOSD1
FCAMR
FNIP2
IPCEF1
FAM46A
NUDT12
VAPA
LOC102724933
ACSM3
ZNF821
LOC105375700
TBX10
IL6ST
MIR4687
PLPP7
JAKMIP2
ACADSB
CASP10
SMC2-AS1
B2M
ABHD6

ZNF625
CGRRF1
LOC101927702
TRAK2
TCP10
FRMD4B
STX12
FLNB
CPEB2
TP53INP1
TUNAR
FAM198A
LOC729739
DPY19L2
GPR17
BCL10
RBP7
VLDLR
PTP4A1
HPD
FAM117A
SLC6A16
CDC42BPG
HMGCL
PTPRC
KLRG1
PC
SUN2
RNF11
BTC
ARHGAP10
LRRFIP2
PCDHGB7
ATP23
LOC101929134
PCDHB4
SLC15A2
MAB21L3
ARRB1
ARSA
ELMSAN1
MFNG
PKIG
ACAA1
ATP2B1
LINC01535
SEMA4G
NLGN3
CCNG2
HPSE
FAM83E

LETM1
FBLN2
DOCK10
GIPC2
PYHIN1
DLGAP3
CEACAM3
HLA-DOB
ZNF844
WLS
AVPI1
SYT11
BSG
TLE4
CLGN
EGLN3
PHF7
KIAA1522
PECR
PRKG1
CAB39
RUNX1T1
FGF7
RAB4B
DTX1
GPR162
ZNF788
CLYBL
NEDD9
CD28
C17orf50
ART4
NCOA4
ATP5J
RXFP1
SOX7
POLR2F
AKAP9
CYP2J2
PNMAL2
SGPP1
DPEP2
TUBA1A
FOXA1
RNF186
CNST
ARHGEF35
VNN2
C18orf32
SOX17
MFSD11

KIAA1671
EFEMP1
WNT2B
LINC02202
BTD
FGF12
MPND
ZFP2
ZCWPW2
DYRK2
XK
PTPRF
ARHGAP42
LINC00665
MAP3K15
PCDHB2
MYH3
PSMG3-AS1
GPR183
NHSL1
RNF138
NPY4R
CRTAM
RIPK3
SRP14-AS1
IL18RAP
CHMP1B
IL18
ZADH2
ADGRD1
LOC105376731
DNAJB4
CHST15
MLXIP
TRPM4
FYB
RAB30
SOBP
ST8SIA4
FAM126B
TCEAL3
ZNF491
GAREM1
DNM1P51
TMEM127
GNAQ
PRPH2
MPP5
SUSD6
C1orf106
MSRA

PQLC1
IL11RA
PCSK6
HECTD2
ALDH6A1
CTB-178M22.2
NGF
LINC01207
MAGEH1
FAM159A
ACSS2
AXL
DECR1
GPLD1
PDCD6IP
TRABD2B
PDLIM2
GZMK
TLN2
S1PR1
LRRC18
TRAF3IP3
ADCYAP1
HHEX
PURG
LOC100132077
RASSF2
TOX2
DAB2IP
TNFSF13
ANO4
C4orf33
ST3GAL4
ATP1B3
MAGIX
SOCS2-AS1
CAMTA2
GBA
ARPIN
A1BG
LOC100507053
DOLPP1
ALDH1A1
MFSD7
PLEKHH2
LOC101928820
KLK1
LPP-AS2
FOXO4
RAB42
SATB2

FZD7
ACO2
EPHX1
LGALS9
MUC13
COL4A5
KCNJ12
TMEM9B
PLEKHA7
DMGDH
TRIM34
MIGA2
NANS
1-Sep
SPON1
AP5M1
CYB561A3
KCNK12
LOC553103
MIR6872
FCGRT
BMP8B
MAP10
VAV1
ZDBF2
LMBRD1
ZSWIM6
FOSL2
B3GNT5
PRSS12
FEZ2
VAMP8
TRPC1
NXPE3
LRRC4B
MAP2K6
CDC42EP3
KCNH1
ZNF287
KTN1
SLC6A12
OTOGL
ILK
BEND6
CYYR1
LINC00304
FLRT2
VCL
GSKIP
WDFY3-AS2
BAD

MGST3
PPM1A
SHROOM3
SALL1
CYB561D1
THSD4
UNC5B
SPATA24
KLC4
SLC31A2
CYTL1
CDK20
TCP11L1
METTL7B
OLFML3
BCL2L2
OAF
ATG4A
CD4
ACSM4
FITM1
RPS6KA1
EGLN1
SLC35F3
4-Mar
WASHC2A
RCVRN
CALML4
C1QC
MXRA7
RBFOX1
SHC4
KCNK5
TLR9
LCP1
DOC2B
ZCCHC24
EBF4
TYRP1
BRPF3
MYL6
HSBP1L1
TUBB2A
TNK1
BPNT1
FAM19A2
ITM2B
KLF15
THAP7-AS1
SIRT4
LOC103611081

ARHGAP30
TRPA1
RGP1
CD8B
CERK
KLF6
ABLIM3
S100A14
MCF2L2
MYO1C
C3orf18
SYNJ2BP
SHISA7
C2CD2L
STAR
CALM1
GLIPR1L2
MOB3A
CEP70
IL18R1
DGKA
PVALB
AQP1
HSPA1A
PLD1
EPHA4
CAST
SMAP1
IDH3A
SMIM5
NFE2L1
FZD5
NTN4
IL1R1
TLR1
GNE
DCX
NCAM2
TBC1D1
OPRL1
CAT
RNASE1
TDRP
SLC16A1
LOC102724163
CLRN3
TEP1
CALHM1
ST8SIA5
HID1-AS1
CRADD

CAMK1D
DIRAS3
INPP5J
CDC42SE2
F8
BORCS6
GOT1
ABCC3
FLG-AS1
MAPK3
OR7E14P
LOC102724200
GRASP
LGALSL
SLC27A2
LIX1L
LINC01504
CRAT
KLF8
NLRC3
MAST3
GMFG
ARSF
GCK
MAPK6
ARHGAP9
CTSA
LOC339803
ZNF347
CASZ1
SH3KBP1
IL2RB
CAMK2G
KIAA0232
LINC00324
SPATA41
RAB6C
RGS1
MTHFR
EPHB6
EAPP
TLR8
KCNJ2
MBOAT4
SLC6A17
HECTD3
INPP5A
SLC17A5
TBC1D8-AS1
STARD9
BLNK

TMBIM4
ZNF43
SLC47A2
MIR5572
TAPT1
RGS6
SLC35A3
LOC101927755
SAMD4A
GOLGA8R
ZFP36
MVP
DNASE2B
VIM-AS1
FAM78A
TNFRSF1A
FAM102A
LOC646588
CYP27C1
TPMT
HLX
SPTLC3
PCK2
TNFAIP8L2
SERINC1
ITPKB
KCNN1
BTG1
HLA-DQA2
ADAP2
FAM127C
RNF185
OXCT2P1
RASSF5
CTSD
TPK1
SLC25A18
MPI
TSPAN8
PLD4
NOX5
ARL4A
CYP4V2
AMT
ZC3H12D
MBNL1
DOK4
CD2
GSTM3
RRAS2
SERINC2

ATP5F1
SECISBP2L
C1QL1
NCOA1
NDNF
NMT2
DIRC2
ACADVL
ATP6V1D
CDS1
CAMK2D
SLC44A1
FBXL5
UBASH3A
FYCO1
MITF
MRPL35
RARRES2
PIK3R5
MROH7
ATP8B4
LINC01447
CDC42BPA
NCR1
TSPAN3
HLA-DMB
MAPRE3
ATP2B1-AS1
ADD1
GON7
CCDC85C
SPINT1
TMEM251
SUCLG1
NXN
SLC12A3
FGD2
PPM1E
RNASE6
GGT1
NMUR1
FCRL6
PRELID2
HIST1H3E
PFKFB2
ECHDC1
TAC3
TSLP
GNPTAB
LOC101929422
WTH3DI

LOC105376527
HIBCH
AKAP2
PDE6B
VTI1B
TMEM246
CYCS
STAP2
ZNF684
ABO
SAMSN1
PCDHGC3
PLA2G4F
SV2C
GHITM
CD226
ZNF829
GBP2
PSEN1
TBC1D10C
TMOD2
MYOZ1
PRNP
HCST
PTGFR
ALDH3A2
UAP1
TMEM240
AGBL5-AS1
QRFP
TRIM31
IMPA2
C15orf52
PCSK7
ZNF256
EXOC6B
LOH12CR2
ZFYVE1
DQX1
AIM1
P2RY2
SLC35D2
SLC25A4
MUT
SLC25A35
RASL11A
TNIK
IFIT1
KRT19
ASB8
C1orf226

AGL
HDHD2
GDE1
MKRN2OS
FAM13B
UBL3
BAK1
HLA-DOA
DENND1B
MDFIC
PPP1R12C
CD2AP
LINC01589
TUBA3FP
PPP2CB
ACTN2
CDH17
RNLS
FERMT3
DTX4
ERBIN
AIFM3
SMARCA1
SIT1
SFMBT2
PLGRKT
TADA2B
PPP1R14C
FRRS1
LINC01842
INSC
ADAM23
CLEC14A
FAM213A
DCAF11
DIP2C
NUDT7
COX5A
MYO5A
SLC25A24
BDH1
MGST1
FRAT1
RPL34-AS1
ZMIZ1
PPP1CB
THSD7A
MIR6852
TMEM170A
BTBD9
ZNF467

RHOJ
MYO7B
A2MP1
TLR5
C14orf28
LINC01934
GPC6
ZNF404
NKG7
MINDY1
KCNA2
PNMA1
CYP46A1
MUL1
LHFPL3-AS2
GABARAPL1
SMIM10L2A
CARNS1
MTMR9
LINC00987
ZNF595
C1orf162
AVPR2
BBOF1
FEM1C
LOC643733
CADM1
SYNJ1
MTUS1
ADSSL1
SASH1
SNRK
EFCAB5
EIF4EBP2
ZNF350
ASB7
DOK2
C17orf107
CTNND1
SCNN1A
ADCY6
ILDR1
EPHA3
ACOT13
RALGAPA1P1
ADGRL2
ARHGAP35
PKP2
PIGS
RNF5
KALRN

CC2D2A
WIPF2
ELAC1
MYL12B
STARD8
CDC42EP5
AUH
ITGA3
NRROS
ZNF662
CHFR
MAPK7
SLC25A48
MEF2D
RGS10
HTR2A
NEURL3
PTK6
TXNDC11
REM1
DSTN
MYO5B
EQTN
SCO1
PTBP2
SCN5A
HLA-DQA1
NIPSNAP3A
FCGR2B
ARHGEF9
MIR5187
OXCT1-AS1
SIRT6
NUDT16
IFIT2
AZIN2
FBXO8
CHMP6
PRADC1
LINC00327
RORC
ANKRD12
POMC
PRR13
NR2F2-AS1
CD14
MTMR3
ATP5I
OPTN
ITGA4
OSGIN1

ETFBKMT
FAM8A1
SEC23A
GOLGA8N
SAP30L
RNF103
NRARP
TBC1D10A
PIEZO2
SYT2
AKT3
CYP4F22
HRC
LOC102724532
DSCR3
MAGEE1
NTAN1
KLF13
WASF3
MILR1
NRIP1
CATSPERG
SMARCD3
C19orf35
ATP5O
EVC2
ANKRD44
PGGT1B
SDHA
PRCD
CHCHD10
CYGB
TTC6
MMRN2
GPBP1L1
MYLK3
ZNF624
APC
DTX3
GPR146
ECSCR
ZNF528-AS1
ABCA1
RRAGC
SORBS3
ATP8B2
LPCAT4
SLC35B3
RNASEK
GAL3ST2
SLC20A2

KLF3
ARF3
TRAPPC8
HEXIM1
SLC25A30-AS1
FZD4
CEACAM21
GPRASP2
LOC101928414
ANKS4B
CCDC107
NDUFB1
TNIP1
EGR1
SNORD138
ZNF883
ZNF682
ATP6V0D1
MTF1
FAM118B
TTC30A
SH3RF1
ABI3
ST3GAL3
CCDC87
LAMA3
KIAA1614
ARNTL
ATOH8
PRKCD
MRPS36
SIPA1L3
HIST1H2AC
GIPC1
PRKAR2A-AS1
FGD4
LOC100129534
LOC101928530
ALAD
LOC105369364
ANKRD6
SETD9
FOXO1
RAB9A
TRIP4
OSBPL11
SLC5A10
CTDSP2
MICAL2
SPAG16
SHOC2

ZNF569
UPP1
AK1
HERPUD1
ZNF429
ZNF658
ACOT11
CCNDBP1
MMP15
C6orf47
INCA1
PTTG1IP
PLPBP
GRID1
ZNF718
CREBRF
ABHD15
NDUFB3
NBR1
KLLN
DMXL1
ID3
CCDC184
IPMK
LOC100996455
DERA
FAM229B
PODN
KATNAL2
SPATS2L
SLAMF6
LOC101928728
SEMA3B-AS1
FRYL
LINC00667
TICAM2
YPEL5
XYLT1
SLC35A1
AKR7A3
APLP2
NCKAP5
ID2
STAT4
LOC102724064
LRP10
LYL1
KLF10
LHFPL2
GSDMB
PINLYP

C14orf159
CSRNP1
GALNT7
VCAM1
WIPF1
SLC5A5
CTSO
ADGRA3
ZFP91
KDM4A
E2F2
ZNRF2
HNF1B
RAB5B
SERF1B
MFN2
FAM83G
CYTH4
CMAS
SGPP2
LOC105378753
ATP10B
PRDM1
PAOX
RIPK1
SH2D1A
PAFAH1B1
CHMP2B
LTA
ZNF75A
SYNJ2BP-COX16
NDUFA5
SIDT2
HES2
CPEB4
SDHB
CD6
TPRN
FAM149A
VIM
PYROXD1
ALAS1
BTG2
TMCO1
PPM1L
RBP5
ANKDD1B
TAB2
ECH1
TTC38
ZDHHC7

ARHGAP17
IQSEC2
NDST1
CNPPD1
GATA1
CA11
NPTN
MTMR7
ABCG1
CHADL
ZNF366
LOC100996717
PNPLA7
URGCP-MRPS24
P2RX7
CRY2
ZNF382
BHLHA15
TOGARAM2
DAPK2
TTC39C-AS1
SNAP29
SIDT1
SEL1L
ZBTB4
TMEM164
SPECC1L-ADORA2A
WBP1L
LOC100506679
ATP1A1
SGCB
PDLIM1
SKAP2
CLEC1A
ATP5G3
SMPDL3B
SLC16A2
NIT1
MKNK2
FAM171B
TMEM254
CYTIP
RDX
KIFAP3
SCARB2
SLC27A4
CD34
TPRG1L
SNX32
KRAS
GAB3

LOC100506368
ATG4D
BAHD1
AVIL
CCDC106
LUZP1
RASAL3
MIR600
NCK1
EVL
SAR1B
MGC16275
EXT1
ZNF25
SHD
CYP2U1
VPS37B
SEMA6C
ACOT2
USP38
FAM120AOS
SEPHS2
POU6F1
LINC01637
USP30
SIGLEC10
MTFR1L
SLC45A1
PRKCE
SDK2
SLC44A2
VPS13D
BORCS7
EID1
SFXN1
MTMR10
CD53
GAK
TMCC2
ZNF763
UBQLNL
TMEM63B
NCOR1
CCR5
MPDU1
LYSMD3
ZBED5-AS1
EMP3
PTPRS
FBXL17
CRLF1

LPIN2
HOTAIRM1
RASSF3
MAP2K4
AFF1
ADORA2B
ZNF271P
PLEKHA6
SSFA2
MPC2
NAIP
NFKBIA
ITSN1
NDUFA6-AS1
C15orf65
PTENP1
NDUFS1
HOXA13
KCNJ10
PGAP3
SRSF5
TCF7L2
ETFA
TMED6
PTPN22
LOC100996763
MMAA
C11orf63
SLC25A12
ZNF331
PRKACA
OSBPL7
RAB1B
GBA2
ACER3
HLA-DPA1
LMTK2
VSIG10
MIEF2
LOC102724362
CAP1
PCED1B-AS1
SPOCK2
SH3GLB1
SEMA5A
SFXN5
FZD1
ST5
MAP1LC3B2
RALB
PBX3

DSG2
FAM162B
AIG1
EP400
KIF3C
PCDH12
SMN1
LOC100287042
TAF9
RTN2
ZNF600
SBF1
TEN1-CDK3
CCDC66
TSPAN6
EFCAB11
SCLT1
SLC30A7
HGS
CUL4B
SMPD2
TGFB1
MIR3652
DHX57
MAN2A2
RPN1
MTHFD2L
NUDT19
ZNF766
ARPC5L
SLC10A3
FAM86EP
MREG
PHF5A
JARID2
CERS5
LOC100288123
C12orf60
ORC2
ZNF653
MED1
HNRNPU
TFB2M
SPATA5L1
ZC3HC1
PTS
ST20
CTNNBIP1
ATP6V1C1
METAP2
SPEF2

USP27X-AS1
LIPT2
CCDC43
MRPL53
KLC1
MAP3K7CL
RILPL2
USE1
CDK18
COPZ1
EXOSC9
MCAT
PFKP
MIR7113
DBN1
LOC101929704
LMF1
KDM5C
RFXANK
LOC105371050
MIR4651
PPHLN1
RALGAPA2
SAMD1
HMGB1
VARS2
EIF5B
CADPS2
LRRC75B
COMMD7
DTD2
PIAS3
BLCAP
DCXR
LARS
CHAMP1
TMEM51-AS1
GPAT4
TGFBRAP1
TSC2
EPRS
CYB5RL
ZNF138
NEK4
ZNF627
RPL23A
FBXO4
DPY30
SLC44A3-AS1
FUNDC1
ZC3H3

COPRS
PTPRG
MRPS30
TRMT5
TRAPPC2
RIPK4
CEACAM19
FHL2
LINC01123
ZNF12
GPN1
C8orf76
ARF4-AS1
ZSCAN2
TMSB10
PTPMT1
NCOA5
EFNA4
ATG4B
FANCF
GNAS
MRPL30
COG5
TBCE
RASL10A
PHOSPHO2-KLHL23
RWDD2A
LOC101927851
MPLKIP
ADAT1
SHARPIN
PANX1
RPS13
PARP2
KIAA0040
C2orf49
NHLRC3
FAM167B
TRMT10C
MTR
KCTD6
FUT4
RPS6KB2
ZNF496
SRP72
TCF12
FAM86B1
SNX25
SLC35E2
MCFD2
CASK

TIGD5
TMEM27
POFUT2
PCED1B
SIN3B
SREBF1
LOC100507071
NUDT3
NAA50
MANBAL
NUAK1
DNMT3A
SEH1L
TMEM68
FGFR1OP
RAD51C
PITRM1
RPAP2
TEPSIN
DDOST
B3GAT3
PTGES3
MRPL2
BTG3
EPOR
MIR210HG
INTS3
PPIL3
ADGRG5
DEF6
DDHD1
SSR4
MLLT1
AP4M1
DRAP1
PCIF1
DNPEP
MYLK4
ZNF184
POLL
LINC00870
PGM2L1
EFCAB7
SFR1
NME3
AMMECR1L
TSEN34
RPL4
MESDC1
MKLN1
IKBIP

PSMB3
CEP83-AS1
UBE3C
ABHD11
CWF19L1
PHPT1
RRM1
ARMCX6
LOC100288778
CEP97
FAM206A
VRK2
ELMO2
SSNA1
PIM3
DDB2
TTLL5
CYC1
LOC100507670
BRD4
GDI1
ARID5B
ITPRIPL2
NBN
DIDO1
ERCC8
PATL1
TPBG
PPL
ZNF75D
ALDH3B1
RSPH1
GXYLT1
ALG8
SNX22
LINC01806
ADAT3
TXNL4B
NSUN4
HSD11B1L
ARL8A
SAP18
NUP88
PSMD10
GEN1
TP53TG5
MED14
NIPSNAP1
AIP
ASB3
SWI5

GRID2IP
PSME3
KRIT1
COA4
COMMD5
DKK3
PKP3
ABHD14A
TEC
STX6
MOSPD3
CEP290
KRR1
PRDM15
RPP21
CAPN15
PCOLCE
TRIAP1
NUFIP2
ORC3
PIGA
CLIC1
ZCCHC11
CRYZ
RSL24D1
CTGF
APEX2
FGD1
TTC39C
ZNF83
UVSSA
EIF2A
GPS1
PTK2
TRNT1
ZNF544
POGLUT1
RBM15
DPAGT1
ADGRL1
SMC1A
ATF7IP
SFXN2
FUK
RGS17
CEP128
BCL11B
DNAJC6
PRPF38B
FUT11
SESTD1

H2AFY
MAP3K21
PIGW
RPUSD3
MIR3605
TMCO3
EIF5A
RNF169
TCIRG1
DFNB59
IQCH-AS1
OSGIN2
ZNF580
TAF9B
ASB16
TRAF7
SNU13
ACOT7
LOC101929147
RDH11
SFSWAP
FBXL19
ASNA1
ZNF713
RPL31
LSG1
EDEM2
HID1
EIF2S1
ANXA9
EEF1B2
NLN
SEMA7A
CBLL1
ZNF398
SHANK2
SART3
ZFP62
ABT1
DHPS
LILRB4
ZNF324B
ABCB8
APTX
ZNF414
PRRC2C
CRYGS
ZNF503-AS2
GTF2I
LUC7L3
AGER

NRM
NFKB2
EMC9
CXorf40B
NOD1
DPY19L4
URI1
ACCS
TDRD6
MEN1
EYA3
ANP32E
CCDC130
ZNF558
PLXND1
TEAD2
MAPRE1
PPT1
ZKSCAN2
NAXE
CENPB
KIF5B
SLC25A17
MIR611
CCDC134
C5AR1
MPV17L2
MSANTD1
CENPX
HOMER3
PRTFDC1
RANBP2
SENP1
FLT1
DNASE1L1
PRPF31
PMM2
RPS8
JRKL
SLC24A1
PSMA1
ARL13B
ZBED3
MIR7109
KIAA1147
KRTAP5-AS1
NCOR2
TGM2
IGSF22
CEP170
ENAH

DAZAP1
POLR2C
RPL10A
PSMB4
STK35
MED17
ABHD12
ZNF507
LRSAM1
SOGA1
LOC100506603
PGS1
MPHOSPH10
PSMB7
CERS6
ICE2
PLEKHG2
ATP2C1
CDYL2
C3orf52
TMED7-TICAM2
PSMD8
DHRSX
VGLL4
ARMC5
CCDC162P
TRAF1
ZNF131
SMIM13
COPS8
TMED4
ZNF451
TMEM5
FKTN
ZXDC
FOPNL
LCTL
LZTFL1
ANKAR
ZSWIM3
NSUN2
SUMF2
DZIP1L
TTC5
ZDHHC20
PEX16
SFPQ
RSRP1
RPAP3
RAD23A
MSC-AS1

ACTR3C
FAM84A
METTL6
ZNF37A
UNC119
TIMM9
LIMS1
CATSPER3
B4GALT6
TYW1
DBIL5P
SMARCAD1
ARL4C
DDX19A
CACTIN
ARPC1B
RBM12
USP18
KIAA0930
BIVM
FLVCR1
NR6A1
HIKESHI
UFM1
RPS9
LINC01003
WDR60
AKIP1
APLNR
NOL9
DMTF1
C16orf70
STAM
PRR3
MIR6732
NCOA6
MIR21
DCAF1
CSTF3
RINL
SPATS2
TPRA1
CCDC22
TIGAR
PLEKHH3
CTBP1-AS
LTV1
WRNIP1
U2AF2
SLC4A7
LCAT

SAMD4B
WRAP53
PSMD4
DBR1
ZNF484
FAAP24
PSMD12
CHST14
NME2
KDM3A
WWC3
NARS2
NUDT4
DLG5
SLC2A4RG
TMEM259
BATF3
TRRAP
SPIRE2
KCNE4
VKORC1L1
MIR6878
STK25
TJAP1
COPG2
APBB2
THADA
LOC153684
YAP1
IGSF8
LRRC32
ZNF260
SLC7A6OS
TP53BP1
VPS72
TYK2
GJB1
C21orf58
CLDND2
AAR2
ZNF444
SARNP
RRP7A
C1orf174
MICALL2
RBM33
EIF1AX
STK38L
STT3A
PRRG1
UNC93B1

CAPRIN1
EPPK1
CPSF6
CPT1B
GRK3
ING5
GTF2H3
ADPGK
MRPS35
TAF10
NDUFAF2
FMR1
CSGALNACT2
TMC7
NEDD1
NBPF20
PDCD6
ZKSCAN1
NRAV
SARS2
TMEM261
ZMYM5
PAXBP1
AKIRIN2
TAGLN2
CARD19
RBFADN
RPL37A
PDIA6
CCDC71L
LOC100268168
NOL11
ATP6AP1L
SNRNP25
TAP1
ZZZ3
PFKFB4
DGKG
SMARCD1
C19orf73
ZBTB45
WDR45B
STX2
PSPC1
HEXDC
PRPF40A
FAM193B
ZNF589
CCNT1
POM121
GNL2

NOL10
LINC00094
NFATC3
SELENOO
TSGA10
FASTKD2
FAM20A
TTC41P
SNRNP40
MAFG
LOC101930071
TRAF4
CRAMP1
NPIPA1
LINC01237
PLBD2
H2AFY2
CIAPIN1
PMS2CL
HPCAL1
ZDHHC4
ERH
SERINC3
IL15RA
GSS
TCAP
LOC101928160
FAM49B
HERC2P9
WDFY2
SRSF10
DPM3
ADORA1
SMARCD2
FARSB
CACNA1F
CDK16
GDAP1
RPAIN
SERPINB9
FAM199X
ZYX
MORN4
C17orf75
BAZ1B
USP49
C9orf72
PHLDB3
GTF3C5
NINJ2
SSRP1

ROCK1P1
PABPC4
NCAPH2
PGAP2
JPX
ZNF852
STKLD1
BHLHE40-AS1
CHSY1
RPL7
DCLRE1C
RBM3
CNTNAP2
FAM220A
METTL13
ADAMTS13
ATP9A
PTMS
EBLN3P
CD200
VANGL2
C2CD2
MAD1L1
CDH13
SNRPG
ATG16L2
NUDT21
PXN-AS1
SLC7A8
ZNF77
PPIF
EXOG
MIR632
ELK4
ZC3H15
NPAS1
NCDN
FIBP
SLC25A10
DDRGK1
GALNT4
TUBE1
TRIB2
AGK
RNF213
C1orf56
SRFBP1
SETD1A
TTC25
FAM136A
TUBGCP4

MYLK-AS1
PKD1P6-NPIPP1
HS2ST1
GPR68
NFS1
ADO
SLC25A36
EIF3I
AMPD3
NFYA
SENP5
CCDC15
PGM3
ZNF628
ADAM9
CETN2
SMAD5
SEPT7P2
MIR7107
TIMM23B
ZNF621
GAS2L3
ZNF668
ZNF281
SLC16A13
CARHSP1
ARMC9
HSPA1B
SRGAP2
CA5B
SNRNP48
BDP1
C22orf24
POM121C
C11orf49
MRPL37
SLC35B4
MMS19
GTF3C4
ARFRP1
ARTN
VMO1
ZNF30
VASN
LOC105376736
ATP13A1
CHDH
SRD5A1
DYRK4
ZNF286A
PAQR7

G2E3
MIOS
FAM208B
ZBTB41
EIF3M
EIF2AK2
IKBKB
ELAVL1
TUBB
RPLP1
PRKRIP1
CYHR1
IRF3
TMCC1
TBC1D7-LOC100130357
MIR6727
MEX3B
EIF2B3
VAC14
ZMYM3
FOXK2
SNRNP200
DAP3
INO80E
LOC100507291
METTL5
NUP62
GOLT1B
CACTIN-AS1
STAU1
RPS7
RPL35A
LMAN2
YBX3
USP39
MIR6831
LOC105369635
IRGQ
TMCO6
SNRPB2
NENF
CORO7
MOCOS
MIR1181
CHKB
MED10
ZNF584
ZNF620
MRPL9
ZNF33B
XRCC1

ZNF670
TNFRSF25
GCN1
PPP2R2A
TRIM73
ZNF282
ERCC3
SPRN
LINC01184
WDR19
FBXO45
PSMB2
IDI2-AS1
PTPRN2
KBTBD2
PTCH2
ZFP90
SLC15A4
SEMA4D
RABGGTB
ZNF765
SOCS7
CD47
HNRNPD
CCDC51
DCLRE1B
PEX5
GSE1
AATF
RGS12
IKBKAP
HCFC1
BMS1
DNAH1
RPL38
AHSA1
DUSP12
KANSL2
RUFY4
PSMB1
LINC01176
DEK
ZNF273
MAP3K8
MAP1S
RAB31
LINC01125
YRDC
SEMA4F
DTX2P1-UPK3BP1-PMS2P11
AGBL3

RINT1
SPATA13
CEP89
SLC26A1
ILKAP
USP11
ZNF526
NUDC
TTC27
MIR7162
C14orf37
AP1G2
KARS
POGK
MIR4745
C14orf80
NFKBID
SYMPK
CCT8
AASDHPPT
USP37
SRRT
POLR3H
MRPL23
MAGED2
HNRNPH1
TNRC18
ZNF250
LOC100289361
RPL30
ENPP5
MIR378J
INTS6
RPL3L
SNRPC
GPX7
HN1L
TAF2
CCDC24
PIH1D2
HSD3B7
HDAC7
FHOD1
RNF103-CHMP3
ARMCX3
TRAF2
FZR1
PSMB5
PSMD7
RBM12B
MCUB

TMEM234
C2orf16
NPRL2
KANK1
UHRF1BP1
SIL1
FAM98A
DCUN1D3
TSPAN9
FBXW8
ARID5A
NUDCD3
PARP10
DIAPH2
MYRF
PPME1
SEPHS1
PSME4
EMG1
SRSF9
GHDC
PINX1
CAPN8
PSTK
ANAPC5
FAM86C1
BSPRY
C8orf44
IFRD1
PPP1R3D
DDX41
MIR3940
TSEN54
SH3GL1
UTP6
NXT2
ZXDB
FAM78B
FIS1
ZNF565
CCDC167
ALG1
MIR3198-2
MIR3653
REPIN1
ATXN2
WDR31
TPP2
GATC
INTS2
MTDH

TP53
ITGB3BP
ZNF84
ZMYM1
ATP11C
SRPK3
KLF16
RBBP7
RSRC1
LAMP3
BTAF1
NSUN6
XPO1
MOGS
PITPNB
HIGD1B
FCGR2A
COL15A1
N4BP2L2
CCDC183
POLE
ITGB4
MPHOSPH9
ZNF785
CRB2
SAAL1
RGS9BP
CHMP4C
LYRM1
FKBP11
MDN1
RNF44
SLX4
FUT10
SETD5
CDCA7L
NSMCE2
LOC101928238
ECM1
TOX3
SEC13
MON1A
ZNF566
PDGFB
EIF3D
DDX54
FAM133B
PROC
SNRPA
ZNF490
AAAS

C12orf10
CEP135
C15orf61
RCN2
HMGXB3
TMEM205
FAM210B
BTBD2
GPC4
PCGF1
TALDO1
TTC12
PSMB9
ALG5
PCAT7
PCYOX1L
POP5
NABP2
LMNB1
MSR1
AVEN
TMEM266
ANKRD22
GTPBP6
GTF3C3
HOXD9
LOC101928659
NCBP2
PRR16
RNF8
HAUS2
OTUD3
PGD
RAD1
WDR59
ARHGEF10
SRSF6
ARL5B
RFX7
TCF20
TRMT2B
FGFBP3
PIGQ
ALDH1B1
YWHAG
STT3B
HMGB3
MIER2
NDC1
ZNF680
WDCP

DNAJA1
FANCL
IQCB1
CHCHD2
NR2C1
TIMM44
AGAP9
ILF2
MCTS1
ZFP30
ZNF276
ERI1
ZNF674-AS1
DOHH
ATP13A3
3-Sep
NEU1
ATP13A2
DALRD3
EIF3H
LARP4
TRIM24
METTL2B
LINC00857
MIR6844
RPL10
RPS11
U2SURP
CHCHD5
TYW5
LOC100130357
CETP
GOLGA6L10
MRPL38
DFFA
STK4
MKS1
TAF1
PRDX2
PIK3R2
TDP1
TFRC
SH3TC1
KMT2B
C21orf59
ITPA
LYPLA1
PPP1R32
CD82
MDK
ZNF782

ZNF551
RPL27
RPA3
TMEM204
MZF1-AS1
TMEM189
WDPCP
YARS2
ZNF789
B4GALT2
DCBLD1
LOC100506302
TMEM163
SLC41A3
HOXB9
TRIM2
DENND4B
LINC00622
POLR3F
NR1D1
FKBP9
PM20D2
DGKE
UHRF2
C11orf45
KIAA1958
ANKRD19P
UQCC2
FBRSL1
PXDN
MIR6886
LOC101927420
HOOK3
C1orf53
PPFIBP1
ZNF574
SHANK1
FAM189B
UBL4A
AGAP4
THAP10
PCDHB10
ABCC11
ANKLE2
STRAP
ZNF295-AS1
ZNF714
LOC102724094
TAF1B
MLH3
SUGT1

RPUSD2
HDHD5
DZANK1
SLC25A19
LOC100130691
INTS4P1
C1orf204
MANF
TFB1M
ZBED8
PFKM
DCAF16
ALS2
ITPR2
CDC123
ERMARD
CEP85
LEKR1
CAPRIN2
KNOP1
YAF2
SLC26A8
MMAB
SEC24B-AS1
SDC4
MST1R
TCF19
EXOSC6
CSTF2
RAP2A
GTF2E2
MRPL50
SEC23B
MIR4647
TMPO
ZNF777
AATBC
DUSP23
MXRA8
ABL2
LOC105374952
GCSH
ZNHIT6
ZNF443
BORCS5
ZNF736
GALNT18
SYNCRIP
LSM4
MIR6741
RNF187

RAD9B
ADAM15
CCDC74B
EIF3C
AZIN1
VRK1
LRRC49
UMPS
PCGF2
DPY19L2P3
ADM5
APEX1
SF3A2
RNASEH2C
ACTRT3
CROCCP3
FCHSD1
ZMYM2
CLN6
SELENOI
UQCRHL
CACYBP
FAT1
FAM86DP
MIR6733
MMP24
POU2F1
TMEM121
PAPD7
LPGAT1
IDH2
TAF1A-AS1
IFT52
MCF2L
PROSER3
RHBDD3
C5
GMEB2
COX19
SEC14L1P1
FAM207A
MED30
SCN8A
AKAP11
DNAJC9
DIMT1
ENTPD2
CA5BP1
KRI1
MRM2
TYSND1

MTMR1
PRPF3
MMEL1
TCTN2
SDHAF4
CENPT
POLR2H
CNPY2
IQCC
MIR6845
TPST2
CEACAM4
PDK3
LCA5L
RPL13P5
MDC1
AKAP17A
RAI1
CROCC
EIF4A3
GAR1
MTRF1
C17orf51
INTS6-AS1
COL4A2
DUOX1
PARP6
ALG10
ZNF623
RPL29
SASS6
B3GALNT1
FMNL3
TUBGCP6
FAM110A
UBQLN4
CCDC137
MDM4
ZNF890P
TDRKH
SNRPD1
SAP30
SURF2
CLASRP
PTCD2
CCDC124
TRIT1
RHOT2
FKBP4
RBAK
OCIAD2

SH3RF3
ELK1
KIRREL
C15orf41
SLC41A1
ARMCX5
CCDC77
LOC101927635
STK26
STOML2
USP13
SUPT20H
ZGPAT
ZFP41
SLC39A3
PHF20L1
LOC645513
TOMM20
NEDD4
MFGE8
OSBPL8
REXO4
BAG4
NNMT
KEAP1
POLR3A
AHR
MIR943
PHF8
SPAG4
RIOX2
DDX49
FAAP100
TMEM17
FAM81A
THAP9-AS1
NARF
ACP1
LSM5
POLN
TCERG1
GBAP1
ZNF597
CBWD1
LRP5
DEF8
SLC39A13
AKAP8L
CDC42EP2
PPP1R2P3
LIN9

FOXN2
MGRN1
UTAT33
PJA1
CDK12
GEMIN8
EFHC1
FANCE
CLNS1A
CCDC183-AS1
AP5Z1
LINC01970
HUWE1
TSSK6
MIR3682
LOC100133091
ZNF92
COL16A1
MRPS24
ITGA6
HNRNPA1
EFTUD2
RFT1
HIP1
R3HDM4
PSMC2
CELF5
RPLP2
C11orf1
PWP1
MED27
JMJD4
WDR73
FAIM
NUPL2
LRRC45
NCAPD2
LOC400499
ZNF517
E2F4
SLC22A1
GNPNAT1
NDC80
RHOBTB1
BICC1
BCL11A
GPR89B
LOC105371824
ZNF48
TSC22D1-AS1
HPS4

OGFOD1
LINC00888
MIR1244-1
LRRC2
MATN1
QRICH2
LOC100130370
SLC27A1
ZNF146
MORF4L2
ABCF2
ATXN2L
PAAF1
CARNMT1
SNTN
EEF1AKMT3
ELOA-AS1
WDR13
LYRM4-AS1
ITPRIP
STX10
MNAT1
FAM111A
RAD54L2
CFAP69
ZDHHC24
SCNN1D
ORC5
PNPO
G3BP1
TIGD2
HSPB9
NAE1
IRF2BP2
IPO9
WDR83
KCNC3
ZNF3
LINC00398
ZNF431
MYO19
MAMDC4
LMNTD2
TMCC1-AS1
PRPF19
ZNF32-AS1
CEP95
CNNM3
GPN3
METAP1D
MUTYH

RASGRF2
LRRC58
IL17RD
C1QBP
CMTM2
SYNGR2
LRRC37A6P
SMG9
ALS2CL
UQCC1
LOC105370941
TUT1
PDCD2
TLR2
SPNS1
ADSL
NUP160
LOC102723566
OTULIN
SPIRE1
IRF9
CCDC14
C20orf96
BOLA2B
SMPD4
LOC100506136
NKTR
DHX35
SKIL
COMTD1
LOC100505585
HLCS
RNPS1
UTP23
TMEM267
METRN
SLBP
GVQW2
LPIN1
MIR5581
MPV17
CDR2
MROH1
C12orf75
INTS1
SFI1
SLC16A10
RPARP-AS1
MAPKAPK3
LETM2
SEMA4C

TOPBP1
TMPO-AS1
GRHL2
POLR3C
CCDC120
CCDC122
PTPDC1
FAM86C2P
TMEM187
CSAD
DUSP7
QTRT2
CCDC157
HDAC2
USP35
POLE3
GLCE
STPG3
FZD6
WWC1
PNKP
THNSL1
ZNF93
DDX18
TMEM150A
CDKAL1
TMEM200C
PFN4
ZBTB25
DDX28
ABCA2
ZNF770
LOC105372482
FOLH1
FAIM2
HUS1B
JRK
QSER1
NCKAP5L
EGFLAM
PHB
KLHL25
CNPY3
ZGLP1
SLC29A3
LIG3
RBMX
AXIN1
DHRS13
SLC25A39
AUTS2

TANGO6
DEAF1
ESF1
GPR89A
PELO
TMEM79
FAM72B
MIR6836
DNAJC10
PAXIP1-AS1
C7orf50
FOCAD
SUPT3H
ANAPC1P1
MYLK2
MIPEP
RAD51
NUDT5
LINC01503
ALG1L9P
GRWD1
SMS
MIR621
CCT6B
DCLRE1A
FANCM
BOK
PCBD1
STAMBPL1
FAM221B
RIF1
E2F8
POC5
AGTPBP1
ZNF133
LARP1
HOXA11
C1orf159
ENTPD1-AS1
PXYLP1
MSH6
PNN
CSGALNACT1
GOLGA6L9
MRPL58
GDPD4
LLGL1
GSTO2
PAN2
RBM26-AS1
HSF1

SELENOH
PMPCA
LOC100506098
EEFSEC
CRACR2B
SRGAP3
PI4KAP1
WDR36
SNHG5
DNAJB1
FAM225A
CUL7
SLC29A2
MTG1
MGME1
POMGNT2
ZNF771
DCST1
RPL7L1
SSB
KLHL3
KIF3A
LOC105371730
GUSBP2
TRPC4AP
LUM
ZNF195
RPL26L1
SUGP2
IPO7
NEURL2
ZNF7
PML
ATRX
CNOT9
UBE2I
MIR614
SMC2
EIF3G
AGAP5
SNRPA1
CYB5B
CHN1
NOX1
LOC653712
RIOK1
UBE2V1
NSMCE1
FXN
TMEM216
PDIA4

PSPC1-AS2
TMEM184B
EPB41L2
IRAK1BP1
FAM161A
ZNF142
TMED1
IFT81
ZWINT
RNF113A
MFSD13A
RHEB
HRAS
NPAS2
ST3GAL1
LOC105372476
POTEE
INPP5E
ILF3
SCRN1
PIGL
SULF2
PSTPIP2
WEE1
FBXO22
LYRM4
CFAP20
TPM4
TESMIN
GPATCH2
WDR76
DDX39B
RNASEH1
ZNF263
NCOA7
RRP15
HCN3
C10orf10
CCDC3
YIPF2
HERC2P2
METTL3
IPO11-LRRC70
RGS14
CASP4
FAAH2
PPIH
GLMN
ELOC
GID8
HNF1A

UBAC2
TLE2
SNHG19
SH2B2
ANO6
CCDC59
ING1
APMAP
MARS2
STOX1
NOTCH4
OGFR
TIA1
ADCY10P1
CENPQ
POMGNT1
CASC2
GHRLOS
PTCD3
TMEM123
GRK2
CRISPLD1
CEP41
CHKB-AS1
RBM19
ZNF587
PFKFB3
TIMM10
CTD-2201I18.1
TRAPPC2L
C2orf50
WDR5
RAB22A
SRSF7
DHX33
ACOT9
ANAPC1
XRN2
NBPF9
PKN3
IFITM4P
PIBF1
DYNLRB1
KBTBD6
FAM86B2
PRPS2
ACLY
RFC5
CRLS1
RPL32P3
MTA1

MAK16
IL17D
SMC4
GPRIN1
MAP3K10
STRC
CRYBB2P1
NEK3
FNBP4
TOMM5
TAF4B
C16orf95
LRRC14
ZNF215
POLR2J3
PKN1
LSM14B
SLC2A3
GPX8
MX2
FAM219A
C8orf33
LY6G5B
TMC6
DTX2
NMNAT3
UPF3A
MFSD10
TTL
PKP4
COPS6
TMEM237
SACS
NCL
AARSD1
MIB2
YIF1A
TMED3
RASL11B
CATSPER2
RILPL1
YIF1B
ASCC3
CDC16
EPS8L1
FAM91A1
ZNF805
PAK1IP1
UBA6
ZNF202
ZNF267

PPM1G
PDZD8
CLEC4E
ZNF432
POLRMT
EARS2
PLA1A
ZNF486
NOP53-AS1
CAMKK1
GEMIN4
RMI2
LINC02210
CD99P1
BNIP3
FAM19A1
EPSTI1
SNORD14A
HSPBAP1
NCLN
G6PC3
LOC284023
ADGRF4
DNHD1
RPL37
PLAUR
GIGYF1
SEC31B
RPRD1B
HKDC1
LOC283922
RAD51B
INTS8
LOC101927811
RPS4X
RPL18A
GIT1
TAF1C
ASPN
DESI1
C2orf81
DICER1-AS1
UBAP2L
SPOUT1
C11orf72
FLAD1
RPF2
MIS18A
PRMT5
WWOX
NAB1

PTRH2
CAMKMT
GREM1
ACACA
AMDHD2
HPRT1
CTNNB1
SET
TBCB
PROCA1
SNRPE
TTLL10
PLP2
MIR3917
AHI1
MOSPD1
CERCAM
C6orf222
UTP18
ZNF335
LOC399900
POLR3D
C8orf37
DACT1
SCAND1
RNASEH2B
MIR937
NPDC1
PA2G4
NPIPB14P
TSR1
VWA8-AS1
SLC35C2
HMBS
TULP4
RASAL2
LOC105373378
KLC2
RDH10
IFFO2
RFX3
LY6G5C
NME4
TM9SF4
FAAP20
EBP
CYP51A1
TMEM243
SLC12A9
IFI30
RRP1B

CCSER1
H2AFZ
INO80B
DPYSL2
PTDSS1
CHID1
IRS1
G6PD
C4orf46
SLCO3A1
NPPA
ZFP64
RNF216P1
KHDRBS3
MSH2
RPS6
HSPE1-MOB4
ARSJ
ZNF746
MRPL3
DNAJA3
PPIL6
FAM71F2
LAT
CCDC138
ABCC10
LOC171391
KATNB1
RAB11FIP3
RNF126
TSEN15
BRD9
POT1
LSM2
LOC105372233
ROBO1
ZNF449
PKD1L1
PLOD1
NOSIP
LDHB
RNF215
NUP85
C16orf91
RASSF1
USP27X
SAMD12-AS1
SYBU
BCL7A
CMTM1
LOC105370105

FPR1
KANSL1L
PREB
CCDC188
ZMYND8
TGFB3
PHF20
LOC102723385
PTPN12
LOC101928674
IDH3G
APBA3
TFPI2
ERF
CENPO
FSD1L
ZNF618
TMEM39A
HDHD5-AS1
GET4
SSX2IP
CLSTN3
MAP4K4
FAM171A2
ZBTB39
DANCR
YEATS4
CEP78
MLKL
PTGES2
MRE11
LOC81691
TIGD3
CACNA1C-AS2
ERICH6B
SEC61A2
ARAP3
CEP131
C12orf45
GLRX3
EIF3E
KIAA0319
RBM41
PRPF40B
LINC01347
D2HGDH
MIR6873
CEACAM6
CRYBA4
GCAT
LMLN

COL6A3
UBE2O
ODF3B
CARM1
CEP295
KCNMB3
CASP2
COPS7B
ZNF696
BZW2
BCAP31
RAD51D
CDK8
C17orf67
GSTCD
NUP43
SAMD5
OLA1
CDC34
GYG2
PIDD1
PMS1
CKAP5
CCT5
ODF2
PPAN
DLL4
GALR3
LINC01410
RBMX2
UBA52
ZNF107
PQBP1
HSPE1
MIR1825
HRAT92
CEP164
PAX8-AS1
PPP1R14B
CCDC34
NABP1
GEMIN5
QPCTL
RLN2
LOC100128531
RPS28
SLC38A7
ZNF8
FAM118A
STX18-AS1
PPP4C

LINC01772
ICAM1
ACYP1
MEIS3
SYCE2
FCGR3B
CTPS2
TPRG1
ARHGAP27P1
FZD2
CLK2
SNRNP70
PEX10
LINC01607
CAMSAP1
CCDC18
NHP2
BUB3
NOL8
PRIM2
MRPL36
POLR2G
ERCC2
ZNF786
LOC101927592
SH2D2A
UBA2
LOC101927446
PRH1-PRR4
ALKBH3-AS1
MRNIP
CFAP61
CYB5R2
GPR75
ZBED6CL
C6orf1
TBL2
SLC39A6
TUBA3D
SNORD59A
SLC43A3
TMEM154
ZNF717
CCDC28B
BANP
RASAL2-AS1
NUBP2
UBAP2
TMA16
STPG3-AS1
MXD3

DDIT3
SH3D21
DIS3
PPT2-EGFL8
DCTD
PPOX
MTFR1
CPSF3
CEP152
GRK7
PDE7A
GADD45GIP1
RFC2
C12orf29
TMC1
MYDGF
DLGAP4
TSSC1
SRPK1
IMPDH2
SLX4IP
P3H3
GADD45G
CCNE2
NEK8
ADM2
EEF2
SYCE1L
NORAD
REXO2
RBM14-RBM4
RP9
INSIG1
IER3
SRPRB
ZNF182
TNPO2
PRMT7
R3HDM1
ADCK5
SYNPR-AS1
WRAP73
LINC00649
IDUA
LOC729603
FAM50A
INCENP
DNAJB13
GABBR2
ENO1
GAS8

CDK5RAP3
MIR616
GAPDH
CES4A
TOMM40
ZNF703
SLC1A4
ARGLU1
SPAG5
MVD
NLRC5
DPM1
BCS1L
ZSWIM1
TMC2
DHX30
CREBZF
CENPL
NT5C3B
LINC00937
DEPDC1
LOC105372419
SSBP1
ATP8B3
NUP35
CELSR2
NMD3
TSPOAP1
LOC101927204
LINC02159
LOC339666
NIT2
UPF3B
TIMM13
ANKK1
HELB
ZG16B
THOC6
LINC00839
INHBC
OLFM2
LINC01771
FBXL8
THOC3
KYNU
CAPG
CCDC84
SBNO2
TTF2
KIF15
NOTCH1

SPATA5
TMSB15A
EEF2KMT
DGCR6
UBFD1
NOP14
PUF60
KIAA1755
FKBP14
PHF14
CCL4
TASP1
SLC9B1
POMP
C1QTNF6
STIP1
LOC729867
MIR3685
RPL7A
ASPH
B4GALT7
ANGEL1
LPIN3
PDXDC2P-NPIPB14P
ENKD1
ENTPD6
FUS
DRAM1
PIGU
SMARCA4
FARSA
RCC2
NUP37
SLC1A3
KCNAB2
POP7
POC1A
HS3ST1
UCHL3
TUBB1
EVA1B
ARRDC1
NUP93
DRAXIN
GGCT
HK3
UAP1L1
ATF7IP2
CHEK2
SGO2
BMS1P5

MIR4649
IFT140
PRPF4
SRD5A3
RFXAP
CHD6
TACC3
ANKEF1
TRIM16
CSF3R
LOC730102
TRMT2A
IL17RB
ELOB
SPIN2B
DEGS2
FAM151B
ACSS1
PGK1
CUL4A
PIP5KL1
MORC2
XAB2
KIF27
ANKS3
FN1
RSL1D1
SAP25
DPM2
DDX51
HNRNPA1L2
SMARCC1
RPS18
LOC102724323
LOC101929718
MIIP
COL18A1
NAA15
MEMO1
OBSCN
CCL4L2
XKR6
ATG101
UBN2
ZFAND1
FBXW9
GTF2IRD1P1
LOC105371397
GDF11
ACAP3
CSNK2A2

DXO
ANO8
FAM58A
OSBPL10
TAS2R10
TRIM65
PCBP4
ALDH1A3
BBS5
LAMC2
ACTL6A
DPH6
MSTO1
COA1
ZNF200
MRI1
TMEM120B
POLG2
NECTIN1
ZDHHC8
CNTD1
MIR4758
POLR2D
TCTN1
LINC00638
PDZK1IP1
GOLGA8A
RIBC1
ZNF354B
PITPNC1
MIR1257
YEATS2
DFFB
CCT6P3
PPP1R1C
BCL2A1
DARS
ZNF503
CLTCL1
STOML3
LPCAT2
FAM217B
ADGRE2
PLPPR4
CHML
MRPL57
FAM83D
NIFK
ZNF205
EIF6
FCGR1A

MSANTD3
RACK1
KLF7
CDC42EP1
IMP4
ZNF792
PLD6
AP4B1-AS1
OGFRP1
MDS2
USP36
LOC101927905
TIPIN
RAET1E
UTP15
LSM8
NOC2L
MALSU1
PAXIP1
LINC00471
FAM183A
MIR6132
CDKL3
THOC2
ICAM5
NRIP3
RPS14
RPL12
ZPR1
WDR5B
CTNNBL1
SESN1
TNFRSF9
MFSD14C
ZNF23
IFT22
NBPF8
RPS19
INTS7
RAPGEF4
BCAN
BTN2A3P
VWA1
LINC00562
XRCC3
PSMD14
FBXO5
PLXNB1
LINC01063
NAT6
EP400NL

CECR3
DCPS
BST2
RASAL1
MAP6D1
ZNF343
ANKRD23
ABCA13
GPC1
RPIA
BARD1
LIPM
PES1
NDUFAF8
FBXL19-AS1
SHISA5
LRRC38
EMC8
DNAJC3-AS1
LACTB2
RPGR
KRTCAP3
RPLP0
NAT14
RBM26
CCER2
MFSD3
PFAS
ZNRD1
TMEM262
BMP8A
CKLF-CMTM1
TWISTNB
ACTN1-AS1
BOD1
LINC00605
UNKL
NIP7
AFDN-AS1
DIEXF
TMEM165
OXLD1
E2F6
POLR1A
LOC105370027
ZCCHC7
TBC1D8
PTDSS2
NPIPA5
C1QTNF3-AMACR
RNF114

MIR4258
ZFC3H1
CFI
PLCH2
TFAP2C
FAM24B
SLC25A14
EEF1E1
BCAS4
GPATCH4
DNM1
B3GALNT2
IQCA1
DONSON
GMPS
RNASEH1-AS1
NEU3
TMEM182
SAMD14
C20orf24
CDK5
BUD23
LINC01136
GZF1
PIPSL
GPX4
ASAP1
ISLR
NEIL2
AGAP6
LOC100129917
C1orf198
CSPP1
ARHGEF28
C19orf53
ZDHHC1
DGKZ
SMYD2
PRKAR1B
CCT7
MIR429
LINC01816
MIP
MURC
E2F3
FBXO43
ZNF32-AS2
AARS
GOLT1A
DPY19L3
GPR176

BAIAP2
ETV7
RPL35
KCTD13
GPALPP1
ZNF341-AS1
TBC1D31
LBH
NUDT8
ZNF767P
IGFBP7
FITM2
NPIPB15
SIVA1
PATZ1
C9orf142
PDDC1
LINC02166
FBXL2
TCTEX1D2
CENPU
GPR35
SNAPC4
FOXC1
ADNP
SUN3
PRIM1
BORA
ARSI
METTL12
GTF2F2
ZNF572
PLEK2
PCLAF
WDYHV1
LOC101929574
MICB
POLR1E
PARD6B
GPX1
SNHG21
PRRX2
CDK10
CASTOR2
C1orf35
LINC01006
OPCML
B3GALT6
LOC102724814
NPM1
NDUFB2-AS1

MIR4453
NUP107
ST6GALNAC5
C9orf16
ENOX2
HAVCR1P1
MRTO4
KCNE3
TULP3
DNPH1
ADNP-AS1
UBALD1
LOC100128568
P4HTM
RPL36A
DUS1L
PALB2
SPTY2D1-AS1
LINC01686
MYL6B
ETS2
ZNF391
DHX37
ANAPC7
LYPD6
TRMU
FLJ45513
MB21D1
DUSP18
TIMM50
TRMT11
DLEU1
PAK6
TBC1D4
METTL22
TEX29
MBTPS2
FAM86JP
CARD11
NEK6
EMD
GSAP
FOXK1
TRMT61A
DNAH6
POM121L9P
POC1B
ABCE1
CCL7
CLCF1
RGL2

CARD9
RBM39
MIR3191
BEND3
TIMM17B
AFMID
KRT18
USP6NL
TGS1
CALU
COLGALT1
C10orf67
POLA2
UTP20
GJA4
ZNF581
PDCD11
PSMG3
AK8
CPOX
LOC728554
ANKRD39
CSNK2A3
WDR3
ZBTB18
TNFRSF4
TGIF1
MIR4326
SLC4A2
L3HYPDH
CD80
ARHGAP11A
SLC19A1
NAA20
TPM3P9
HSPBP1
LINC00441
CFAP65
STK36
PORCN
FAM86B3P
LIG1
FAM72C
LINGO1
PFDN4
PLEKHA8
NUP188
MTERF3
TAZ
CFAP97
NPIPB4

SYCP2L
DDX43
FAM95C
RAET1G
KNDC1
ZNF514
NEMP1
DYRK3
TUBG1
ZNF761
LOC440700
ABHD2
OSBP2
PLK3
ZNF316
SMIM1
CCT4
ADAMTS9
LOC105372440
LOC100506083
LOC102723692
TBCD
NR2F1
CAPN10
PARP8
PRICKLE3
SCARB1
STK3
LOC102723517
ANKRD10
MRPS2
ERO1A
GPX2
CHAF1A
NUP205
IMMP2L
ENO3
LOC101929633
TBRG4
ZBTB33
SMIM3
SHC2
LRCH1
ZWILCH
CCT3
NKAPP1
LOC101928464
MIR5090
DCUN1D2
PLK4
CABLES2

RPUSD4
TMEM241
RGMB-AS1
PIWIL4
PLCB1
SNHG9
MAPK8IP3
KIF20B
CENPK
SRXN1
SSBP4
PSD2
RPL13
NPIPB11
RASGRF1
MANEA-AS1
RAB26
FAM122B
FUT8
TDRD5
TRIM27
MIR24-2
RNF219
CHST1
TBC1D24
NUDT14
PLA2G16
LINC01138
PUS7L
ZHX1-C8orf76
MRPL4
C12orf66
PACSIN3
LOC102724009
RPS3
SH3PXD2B
FAM84B
APBA2
ATR
HDAC10
GUK1
NONO
LOC285095
ARMC6
LOC105370681
ALYREF
CLHC1
MSL3P1
CSTF1
SP2-AS1
MIR6758

FAM120C
AMOTL2
PNPT1
ACP6
LRRC20
OIP5
MARCKSL1
TBXAS1
EIF2S3
MCM6
TIMM8A
RPL18
C15orf62
C12orf73
CBWD5
POLB
MIR484
MASTL
PPM1J
C1QTNF1
G0S2
LOC728084
P3H4
UBL4B
TBX3
FOXD4L1
ACSL4
TRABD
RNF148
TIMELESS
JMJD7-PLA2G4B
ATP7B
ANTXR1
ADAM19
LOC105371814
PHF6
FPR2
CLUHP3
NMRAL1
BIK
SLC39A4
HCG25
KLHL11
ICA1
MCM3AP-AS1
MIR7705
ZRANB3
RABEPK
AP1S1
DPH2
LUC7L

OSER1
GNA15
CABP4
WDR35
PTCD1
ALDOC
RANBP1
ATIC
RNF207
EXOSC3
MRPS23
IL5
NUP155
GINS3
DPY19L1
DDX47
IL27RA
CLDN12
PGP
SNX10
LOXL1
RNF219-AS1
ZNF587B
EIF4A1
LOC100131347
IFI44
SLC19A2
DCAF15
MNX1
FSCN2
KCNQ1
ADAMTS4
TMEM185B
CCL18
C1orf109
MIR3677
HEYL
RPN2
HOXB-AS3
SMAD6
NUP58
NOC4L
BRAT1
ESCO2
AHSA2
MIR1254-1
MAGOHB
TRMT6
PUSL1
ZNF783
RAB3D

FEN1
HSD17B7
ARHGAP4
HENMT1
ULK4
ZC3H8
NBPF15
PGAM5
CD1A
TUBGCP3
LOC105372493
SAC3D1
FTCDNL1
SYCP2
TMEM41A
EHMT2
CEP83
ANKRD16
POLR2L
CCNJ
POU5F1P3
CDC7
RCCD1
C11orf95
FAM225B
FBF1
SLC16A4
FAM83H
H2AFX
SDF2L1
GGH
CELSR3-AS1
MIR6838
TPD52L2
TRIM17
CPSF4
FAM216A
TTLL3
WDR34
ZNF251
FAM221A
PBK
ATXN7L2
SNORD7
GBP6
TGM1
MIR6808
PAN3
LINC00449
CCDC170
ZNF525

RNF24
ZNHIT2
ZNRD1ASP
CLCN7
LOC150776
LOC101929657
9-Mar
PSMC3IP
MKI67
SNRPD2
FBXO44
P4HA1
TAMM41
USPL1
PAM16
C2CD4D
EBPL
ASXL1
CBWD6
DRC1
ARMC10
GNL3
HOXB3
MSC
FLJ33534
CLEC11A
RBCK1
ORC1
TTPAL
MATN1-AS1
CHORDC1
TMTC4
LPAR2
MLST8
TFDP1
ASIC3
ANKZF1
RTKN2
CCT2
SSSCA1
CACNA1E
RBM44
ZNF185
SPRY4
FDXR
WDHD1
LENG8
KRTAP5-10
WDR12
CHRNA5
TMEM231

CCNL2
ARL6IP4
ALG3
NAP1L1
TRMT112
C6orf141
SF3B3
NAA16
LOC148709
ACSBG2
SSUH2
OSTCP1
LOC100507487
AP3M2
CCNI2
LINC00240
AMMECR1
TXLNG
ATP6V1F
DIO3
OAS3
PLXDC1
TAF7L
BRIX1
BLM
PDCD5
PRKACG
FUT7
IGFBP2
TSNARE1
LOC105376430
CERS6-AS1
CENPM
ALKBH6
RPL23P8
DLK2
PRPF6
NTN5
C4orf47
CXXC4
AGO2
CAPNS2
IMPG1
ELOVL5
PNO1
CXorf65
PEMT
RPS16
SPDL1
LINC01126
RCL1

RDM1
SNHG8
ST3GAL2
DDX10
TCHH
SHC3
TARBP2
NOL3
EIF2S2
STX16
ENTHD1
FANCG
TMEM201
LINC00282
POLR3E
SAMD15
C19orf71
OSCAR
CCDC57
LOC100134368
ACTR5
LMBR1
ESPL1
DUS4L
EEF1AKMT1
ANKRD13D
ASPSCR1
ARSE
FAM156A
ZNF252P-AS1
SLC25A15
SLC25A32
WDR75
ZNF337
SGK494
ACVR2B
DDX56
CCNB2
KMT5C
PTGER1
SPICE1
COL24A1
CEP250
DGKH
AMER1
INO80B-WBP1
SS18L1
LOC652276
CXCL16
CENPJ
ZNF497

TBX6
MRPS34
FAM19A3
THUMPD3-AS1
SEMA5B
GINS2
DPH7
YTHDF1
PSMG1
LOC389641
WDR18
THUMPD2
NTM
GNB3
MRPS12
RPL36
PNPLA1
MIR3178
DNMT1
CPZ
MIR3164
CKS1B
ERGIC3
LHX4-AS1
BICDL1
UFSP1
LOC100506127
DNAH17
PRPS1
TCF3
KRT19P2
HSD17B10
GRB10
MIR4329
TUBA4A
ZNF117
SCML2
NAT10
ZNF26
TMEM190
DISP3
MIR4754
CCDC73
LOC400927-CSNK1E
OAS2
ARFGEF3
MCM5
COL9A2
HTR1B
PVR
GLA

HAR1B
QPCT
EPDR1
MRPS26
URB1
SKA1
RANGAP1
TCOF1
CDH24
RP9P
PKM
LOC100288637
MAPK12
LINC00299
ZMIZ2
ABLIM2
ENOPH1
PDP1
KDM1A
POLR1D
ZNF473
MIR924HG
LOC105374989
ZDHHC11
LOC145694
CDK5RAP1
MIR196B
E4F1
TTLL2
NPHP4
C22orf29
PODXL
CAPS
COL26A1
LINC00174
ATP6V1E2
C19orf24
COLEC10
CBX3
LIMK1
ZNF598
RAN
ANKMY1
ANKS6
APOC1
SKP2
KIAA0907
TMEM9
EXOSC8
LINC00115
CENPW

PTGS2
POLA1
RAD18
PLK2
ERV3-1
CABYR
OR10V2P
VPS37D
PPIL1
MIR1539
SMYD5
LRP11
FAM111B
YAE1D1
C4BPB
SLC22A20
FOXRED2
NSUN5
NOM1
FARP1
KNL1
PCMTD2
NTNG2
KNSTRN
NOP16
PPA1
CCDC154
MCM3
ZNF749
ALCAM
CAGE1
RFC4
RNF133
LOXHD1
IL1RAP
ARHGAP33
MYBBP1A
EPHA1
IRS2
ZNF511
TEX30
LINC01068
LOC107984660
CXXC5
NOP58
NRN1
MEIOC
AGTRAP
NAA25
DKK2
DLGAP5

LOC105371849
CD320
KDELR3
PLAG1
FZD10-AS1
CLCN5
LFNG
KRT36
FCRLB
RNF157-AS1
HAUS6
NCAPG
NEBL-AS1
CTLA4
CCDC40
CCSAP
NR2C2AP
ARHGAP39
LOC401286
HEATR1
SHB
POC1B-GALNT4
MCMDC2
TELO2
FMNL2
STAG3L5P-PVRIG2P-PILRB
LINC01775
TTC34
MEST
SHF
RASGRF2-AS1
NOLC1
C11orf16
BRICD5
SYNDIG1
SOD3
DDX27
LTB4R2
FANCD2
CENPP
PRR22
C2orf15
OOSP1
CARS2
FAM166A
POLE2
CPSF4L
LRRC69
PITRM1-AS1
SND1
MIR27A

NFXL1
PUM3
SLC25A6
MPG
LINC00634
CHD7
C8orf34
PDGFRB
SUSD3
NOC3L
TEX10
RACGAP1
DSN1
THOP1
ZNF367
RFWD3
CATSPER1
GGNBP1
ASF1B
FAM96B
PROSER2-AS1
IL4I1
RHBDF1
MGC12916
PTGES
GTPBP4
TBL3
BID
ACRV1
SFXN3
TRIM46
LOC574538
LOC730101
SLC3A2
HSF2BP
MTG2
CDCA8
RBM11
IPO5
MIR26B
UNQ6494
LPCAT1
MIR3186
NID2
CDKN3
GRAMD2
FPGS
RCN3
LMOD3
ARHGEF38
MIR4292

LINC01933
MIR6849
CDC25A
SYN3
DDX55
AMPD2
FAM173A
TRIM28
FGF4
HOXB4
SETD6
DLX5
GTPBP3
SLC2A8
CD46
CCT6A
GPR180
MGAT5
GARS
HSPD1
FCN3
BCL2L12
EME2
RCC1
SLC22A14
LMCD1
FLJ41278
MCM4
XPO4
MGAT4EP
C20orf196
TSEN2
FOXP4
ADRM1
TMEM67
RPP40
VARS
PPRC1
PLA2G4C-AS1
TKT
FMO3
INTS13
WHRN
ATAT1
ODC1
CCDC189
RPUSD1
GS1-124K5.4
PTTG1
KIF9
MIR1178

SPC25
ADAMTS7
NCAPD3
IGFBP3
PROCR
BRIP1
SNRPF
LINC00619
DNAJC2
CCND2
MORN3
CFB
IZUMO4
KIAA0895L
MPIG6B
KCNE5
PLPP2
TPGS1
MIR92B
KPNA2
FBXO16
PCDHGB2
TTK
NHS
LONP1
HYAL2
RPS15
LINC01719
NAT9
EFCAB10
HELZ2
ADA
ZDHHC9
CCNB1IP1
PRSS35
HTR1F
PCID2
PMS2P3
PASK
FBXL6
DNAAF5
LOC102724301
CDC20
ALG1L2
LAS1L
RBM28
HACD3
RLIM
BHLHE40
GAL
DPP7

MIR6805
FHAD1
XPO5
C1RL-AS1
SH3BP4
PRKDC
LTBP2
EIF3B
PCNA
NSDHL
IL13RA2
UGT1A6
HSD17B7P2
LOC101929240
DTD1
IPO4
RAB40A
STK32C
PLEKHG5
GRPEL2
TNFRSF10A
ZGRF1
DPCD
MPO
LRP5L
C14orf79
SLFNL1
SNRPB
MCM8
MIR718
TAS2R5
LRFN4
CLDN6
MIR4784
ELN
ARHGEF39
CDC25C
INPP5D
CTSH
RRP1
AGAP2-AS1
SPRY4-IT1
SMG1P1
TLCD1
NOL6
MIR6850
LOC102724589
NCAPH
TMEM147
CDR2L
ALPK2

C16orf46
LOC101929523
PRMT3
LINC01342
TPD52L1
DDX11
ZNF593
DCAF13
RNF208
TRPM8
ZNF280B
DDX31
NUDT1
MFSD2B
RFX3-AS1
PLS3
MIR3613
LOC101929464
SLC52A2
PRRT3-AS1
ME1
FAM167A
MIR4635
DOCK6
DDX39A
PHF19
ARVCF
DBF4
LINC00866
MOK
ADAMTSL5
CD164L2
KCNS1
NUTF2
LYAR
GCC2-AS1
LINC01638
UBTD1
ROMO1
ZFHX2-AS1
USP31
MID1IP1
PARVB
IL1RL2
RHOD
PAICS
ZNF485
CLCN1
POLR3K
LIPG
CCDC168

SUV39H2
HOXB6
TM4SF19-AS1
CRIPAK
NUSAP1
RIN1
TP53RK
LOC105369302
ERICH6
IARS
ABCC1
PCAT6
POLR1B
LAGE3
MOCS3
FAM71E1
RRP7BP
BTBD11
FSTL4
MXRA5
PXT1
OTUD6B
LOC105378668
TTLL4
AOC2
EEPD1
CLSPN
AMIGO3
KIF24
LINC01664
PRR11
LINC01235
EXOSC2
AFG3L1P
LRRN1
MANEAL
SGO1
INSRR
GREB1
ZNF37BP
QPRT
MIR1247
HGH1
PFDN2
SDCCAG3
MIR6516
RCBTB1
SLC25A22
BRCA1
SMTNL1
FAM60A

LRIT3
CBS
CDCA2
CHAF1B
TRIM31-AS1
LOC105373958
EXOSC7
LOC388813
DNLZ
ALG1L
HAUS7
FNDC1
SMYD3
KIFC1
LOX
LRRC37A3
LOC100507600
HGFAC
LOC644656
HDAC2-AS2
PCED1A
LRRC10B
LOC101929524
XPNPEP3
TTC9B
RIBC2
C17orf47
SMG7-AS1
TTI1
NFAT5
ZMIZ1-AS1
KYAT1
FADS3
MIR6753
COL5A3
ZNF579
HSPA4L
SLFNL1-AS1
ACD
SLC11A2
CTPS1
WFS1
TGDS
PTPN13
LOC100128494
ZNF860
CCL3
DRD4
CENPH
RAD51AP1
DEPDC1B

KRT39
DTL
POLD1
ABCB6
NUTM2G
UBAC2-AS1
TM4SF1
DUSP2
LINC00540
OGFR-AS1
FAM178B
CENPE
ZNF841
TM7SF2
ATP2A1
PLSCR3
STAM-AS1
PLCG1
LINC01287
ARHGAP8
DUSP10
RDH12
FIGNL1
HS1BP3-IT1
BUB1B
SEC16B
RTKN
AGRN
HUNK
MIR4653
LINC00893
LOC100288181
MIR4639
CBWD3
LOC100507406
CLPB
TOP2A
LINC01545
CNN2
SLC7A1
SPATA17
OGT
LOC100129215
LRRC34
KDELC1
LAPTM4B
LENG8-AS1
SPIN3
MCOLN3
SLC30A2
MIR4722

APLF
KNTC1
DKFZp686K1684
FCGR3A
MIR1304
KLF1
LRP3
C1orf112
OR51E1
AGAP3
GOLGA2P7
DARS-AS1
B3GNT4
MMP14
EPHB2
PCDH17
GPR4
PROSER1
CD276
DDX21
ZFP69B
MIR200B
CCDC74A
SLC1A7
POLR1C
MRPL17
PLAGL2
TCFL5
DDX11-AS1
CTU2
MIR7108
ISYNA1
MMP17
CENPF
TRIM59
RRS1
NUFIP1
DBF4B
TNNC2
CECR2
FAM186B
ATAD3A
CTAGE7P
LOC105371049
CCDC86
LRRC61
AADAT
STMN3
RNF144A-AS1
KLHL7-AS1
PRC1

HMMR
MZF1
TBC1D16
NTN3
HM13
CCL20
FAM83A
UBXN11
LINC01569
C9
PDRG1
NEIL3
FNDC10
FOXJ1
HPDL
SLC22A13
LOC104968399
MINDY4
TARBP1
FTSJ1
TUBA8
GSG2
MIRLET7DHG
ABHD14A-ACY1
GNL3L
PARPBP
MACROD1
MIR4664
SLC12A8
LINC00567
UGGT2
CHURC1-FNTB
CITED4
MIR200A
LINC00628
CHCHD6
PYCARD-AS1
PPP1R35
BNIPL
PLXNA3
ZBED2
ZNF697
FBL
KIFC3
CMTM7
LOC101929054
ATAD5
GPR19
ADCY3
THEM6
SLC29A1

MRPL55
LOC101928303
FLJ21408
MSI2
C12orf50
WNT3A
SLC1A5
LOC101559451
KIF21B
C19orf60
COCH
ATAD3B
RUVBL2
SPC24
PNMA2
CCDC33
RPS5
GPR157
BACE2
PCDHB14
ARHGEF19
STIL
TRAIP
KIFC2
LRRC6
TEKT4
SEZ6L2
BATF
TEX22
PARD6A
SCARF2
HAUS5
AURKB
TMEM89
ARID3C
GINS4
ZNF850
KIAA1524
CDH6
SPATA33
RSPH10B2
CMTM8
WDR43
KCTD14
ATP5L2
RELT
WFDC3
GCNA
RPL13AP6
TAL2
SERPINE2

TGIF2-C20orf24
SLC9B2
MROH5
UTP4
UBD
NPRL3
MYO7A
LOC155060
QTRT1
APOC1P1
MIR6832
CYTOR
RBBP8NL
TENM4
TBC1D30
OR7A5
SERPINE3
TREX2
SNHG12
SNHG11
NPIPB13
VASH2
DUS3L
RRH
CENPN
CD1B
SPATA25
PSRC1
LINC00173
NTMT1
NKRF
LOC100507144
EIF5A2
RGL3
VSIG1
FRMPD3
SDR16C5
RBL1
MTFR2
NCR3LG1
GNMT
MIR4712
YDJC
SNHG7
UBE3D
URB2
SLC43A1
LOC101929076
PIMREG
PRDX4
NOP2

TSPO2
LOC101929709
OR2B6
LINC00176
LINC00365
SLC22A15
ANO9
TXNRD3
ADAMTS14
TMEM215
KCNIP2-AS1
SYTL1
MIR3176
F2R
C20orf27
NPEPL1
ASPM
C6orf48
ADAM8
IL17F
SLC6A1
GPC2
C9orf43
LOC105371083
IL1B
MIR7114
LOC101930094
CTTNBP2
IFRD2
SH2D4B
MND1
HIST2H2BC
MAD2L2
P3H1
ANO1
LOC254896
PIK3IP1-AS1
METTL1
WDR74
RAE1
RFX4
DCUN1D5
SUV39H1
GRINA
CAPN10-AS1
ANKRD1
LMF1-AS1
QSOX2
LINC00244
LRP4
IBA57-AS1

ZNF74
LINC00853
POLR2I
NBEAL2
CDK4
POLD2
CYP4F3
TSPAN5
ATRIP
CTSV
TMCO2
LINC01600
DTNB
COL4A1
LOC105369739
PCNX2
FAM72A
IL36G
WDR54
ALKBH2
ZNF878
SMOC2
BUB1
FOXM1
S1PR5
SMG1P7
CYP2S1
KIF2C
GPSM2
KIAA1024
PTPRVP
TMEM52
NFE2
GTSE1
PRSS50
STAG3L5P
TNFRSF10B
LINC00571
XPOT
KIF18A
SLC25A29
LINC01561
E2F5
KRTAP5-2
LOC283194
FBXW4P1
TMEM145
ZNF337-AS1
VMA21
ZFPM1
MZT1

RRP12
CKAP2L
HYAL3
RAC3
LINC01993
TONSL
LOC100506403
CSF3
APOLD1
KIF23
HTRA3
TGFB2-AS1
UTP14A
C17orf53
NUDCD1
RPS10-NUDT3
IL20RA
IQCH
FSTL3
TCTE3
CTRL
PPP1R13L
GPR78
HMGA1
ABHD1
DHX34
TMEM161A
WTAPP1
PCP2
MIR5001
ZMYND19
FAM53A
DCDC2C
SPIN4
ITGB8
UBE2T
DNTTIP1
BICD1
SKAP1
NLE1
LINC01869
POLQ
NACAP1
SLC9A5
CMSS1
ZNF541
MOXD2P
RAB15
CENPA
HMGA2
FALEC

YY2
DCLK3
NCAPG2
FGFR4
C10orf35
FERMT1
ACTR3B
TAF1A
MOV10L1
NEK2
PSMA7
LINC00867
FANCI
SEMA3F
RNF139-AS1
SLC25A27
SSC4D
SGIP1
LIN28A
FAM92A
CDH15
MEX3D
GUCA1A
CBFB
TNFRSF19
DNA2
LOC100506691
SNORD6
LOC105375614
POP1
LPAL2
STXBP1
LOC440028
LOC100288175
DSCR9
PSPH
SYCE3
HS3ST3A1
MIR1292
LENEP
WBSCR27
LINC01376
MINCR
PSMG4
CATIP-AS1
METTL26
EZH2
GMPR
RNF39
SLC35E4
ANAPC11

SHCBP1
BIRC5
SIGMAR1
MMS22L
SLN
CSE1L
ISM1
VEGFA
POMK
NKILA
ZMYND15
DDIT4
RUVBL1
LYG1
RNF32
TNFAIP6
DBX1
IQUB
TSTA3
KIF4A
SERPINH1
NANP
PKD2L2
SLC2A1-AS1
FBXO39
GAS5
PGAM1P5
NME1-NME2
C11orf65
CDKL4
PRR4
KCNRG
MIR6875
SNHG16
ARFGAP1
NOB1
ASNS
IL13
ITGAX
XKR5
PLOD3
MIR1908
PLA2G4D
EIF4EBP1
LOC105370868
GJB3
LTB4R
LINC00114
MELK
TMEM63A
SLC7A6

ARPC4-TTLL3
PERP
ADGRG1
MCM7
ASIP
CD44
CDCA5
CEBPB
LOC343052
FAM169A
KLHL29
LOC101928858
RPGRIP1L
MIR4677
INHBE
KAT2A
MIR15A
CDCA3
MAD2L1
WDR27
RPS2
LINC01012
RAD54L
TTLL9
SPNS3
DEPDC1-AS1
NTHL1
LRRC46
CDK1
LCNL1
NUP210
LINC01814
LOC100129484
SOX12
POSTN
SQLE
ADAT2
WDR77
DNMT3B
TRMT1
LOC100506885
CFAP43
SCX
LOC101928035
CKS2
SPATA6L
CYP4F35P
TRAPPC5
TMEM238
LINC01152
POT1-AS1

C5orf34
TMEM178B
MTHFD2
LOC101927272
PDF
PROZ
LOC105371084
ECE2
SNHG3
CCNB1
ANKFN1
LOC101928402
PLA2G4B
GRIN3B
BTNL9
LOC100506082
WFDC21P
LOC101929224
LINC01762
JADE3
LOC101927762
MIR3606
RRM2
GABRB1
TM4SF1-AS1
LINC00589
OXGR1
DHCR7
SRRM5
MIR664B
TRAP1
LOC100132813
NPM2
AMZ1
ANGPT2
LOC101927620
LINC01273
NSUN5P2
TMEM198
SAMD10
FAM72D
AANAT
RIPK2
GPCPD1
SLC4A8
MIR203A
SCUBE3
RRP9
ARMC2
CKAP2
PRORY

HSPH1
CDRT1
CYP39A1
TDO2
BYSL
TWNK
CDC6
SPON2
RPS6KL1
GAPLINC
LOC101928525
LOC100996720
RFPL4B
ZNF692
TNFSF4
CCDC63
ETV5
TRAF5
NELFCD
MIR3142HG
LINC00880
KCNK16
DCAF4L1
KIAA0895
TICRR
LINC01252
FZD3
TERB1
CASC11
PLEKHB1
DLX2
DNAH10
EFNA3
CCNE1
FBXL16
MCM2
CCL3L3
DYX1C1
SCART1
NAA10
FAM19A5
HELLS
EXO1
SHMT2
KIF20A
RIIAD1
METTL21EP
INE1
ZSWIM4
EXOSC5
ZNF705A

FCHO1
BAMBI
LINC00525
LOC107986453
LOC729652
DAPL1
PLXNA1
LINC00106
ART5
ZMYND10
LOC101928295
PHKA1
TMEM206
CDCA4
CALML6
FANCA
MIR6847
AEN
TNFSF15
CAPN14
SLC25A31
LOC101928100
AKNAD1
FAM57A
RPL13AP5
ANXA3
LYPD6B
PODXL2
SLC22A2
AP1S3
PHLDA2
LINC00323
ANKRD61
SNORD91A
MIR5587
CACNA1D
C3orf67
IGFN1
PRC1-AS1
LSM7
CAD
LINC01258
PPP2R3B
B9D1
PLCB4
CEP55
MIR589
RHBDF2
UMODL1
TRPV5
LOC400794

WDR24
HIGD2B
LRRC73
TTLL10-AS1
LOC100131315
GPR84
TMEM97
CCDC175
TFAP4
ADGRF2
C2
ZP2
PDCD2L
LINC01759
ANP32AP1
SLC9A7
IRAK2
KCNN4
FAM27E3
GRAMD1A
PRSS53
C11orf84
LINC00630
MIR6814
PIF1
EPHA1-AS1
B3GNTL1
CD55
PCSK4
GCKR
NXT1
RNVU1-3
1-Dec
KRT81
CCNF
KLHL32
ATAD2
MPP3
NME1
PPFIA4
RNASEH2A
BTBD19
FOXP3
RBM12B-AS1
NUF2
NOTCH3
POC1B-AS1
IL17A
MIR4435-2HG
CDCA7
SNORD83A

SCLY
THY1
CBX4
URAHP
RBPMS-AS1
SPRED3
LOC730668
MIR1914
WNT5A
LINC00954
LOC100506606
DIAPH3
SRD5A2
RNASE7
HJURP
FGF17
SYT8
TNFSF11
ZNF280C
SRM
GOLGA8B
MORC4
LOC102723373
FASN
TFF1
BDNF
SOX4
NTF4
TAF1L
CHSY3
TSPOAP1-AS1
FRMD5
NOD2
PUS1
ITGA2
CPLX1
OLFML2B
UBE2Q1-AS1
AMBP
GALK1
PPAT
LRP1-AS
WDR4
SLC12A2
LINC00239
PLEKHS1
UBOX5-AS1
CFAP74
ACMSD
TMEM158
SPEF1

NDP
PLK1
MIR641
PDIA2
LOC107986266
LINC01968
MYH7B
IFI6
LOC102723322
LHB
CNGB3
GNRH2
MIR1249
KIF12
ZNF121
MIR499A
H3F3C
RNFT2
FOXD4
C4orf48
PUS7
MIR4763
ENGASE
GPT2
SLC5A2
LINC00885
RNF151
MYT1
USH2A
MIR6819
HOXC-AS3
ATP2A1-AS1
LOC105369205
CTU1
ANKRD18A
ARL9
TMEM191A
RGS20
ACTL7B
CLIC3
IFITM3
CNIH3
NPM3
UPK3A
PHGDH
LOC101927189
GNB1L
LOC101059948
OVGP1
MHENCR
PMFBP1

LINC01558
PRRX1
LOC102724434
AGRP
MIR4761
LOC105376557
RCOR2
DLEU7
CATIP
SMG1P6
SPARC
CLCN4
GTF3A
TTC9
PRMT1
TTYH3
NUP62CL
COL9A1
MRGBP
GUCY1B2
C1orf234
MRPS31P5
CCDC114
PRAME
CDT1
CFAP73
FBXW12
MIR25
TREM2
SYT12
EME1
RTP3
PLEKHA8P1
SLC34A1
LRRC74A
OTOG
DNAH14
CDC45
TUBB3
PHACTR3
ZNF239
FCAR
LOC107985075
MCM10
LINC01914
LOC105373191
MEGF6
TBX18
SLC6A14
MIR6772
SPNS2

DPF1
ELFN1
MPP4
TOMM34
FGF18
TIGD4
CCND1
REM2
KIF14
SYT7
MIR6821
KCNA7
CENPI
ODF3L2
JMJD7
MTBP
PPIAL4A
AREG
CFAP46
KCP
LIME1
VCAN
COL6A6
TOP1MT
RANBP17
MFSD12
PPM1N
SERPINF2
KCNK15
RHEBL1
TMEM160
EWSAT1
MIR148A
TPX2
MIR33B
ACTL10
LINC01910
BECN2
KIAA1210
SNORD36C
RTN4R
SNORD100
AKR1E2
LGALS7
SLC2A1
MIR6835
MST1
C16orf59
PLA2G4E
SCML1
C8orf37-AS1

LINC01132
PMEPA1
CA5A
DGCR5
BCAR4
LOC101929626
CCDC85B
BCYRN1
ATP6V1B1
MIR6510
TRPM2
LOC101929536
LRRC2-AS1
LINC01270
SP6
NRCAM
UBE2S
LOC101929634
S100A11
FOXA2
JARID2-AS1
COL5A1
LOC101927394
RFC3
JPH1
HOXC9
FZD9
LOC101927476
SYNE4
OOEP
CD3EAP
MED4-AS1
CARMIL3
IFIT1B
DUSP14
LOC440311
LINC01460
EDNRA
CBX8
SNHG1
POLR3G
NSUN5P1
LINC01315
LOC105370623
KIF18B
AGBL4
ANKRD36B
ALPK3
C9orf116
MYLPF
IDI2

CFAP45
TERT
PTPRU
FADS2
XIRP1
EVPL
VTRNA1-3
CATSPER4
LOC100506085
LZTS1
MIR1182
FBXL13
TRNP1
RPS21
KCNJ14
NPBWR1
COL1A2
ELFN2
TEX41
MIR4768
RAD54B
PYCRL
LOC400684
MPC1L
TTC26
DGAT2
CSNK1A1L
RUBCNL
SNORA5C
PMCH
MYBPH
DSCC1
RHBDL1
PAFAH1B3
SNORD63
LINC02036
TROAP
WDR90
PDPN
MIR6840
APOC4
AKAP14
ECT2
RPL22L1
NT5DC2
GRM2
CPXM1
DCDC2
SMOC1
LRP4-AS1
CHAC1

PODNL1
FREM1
LVCAT8
LINC00184
CLEC6A
COL22A1
HAPLN3
CPNE9
DCDC5
ZBTB12
L3MBTL1
FAM222A
HCN1
CCDC192
ASIC1
IFITM1
ACPT
PTPRO
IER5L
OTUB2
SLC6A3
PLAC1
CYP2F1
LOC105372240
CSMD2
ERCC6L
ODAM
HECW1
LOC105369332
GALNT6
CCDC150
TUSC8
GRB7
FOXS1
LINC-PINT
PRL
LIF
SNAI1
FAM155B
DDIAS
OPRD1
TAAR3P
NMB
MYO3B
KRT18P55
STX16-NPEPL1
ANKRD31
SLC16A8
RBP1
TMEM52B
MIR3183

APOL5
KLHL31
OSBPL3
KCTD16
C11orf87
HLA-L
ADAMTSL2
EYA1
ESPNP
PRR5-ARHGAP8
CCDC113
LRRC15
EGR4
GINS1
CHPF
LINC02097
TMEM255B
AQP6
MGC20647
TRIM7
HECW2
PYCR1
MIR3198-1
TMEM147-AS1
SERPINE1
LOC101927604
ZNF670-ZNF695
KLHL17
UNC5CL
PALM2-AKAP2
HAMP
PITX1
MC1R
SBSN
PRODH2
LINC01007
ECEL1
SNORD124
ALDH4A1
DNAH12
STX1A
ZFAS1
BRCA2
FSCN1
PMAIP1
SLC5A6
ORC6
ERVMER34-1
SKA3
C2CD4B
LINC02126

ZNRF3
LOC441178
PCDHB9
SLC22A10
PPP1R27
TCF7
CXCL2
LOC101928075
PRMT5-AS1
LOC100505478
LINC01747
LINC01572
E2F7
TBC1D29
PAX2
ENC1
OBP2A
SNORD93
LDLRAD3
LYPD3
LOXL2
LSAMP-AS1
OLAH
NPIPB5
COL5A2
LGR6
SNHG15
CASC8
ZP3
LRP2
SNORD99
LINC00311
MIR324
WDR62
E2F1
MIR7112
C19orf48
RHPN1-AS1
IL1RN
SBK1
PBX4
F12
HTRA4
SLX1A
LINC01426
MYC
PRR19
MNX1-AS1
CPNE1
GZMB
LOC105371795

TSACC
SLC22A3
UCN
MYBPC3
CEP72
TGIF2
SMARCA5-AS1
LINC02182
MIR647
SRCIN1
KRT40
LOC105371184
TWIST1
CDH11
FBXO2
RPLP0P2
CASC15
KCTD19
LOC100506071
SLC39A10
SNORD1B
LDLRAD1
LINC01649
S100A5
CHTF18
LCN1
ALMS1-IT1
LOC105376468
PRELID3A
SLC2A12
FBXO41
RNF224
SOX9
BRDT
NECTIN4
RSPH14
JAG2
LOC101929516
ZNF695
NR5A1
SYNPR
HSF4
MIR6719
SLC24A2
BMP4
KIAA2012
SLC16A1-AS1
IQGAP3
COL8A1
TRPV4
BFSP1

LINC01091
PYDC1
PTPRG-AS1
MIR548AA1
THEG
NPIPA3
F2RL2
SLCO5A1
XKR9
MIR6761
TNNT2
KRT42P
CDH7
FAM83C-AS1
UBE2C
MELTF
LINC01186
SNORD60
AUNIP
VTCN1
ZNF469
MPP6
ANLN
COL27A1
LOC105369340
USP41
LAMA5-AS1
UBE2NL
KCNJ11
DNAAF3
IFITM2
LINC01271
BEAN1
DMKN
PKMYT1
HOMER1
BIVM-ERCC5
ANKRD18B
PROX1
PLS3-AS1
MELTF-AS1
LOC101927040
DENND5B-AS1
CCDC148
SCD
RAB36
OR2AE1
LINC00346
KCNJ15
ZNF114
SAPCD2

NOXO1
C1QL4
GBP7
MIR1276
CELP
XRCC2
RTEL1-TNFRSF6B
MIR6891
SLC27A5
SAPCD1
SIGLEC15
SNORD42B
FOCAD-AS1
PAQR4
GTF3C2-AS1
LOC541473
SNORD51
UPK1A-AS1
MROH6
ANKRD36
RTP2
TIMP1
LAIR2
PCAT1
SNORD101
FANCB
MIR6859-1
CCL24
CREG2
HHLA1
HES6
GCNT7
PPP2R2C
TTLL8
SIX3
SNORA55
GSC
RPEL1
LMTK3
CYP27B1
LBX2
LOC101927196
SHROOM4
TYRO3
FXYD5
GRM4
SLC25A2
PALD1
MIR3174
MSH5
NEK5

CFAP157
SPINK7
UROC1
GAS2
RGS16
WFDC13
RASL10B
CADPS
HCAR2
VGF
TNFRSF11B
SLC38A5
LINC01121
PIN1P1
LOC101928797
ACOD1
SULF1
SNORA3B
PCDHGA1
UHRF1
CDC25B
PLEKHG4
COL6A4P1
GRM8
ARID3A
PRR25
MYOM3
DUSP15
HSP90B2P
VSIG8
GRHL1
RECQL4
RETN
LZTS3
RELL2
LINC00896
MIR3153
MYBL2
RIPPLY1
TRIP13
LINC01087
HES4
CSNK1G2-AS1
LOC100130705
LINC01449
CXCL6
MIR6812
LOC101929759
SNORD19
SHH
ERICH2

ATAD3C
PABPC3
STC1
DLGAP4-AS1
ULBP3
INHBB
MIR5580
ITGA11
EPHB3
MSH4
TMEM88B
COL12A1
P4HA3
SNORD104
CARD14
CALHM3
IL20
NT5DC4
SNHG17
DOK7
EN1
SBSPON
GABRR1
SPOCD1
CAPN12
LINC01484
DBNDD1
UNC5A
CFAP161
CXCL11
HOXC6
SNORD69
LINC01840
KRTAP5-1
SMTNL2
PTK7
FBXO47
DACH1
SRMS
PCDHB8
LINC01623
GTF2IRD1
PTRH1
SPHK1
MAP7D2
INTS9-AS1
ACBD7
DIO2
FKBP10
POU3F2
LINC01254

HIST3H2A
LRRC43
RTN4RL2
ASB9
TAS2R1
CCR8
LINC01972
TP73
RAD51AP2
HNRNPCL1
CXCL1
SLC6A20
SNORA65
CLDN9
MMP10
R3HDML
LINC01764
ZAN
HPN-AS1
LY6E
LEF1
DLL3
MIR6797
PANO1
SNORD62A
LOC105369848
FGGY
PTP4A3
KCNV2
CYP4F8
OSM
ZC3HAV1L
WNT11
WFDC10B
ADRA2C
CNDP1
LINC01305
CYSRT1
C10orf55
SPATA3-AS1
NODAL
LOC101928565
AXIN2
FXYD4
ATP6V1C2
ABALON
LINC01494
KCNH4
SAMMSON
AGT
ATP5EP2

SLC17A9
PPM1H
TLX1NB
HILPDA
SNORD121B
PGLYRP4
SETSIP
DKK1
CER1
RPA4
TEAD4
KIF25
PDE10A
ANP32D
FRMD8P1
C8orf34-AS1
SMCR2
FAM156B
ASPHD1
PANX2
SNTB1
CGB7
MIR34AHG
KREMEN2
DLX4
IFNL2
IZUMO1
LINC01267
LINC01749
TSPEAR-AS1
MYH6
HIST3H2BB
KIF4B
ABCC2
MIR4713HG
DSG1
TTC16
GPR143
S100A3
AQP9
TNFRSF12A
MACC1
MTHFD1L
LOC105372343
ATP11A
LOC105370400
SNORD15A
LOC105375800
IL1A
C20orf144
GREB1L

LOC221122
RHBG
FANCD2OS
PLCG1-AS1
FOSL1
PRSS1
LINCR-0001
SNORD14E
DSG4
ANKRD13B
ORM2
PRSS30P
PHLDA1
MIR6768
SMCO2
LINC01124
FOXP4-AS1
SMOX
LRRC9
LINC01320
MIR675
FAM131C
MIR181A2HG
PADI1
LINC00485
WBP11P1
GNAT1
S100P
RNF43
RHCG
GRP
PRLHR
AFP
RAPGEF4-AS1
TUBA4B
ARNTL2
KIAA1549
KRT78
CCDC166
NEBL
TBX4
C19orf45
LOC105375401
FNDC11
NMUR2
ITIH6
SNORA5A
WNT8B
GPRC6A
VPS9D1-AS1
KCNT1

FER1L4
SKOR2
ANKRD33
LOC101927911
STRIP2
NFE2L3
LOC101929777
MYH4
ADAMTS6
MIR1289-1
LINC02043
ARSH
GPR3
ZNF460
LEF1-AS1
MSLN
SSTR5-AS1
EDDM3A
UPK1A
TBX15
CELSR1
FAM227A
TMPRSS3
KAAG1
CXCL3
LINC01206
EXTL3-AS1
RASSF10
SLC38A3
CLLU1OS
LINC01618
TAS2R42
LOC389602
TMEM132A
CILP2
KISS1
SAA4
MIR98
BGN
LINC00894
KIAA1549L
ADAMTS2
FGF8
TYR
ROBO2
TMEM105
LCN12
GLS2
DBET
LINC01356
SLC12A5-AS1

NMU
LINC01844
GSDMA
HCAR3
THBS2
MIR4793
CHRNA6
DLEU7-AS1
PITX3
MIR378D2
LOC101928782
LINC00862
LOC105376382
GFY
WDR97
SULT1C2
RPSAP52
CBARP
SNORD117
MIR3197
ASGR1
LRRC8E
MIR4665
MIR23C
LINC00618
GOLGA7B
PALM3
GCM1
WBSCR28
LOC101929460
SULT1C2P1
B4GALNT4
SNORA71B
DSCAM-AS1
LINC01146
RNVU1-1
SLC28A3
GSDMC
IL23A
GABRP
IRX2
FRMD7
FAM83H-AS1
MIR3065
LINC01269
VSNL1
POU4F1
ARHGAP40
LMO7-AS1
SH2D5
ERVV-1

MLXIPL
MEGF11
POTEF
MIR6750
IL36RN
IL17C
ERP27
MFAP2
FRGCA
FIBCD1
CYMP
CDIPT-AS1
LINC01551
PCSK6-AS1
GNG4
LINC00624
MS4A15
ZNF32-AS3
ONECUT2
LINC01942
LINC01338
CNPY1
LINC00922
GDPD5
MAGEA10
C1orf105
INHA
CHRNG
TM4SF19
PSAT1
KCNH8
LOC101927780
SPAG17
NDUFA4L2
LINC01555
HCRT
DUSP13
CCL26
MIR6851
C2orf78
MIR3165
SLC7A11
SLC11A1
SPACA3
MIR1204
SNORD46
SNORD88A
SBF1P1
NXF4
LRRN4
RDH16

CASP14
RFX8
MYADML2
SPTBN5
LOC101928336
XKRX
KRT7
LMLN-AS1
SNORA26
FUT1
LINC01929
C1QTNF8
CCDC187
MIR452
KCNK9
SIX2
PRDM12
WISP1
TH
MAS1
SIX4
COL1A1
TMEM74B
MATN3
DNASE1L2
MIR17HG
LINC02154
LHX3
FJX1
SPATA21
CYP26A1
PDCL2
CYP2D7
PGK2
EDAR
BRSK2
PCSK9
PERM1
TAS2R38
SNORD105
C17orf99
LOC101927657
MIR7111
MEX3A
LOC105375744
MAFG-AS1
LOC100507194
LINC01833
DKFZP434K028
HOXB8
CACNG4

C5orf38
LINC00552
DNAH5
WDR66
OTP
LRRC36
FAM25A
AJUBA
GABRD
SLC6A6
PTGES2-AS1
MIR6810
RBAKDN
ESRG
CYP4Z1
MIR6877
CITED1
DSG1-AS1
KRTAP10-4
SERPINA10
MIR424
MIR6895
GJC3
MIR6728
PLPP4
IQCF1
KCNE1B
ISM2
LGR5
CLDN16
CD300LD
OXTR
C20orf141
LINC01730
MYEOV
TDGF1
FRMPD2
GRPR
EGFLAM-AS4
CHI3L1
LOC100287413
PPEF1
HTR1D
HOGA1
NXF3
LOC101927787
REG4
LOC344967
RAET1K
CLDN14
LOC100505685

C9orf50
PLPPR1
LINC00658
LOC105369758
LOC103191607
TMPRSS5
LRP8
MIR6783
MIR4479
RNVU1-4
APCDD1
SNORD72
SPDYC
CKMT2
LINC01979
TMEM8C
WASIR2
SOHLH1
CSF2
GDF15
MIR3189
PLK5
ITGBL1
KLRG2
TEX19
EGOT
KIF26B
CCNO
KCNA10
FOXD1
PADI3
LOC105375451
PABPC1L
SH3PXD2A-AS1
LINC01792
H2BFM
MIR711
MIR146B
PAX9
LINC02011
SLC7A5
ZPBP
KLK12
ACAN
LINC00939
LOC107987205
DLX3
SNORA13
C8orf49
OR1F2P
STEAP2-AS1

HPN
PRSS2
CYP4X1
TFR2
MIR8078
NPFFR1
CHRNB3
AZGP1
ALOXE3
OR7C1
NACA2
SPTBN2
LINC01722
MSX1
LOC101927795
DCSTAMP
SRGAP3-AS2
UPK2
LOC101929532
CST9
TBX20
LHX1
MCEMP1
MAT1A
MIR5684
RAX
TNS4
LOC101929227
EVX1
DNAH2
TSPEAR
LOC440742
BMP7
LOC101928012
IGFBP1
PSAPL1
LOC255187
RHPN1
WDR72
MIR29B1
MIR4648
EGFL6
LINC02081
NEB
C2-AS1
RNF183
CDKN2A
APLN
LINC01657
MIR328
TMEM249

ANXA10
SP9
NOX4
AKR1C8P
PRSS54
AZGP1P1
STC2
SRPX2
ANOS1
KRTAP5-3
FAP
HOXC12
HOXC-AS2
TRIM54
MEPE
ANKRD30A
HCAR1
DLX6
BTBD16
DUXAP8
WNT7A
LINC01978
LINC01549
UCP1
CPA4
RIPPLY3
PLEKHN1
DIO2-AS1
TMPRSS13
WIF1
MIR2052HG
EPOP
PRDM13
SHISA2
CXCL8
FGFRL1
SEMG1
FZD10
ALOX12P2
MDFI
TGFBI
NAT16
SERPIND1
UCA1
CXXC1P1
EREG
S100A2
LINC01276
MIR6858
APELA
MIR3651

LOC105378470
LY6G6C
METTL11B
FGF19
SFRP4
CHRND
LINC00545
USP17L7
PNPLA3
TREML3P
CSTL1
VAC14-AS1
ZNF556
RNF182
MIR6801
LINC01391
TMEM40
GNGT1
LUCAT1
PRR7
HES7
FOLR1
KRTAP10-2
SERPINA7
LINC01630
ALKAL1
MIR6087
PGGHG
WFDC11
SLCO4A1
LINC01676
FAM222A-AS1
WT1-AS
CGREF1
KLK11
CPXCR1
DDC-AS1
LINC02188
LINC01433
BLACE
LOC101926941
CCDC70
SLURP1
LOC284798
ZNF750
KRTAP12-1
MIR4737
C2orf70
LOC101929210
IRX3
DIRC1

ASCL2
MEIKIN
HAGHL
CBX2
GAST
SIX1
LINC01615
TNFSF9
MAFA-AS1
BAAT
C9orf57
C19orf81
SLAMF9
ERFE
LINC01721
EVA1A
HS3ST4
CLEC5A
IGFL3
CLDN10
SH3TC2
TLX1
TMEM30CP
PLA2G3
LINC01048
SERPINB5
TFAP2A
LINC01694
TMPRSS12
IZUMO2
RAET1L
SP5
ULBP1
GRIN2B
LINC01246
MRPL23-AS1
DUSP4
GJA3
LOC101928227
FOXL2NB
GIF
LINC02029
LOC105379109
KLK5
CCDC78
ADAMTS12
SNORD12B
ASCL5
ATG9B
LINC01711
HS6ST2

SLCO4A1-AS1
RPL29P2
SLC4A11
SNORD12C
PRKCG
LINC01583
FGB
LOC100996351
MIR935
SPATA12
KIAA1257
LINC01819
MIR4641
PSORS1C2
CORIN
PRSS22
FAM159B
IGF2BP3
GRK1
TRIB3
GRHL3
WISP3
OLR1
POU6F2
LOC388282
MIR4284
CTHRC1
OR1F1
GUCA1C
IGFBP7-AS1
KRT85
GATA4
IFNL3
MIR3188
ALDH3B2
IGFL2
DUSP9
MIR4321
LINC01633
WFDC10A
DSG3
LINC02120
RGR
TMEM211
LOC101926964
LINC00524
SMILR
LINC01827
C1orf158
CLPSL1
MCIDAS

TPRXL
MMP20
LINC01429
LOC101927120
LOC100129940
KRT6C
SMKR1
LEMD1-AS1
MYO16-AS1
LHX5
PKP1
MUC5AC
DLGAP1-AS5
MAPK15
LPO
LOC101927136
KRT7-AS
RNF225
GJB4
SERPINB2
LINC01060
GCM2
SNORD70
LINC01756
KRT6A
RPTN
C2CD4A
NPC1L1
HOTAIR
LYZL4
BHMG1
IFNE
NHEG1
LOC101929705
FGF21
SNORA70B
NPSR1
CAMKV
NANOS3
CXCL5
CPN1
FLJ16779
SPP1
PGLYRP3
PGC
KLHL35
CHST4
SPZ1
SNORD48
NKD2
HOXC11

NKD1
CRAT40
MIR4742
KRT6B
ACSL6
ABCA9-AS1
SLC22A11
LINC00698
LINC00592
LINC01977
ADAM12
ADAM18
VWA2
CALCA
TG
SLC22A12
TRIM29
SNORA71C
LOC101929128
TESC
STK31
SOX1
SSTR5
CYP2W1
POU5F1B
LINC00941
FOXG1
MIR4449
DMRT3
DMRTA2
GJB5
ULBP2
TCF24
EPHA8
KRT17
UNC93A
LINC01613
DSC3
IRX5
IL11
SLC13A3
VGLL1
SIM2
C10orf62
KRT74
TNNI3
FEZF2
TNNT1
CCAT2
TDGF1P3
LINC01704

CST5
PRR36
CTB-113P19.1
LOC101929504
ST8SIA6-AS1
MSX2
CLDN1
MC2R
EPHX4
DDN
LINC01729
DCAF8L2
C6orf223
RXFP3
LOC101927480
LINC01594
KRTAP10-3
LARGE2
EVX1-AS
MMP11
KRTAP3-2
CLDN2
PAEP
ADAM2
MAP3K20-AS1
OFCC1
LINC00967
MIR3661
LINC00348
KRT16
KRT83
TACSTD2
MIR566
PDX1
PTTG3P
SPRR3
AKAP4
SALL4
EIF3IP1
GRIN2D
UCN2
LOC401585
FGF3
TRIM71
SLC35D3
BBOX1-AS1
SNORD41
TCAM1P
MAGEB17
CXCL17
ELFN1-AS1

MC3R
C2orf61
CLPSL2
LY6G6D
CEMIP
IGFL4
ERVV-2
DMBX1
ETV4
OTOP1
GAD1
ZNF280A
LOC105377390
CNTD2
WT1
RP1
TRIM72
USP26
TBX5
LINC02027
PRSS33
NEUROG2
SNHG25
LINC01511
LINC00534
KLK10
PIWIL1
INHBA
LOC102724623
SPRR2E
ARNTL2-AS1
CA9
ESM1
KRT38
ZIC2
AKR1C4
SERPINA4
FOXI3
BOD1L2
AMELX
LINC02119
WNT7B
SNORD38A
CLDN10-AS1
MIR613
LINC01996
FAR2P1
LOC100507377
OTX1
LEMD1
FIRRE

LINC01608
WNT2
EN2
KLC3
NXPH1
SPRR1B
C3P1
NXPH4
CPNE7
MIR135B
PCAT2
LOC101927318
CDH3
GBX2
ONECUT3
FAM9A
SPRR2A
CRAT37
LINC00858
LOC284930
LINC01101
MIR4474
STRA6
LINC01411
LY6G6F
OBP2B
MAGEA11
IFITM5
KRT35
SLCO1B3
C14orf105
KRT32
SPRR2D
FOXQ1
LINC01169
AFAP1-AS1
C5orf46
COL11A1
IBSP
BMS1P22
MMP7
COMP
NPSR1-AS1
LOC101929448
KRT80
CST2
SPRR1A
LINC01655
CEL
LOC102724908
LINC01913

KRT23
SNORD30
LINC02187
SFTA2
PTF1A
EPYC
DUSP27
ELF5
LINC01602
SPERT
LINC01050
SOX14
LINC01234
COL10A1
ZIC5
LINC01593
C6orf15
CST4
LOC101927884
CST1
KLK7
KLK8
FEZF1
KLK6
GOLGA6L2
FEZF1-AS1
LOC100190940
RNA5S9
NOTUM

Table 5S

node1	node2	node1_stri	node2_stri	node1_exte	node2_exte	neighborho	gene_fusio	phylogenet	homology	coexpressi	experiment	database_a	automated_	combined_score
CDC42	WAS	4446990	4444767	9606.ENSPC	9606.ENSPC	0	0	0	0	0.063	0.974	0.9	0.97	0.999
HSPA8	HSP90AA1	4449616	4440643	9606.ENSPC	9606.ENSPC	0	0	0	0	0.771	0.73	0.9	0.929	0.999
DHFR	TYMS	4447922	4439011	9606.ENSPC	9606.ENSPC	0.219	0.317	0.437	0	0.479	0.122	0.9	0.96	0.999
CCNA2	CDK2	4436109	4435732	9606.ENSPC	9606.ENSPC	0	0.006	0	0	0.403	0.998	0.9	0.973	0.999
CCNA2	CDK6	4436109	4435642	9606.ENSPC	9606.ENSPC	0	0.003	0	0	0.163	0.994	0.72	0.769	0.999
CDC42	TNK2	4446990	4445619	9606.ENSPC	9606.ENSPC	0	0	0	0	0	0.974	0.9	0.641	0.998
HSPA8	HSP90AB1	4449616	4443770	9606.ENSPC	9606.ENSPC	0	0	0	0	0.641	0.745	0.9	0.815	0.998
RXRA	PPARG	4449061	4436745	9606.ENSPC	9606.ENSPC	0	0	0	0.694	0.061	0.961	0.9	0.821	0.997
TYMS	TK1	4439011	4437779	9606.ENSPC	9606.ENSPC	0.169	0	0	0	0.787	0	0.9	0.856	0.997
HSP90AB1	HSP90AA1	4443770	4440643	9606.ENSPC	9606.ENSPC	0	0	0.449	0.984	0.78	0.883	0.9	0.717	0.997
GART	MTHFD1	4445605	4432975	9606.ENSPC	9606.ENSPC	0.37	0	0	0	0.695	0	0.9	0.82	0.996
AKR1C1	AKR1C3	4445467	4445414	9606.ENSPC	9606.ENSPC	0	0	0.449	0.983	0.214	0.943	0.9	0.836	0.995
RXRA	THRB	4449061	4446585	9606.ENSPC	9606.ENSPC	0	0	0	0.691	0	0.974	0.8	0.782	0.995
KIF11	AURKA	4434801	4432988	9606.ENSPC	9606.ENSPC	0	0	0	0	0.94	0.482	0	0.831	0.994
GP1BA	F2	4440098	4438421	9606.ENSPC	9606.ENSPC	0	0	0	0	0	0.909	0.9	0.481	0.994
TYMS	DTYMK	4439011	4438077	9606.ENSPC	9606.ENSPC	0.182	0	0	0	0.467	0.05	0.9	0.839	0.992
NR1H4	RXRA	4450225	4449061	9606.ENSPC	9606.ENSPC	0	0	0	0.668	0.062	0.904	0.9	0.745	0.992
APRT	ADK	4445041	4436683	9606.ENSPC	9606.ENSPC	0.043	0	0	0	0.088	0.159	0.9	0.909	0.992
HSP90AA1	NOS3	4440643	4437409	9606.ENSPC	9606.ENSPC	0	0	0	0	0.06	0.379	0.9	0.873	0.991
DTYMK	TK1	4438077	4437779	9606.ENSPC	9606.ENSPC	0.05	0	0	0	0.569	0	0.9	0.817	0.991
AKR1C1	AKR1C2	4445467	4445450	9606.ENSPC	9606.ENSPC	0	0	0.449	0.986	0.405	0.86	0.9	0.892	0.991
CCNA2	KIF11	4436109	4434801	9606.ENSPC	9606.ENSPC	0	0	0	0	0.977	0.15	0	0.478	0.989
PNP	ADK	4442485	4436683	9606.ENSPC	9606.ENSPC	0.167	0	0	0	0.061	0	0.9	0.88	0.989
ESR1	IGF1R	4448358	4435797	9606.ENSPC	9606.ENSPC	0	0	0	0	0	0.407	0.9	0.835	0.989
HSP90AA1	EGFR	4440643	4436186	9606.ENSPC	9606.ENSPC	0	0	0	0	0	0.487	0.9	0.817	0.989
HSP90AA1	PPP5C	4440643	4432591	9606.ENSPC	9606.ENSPC	0	0	0	0	0.106	0.581	0.9	0.718	0.988
AR	HSP90AA1	4444436	4440643	9606.ENSPC	9606.ENSPC	0	0	0	0	0	0.474	0.9	0.803	0.988
DHFR	GART	4447922	4445605	9606.ENSPC	9606.ENSPC	0.346	0	0	0	0.116	0	0.9	0.827	0.988
RXRA	RARG	4449061	4447554	9606.ENSPC	9606.ENSPC	0	0	0	0.79	0.084	0.853	0.9	0.759	0.987
CCNA2	AURKA	4436109	4432988	9606.ENSPC	9606.ENSPC	0	0	0	0	0.964	0.177	0	0.582	0.986
HSP90AA1	PGR	4440643	4439740	9606.ENSPC	9606.ENSPC	0	0	0	0	0	0.523	0.9	0.72	0.985
AKR1C2	AKR1C3	4445450	4445414	9606.ENSPC	9606.ENSPC	0	0	0.449	0.983	0.214	0.912	0.8	0.836	0.985
ESR1	NOS3	4448358	4437409	9606.ENSPC	9606.ENSPC	0	0	0	0	0	0.472	0.9	0.749	0.985
APRT	PNP	4445041	4442485	9606.ENSPC	9606.ENSPC	0.219	0	0	0	0.084	0	0.9	0.805	0.984
TYMS	MTHFD1	4439011	4432975	9606.ENSPC	9606.ENSPC	0.208	0	0	0	0.159	0	0.9	0.79	0.984
HSP90AA1	NR3C1	4440643	4433539	9606.ENSPC	9606.ENSPC	0	0	0	0	0	0.474	0.9	0.71	0.983
AKT2	HSP90AA1	4445979	4440643	9606.ENSPC	9606.ENSPC	0	0	0	0	0.061	0.713	0.9	0.428	0.982
ELANE	CTSG	4450799	4432948	9606.ENSPC	9606.ENSPC	0	0	0.437	0.821	0.784	0	0.9	0.92	0.982
CCNA2	CDK7	4436109	4434485	9606.ENSPC	9606.ENSPC	0	0.003	0	0	0.081	0.451	0.9	0.669	0.981
CDA	TK1	4444499	4437779	9606.ENSPC	9606.ENSPC	0.292	0	0	0	0.061	0	0.9	0.742	0.98
DHFR	MTHFD1	4447922	4432975	9606.ENSPC	9606.ENSPC	0.146	0	0	0	0.17	0	0.9	0.739	0.979
ESR1	HSP90AA1	4448358	4440643	9606.ENSPC	9606.ENSPC	0	0	0	0	0	0.474	0.9	0.633	0.979
HDAC8	KAT2B	4444214	4435311	9606.ENSPC	9606.ENSPC	0	0	0	0	0.094	0.266	0.9	0.708	0.978
AR	HSP90AB1	4444436	4443770	9606.ENSPC	9606.ENSPC	0	0	0	0	0	0.474	0.9	0.609	0.977
HSP90AB1	PPP5C	4443770	4432591	9606.ENSPC	9606.ENSPC	0	0	0	0	0.103	0.581	0.9	0.456	0.976
CDA	PNP	4444499	4442485	9606.ENSPC	9606.ENSPC	0.219	0	0.215	0	0.077	0	0.9	0.642	0.976
HCK	EGFR	4450141	4436186	9606.ENSPC	9606.ENSPC	0	0	0	0.679	0	0.701	0.9	0.722	0.975
CDK2	CDK6	4435732	4435642	9606.ENSPC	9606.ENSPC	0	0	0.439	0.927	0.108	0.711	0.9	0.908	0.974
ADH1C	ADH1B	4449347	4438237	9606.ENSPC	9606.ENSPC	0	0	0.449	0.986	0.179	0.692	0.9	0.921	0.973
CDC42	EGFR	4446990	4436186	9606.ENSPC	9606.ENSPC	0	0	0	0	0	0.126	0.9	0.714	0.972
ERBB4	ADAM17	4441238	4438549	9606.ENSPC	9606.ENSPC	0	0	0	0	0	0.379	0.9	0.57	0.971
DHFR	CBR1	4447922	4436857	9606.ENSPC	9606.ENSPC	0	0	0	0	0.062	0	0.9	0.725	0.971
CDK2	MDM2	4435732	4434604	9606.ENSPC	9606.ENSPC	0	0	0	0	0.05	0	0.9	0.697	0.968
HSP90AB1	NR3C1	4443770	4433539	9606.ENSPC	9606.ENSPC	0	0	0	0	0	0.403	0.9	0.509	0.968
CHEK1	MDM2	4447561	4434604	9606.ENSPC	9606.ENSPC	0	0	0	0	0	0	0.9	0.698	0.968
AR	CDK6	4444436	4435642	9606.ENSPC	9606.ENSPC	0	0	0	0	0	0.524	0.9	0.399	0.968
ELANE	MMP8	4450799	4433667	9606.ENSPC	9606.ENSPC	0	0	0	0	0.175	0	0.9	0.633	0.967
AR	KAT2B	4444436	4435311	9606.ENSPC	9606.ENSPC	0	0	0	0	0	0.478	0.9	0.43	0.967
NR3C2	HSP90AA1	4442077	4440643	9606.ENSPC	9606.ENSPC	0	0	0	0	0	0.474	0.9	0.425	0.967
AKR1C3	CBR1	4445414	4436857	9606.ENSPC	9606.ENSPC	0	0	0	0	0.063	0	0.9	0.668	0.966
ESR1	HSP90AB1	4448358	4443770	9606.ENSPC	9606.ENSPC	0	0	0	0	0	0.474	0.9	0.421	0.966
MMP9	MMP8	4443919	4433667	9606.ENSPC	9606.ENSPC	0	0	0	0.738	0.546	0	0.9	0.915	0.964
CRABP2	RARG	4451723	4447554	9606.ENSPC	9606.ENSPC	0	0	0	0	0.098	0.064	0.9	0.63	0.964
AKT2	PIK3CG	4445979	4442215	9606.ENSPC	9606.ENSPC	0	0	0	0	0.063	0.174	0.9	0.594	0.964
ESR1	PGR	4448358	4439740	9606.ENSPC	9606.ENSPC	0	0	0	0.679	0.102	0.472	0.9	0.964	0.964
ESR1	CYP19A1	4448358	4446543	9606.ENSPC	9606.ENSPC	0	0	0	0	0	0.085	0	0.96	0.962
CHEK1	CDK2	4447561	4435732	9606.ENSPC	9606.ENSPC	0	0	0.308	0.719	0.325	0.261	0.9	0.885	0.962
KAT2B	MDM2	4435311	4434604	9606.ENSPC	9606.ENSPC	0	0	0	0	0	0.379	0.9	0.426	0.961
MMP9	MMP1	4443919	4439553	9606.ENSPC	9606.ENSPC	0	0	0	0.75	0.518	0	0.9	0.91	0.961
CCNA2	MDM2	4436109	4434604	9606.ENSPC	9606.ENSPC	0	0	0	0	0.062	0.108	0.9	0.58	0.96
GSTM1	GSTM2	4438694	4433798	9606.ENSPC	9606.ENSPC	0	0	0.447	0.981	0.83	0.379	0.65	0.831	0.96
HSPA8	EGFR	4449616	4436186	9606.ENSPC	9606.ENSPC	0	0	0	0	0	0.393	0.9	0.386	0.959
MMP9	MMP3	4443919	4437610	9606.ENSPC	9606.ENSPC	0	0	0	0.791	0.518	0	0.9	0.911	0.959
CDK6	CDK7	4435642	4434485	9606.ENSPC	9606.ENSPC	0	0	0.393	0.846	0.063	0.504	0.9	0.805	0.958
CTSK	MMP13	4435989	4434767	9606.ENSPC	9606.ENSPC	0	0	0	0	0.062	0	0.9	0.591	0.958
MMP9	CTSG	4443919	4432948	9606.ENSPC	9606.ENSPC	0	0	0	0	0.089	0	0.9	0.569	0.957
CDK2	CDK7	4435732	4434485	9606.ENSPC	9606.ENSPC	0	0	0.408	0.926	0.067	0.539	0.9	0.92	0.957
RXRA	PPARD	4449061	4438644	9606.ENSPC	9606.ENSPC	0	0	0	0.705	0.062	0.467	0.9	0.604	0.955
PCK1	PPARG	4439313	4436745	9606.ENSPC	9606.ENSPC	0	0	0	0	0.062	0.05	0.9	0.564	0.955
VDR	RXRA	4450226	4449061	9606.ENSPC	9606.ENSPC	0	0	0	0.635	0	0.438	0.9	0.677	0.955
GNPDA1	GPI	4449262	4448366	9606.ENSPC	9606.ENSPC	0.111	0	0	0	0.063	0	0.9	0.53	0.955
CDC42	MET	4446990	4439114	9606.ENSPC	9606.ENSPC	0	0	0	0	0.063	0.271	0.9	0.429	0.955
FDPS	FNTA	4441886	4437958	9606.ENSPC	9606.ENSPC	0	0	0	0	0.061	0	0.9	0.552	0.954
MMP3	EGFR	4437610	4436186	9606.ENSPC	9606.ENSPC	0	0	0	0	0.076	0	0.9	0.543	0.954
MMP1	CMA1	4439553	4434128	9606.ENSPC	9606.ENSPC	0	0	0	0	0	0.379	0.9	0.311	0.953
CDC42	MAPK10	4446990	4442217	9606.ENSPC	9606.ENSPC	0	0	0	0	0.063	0.162	0.9	0.476	0.953
MMP1	MMP3	4439553	4437610	9606.ENSPC	9606.ENSPC	0	0	0.298	0.953	0.529	0	0.9	0.921	0.953
CHEK1	CCNA2	4447561	4436109	9606.ENSPC	9606.ENSPC	0	0	0	0	0.853	0.064	0	0.685	0.953
CYP19A1	AKR1C3	4446543	4445414	9606.ENSPC	9606.ENSPC	0	0	0	0	0.061	0	0.9	0.531	0.952
CTSS	LYZ	4443237	4434847	9606.ENSPC	9606.ENSPC	0	0	0	0	0.484	0	0.9	0.156	0.952
PLAU	F2	4444017	4438421	9606.ENSPC	9606.ENSPC	0	0	0.415	0.645	0	0.379	0.9	0.434	0.952

HSP90AB1	NOS3	4443770	4437409	9606.ENSPC	9606.ENSPC	0	0	0	0	0.06	0	0.9	0.525	0.951
AKT2	MDM2	4445979	4434604	9606.ENSPC	9606.ENSPC	0	0	0	0	0	0.05	0.9	0.529	0.951
PLAU	EGFR	4444017	4436186	9606.ENSPC	9606.ENSPC	0	0	0	0	0.088	0	0.9	0.513	0.951
ELANE	RNASE2	4450799	4437950	9606.ENSPC	9606.ENSPC	0	0	0	0	0.335	0	0.9	0.315	0.95
ERBB4	MDM2	4441238	4434604	9606.ENSPC	9606.ENSPC	0	0	0	0	0	0.305	0.9	0.33	0.949
EPHX2	CYP2C8	4449539	4443714	9606.ENSPC	9606.ENSPC	0.056	0	0	0	0.051	0.081	0.9	0.481	0.949
LCN2	MMP8	4444080	4433667	9606.ENSPC	9606.ENSPC	0	0	0	0	0.097	0	0.9	0.473	0.948
ESR1	KAT2B	4448358	4435311	9606.ENSPC	9606.ENSPC	0	0	0	0	0	0.194	0.9	0.398	0.947
ELANE	LCN2	4450799	4444080	9606.ENSPC	9606.ENSPC	0	0	0	0	0.12	0	0.9	0.442	0.946
HSP90AB1	PGR	4443770	4439740	9606.ENSPC	9606.ENSPC	0	0	0	0	0	0.299	0.9	0.296	0.946
HSP90AA1	AURKA	4440643	4432988	9606.ENSPC	9606.ENSPC	0	0	0	0	0.072	0.092	0.9	0.444	0.946
LYZ	CTSG	4434847	4432948	9606.ENSPC	9606.ENSPC	0	0	0	0	0.213	0	0.9	0.378	0.946
KAT2B	NR3C1	4435311	4433539	9606.ENSPC	9606.ENSPC	0	0	0	0	0.083	0.194	0.9	0.349	0.945
PAH	DHFR	4450247	4447922	9606.ENSPC	9606.ENSPC	0	0	0	0	0.062	0	0.9	0.463	0.945
AR	NR3C2	4444436	4442077	9606.ENSPC	9606.ENSPC	0	0	0	0.779	0.061	0.379	0.9	0.67	0.945
AR	NR3C1	4444436	4433539	9606.ENSPC	9606.ENSPC	0	0	0	0.816	0	0.379	0.9	0.806	0.944
HSPA8	NR3C1	4449616	4433539	9606.ENSPC	9606.ENSPC	0	0	0	0	0	0.166	0.9	0.386	0.944
ESR1	EGFR	4448358	4436186	9606.ENSPC	9606.ENSPC	0	0	0	0	0	0.402	0	0.908	0.943
ELANE	MMP1	4450799	4439553	9606.ENSPC	9606.ENSPC	0	0	0	0	0.052	0	0.9	0.45	0.943
NR3C2	NR3C1	4442077	4433539	9606.ENSPC	9606.ENSPC	0	0	0	0.861	0	0.379	0.9	0.942	0.943
RNASE2	CTSG	4437950	4432948	9606.ENSPC	9606.ENSPC	0	0	0	0	0.333	0	0.9	0.205	0.942
CRABP2	RXRA	4451723	4449061	9606.ENSPC	9606.ENSPC	0	0	0	0	0	0.064	0.9	0.432	0.942
ADK	AHCY	4436683	4433021	9606.ENSPC	9606.ENSPC	0.135	0	0	0	0.389	0.28	0	0.863	0.941
NR3C1	PPP5C	4433539	4432591	9606.ENSPC	9606.ENSPC	0	0	0	0	0	0.311	0.9	0.225	0.941
CHEK1	TYMS	4447561	4439011	9606.ENSPC	9606.ENSPC	0	0	0	0	0.811	0.275	0	0.597	0.94
HSP90AB1	NR3C2	4443770	4442077	9606.ENSPC	9606.ENSPC	0	0	0	0	0	0.311	0.9	0.201	0.94
HSP90AA1	IL2	4440643	4433376	9606.ENSPC	9606.ENSPC	0	0	0	0	0	0	0.9	0.416	0.939
PNP	TK1	4442485	4437779	9606.ENSPC	9606.ENSPC	0.21	0	0	0	0.081	0	0.9	0.239	0.937
HSPA8	AR	4449616	4444436	9606.ENSPC	9606.ENSPC	0	0	0	0	0	0.109	0.9	0.348	0.936
MMP1	CTSG	4439553	4432948	9606.ENSPC	9606.ENSPC	0	0	0	0	0	0	0.9	0.389	0.936
MMP9	CTSS	4443919	4443237	9606.ENSPC	9606.ENSPC	0	0	0	0	0.134	0	0.9	0.319	0.935
LYZ	MMP8	4434847	4433667	9606.ENSPC	9606.ENSPC	0	0	0	0	0.116	0	0.9	0.325	0.935
NQO1	ADK	4439310	4436683	9606.ENSPC	9606.ENSPC	0	0	0	0	0	0.869	0	0.527	0.935
NMNAT1	BST1	4444851	4435534	9606.ENSPC	9606.ENSPC	0	0	0	0	0	0	0.9	0.377	0.935
ERBB4	EGFR	4441238	4436186	9606.ENSPC	9606.ENSPC	0	0	0	0.946	0	0.347	0.9	0.905	0.935
ELANE	LYZ	4450799	4434847	9606.ENSPC	9606.ENSPC	0	0	0	0	0.112	0	0.9	0.323	0.934
GART	TYMS	4445605	4439011	9606.ENSPC	9606.ENSPC	0.291	0	0	0	0.14	0	0	0.899	0.933
RXRA	KAT2B	4449061	4435311	9606.ENSPC	9606.ENSPC	0	0	0	0	0.061	0.194	0.9	0.222	0.933
RNASE2	LYZ	4437950	4434847	9606.ENSPC	9606.ENSPC	0	0	0	0	0.263	0	0.9	0.171	0.933
AKT2	NOS3	4445979	4437409	9606.ENSPC	9606.ENSPC	0	0	0	0	0.049	0.065	0.9	0.326	0.932
CDK2	AURKA	4435732	4432988	9606.ENSPC	9606.ENSPC	0	0	0.431	0.76	0.303	0.88	0	0.587	0.932
LCN2	LYZ	4444080	4434847	9606.ENSPC	9606.ENSPC	0	0	0	0	0.101	0	0.9	0.297	0.931
HSPA8	PGR	4449616	4439740	9606.ENSPC	9606.ENSPC	0	0	0	0	0	0.183	0.9	0.229	0.931
MMP9	LYZ	4443919	4434847	9606.ENSPC	9606.ENSPC	0	0	0	0	0.134	0	0.9	0.251	0.929
ESR1	PPP5C	4448358	4432591	9606.ENSPC	9606.ENSPC	0	0	0	0	0	0.324	0.9	0	0.929
DHFR	TPH1	4447922	4434115	9606.ENSPC	9606.ENSPC	0	0	0	0	0.061	0	0.9	0.289	0.927
HCK	F2	4450141	4438421	9606.ENSPC	9606.ENSPC	0	0	0	0	0	0.05	0.9	0.287	0.926
CHEK1	CDK7	4447561	4434485	9606.ENSPC	9606.ENSPC	0	0	0.31	0.642	0.062	0	0.9	0.493	0.926
ESR1	NR3C1	4448358	4433539	9606.ENSPC	9606.ENSPC	0	0	0	0.66	0	0	0.9	0.751	0.925
RXRA	ACADM	4449061	4443625	9606.ENSPC	9606.ENSPC	0	0	0	0	0.061	0	0.9	0.268	0.925
PNP	BST1	4442485	4435534	9606.ENSPC	9606.ENSPC	0	0	0	0	0	0	0.9	0.271	0.924
RXRA	NR3C1	4449061	4433539	9606.ENSPC	9606.ENSPC	0	0	0	0.632	0	0	0.9	0.641	0.923
RXRA	PCK1	4449061	4439313	9606.ENSPC	9606.ENSPC	0	0	0	0	0.061	0.05	0.9	0.239	0.923
MMP9	EPHB4	4443919	4442085	9606.ENSPC	9606.ENSPC	0	0	0	0	0	0	0.9	0.269	0.923
EPHA2	EPHB4	4442119	4442085	9606.ENSPC	9606.ENSPC	0	0	0	0.919	0.126	0.128	0.9	0.691	0.921
IGF1R	MDM2	4435797	4434604	9606.ENSPC	9606.ENSPC	0	0	0	0	0	0.56	0.6	0.589	0.921
CTSS	MMP8	4443237	4433667	9606.ENSPC	9606.ENSPC	0	0	0	0	0.09	0	0.9	0.194	0.92
MMP9	MMP13	4443919	4434767	9606.ENSPC	9606.ENSPC	0	0	0	0.797	0.064	0	0.9	0.878	0.919
AR	PGR	4444436	4439740	9606.ENSPC	9606.ENSPC	0	0	0	0.813	0.069	0	0.9	0.904	0.919
LCN2	MMP9	4444080	4443919	9606.ENSPC	9606.ENSPC	0	0	0	0	0.095	0.379	0	0.867	0.919
HSPA8	NR3C2	4449616	4442077	9606.ENSPC	9606.ENSPC	0	0	0	0	0	0.109	0.9	0.163	0.919
AKR1C3	SULT2B1	4445414	4432786	9606.ENSPC	9606.ENSPC	0	0	0	0	0	0	0.9	0.221	0.918
RARG	KAT2B	4447554	4435311	9606.ENSPC	9606.ENSPC	0	0	0	0	0	0.194	0.9	0.043	0.916
MAPK10	NR3C1	4442217	4433539	9606.ENSPC	9606.ENSPC	0	0	0	0	0.051	0	0.9	0.179	0.915
VDR	RARG	4450226	4447554	9606.ENSPC	9606.ENSPC	0	0	0	0.705	0.061	0	0.9	0.466	0.914
NR3C2	PGR	4442077	4439740	9606.ENSPC	9606.ENSPC	0	0	0	0.809	0.061	0	0.9	0.663	0.914
CHEK1	KIT	4447561	4436763	9606.ENSPC	9606.ENSPC	0	0	0	0.554	0	0.057	0.9	0.296	0.913
TYMS	KIF11	4439011	4434801	9606.ENSPC	9606.ENSPC	0	0	0	0	0.897	0	0	0.181	0.912
PGR	NR3C1	4439740	4433539	9606.ENSPC	9606.ENSPC	0	0	0	0.839	0	0	0.9	0.78	0.912
HCK	ITK	4450141	4448039	9606.ENSPC	9606.ENSPC	0	0	0	0.845	0.081	0	0.9	0.501	0.911
MMP3	MMP13	4437610	4434767	9606.ENSPC	9606.ENSPC	0	0	0.249	0.944	0.085	0	0.9	0.893	0.91
MMP1	MMP13	4439553	4434767	9606.ENSPC	9606.ENSPC	0	0	0	0.935	0.077	0	0.9	0.863	0.909
MAPK10	BCL2L1	4442217	4437866	9606.ENSPC	9606.ENSPC	0	0	0	0	0	0.053	0.8	0.554	0.908
HCK	EPHA2	4450141	4442119	9606.ENSPC	9606.ENSPC	0	0	0	0.77	0.061	0	0.9	0.255	0.907
TYMS	CCNA2	4439011	4436109	9606.ENSPC	9606.ENSPC	0	0	0	0	0.838	0	0	0.447	0.907
RARG	CDK7	4447554	4434485	9606.ENSPC	9606.ENSPC	0	0	0	0	0	0	0.9	0.111	0.907
CDA	MMP9	4444499	4443919	9606.ENSPC	9606.ENSPC	0	0	0	0	0.089	0	0.9	0.07	0.907
RARG	THRB	4447554	4446585	9606.ENSPC	9606.ENSPC	0	0	0	0.814	0	0	0.9	0.426	0.907
PDE4D	ADK	4441530	4436683	9606.ENSPC	9606.ENSPC	0	0	0	0	0	0	0.9	0.09	0.905
PLAU	ITGAL	4444017	4441903	9606.ENSPC	9606.ENSPC	0	0	0	0	0	0	0.9	0.089	0.905
CDA	LYZ	4444499	4434847	9606.ENSPC	9606.ENSPC	0	0	0	0	0.095	0	0.9	0	0.905
ITGAL	BST1	4441903	4435534	9606.ENSPC	9606.ENSPC	0	0	0	0	0.085	0	0.9	0	0.904
TYMS	AURKA	4439011	4432988	9606.ENSPC	9606.ENSPC	0	0	0	0	0.86	0	0	0.339	0.904
CLEC4M	CD209	4439046	4439000	9606.ENSPC	9606.ENSPC	0	0	0	0.979	0	0.534	0.8	0.89	0.904
GP1BA	KAT2B	4440098	4435311	9606.ENSPC	9606.ENSPC	0	0	0	0	0.062	0.052	0.9	0	0.903
APRT	PDE4D	4445041	4441530	9606.ENSPC	9606.ENSPC	0	0	0	0	0	0	0.9	0.079	0.903
CDA	MMP8	4444499	4433667	9606.ENSPC	9606.ENSPC	0	0	0	0	0.069	0	0.9	0	0.902
CDA	CTSS	4444499	4443237	9606.ENSPC	9606.ENSPC	0	0	0	0	0.065	0	0.9	0	0.902
PLAU	BST1	4444017	4435534	9606.ENSPC	9606.ENSPC	0	0	0	0	0.061	0	0.9	0	0.902
ELANE	IMPDH1	4450799	4441488	9606.ENSPC	9606.ENSPC	0	0	0	0	0	0	0.9	0.067	0.902
IMPDH1	LYZ	4441488	4434847	9606.ENSPC	9606.ENSPC	0	0	0	0	0	0	0.9	0	0.9
IMPDH1	RNASE2	4441488	4437950	9606.ENSPC	9606.ENSPC	0	0	0	0	0	0	0.9	0	0.9
IMPDH1	CTSG	4441488	4432948	9606.ENSPC	9606.ENSPC	0	0	0	0	0	0	0.9	0	0.9

CHEK1	HSP90AA1	4447561	4440643	9606.ENSPC	9606.ENSPC	0	0	0	0	0.062	0.416	0	0.803	0.883
AKT2	BCL2L1	4445979	4437866	9606.ENSPC	9606.ENSPC	0	0	0	0	0.05	0	0.8	0.429	0.882
CHEK1	KIF11	4447561	4434801	9606.ENSPC	9606.ENSPC	0	0	0	0	0.821	0	0	0.359	0.88
HCK	WAS	4450141	4444767	9606.ENSPC	9606.ENSPC	0	0	0	0	0.314	0.472	0	0.694	0.879
CYP19A1	AR	4446543	4444436	9606.ENSPC	9606.ENSPC	0	0	0	0	0	0.085	0	0.873	0.879
CTSS	CTSB	4443237	4441541	9606.ENSPC	9606.ENSPC	0	0	0.223	0.675	0.103	0	0.8	0.77	0.868
PGF	TEK	4450339	4445310	9606.ENSPC	9606.ENSPC	0	0	0	0	0	0.058	0.6	0.673	0.866
EGFR	MDM2	4436186	4434604	9606.ENSPC	9606.ENSPC	0	0	0	0	0	0	0.6	0.671	0.862
BCL2L1	CDK2	4437866	4435732	9606.ENSPC	9606.ENSPC	0	0	0	0	0.05	0.13	0	0.845	0.861
TNK2	AR	4445619	4444436	9606.ENSPC	9606.ENSPC	0	0	0	0	0	0.379	0	0.781	0.858
AKT2	HSP90AB1	4445979	4443770	9606.ENSPC	9606.ENSPC	0	0	0	0	0.061	0.584	0.6	0.201	0.858
CYP19A1	PGR	4446543	4439740	9606.ENSPC	9606.ENSPC	0	0	0	0	0	0.085	0	0.851	0.857
DHFR	DHODH	4447922	4433071	9606.ENSPC	9606.ENSPC	0	0	0	0	0.08	0	0	0.849	0.855
ESR1	MAPK10	4448358	4442217	9606.ENSPC	9606.ENSPC	0	0	0	0	0	0	0.8	0.293	0.852
SDS	AHCY	4434551	4433021	9606.ENSPC	9606.ENSPC	0.043	0	0	0	0.116	0	0.8	0.221	0.85
ADAM17	EGFR	4438549	4436186	9606.ENSPC	9606.ENSPC	0	0	0	0	0.061	0	0	0.842	0.846
CHEK1	AURKA	4447561	4432988	9606.ENSPC	9606.ENSPC	0	0	0.288	0.675	0.754	0.162	0	0.672	0.845
BCL2L1	ANXA5	4437866	4437297	9606.ENSPC	9606.ENSPC	0	0	0	0	0	0	0	0.837	0.837
GLO1	SDS	4444169	4434551	9606.ENSPC	9606.ENSPC	0	0	0	0	0	0.055	0.8	0.203	0.836
GART	DHODH	4445605	4433071	9606.ENSPC	9606.ENSPC	0.37	0	0	0	0.311	0.057	0	0.644	0.835
GART	IMPDH1	4445605	4441488	9606.ENSPC	9606.ENSPC	0.353	0	0	0	0.261	0	0	0.683	0.835
GART	APRT	4445605	4445041	9606.ENSPC	9606.ENSPC	0.367	0	0	0	0.082	0.076	0	0.724	0.832
MMP9	EGFR	4443919	4436186	9606.ENSPC	9606.ENSPC	0	0	0	0	0	0	0	0.828	0.828
TK1	CCNA2	4437779	4436109	9606.ENSPC	9606.ENSPC	0	0	0	0	0.698	0	0	0.454	0.828
TYMS	CDK2	4439011	4435732	9606.ENSPC	9606.ENSPC	0	0	0	0	0.686	0	0	0.467	0.825
MAPK10	PRKCQ	4442217	4435203	9606.ENSPC	9606.ENSPC	0	0	0	0.588	0	0.157	0.8	0.048	0.824
HSP90AB1	EGFR	4443770	4436186	9606.ENSPC	9606.ENSPC	0	0	0	0	0	0.487	0	0.668	0.823
MAOB	GLO1	4444997	4444169	9606.ENSPC	9606.ENSPC	0	0	0	0	0	0	0.8	0.133	0.819
MAPK10	PGR	4442217	4439740	9606.ENSPC	9606.ENSPC	0	0	0	0	0.08	0	0.8	0.082	0.816
PDE5A	NOS3	4441685	4437409	9606.ENSPC	9606.ENSPC	0	0	0	0	0	0	0	0.814	0.814
PGR	EGFR	4439740	4436186	9606.ENSPC	9606.ENSPC	0	0	0	0	0	0.077	0	0.804	0.811
EGFR	IL2	4436186	4433376	9606.ENSPC	9606.ENSPC	0	0	0	0	0	0	0.6	0.548	0.811
CDK2	KIF11	4435732	4434801	9606.ENSPC	9606.ENSPC	0	0	0	0	0.373	0.162	0	0.666	0.809
ELANE	MMP9	4450799	4443919	9606.ENSPC	9606.ENSPC	0	0	0	0	0.094	0	0	0.794	0.805
MTAP	APRT	4445335	4445041	9606.ENSPC	9606.ENSPC	0.219	0	0	0	0.064	0	0	0.753	0.803
AKR1C2	MIF	4445450	4432873	9606.ENSPC	9606.ENSPC	0	0	0	0	0	0.794	0	0.086	0.803
AR	EGFR	4444436	4436186	9606.ENSPC	9606.ENSPC	0	0	0	0	0.062	0.402	0	0.678	0.803
EGFR	LGALS3	4436186	4434352	9606.ENSPC	9606.ENSPC	0	0	0	0	0.076	0.379	0	0.685	0.803
CASP7	BCL2L1	4443299	4437866	9606.ENSPC	9606.ENSPC	0	0	0	0	0	0.168	0	0.765	0.796
ADH1C	GSTM1	4449347	4438694	9606.ENSPC	9606.ENSPC	0	0	0	0	0.061	0	0.65	0.428	0.795
GSTM1	ADH1B	4438694	4438237	9606.ENSPC	9606.ENSPC	0	0	0	0	0.061	0	0.65	0.413	0.79
MMP9	CTSK	4443919	4435989	9606.ENSPC	9606.ENSPC	0	0	0	0	0.089	0	0	0.779	0.79
CASP7	BIRC7	4443299	4433001	9606.ENSPC	9606.ENSPC	0	0	0	0	0.048	0.57	0	0.528	0.79
GSTP1	CYP2C8	4446837	4443714	9606.ENSPC	9606.ENSPC	0	0	0	0	0	0.077	0.65	0.399	0.789
PGF	KIT	4450339	4436763	9606.ENSPC	9606.ENSPC	0	0	0	0	0	0.058	0.6	0.481	0.787
PPARG	EGFR	4436745	4436186	9606.ENSPC	9606.ENSPC	0	0	0	0	0.062	0.402	0	0.65	0.787
CYP2C8	GSTM1	4443714	4438694	9606.ENSPC	9606.ENSPC	0	0	0	0	0	0.077	0.65	0.391	0.786
PIK3CG	KIT	4442215	4436763	9606.ENSPC	9606.ENSPC	0	0	0	0	0	0.719	0	0.267	0.785
AR	IGF1R	4444436	4435797	9606.ENSPC	9606.ENSPC	0	0	0	0	0.051	0.085	0	0.77	0.783
MMP9	NOS3	4443919	4437409	9606.ENSPC	9606.ENSPC	0	0	0	0	0.062	0	0	0.778	0.783
PGF	EGFR	4450339	4436186	9606.ENSPC	9606.ENSPC	0	0	0	0	0	0	0.6	0.473	0.78
ESR1	AKT2	4448358	4445979	9606.ENSPC	9606.ENSPC	0	0	0	0	0	0.407	0	0.638	0.777
CASP1	BCL2L1	4449658	4437866	9606.ENSPC	9606.ENSPC	0	0	0	0	0	0.461	0	0.596	0.773
AKR1C3	AR	4445414	4444436	9606.ENSPC	9606.ENSPC	0	0	0	0	0	0.05	0	0.769	0.772
APRT	TK1	4445041	4437779	9606.ENSPC	9606.ENSPC	0.174	0	0	0	0.136	0	0	0.699	0.767
APRT	CDA	4445041	4444499	9606.ENSPC	9606.ENSPC	0.23	0	0	0	0	0	0	0.704	0.762
NOS3	PPARG	4437409	4436745	9606.ENSPC	9606.ENSPC	0	0	0	0	0.053	0.091	0	0.741	0.757
DHFR	TK1	4447922	4437779	9606.ENSPC	9606.ENSPC	0.133	0	0	0	0.227	0	0	0.664	0.756
GART	CDA	4445605	4444499	9606.ENSPC	9606.ENSPC	0.326	0	0	0	0	0	0	0.653	0.756
ESR1	ERBB4	4448358	4441238	9606.ENSPC	9606.ENSPC	0	0	0	0	0	0.402	0	0.605	0.754
MMP9	LGALS3	4443919	4434352	9606.ENSPC	9606.ENSPC	0	0	0	0	0.071	0	0	0.743	0.751
AR	MET	4444436	4439114	9606.ENSPC	9606.ENSPC	0	0	0	0	0	0.085	0	0.738	0.75
CTSB	BCL2L1	4441541	4437866	9606.ENSPC	9606.ENSPC	0	0	0	0	0.044	0	0.6	0.398	0.749
PGF	MMP9	4450339	4443919	9606.ENSPC	9606.ENSPC	0	0	0	0	0	0	0	0.748	0.748
HSPA8	PPP5C	4449616	4432591	9606.ENSPC	9606.ENSPC	0	0	0	0	0.087	0.235	0.54	0.304	0.746
TNK2	WAS	4445619	4444767	9606.ENSPC	9606.ENSPC	0	0	0	0	0.065	0.379	0	0.597	0.746
FGFR2	MDM2	4448546	4434604	9606.ENSPC	9606.ENSPC	0	0	0	0	0	0	0.6	0.389	0.745
NQO1	GSTM1	4439310	4438694	9606.ENSPC	9606.ENSPC	0	0	0	0	0.057	0	0	0.738	0.743
GSTP1	ADH1B	4446837	4438237	9606.ENSPC	9606.ENSPC	0	0	0	0	0	0	0.65	0.296	0.743
ADH1C	GSTP1	4449347	4446837	9606.ENSPC	9606.ENSPC	0	0	0	0	0	0	0.65	0.291	0.741
ANXA5	EGFR	4437297	4436186	9606.ENSPC	9606.ENSPC	0	0	0	0	0.065	0	0	0.731	0.738
GPI	SORD	4448366	4435781	9606.ENSPC	9606.ENSPC	0.07	0	0	0	0.064	0.413	0	0.548	0.738
BMP2	MMP13	4445120	4434767	9606.ENSPC	9606.ENSPC	0	0	0	0	0	0	0	0.736	0.736
HSP90AA1	CDK2	4440643	4435732	9606.ENSPC	9606.ENSPC	0	0	0	0	0.054	0.414	0	0.561	0.735
ADH1C	MAOB	4449347	4444997	9606.ENSPC	9606.ENSPC	0	0	0	0	0.092	0	0.65	0.23	0.734
PGF	MET	4450339	4439114	9606.ENSPC	9606.ENSPC	0	0	0	0	0	0	0.6	0.364	0.734
PGF	FGFR1	4450339	4447790	9606.ENSPC	9606.ENSPC	0	0	0	0	0	0.058	0.6	0.348	0.732
MMP1	LGALS3	4439553	4434352	9606.ENSPC	9606.ENSPC	0	0	0	0	0.097	0	0	0.715	0.732
CASP7	ANXA5	4443299	4437297	9606.ENSPC	9606.ENSPC	0	0	0	0	0	0	0	0.732	0.732
BCL2L1	MDM2	4437866	4434604	9606.ENSPC	9606.ENSPC	0	0	0	0	0.055	0.189	0	0.675	0.729
TK1	AURKA	4437779	4432988	9606.ENSPC	9606.ENSPC	0	0	0	0	0.618	0	0	0.31	0.725
PLAU	MMP9	4444017	4443919	9606.ENSPC	9606.ENSPC	0	0	0	0	0.076	0	0	0.713	0.724
CDA	TYMS	4444499	4439011	9606.ENSPC	9606.ENSPC	0.052	0	0	0	0.061	0	0	0.713	0.723
GSTP1	NQO1	4446837	4439310	9606.ENSPC	9606.ENSPC	0	0	0	0	0.064	0	0	0.717	0.723
ESR1	MDM2	4448358	4434604	9606.ENSPC	9606.ENSPC	0	0	0	0	0	0.393	0	0.563	0.723
TK1	KIF11	4437779	4434801	9606.ENSPC	9606.ENSPC	0	0	0	0	0.508	0	0	0.46	0.723
CASP1	CTSB	4449658	4441541	9606.ENSPC	9606.ENSPC	0	0	0	0	0.107	0	0	0.703	0.723
PLAU	CTSB	4444017	4441541	9606.ENSPC	9606.ENSPC	0	0	0	0	0.077	0.379	0	0.553	0.721
AR	MDM2	4444436	4434604	9606.ENSPC	9606.ENSPC	0	0	0	0	0	0.393	0	0.557	0.72
GSTP1	GSTM1	4446837	4438694	9606.ENSPC	9606.ENSPC	0	0	0	0.782	0	0	0.65	0.927	0.72
ERBB4	PGR	4441238	4439740	9606.ENSPC	9606.ENSPC	0	0	0	0	0.071	0.077	0	0.7	0.72
ESR1	CDK2	4448358	4435732	9606.ENSPC	9606.ENSPC	0	0	0	0	0	0.354	0	0.582	0.718
AR	MMP9	4444436	4443919	9606.ENSPC	9606.ENSPC	0	0	0	0	0	0	0	0.716	0.717

THRB	KAT2B	4446585	4435311	9606.ENSPC	9606.ENSPC	0	0	0	0	0.061	0.194	0.6	0.177	0.717
MET	EGFR	4439114	4436186	9606.ENSPC	9606.ENSPC	0	0	0	0.58	0.229	0.418	0	0.933	0.715
AKT2	CCNA2	4445979	4436109	9606.ENSPC	9606.ENSPC	0	0	0	0	0	0.354	0	0.571	0.711
CHEK1	HSP90AB1	4447561	4443770	9606.ENSPC	9606.ENSPC	0	0	0	0	0.062	0.416	0	0.513	0.71
MAOB	BCHE	4444997	4435424	9606.ENSPC	9606.ENSPC	0.045	0	0	0	0.086	0	0	0.694	0.71
CYP2C8	GSTM2	4443714	4433798	9606.ENSPC	9606.ENSPC	0	0	0	0	0	0.077	0.65	0.176	0.71
MMP9	PPARG	4443919	4436745	9606.ENSPC	9606.ENSPC	0	0	0	0	0	0	0	0.709	0.709
MAOB	ADH1B	4444997	4438237	9606.ENSPC	9606.ENSPC	0	0	0	0	0.104	0	0.65	0.142	0.707
EPHA2	HSP90AA1	4442119	4440643	9606.ENSPC	9606.ENSPC	0	0	0	0	0.048	0.244	0	0.625	0.707
HSP90AA1	MDM2	4440643	4434604	9606.ENSPC	9606.ENSPC	0	0	0	0	0	0.348	0	0.568	0.706
AR	BCL2L1	4444436	4437866	9606.ENSPC	9606.ENSPC	0	0	0	0	0	0.064	0	0.698	0.706
GSTP1	GSTM2	4446837	4433798	9606.ENSPC	9606.ENSPC	0	0	0	0.803	0	0	0.65	0.801	0.704
HSP90AA1	MET	4440643	4439114	9606.ENSPC	9606.ENSPC	0	0	0	0	0	0.452	0	0.483	0.704
PGF	IGF1R	4450339	4435797	9606.ENSPC	9606.ENSPC	0	0	0	0	0	0	0.6	0.287	0.702
F2	BCHE	4438421	4435424	9606.ENSPC	9606.ENSPC	0	0	0	0	0.126	0.052	0	0.669	0.701

Table 6S

Top 15 in network 0.7PPIstring_interactions ranked by degree method

Rank	Name	Score
1	EGFR	20
2	MMP9	19
3	HSP90AA1	18
4	AR	17
5	ESR1	15
6	MDM2	13
7	HSP90AB1	12
8	TYMS	11
8	CDK2	11
8	RXRA	11
8	PGR	11
8	NR3C1	11
13	CCNA2	10
13	CHEK1	10
15	BCL2L1	9

Table 7S

Pw	Count	PValue	FDR
cytosol	77	4.21E-20	5.30E-17
extracellu	58	6.52E-12	8.22E-09
extracellu	40	3.12E-10	3.93E-07
extracellu	35	2.02E-09	2.54E-06
cytoplasm	68	1.19E-05	0.0149911
nucleoplas	44	1.40E-05	0.0175977
cell surfa	16	3.60E-05	0.0453097
endolysoso	3	3.89E-04	0.4890617
receptor c	7	5.98E-04	0.7513211
extracellu	10	7.13E-04	0.8944314
focal adhe	10	0.00471	5.7772062
mitochondr	9	0.0053124	6.4934207
membrane	30	0.005562	6.788777
myelin she	6	0.0080481	9.6837811
melanosome	5	0.0092819	11.089567
cell-cell	6	0.0132426	15.467336
intracellu	11	0.015806	18.193978
basolatera	6	0.0158392	18.228727
cyclin A2-	2	0.0161768	18.581572
nucleus	57	0.0203152	22.794604
nuclear ch	6	0.0207427	23.218127
mitochondr	19	0.0226223	25.055208
ruffle mem	4	0.0291687	31.142207
RNA polyme	3	0.0293451	31.299724
apical pla	7	0.0316746	33.34926
lysosomal	4	0.0319701	33.605116
lysosome	6	0.0373457	38.105618
spindle mi	3	0.0496366	47.360724
leading ed	2	0.0631641	56.062857
proteinace	6	0.0677539	58.700332

Table 8S

Pw	Count	PValue	FDR
steroid hc	13	5.71E-14	8.15E-11
serine-typ	17	9.39E-10	1.34E-06
transmembr	9	1.19E-09	1.70E-06
ATP bindir	38	5.48E-09	7.83E-06
protein ty	12	2.62E-08	3.75E-05
zinc ion b	32	2.65E-08	3.79E-05
identical	24	1.71E-07	2.44E-04
drug bindi	9	3.46E-07	4.95E-04
RNA polyme	7	6.69E-07	9.56E-04
endopeptic	7	7.79E-06	0.0111211
enzyme bir	14	8.15E-06	0.0116465
protein ki	14	1.81E-05	0.025886
oxidoreduc	4	3.65E-05	0.0521733
nitric-oxi	4	3.65E-05	0.0521733
steroid bi	5	8.73E-05	0.1246917
cysteine-t	6	2.00E-04	0.2855515
phosphatic	6	2.16E-04	0.3083788
ketosteroi	3	2.31E-04	0.3291885
retinoid X	4	2.84E-04	0.4044211
cysteine-t	5	3.41E-04	0.485572
oxidoreduc	9	4.04E-04	0.5756161
phenanthre	3	4.59E-04	0.6535665
trans-1,2-	3	4.59E-04	0.6535665
metalloenc	7	4.97E-04	0.7079339
Ras guanyl	7	5.46E-04	0.7768751
protein hc	17	7.32E-04	1.0401524
kinase act	9	0.0013605	1.9258602
alditol:NA	3	0.0015786	2.2313228
carboxylic	3	0.0015786	2.2313228
collagen b	5	0.0019372	2.7317817
bile acid	3	0.0020926	2.9478305
sequence-s	13	0.0021673	3.0515356
protein bi	96	0.0024633	3.4614612
virion bir	3	0.0033244	4.6449806
receptor s	3	0.0033244	4.6449806
glutathior	3	0.0040396	5.6178193
proteoglyc	3	0.0040396	5.6178193
receptor b	10	0.0041547	5.7735066
catalytic	7	0.0065051	8.9007705
MHC class	3	0.0085628	11.558993
scaffold p	4	0.0087198	11.758923
transferas	5	0.0103444	13.802179
retinol de	3	0.0107924	14.357869
iron ion b	6	0.0115478	15.287367
monooxyger	4	0.014595	18.942125
CTP bindir	2	0.0175756	22.374699
protein ph	4	0.0181958	23.071764
protein ki	9	0.0185702	23.489676
protein se	9	0.0185702	23.489676

glycoprote	4	0.019762	24.806138
indanol de	2	0.026248	31.608829
ICAM-3 rec	2	0.026248	31.608829
UTP bindir	2	0.026248	31.608829
sulfonylur	2	0.026248	31.608829
vitamin D	2	0.026248	31.608829
ATPase bir	4	0.0277036	33.054738
carbohydra	6	0.0300332	35.309726
nucleoside	2	0.0348443	39.744942
dATP bindi	2	0.0348443	39.744942
Rho GDP-di	2	0.0348443	39.744942
metallopep	4	0.0348878	39.783707
cyclin-dep	3	0.036107	40.861167
glutathior	3	0.0380779	42.565169
ubiquitin	7	0.0420763	45.882689
TPR domair	2	0.0433653	46.913548
fibroblast	2	0.0433653	46.913548
arachidoni	2	0.0433653	46.913548
retinoic a	2	0.0433653	46.913548
electron c	4	0.0453963	48.500901
peptidase	4	0.0453963	48.500901
alcohol de	2	0.0518115	53.22965
arylestera	2	0.0518115	53.22965
heat shock	3	0.0529549	54.028843
heparin bi	5	0.0534475	54.3692
growth fac	5	0.0550198	55.439985
1-phosphat	3	0.0552247	55.577812
small mole	2	0.0601837	58.794588
non-membra	3	0.0622336	60.05996
retinoid b	2	0.0684824	63.697659
thyroid hc	2	0.0684824	63.697659
unfolded p	4	0.0736087	66.449238
ion channe	4	0.0783823	68.835896
retinal bi	2	0.0848621	71.823597
ephrin rec	2	0.0929443	75.176896
peptide bi	3	0.0956377	76.209206

Table 9S

Pw	Count	PValue	FDR
steroid hc	11	6.31E-11	1.06E-07
negative r	20	1.95E-08	3.28E-05
positive r	18	7.70E-07	0.0012942
positive r	7	1.30E-05	0.0218364
response t	13	1.62E-05	0.0272914
positive r	8	1.33E-04	0.2226868
glucose me	6	3.09E-04	0.518887
positive r	10	5.31E-04	0.8898674
positive r	6	8.77E-04	1.4649836
response t	6	9.26E-04	1.5450522
positive r	5	0.0013016	2.1663835
endocytosi	7	0.0014537	2.4167745
innate imm	12	0.0015107	2.5102745
positive r	4	0.0020723	3.4283755
daunorubic	3	0.0020866	3.4516013
doxorubici	3	0.0020866	3.4516013
mesenchyma	3	0.0020866	3.4516013
angiogenes	8	0.0036501	5.9642369
regulation	3	0.0040281	6.5623449
response t	7	0.0042126	6.8530181
positive r	7	0.0045768	7.4242074
cell proli	10	0.0051941	8.3850301
positive r	5	0.0057026	9.1692703
positive r	7	0.0058311	9.3664356
negative r	14	0.0112413	17.313224
nucleoside	3	0.0119594	18.31734
G1/S trans	5	0.0126462	19.26696
negative r	4	0.0132274	20.062381
cellular c	3	0.0145192	21.804169
regulation	4	0.0181254	26.479321
Notch sigr	5	0.0188582	27.396706
leukocyte	3	0.0202717	29.135693
cellular p	5	0.0205175	29.434061
drug metab	3	0.0234481	32.901895
regulation	4	0.0239022	33.424563
negative r	9	0.0242139	33.781283
regulation	6	0.0243397	33.924619
regulation	3	0.025108	34.794227
Fc-gamma r	5	0.0260224	35.815149
apoptotic	11	0.0286708	38.687859
G2/M trans	5	0.0330569	43.181063
cell surfa	7	0.034598	44.684902
regulation	2	0.0347946	44.873992
cell divis	8	0.0356054	45.647567
extrinsic	3	0.036011	46.030749
positive r	10	0.0388349	48.628704
deoxyribor	2	0.0433037	52.500831
mitotic ce	2	0.0433037	52.500831
negative r	3	0.0441129	53.17199

negative r	3	0.0483932	56.576099
pyrimidine	2	0.0517382	59.07284
tetrahydro	2	0.0517382	59.07284
positive r	3	0.0528178	59.849383
cellular r	4	0.0529858	59.968989
T cell act	3	0.0644692	67.394155
humoral im	3	0.0899906	79.521824
regulation	4	0.0966711	81.908317

Table 10S

Pw	Count	PValue	FDR
Pathways i	26	1.82E-08	2.30E-05
PI3K-Akt s	21	2.57E-06	0.0032423
Prostate c	11	3.22E-06	0.0040628
Progesterc	9	1.49E-04	0.188181
Ras signal	14	1.84E-04	0.2315246
Melanoma	8	2.54E-04	0.3198339
Metabolism	8	3.28E-04	0.4138799
Rap1 signa	13	3.49E-04	0.4396727
Estrogen s	9	3.66E-04	0.4605876
Metabolic	38	4.98E-04	0.6265678
Proteoglyc	12	8.53E-04	1.0713798
Central ca	7	9.30E-04	1.1671398
Pancreatic	7	0.0010093	1.2667145
Drug metab	7	0.0012805	1.6045253
Measles	9	0.0025263	3.1428553
Transcript	10	0.0029123	3.6149281
Chemical c	7	0.0029547	3.6667422
Small cell	7	0.0040007	4.9347696
One carbon	4	0.0050922	6.2416961
Glioma	6	0.0059139	7.2144883
PPAR signa	6	0.0067222	8.1624407
Adherens j	6	0.0085656	10.291073
FoxO signa	8	0.0099392	11.847529
Thyroid hc	7	0.0167224	19.17578
Non-small	5	0.0174847	19.963265
Steroid hc	5	0.0196568	22.168547
Epstein-Ba	7	0.0217359	24.226829
Tyrosine m	4	0.0240853	26.492365
HIF-1 sigr	6	0.0283291	30.427264
Viral carc	9	0.0301066	32.016962
Epithelial	5	0.0314446	33.191488
T cell rec	6	0.0330034	34.536245
Pyrimidine	6	0.0342428	35.58764
Adipocytok	5	0.0361261	37.155535
Phenylalar	3	0.0361997	37.216102
Bladder ca	4	0.0363284	37.321809
Purine met	8	0.0378885	38.590841
Chronic my	5	0.0394595	39.844755
TNF signal	6	0.0422851	42.04094
Insulin re	6	0.0437275	43.133321
Hepatitis	7	0.0450725	44.134832
Antigen pr	5	0.0466373	45.279609
Fc gamma R	5	0.063027	56.038216
AMPK signa	6	0.0689064	59.396499
ErbB signa	5	0.0698614	59.919124
Cell cycle	6	0.0708211	60.438075
Rheumatoic	5	0.0722214	61.184175
Focal adhe	8	0.0754628	62.861844
NOD-like r	4	0.0781099	64.182102

Acute myel	4	0.0781099	64.182102
Regulation	8	0.0817074	65.90713
Biosynthes	8	0.0849394	67.391343
Nicotinate	3	0.0941881	71.316212
VEGF signa	4	0.0952433	71.735194
Arachidoni	4	0.0952433	71.735194
Apoptosis	4	0.0988413	73.121837
